# Control of Redox‐Active Ester Reactivity Enables a General Cross‐Electrophile Approach to Access Arylated Strained Rings**

**DOI:** 10.1002/anie.202205673

**Published:** 2022-07-05

**Authors:** Daniel C. Salgueiro, Benjamin K. Chi, Ilia A. Guzei, Pablo García‐Reynaga, Daniel J. Weix

**Affiliations:** ^1^ Department of Chemistry University of Wisconsin-Madison Madison WI 53706 USA; ^2^ Janssen Research & Development, LLC 3210 Merryfield Row, San Diego CA 92121 USA

**Keywords:** Cross-Coupling, Nickel Catalysis, Quaternary Centers, Redox-Active Esters, Strained Rings

## Abstract

Strained rings are increasingly important for the design of pharmaceutical candidates, but cross‐coupling of strained rings remains challenging. An attractive, but underdeveloped, approach to diverse functionalized carbocyclic and heterocyclic frameworks containing all‐carbon quaternary centers is the coupling of abundant strained‐ring carboxylic acids with abundant aryl halides. Herein we disclose the development of a nickel‐catalyzed cross‐electrophile approach that couples a variety of strained ring *N*‐hydroxyphthalimide (NHP) esters, derived from the carboxylic acid in one step, with various aryl and heteroaryl halides under reductive conditions. The chemistry is enabled by the discovery of methods to control NHP ester reactivity, by tuning the solvent or using modified NHP esters, and the discovery that ^*t‐*Bu^BpyCam^CN^, an L2X ligand, avoids problematic side reactions. This method can be run in flow and in 96‐well plates.

## Graphical abstract

Aliphatic strained rings can be (hetero)arylated through a decarboxylative cross‐electrophile coupling. The keys to this advance are: 1) a new ligand that enables tolerance of a variety of strained ring classes and 2) electronic tunability of redox‐active esters to control the rate of radical generation.

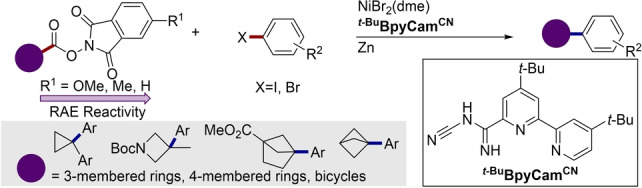

## Introduction

**Scheme 1:**
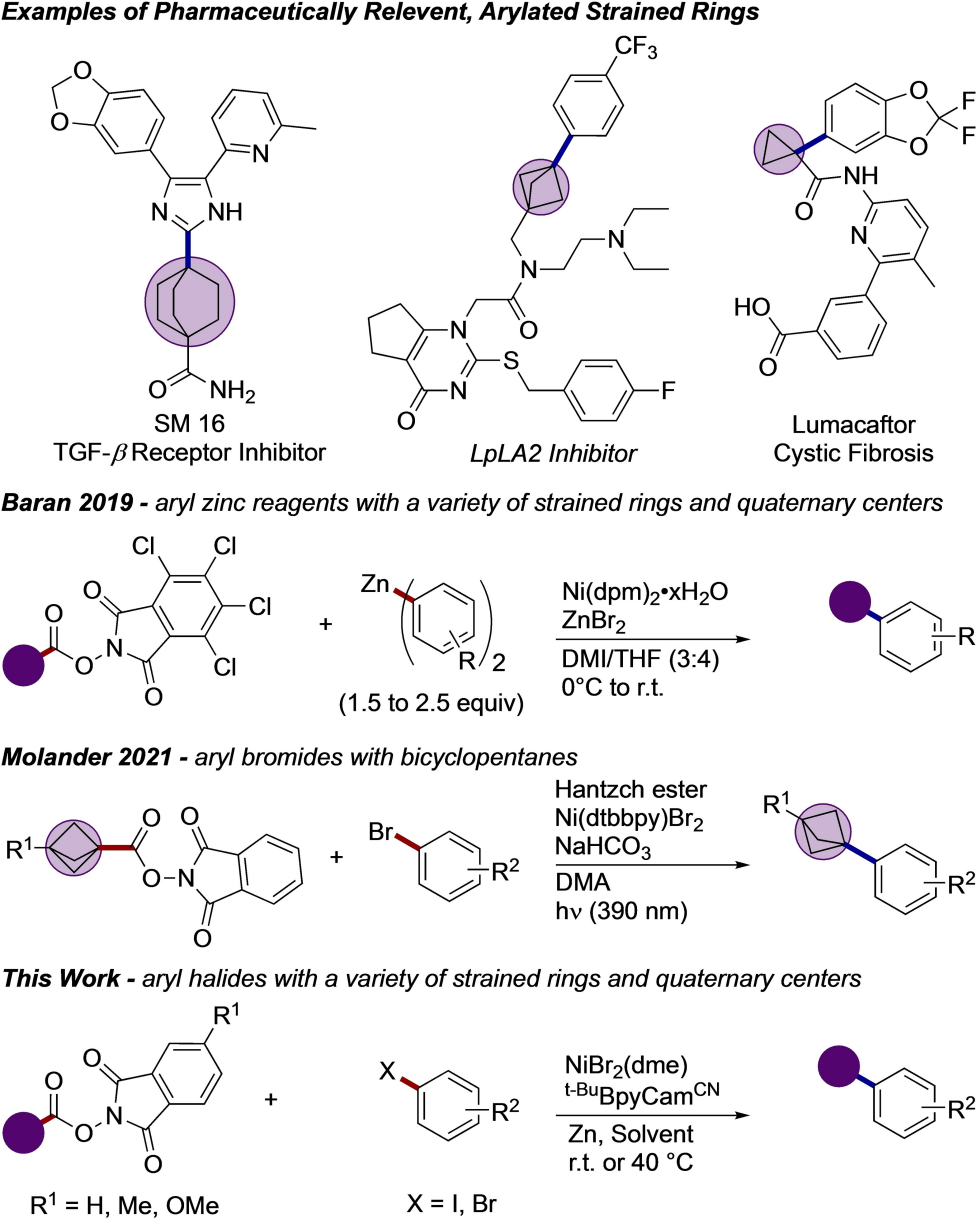
Arylation of strained rings using tuned redox‐active esters.

Molecules with strained rings, including 3‐ and 4‐membered carbocycles, have gained prominence in medicinal chemistry due to the beneficial effects they impart on the pharmacokinetic and pharmacodynamic properties of drug candidates (Scheme 1).^[1]^ These include improved solubility, metabolic stability, and receptor/ligand binding interactions.[^1^, ^2^] Most often, incorporation of strained rings into molecules is accomplished by a ring‐opening^[3]^ or ring‐closing reaction, typically involving a π‐system.^[4]^ These annulation reactions are well‐studied and can be performed in a stereoselective and regioselective fashion.[^4^, ^5^] However, each annulation reaction requires different conditions, and often require multiple steps, making parallel screening of different ring systems difficult.

An ideal strategy to enable the rapid access of these strained ring systems for medicinal chemistry would be a direct cross‐coupling approach that would allow access to large pools of coupling partners and be general for a variety of strained rings.^[6]^ Despite advances in strain‐release methodologies utilizing “spring‐loaded” reagents,^[7]^ and cross‐coupling of strained‐ring units,[^7i^, ^8^, ^9^] current approaches are limited by the availability of requisitely functionalized coupling partners and do not yet offer the substrate compatibility and scope needed to rapidly screen a variety of strained‐rings.[^7i^, ^8^] In general, decarboxylative approaches, be they oxidative,^[10]^ redox neutral,^[11]^ or reductive,^[12]^ would be the most attractive due to the widespread availability of strained‐ring‐containing carboxylic acids, owing to advancements in their syntheses, including strategies that directly furnish cyclorpropyl redox‐active esters.[^5^, ^8^, ^13^, ^14^] Recent studies by Baran^[11a]^ and Molander^[15]^ using *N*‐hydroxyphthalimide (NHP) esters and Huestis^[16]^ using carboxylates are attractive, but limited by the need for diarylzinc reagents (Baran) or were demonstrated for only bicyclo[1.1.1]pentane (Molander) and amino‐oxetane units (Huestis). A general set of conditions that tolerate (hetero)aryl halides and is suited to the incorporation of a variety of strained rings would be ideal.

In order to develop a general cross‐electrophile coupling of aryl halides with a variety of strained‐ring NHP esters, we had to address two major challenges. First, formation of all‐carbon quaternary centers by cross‐electrophile coupling remains challenging[^7d^, ^17^, ^18^] and a limited number of catalysts are reported to be effective. For tertiary radicals of strained rings, which have different catalyst requirements than unstrained tertiary radicals,^[19]^ 2,2′‐bipyridine, dtbbpy (**L1**), 4,4′‐dicarboxymethyl‐2,2′‐bipyridine (**L2**), bathophenanthroline (**L4**), as well as substituted pyridines and diketonate ligands have been reported to be effective for aryl[^11a^, ^15^, ^16^, ^18a^, ^18b^, ^18c^, ^18d^, ^18e^, ^18f^] and acyl^[20]^ coupling partners. We viewed the identification of additional catalysts as crucial to finding conditions suitable for a wide array of coupling partners. Second, cross‐electrophile coupling can be challenging if the relative reactivity of the two substrates and intermediary steps are poorly matched.^[21]^ While tuning the reactivity of alkyl halide radical donors by halide choice (iodide, bromide, chloride) or in situ exchange is broadly useful, few analogous tools for NHP esters exist. Baran and co‐workers found that tetrachloro‐NHP esters are significantly more reactive and provided higher yields in cross‐coupling using aryl metal reagents.^[11]^ Because NHP esters are already more reactive than alkyl iodides,[^12a^, ^12c^] methods to decrease the reactivity of NHP esters to the level of alkyl bromides would be helpful in allowing productive cross‐electrophile coupling by better matching the rate of radical generation with oxidative addition. *In theory*, *NHP esters could allow a degree of fine‐tuning not possible with alkyl halides*.

## Results and Discussion

Initial screens began by investigating bidentate pyridine‐type ligands (**L1**–**L4**) as these have been shown to support nickel‐catalyzed cross‐electrophile coupling reactions and have been utilized in other reactions with NHP esters (Table 1).[^12^, ^22^] Informed by this precedent, we found that several of these ligands, as well as previously reported pyridinecarboxamidine ligands (**L5** and **L6**),^[23]^ were effective at promoting the formation of **3** **a** (entries **1**, **4** and **6**, ligands **L1**, **L4**, and **L6**). However, a new ligand recently reported by our lab,^[24]^ 4,4′‐di‐*tert*‐butyl‐6‐*N*‐cyanocarboxamidine‐2,2′‐bipyridine (^*t‐*Bu^BpyCam^CN^, **L7**) promoted the desired reaction with higher yield due to increased selectivity for the cross‐coupled product over alkyl and aryl dimerization reactions. Both a reductant and nickel catalyst are required, and performing the reaction in the absence of a ligand leads to poor selectivity and an overall diminished yield (entries **9**–**11**).

**Table 1:** Optimization of the reaction conditions for coupling with ArI.

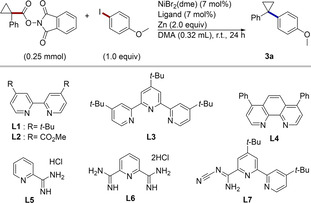				
Entry^[a]^	Variation	**3** **a** Yield [%]^[b]^	Ar–Ar Yield [%]^[b]^	Alk–Alk Yield [%]^[b]^
1^[c]^	**L1**	48	11	20
2^[c]^	**L2**	25	37	0
3^[c]^	**L3**	21	11	25
4^[c]^	**L4**	75	11	0
5^[c]^	**L5**	35	7	30
6^[c]^	**L6**	78	0	0
7^[c]^	**L7** (^*t*‐Bu^BpyCam^CN^)	92	0	0
8^[d]^	**L7**, Mn as reductant	4	0	1
9^[d]^	no ligand	21	2	3
10^[d]^	no nickel, no ligand	0	0	0
11^[d]^	**L7**, no Zn reductant	0	0	0
12^[c]^	**L7**, THF as solvent	72	0	0

[a] A mixture of NHP ester (0.25 mmol), aryl iodide (0.25 mmol), NiBr_2_(dme) (7 mol %), ligand (7 mol %), and Zn (0.5 mmol) was stirred at r.t. (20–22 °C) for 24 h. [b] Corrected GC yield. [c] Remaining mass balance corresponds to formation of cyclopropylbenzene and anisole. [d] Remaining mass balance corresponds to recovered starting material

**Scheme 2:**
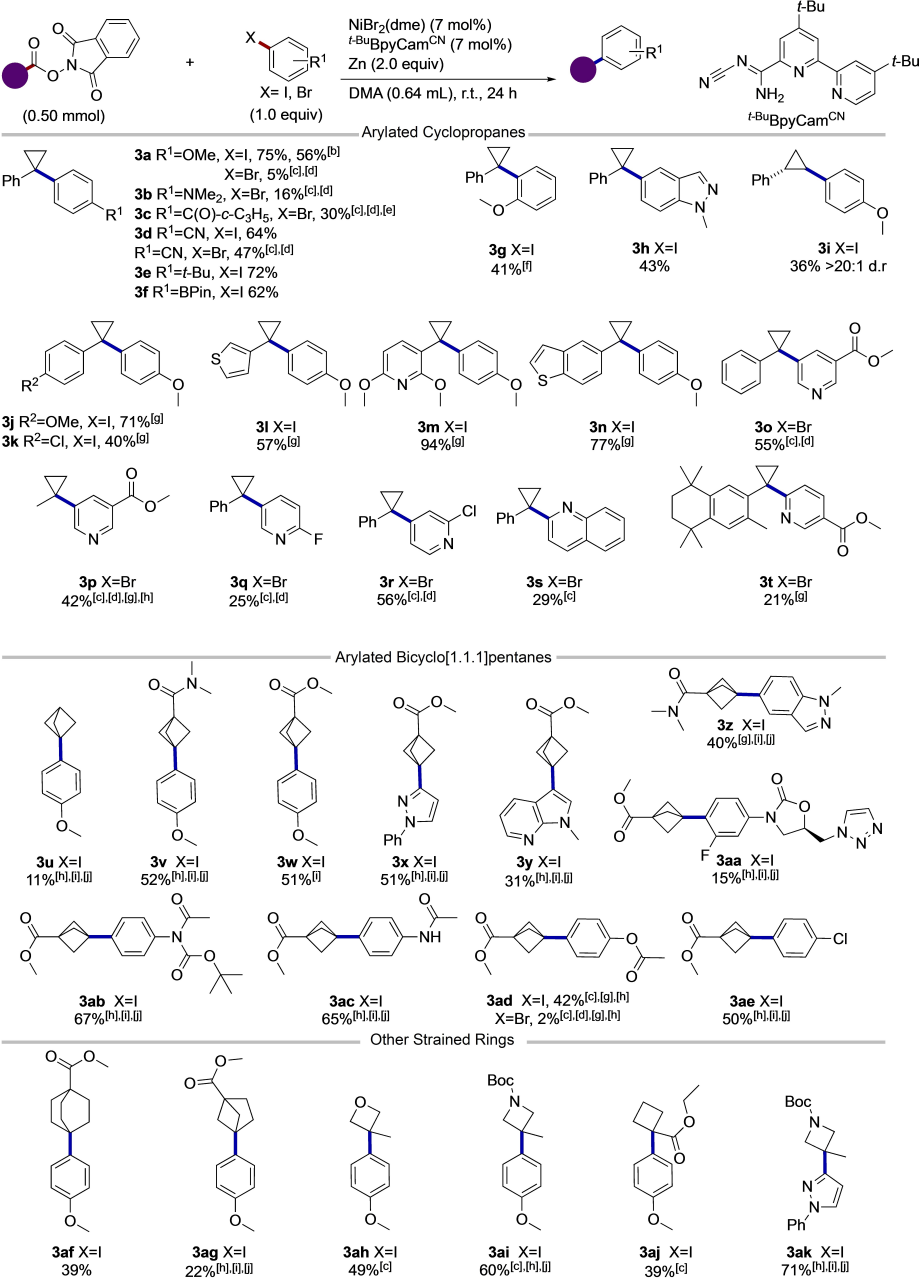
Substrate scope for the decarboxylative coupling of strained‐ring NHP esters with (hetero)aryl halides.^[a]^ [a] Reactions were performed at a 0.5 mmol scale in 0.64 mL of DMA at r.t. (20–22 °C) for 24 h. Yields are isolated yields after purification. [b] NHP ester was generated in situ. [c] Reaction was carried out in THF. [d] Reaction was carried out at 40 °C. [e] NMR yield of product reported. Isolated as an inseparable mixture with corresponding aryl dimer [f] Bathophenanthroline (**L4**) was used as the ligand. [g] Reaction was carried out at 0.25 mmol scale. [h] Reaction was carried out with 20 mol % nickel and ligand. [i] Reaction was carried out in a 9 : 1 mixture of THF:DMA. [j] Reaction was carried out at 0.300 mmol scale.

Applying the optimized conditions to a variety of different carboxylic acid and aryl halide pairs (Scheme 2) demonstrated the utility of this method for the synthesis of diaryl cyclopropanes, a useful replacement for 1,1‐diarylalkenes and diarylmethanes.[^7i^, ^8^, ^25^] Optimized conditions employ a 1 : 1 stoichiometry of NHP ester and (hetero)aryl halide and a typical catalyst loading of 7 mol %, although increasing the catalyst loading to 20 mol % led to improved yields in some cases (**3** **u**, **3** **v**, **3** **x**, **3** **y**, **3** **z**, **3** **aa**, **3** **ag**, **3** **ai**, **3** **ak**). A variety of arene‐based functionalities that enable subsequent elaboration, such as nitriles (**3** **d**), chlorides (**3** **k**, **3** **r**, **3** **t**), esters (**3** **o**, **3** **p**, **3** **t**), and pinacol boronate esters (**3** **f**) were tolerated. Notably, an aryl iodide bearing a substituent in the *ortho* position (**3** **g**) was coupled more efficiently when bidentate **L4** was employed, possibly stemming from increased steric hinderance around the reactive center. Less reactive aryl coupling partners such as aryl bromides (**3** **a**, **3** **b**, **3** **c**, **3** **d**) and heteroaryl bromides (**3** **o**, **3** **p**, **3** **q**, **3** **r**, **3** **s**, **3** **t**) can also engage in the cross‐coupling reaction by changing the reaction solvent to THF and elevating the temperature. Coupling can be achieved at the 2‐, 3‐, and 4‐ position of pyridine and pyridine‐like heterocycles (**3** **o**, **3** **p**, **3** **q**, **3** **r**, **3** **s**, **3** **t**). Aryl halides derived from pyrazole, azaindole, and indazole heterocycles can also be coupled in good yields (**3** **h**, **3** **x**, **3** **y**, **3** **z**, **3** **ak**). For rapid syntheses of analogues, carboxylic acids can be converted to the NHP ester and coupled in one pot to form **3** **a**, albeit with decreased yield (From 75 % with the isolated NHP ester to 56 % with in situ generated NHP ester).

One potential advantage of this approach is that 1,1‐diarylcyclopropanes can be synthesized in a modular fashion from two different aryl halides and cyclopropane carboxylic acid using α‐arylation and decarboxylative cross‐electrophile coupling. Using α‐arylation conditions recently reported by Hartwig,^[26]^ we were able to rapidly synthesize several alternative NHP esters. Changing the arene of benzylic cyclopropyl NHP esters was well tolerated (**3** **j**, **3** **k**, **3** **l**, **3** **m**, **3** **n**). We demonstrate the utility of this approach for the flexible construction of drug‐like molecules through the preparation of the methyl ester of LG100268 **3** **t**, a more potent and specific cyclopropyl analogue of the only FDA‐approved RXR agonist Bexarotene.[^25b^, ^27^] The advantage of this new approach is that it allows for facile modification of the right‐side arene, providing a route for the synthesis of a library of analogues from commercially available aryl halides.

**Scheme 3:**
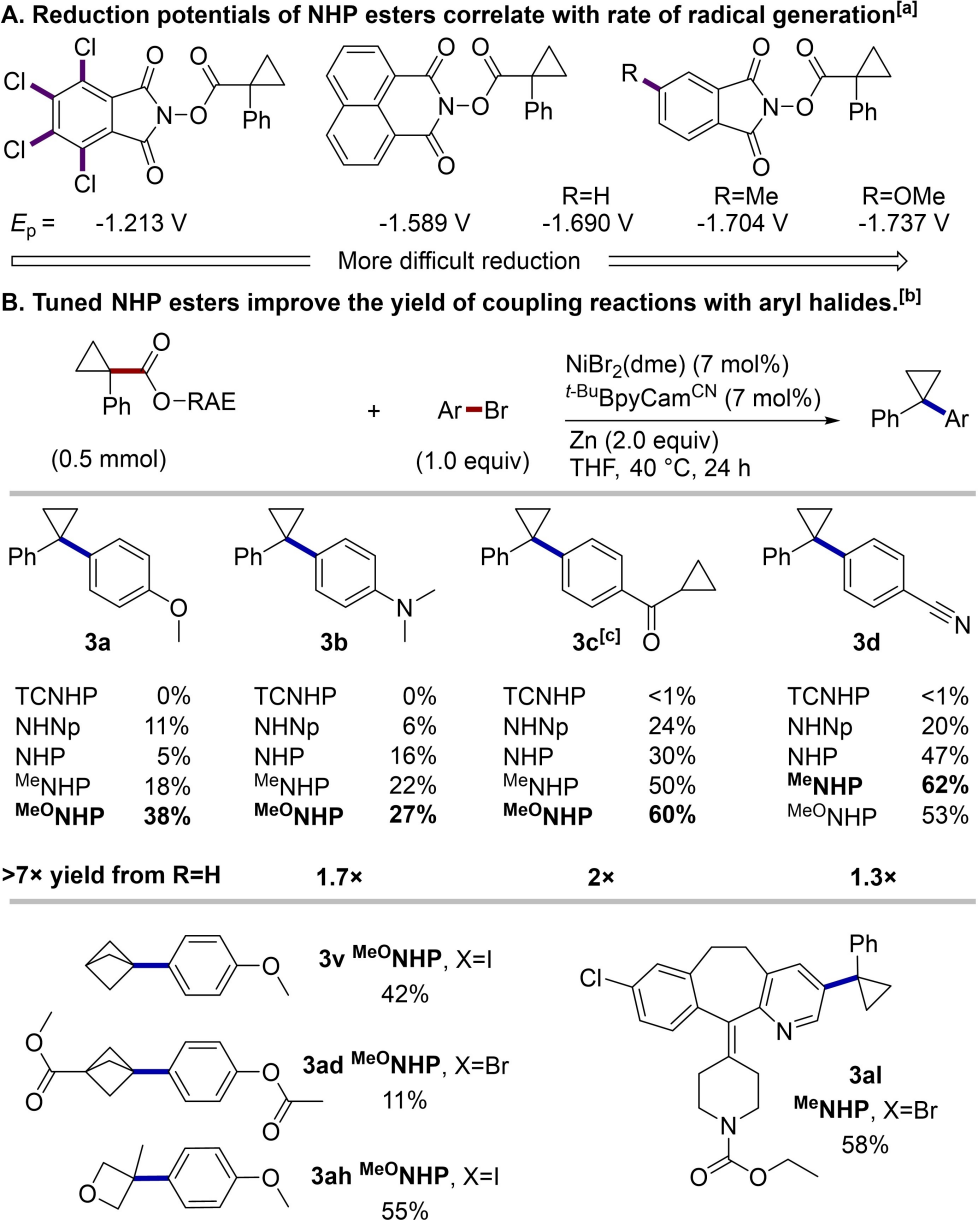
Electronic tuning of NHP esters enables improved yields with ArBr. [a] Cathodic peak potentials vs. Fc^+^/Fc. Radical generation from 0.1 equiv ZnBr_2_ with Zn reductant. See Supporting Information Figures S2–S5. [b] As in Scheme 2. Yields are isolated yields after purification. TCNHP=*N*‐hydroxytetrachlorophthalimide ester, NHNp=*N*‐hydroxynaphthalimide ester. [c] NMR yield of product reported. Isolated as an inseparable mixture with its corresponding aryl dimer.

A variety of other strained‐ring carboxylic acid NHP esters are compatible with these conditions. Non‐benzylic secondary and tertiary strained ring NHP esters (Scheme 2, **3** **i**, **3** **p**) are tolerated under these conditions but are lower yielding, presumably due to the lower stability of the corresponding radicals. Notably, carboxylic acids bearing additional ester functionality can be successfully coupled, providing an easy entry for sequential arylation of bicyclo[1.1.1]pentane, bicyclo[2.2.2]octane, bicyclo[2.1.1]hexane, and cyclobutane ring systems (**3** **w**, **3** **af**, **3** **ag**, **3** **ah**). Other pharmaceutically relevant ring systems such as the NHP esters derived from bicyclo[1.1.1]pentane (**3** **u**–**3** **ae**), bicyclo[2.2.2]octane (**3** **af**), bicyclo[2.1.1]hexane (**3** **ag**), oxetane (**3** **ah**), azetidine (**3** **ai**, **3** **ak**), and, cyclobutane (**3** **aj**) ring systems were also coupled in good yield.^[28]^

This approach appears more general than, and is complementary to, other reported methods. Compared to reactions with arylzinc reagents, these conditions tolerate acidic N−H bonds (e.g., **3** **ac**) and avoid the use of super‐stoichiometric amounts of coupling partners (3–5 equiv).^[11]^ Moreover, in some cases our yields with bicyclo[1.1.1]pentane carboxylic acid NHP esters and aryl iodides were superior to the best yields reported under photochemical conditions with aryl bromides (**3** **ab**, 67 % vs. 24 %; **3** **ad**, 42 % vs. 31 %; **3** **ae**, 50 % vs. 33 %; no aryl iodide couplings were reported in the previous study).^[15]^ However, the coupling to form **3** **ad** from the corresponding aryl bromide was low‐yielding (2 %).

**Figure 1:**
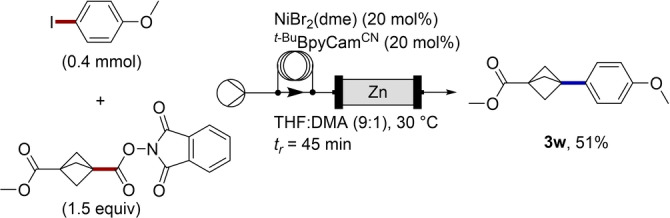
Synthesis of **3** **w** under continuous flow using conditions adapted from Ley and co‐workers.^[29c]^

The chemistry can be scaled in batch (3 mmol, 63 % yield of **3** **a**; Supporting Information Section 3.3.3.) or in flow^[29]^ using the zinc packed‐bed strategy of Ley (*t_r_* =45 min, 51 % yield of **3** **w**; Figure 1).^[29c]^

While exploring the scope with more challenging couplings we observed a mismatch in the reactivity of NHP esters and aryl bromides, often resulting in full conversion of the NHP ester and only partial conversion of the aryl bromide. We envisioned that altering the reduction potential of redox‐active esters would enable us to tune their rate of consumption, thereby providing a new avenue to control the selectivity profile of this coupling. Our group has recently explored the use of modified NHP esters in couplings with alkyl halides, but the reason for their improved reactivity had not been determined.^[30]^ We have found that a combination of solvent effects and NHP ester tuning can improve yields with aryl bromides by slowing the rate of radical generation (Scheme 2). Methyl and methoxy‐substituted NHP (^Me^NHP and ^MeO^NHP) esters are more difficult to reduce (shifts in *E*_p_ of 10–50 mV, Scheme 2A and Figures S2–S4) and are consumed more slowly under reducing conditions (0.1 equiv ZnBr_2_ with Zn reductant, Figure S7).^[31]^ In addition, we found that the time to complete consumption of the NHP ester by ZnBr_2_/Zn^0^ varied with the solvent (10 h in DMA, 17 h in 1 : 1 DMA/THF, >30 h in THF, Figure S8). These effects are complementary, *allowing fine‐tuning of radical generation rates and significant improvements in yields* (up to 7× improvement in yield for **3** **a**) for the coupling of both electron‐rich and electron‐deficient aryl bromides (Scheme 2B). Consistent with the hypothesis that better yields are obtained with esters that are more difficult to reduce, the use of redox active esters that are more easily reduced than NHP (TCNHP ester Figure S5 and *N*‐hydroxynaphthalimide ester Figure S6) led to a significant drop in yield (Scheme 3B). This ester‐tuning strategy was also effective for improving reactions with challenging alkyl fragments that were more likely to participate in deleterious side reactions. Simply employing the ^MeO^NHP ester in place of the NHP ester led to the formation of **3** **v**, **3** **ad**, and **3** **ah** in improved yields (31 %, 9 %, and 6 % improvements respectively). Additionally, an aryl bromide derived from Loratadine could be coupled to form **3** **al** using the ^Me^NHP ester.

**Scheme 4:**
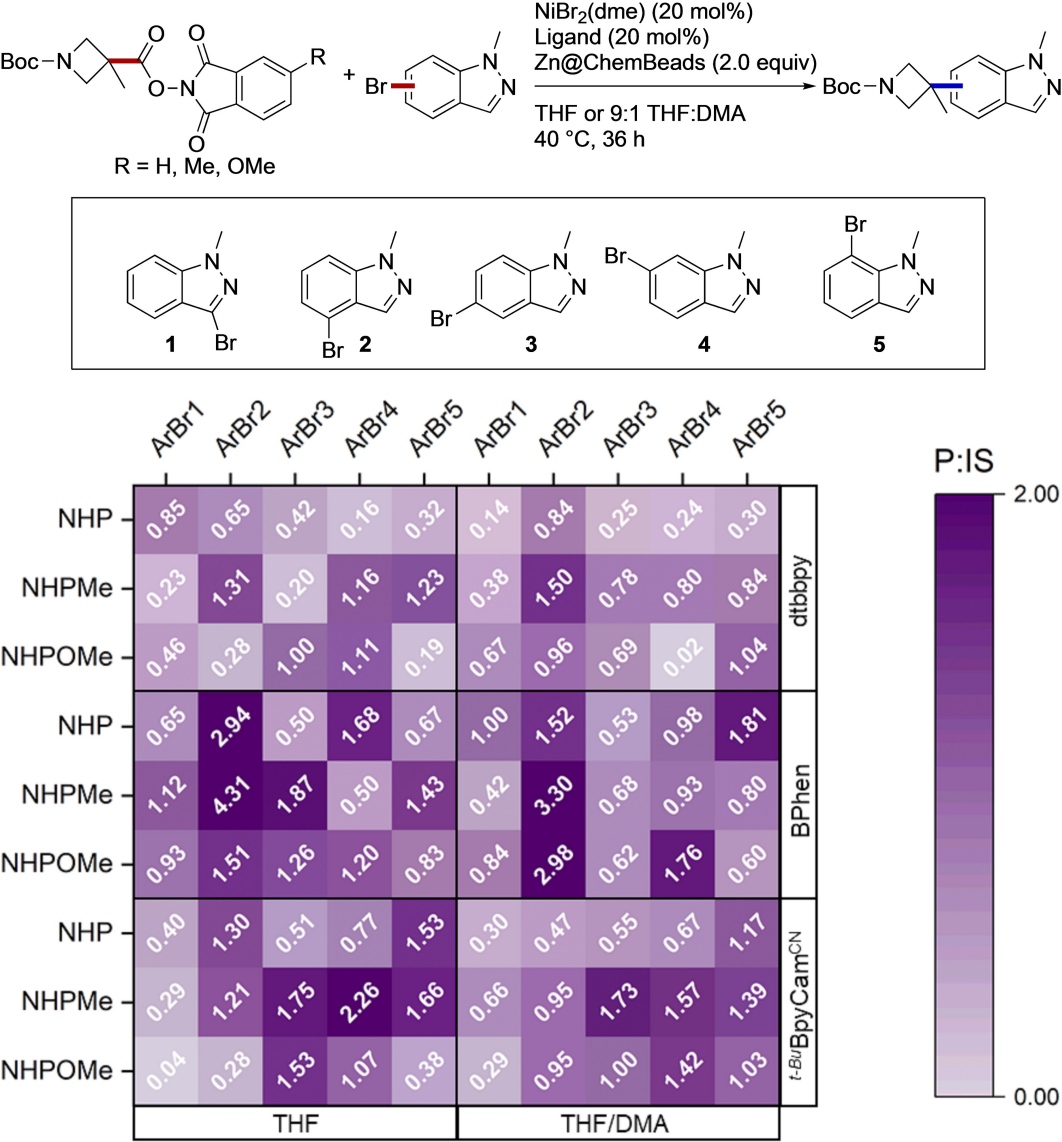
HTE format coupling of NHP esters with bromoindazoles.^[a]^ [a] Reactions run at 10 μmol scale. Assay yields are raw product/internal standard (UV) ratios vs. 1,3,5‐trimethoxybenzene. Note: comparisons of P/IS are only valid among the same aryl halide.

High‐throughput experimentation (HTE) methods are often used in medicinal chemistry to quickly synthesize small collections of molecules to explore structure activity relationships. We were able to adapt strained‐ring cross‐electrophile coupling to a 96‐well plate format at 10 μmol scale using ChemBeads (Scheme 4).[^22^, ^32^] As a representative case, NHP esters of *N*‐Boc‐3‐methylazetidine‐3‐carboxylic acid were coupled to each position of 1‐methylindazole (**ArBr1**–**ArBr5**). To explore how ligand, solvent, and NHP ester can be used to tune reactivity, we examined three different ligands (dtbbpy **L1**, Bphen **L4**, ^*t‐*Bu^BpyCam^CN^ **L7**), three different NHP esters (R=H, Me, OMe), and two different solvent regimes (1 : 1 THF/DMA and THF). These results make two important points. First, a single set of conditions is sufficient for initial screens: methyl NHP esters in THF with **L7** or **L4** provided usable results for all five products. Second, yields can be improved dramatically by adjusting the NHP ester, solvent, and ligand used. For example, while **L4** performed well in this series with methyl NHP esters in THF (best yields on the plate for **ArBr1**, **ArBr2**, **ArBr3**), the yield with **ArBr4** could be more than doubled by switching to **L7**, changing the solvent to THF/DMA, or by using an NHP ester. Finally, **L4** and **L7** performed about equally in this series and exhibited complementary reactivity to each other. **L1**, while common in cross‐electrophile coupling, provided lower yields overall.

For small‐scale screening campaigns, we recommend the following approach. Although ^Me^NHP and ^MeO^NHP esters often outperform NHP esters, NHP esters are sufficient for initial sceens if they are more convenient (due to cost, availability for other reaction types). For couplings with aryl iodides, start with standard NHP esters, ligand **L7** (or **L4** if **L7** is unavailable), 20 mol % catalyst loading, and 9 : 1 THF:DMA mixture (additional DMA if needed for solubility considerations). For aryl bromides, if available, ^Me^NHP esters are an optimal starting point (although NHP esters often still give product). THF (or a mixture of THF and DMA) should be used as the solvent, and these reactions may need to be run at elevated temperatures (40 °C). If the initial conditions do not provide sufficient yields, then an evaluation of the side products can point to optimization strategies: accelerate or slow radical formation using solvent and NHP ester tuning to balance reactivity with the aryl halide. Reactions that consume aryl halide before NHP ester need more polar solvents and more reactive NHP esters. Reactions that consume NHP ester before aryl halide need less polar solvents and less reactive NHP esters. Finally, Scheme 4 shows that a ligand screen could provide improved results. We note that a broader ligand screen that includes low‐performing ligands from Table 1 could be helpful.^[32]^

**Figure 2:**
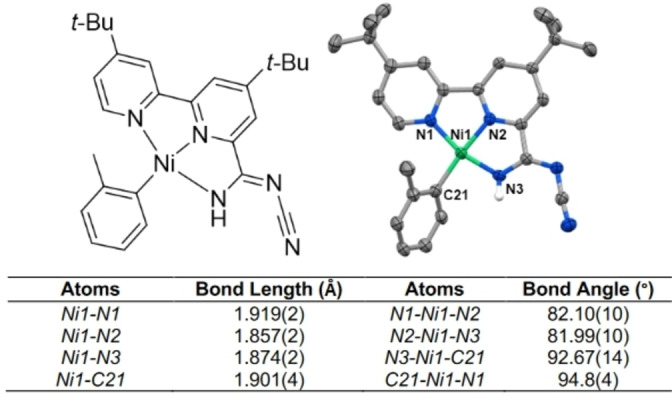
Solid‐state X‐ray structure of (**L7**)Ni(*o*‐tol) at 50 % probability ellipsoids. Relevant bond lengths and bond angles are tabulated below. See Supporting Information Section 4.5 for more information.

Whereas *N*‐cyano carboxamidine ligands like **L7** have proven increasingly useful in cross‐electrophile coupling,^[23, 24^] no structural characterization of their nickel complexes has been reported. We were able to synthesize Ni(**L7**)(*o*‐tol) by reaction of the free ligand with *trans*‐(Ph_3_P)_2_Ni(*o*‐tol)Br. A single‐crystal X‐ray diffraction analysis revealed that Ni(**L7**)(*o*‐tol) crystallizes as a solvate with two symmetry‐independent Ni complexes in the asymmetric unit (Figure 2).^[33]^ The complexes have similar geometries and both display positional disorder of the *o*‐tolyl ligand. Disregarding the minor disorder components, the Ni coordination environment in this neutral metal complex is distorted square planar with the *cis* L−Ni−L angles ranging between 81.99(10)–95.3(7)°. The ^*t‐*Bu^BpyCam^CN^ ligand binds in a tridentate fashion with the *o*‐tolyl occupying the fourth coordination site. The Ni−N distances range between 1.857(2)–1.919(2) Å with the Ni1−N1 distance being ≈0.052(10) Å longer than the other Ni−N distances. These bond lengths fall in the expected range and are not statistically significantly different from 1.90(3) Å, the value obtained by averaging the Ni−N distances in five relevant nickel terpyridyl complexes reported to the Cambridge Structural Database.^[34]^ The Ni−C distances in Ni(**L7**)(*o*‐tol) measure av. 1.900(4) Å and are in excellent agreement with the averaged value of 1.89(3) Å calculated for the Ni−C distances in the same complexes.

The most notable aspects of this structure are the finding that **L7** binds as a monoanionic ligand bound via the unsubstituted nitrogen of the amidinate, reminiscent of the ligand employed in recent work by Sevov.^[35]^ Molander and Gutierrez have studied how LX and L2 ligands can result in different mechanisms and substrate scope in coupling reactions with tertiary radicals.^[19b]^ Further mechanistic studies will be needed to see if related changes in mechanism occur in cross‐electrophile coupling reactions with L2X ligands like ^*t‐*Bu^BpyCam^CN^ (**L7**) and L2 ligands like Bphen (**L4**) and how ligands with such different coordination environments display such a large overlap in substrate scope. These findings are in agreement with recent work by Sevov and co‐workers where both L2 and L2X ligands display similar efficiency in cross‐electrophile coupling reactions.^[35]^

Although we have yet to study the mechanism of this reaction in detail, similarities to other cross‐electrophile couplings with NHP esters and aryl halides suggest an analogous mechanism: (a) initial oxidative addition of the aryl halide to nickel(0) followed by oxidative radical capture by the resulting arylnickel(II) intermediate. Recent studies by the MacMillan group suggest that stoichiometric equivalents of phthalimide can stabilize arylnickel(II), however at this time we are unsure to what extent phthalimide derivatives have the same effect.^[36]^ Reductive elimination from the resulting bisorgano‐nickel(III) species gives the desired product with concomitant formation of a nickel(I) intermediate. The formation of radicals from NHP esters can be mediated by nickel or arise from direct reduction with zinc, assisted by Lewis acid coordination to the NHP ester. Under cross coupling conditions, the NHP ester is fully consumed more quickly than with ZnBr_2_/Zn^0^ alone (<1.5 h vs <10 h), suggesting that the nickel catalyst is also capable of reducing the redox‐active esters.

## Conclusion

In conclusion, we have expanded the scope of decarboxylative C(sp^3^)−C(sp^2^) cross‐electrophile coupling to include seven different classes of pharmaceutically‐relevant strained rings and achieved coupling of NHP esters with (hetero)aryl bromides and iodides. *The generality of this approach with respect to strained‐rings is the widest yet reported for decarboxylative coupling reactions with aryl reagents of any kind*, *including arylmetal reagents*. Two of these ring systems have never been coupled with aryl halides: arylated bicyclo[2.1.1]hexanes have only been synthesized via intramolecular photochemical cyclizations^[37]^ and bicyclo[2.2.2]octane rings have only been coupled to arylzinc reagents.^[11a]^ Additionally, 1,1‐diarylcyclopropanes have not been previously synthesized via a cross‐electrophile coupling approach. This chemistry is enabled by a new ligand (^*t‐*Bu^BpyCam^CN^) that promotes cross‐selective coupling, and the tuning of NHP ester reactivity by altering the substituents on the phthalimide backbone and the reaction solvent. We envision that further ligand design and NHP ester tuning will enable the use of even less reactive coupling partners in the future, expanding the utility of redox active esters as a tool for C−C bond formation. We note that, while this manuscript was in review, the Baran group in collaboration with several pharmaceutical companies reported a complementary approach to tuning NHP ester reactivity under electrochemical conditions by doping the nickel cathode with silver;^[38]^ we imagine that combining new ligands, tuned NHP esters, and electrochemistry could be particularly fruitful.

## Conflict of interest

The authors declare no conflict of interest.

1

## Supporting information

As a service to our authors and readers, this journal provides supporting information supplied by the authors. Such materials are peer reviewed and may be re‐organized for online delivery, but are not copy‐edited or typeset. Technical support issues arising from supporting information (other than missing files) should be addressed to the authors.

Supporting Information

## Data Availability

The data that support the findings of this study are available in the Supporting Information of this article.
