# Supplementary material for: Control of Redox‐Active Ester Reactivity Enables a General Cross‐Electrophile Approach to Access Arylated Strained Rings
Source: Angew Chem Int Ed Engl. 2022 Jul 5;61(33):e202205673. doi: 10.1002/anie.202205673 (PMC9378488; doi:10.1002/anie.202205673)

Angewandte Chemie
Eine Zeitschrift der Gesellschaft Deutscher Chemiker

Supporting Information

Control of Redox-Active Ester Reactivity Enables a General Cross-Electrophile Approach to Access Arylated Strained Rings

D. C. Salgueiro, B. K. Chi, I. A. Guzei, P. García-Reynaga, D. J. Weix**

Supporting Information
Table of Contents

Supporting Information **1**
Table of Contents **1**
1. General Information **2**
1.1 Reagents **2**
Metals 2
Ligands 2
Substrates 2
Solvents 2
Other Reagents 2
1.2 Methods **3**
NMR Spectroscopy 3
Gas Chromatography (GC) 3
GC/MS Analysis 3
Supercritical Fluid Chromatography Mass Spectrometry (SFC/MS) 4
Liquid Chromatography Mass Spectrometry (LC/MS) 4
Infrared Spectroscopy 4
Chromatography 4
Elemental Analysis 4
High Resolution Mass Spectrometry 4
2. Supplemental Data **5**
2.1 Figure S1. Commercial Availability of Strained Ring-Containing Building Blocks[a] **5**
2.2 Cyclic Voltammograms of Substituted NHP Esters **5**
2.2.1 Figure S2. Cyclic Voltammogram of 1,3-dioxoisoindolin-2-yl 1-phenylcyclopropane-1-carboxylate 6
2.2.2 Figure S3. Cyclic Voltammogram of 5-methyl-1,3-dioxoisoindolin-2-yl 1-phenylcyclopropane-1-carboxylate 6
2.2.3 Figure S4. Cyclic Voltammogram of 5-methoxy-1,3-dioxoisoindolin-2-yl 1-phenylcyclopropane-1-carboxylate 7
2.2.4 Figure S5. Cyclic Voltammogram of 1,3-dioxo-1H-benzo[de]isoquinolin-2(3H)-yl 1-phenylcyclopropane-1-carboxylate 7
2.2.5 Figure S6. Cyclic Voltammogram of 4,5,6,7-tetrachloro-1,3-dioxoisoindolin-2-yl 1-phenylcyclopropane-1-carboxylate 8
2.3 Figure S7. The effect of NHP ester substitution on the rate of decarboxylation **8**
2.4 Figure S8. The effect of solvent on the rate of decarboxylation **9**
2.4 Figure S9. Time course for the model reaction **10**
3. General Reaction Procedures **11**
3.1 Synthesis of Tert-butyl Esters **11**
3.2 Synthesis of NHP Esters **12**
3.2.1 General Procedure A: Synthesis of NHP Esters Using DIC. 12
3.2.2 General Procedure B: Synthesis of NHP Esters Using PITU. 12
3.3 General Procedures for Decarboxylative Cross-Electrophile Coupling **13**
3.3.1 General Procedure C. 13

3.3.2 General Procedure D. Ni-catalyzed Decarboxylative Cross-Electrophile Coupling without the use of a Glovebox. 13
3.3.3 Preparative-Scale Benchtop Procedure 15
3.3.4 Decarboxylative Cross-Electrophile Coupling Under Continuous Flow Exemplified for the synthesis of 3-(4-methoxyphenyl)bicyclo[1.1.1]pentane-1-carboxylate (3w). 16
3.3.5 Decarboxylative Cross-Electrophile Coupling in a High-Throughput Experimentation (HTE) Mode 17
4. Specific Procedures and Product Characterization 23
4.1 Synthesis of 4,4'-di-*tert*-butyl-*N*-cyano-2,2'-bipyridine-6-carboximidamide (L7) 23
4.2 NHP Esters 29
4.3 Products 38
4.4 Preparation of ($^{t\text{-Bu}}$BpyCam^{CN})Ni(*o*-tol) 58
4.5 Crystallographic Data 58
5. References 77
6. NMR Spectra 80

1. General Information

1.1 Reagents

Metals

All metal catalysts and metal reductants, unless otherwise noted, were stored and handled in a nitrogen-filled glovebox. Nickel(II) bromide ethylene glycol dimethyl ether complex ($NiBr_2$(dme)) was purchased from Millipore Sigma and used as received. The reductant used was zinc flake, -325 mesh, 97% (Alfa Aesar). We observed no difference in reactivity between zinc flake and zinc dust.

Ligands

Pyridyl carboxamidine ligands were synthesized according to literature procedures. All other ligands were purchased from commercial suppliers and used without purification.

Substrates

Cyclopropyl carboxylic acids were synthesized from the corresponding *t*-Bu esters (**SI-1** – **SI-3**) prepared according to a literature procedure.[1] All other carboxylic acids were purchased from commercial suppliers.

Solvents

Tetrahydrofuran (THF) and dichloromethane (DCM) were purified by passage though activated alumina and molecular sieves in a solvent purification system (Inert Corporation) and stored in a nitrogen-filled glovebox. Anhydrous dimethylacetamide (DMA) was purchased from Millipore Sigma, stored in a glovebox, and used as received.

Other Reagents

All starting materials were purchased from commercial suppliers and used without purification unless otherwise indicated.

1.2 Methods

NMR Spectroscopy

UW-Madison: ^{1}H and ^{13}C-NMR spectra were acquired on a 500 MHz Avance spectrometer equipped with a DCH cryoprobe (Bruker), at a sample temperature of 25 °C. NMR spectra were recorded with TopSpin 3.5.6 (Bruker).

Janssen: 500 MHz: ^{1}H NMR spectra were recorded on a Bruker Avance Neo NMR spectrometer operating at 500.13 MHz for ^{1}H with the following spectral parameters: acquisition time = 4 s, number of scans = 16, number of data points = 32 K and spectral width = 8197 Hz. ^{13}C NMR spectra were taken on the same instrument operating at 125.758 MHz for ^{13}C with the following spectral parameters: acquisition time = 1.1 s, number of scans = 2048, number of data points = 32K and spectral width = 30120 Hz. ^{19}F spectra were collected operating at 470.592 MHz for ^{19}F using acquisition time = 0.58 s, number of scans = 16, number of data points = 65K and spectral width = 113636 Hz. Measurements were made using 5 mm tubes in a BBFO probe.

600 MHz: ^{1}H NMR spectra were recorded on a Bruker Avance Neo NMR spectrometer operating at 500.13 MHz for ^{1}H with the following spectral parameters: acquisition time = 2.6 s, number of scans = 1, number of data points = 32 K and spectral width = 12500 Hz. ^{13}C NMR spectra were taken on the same instrument operating at 150.903 MHz for ^{13}C with the following spectral parameters: acquisition time = 0.92 s, number of scans = 512, number of data points = 32K and spectral width = 35713 Hz. ^{19}F spectra were collected operating at 564.686 MHz for ^{19}F using acquisition time = 0.5 s, number of scans = 4, number of data points = 65K and spectral width = 131579 Hz. Measurements were made using 5 mm tubes in a Prodigy Nitrogen cooled BBO cryoprobe.

Referencing and absolute referencing to TMS, apodization, Fourier transform, phase and baseline corrections, and spectral analyses were carried out with MestReNova 12.0.4 (Mestrelab Research). NMR chemical shifts are reported in ppm and are referenced to TMS (δ = 0.00 ppm). Coupling constants (J) are reported in Hz.

Gas Chromatography (GC)

GC analyses were performed on an Agilent 7890A GC equipped with dual DB-5 columns (20 m × 180 µm × 0.18 µm), dual FID detectors, and hydrogen as the carrier gas. A sample volume of 1 µL was injected at a temperature of 300 °C and a 100:1 split ratio. The initial inlet pressure was 20.3 psi but varied as the column flow was held constant at 1.8 mL/min for the duration of the run. The initial oven temperature of 50 °C was held for 0.46 min followed by a temperature ramp of 65 °C/min up to 300 °C. The total run time was 5.0 min and the FID temperature was 325 °C.

GC/MS Analysis

GC/MS analyses were performed on a Shimadzu GCMS-QP2010 equipped with an RTX-5MS column (30 m × 0.25 mm × 0.25 µm) with a quadrupole mass analyzer using helium as the carrier gas or with an Agilent 5977A GC/MSD using MassWorkds 4.0 from CERNO bioscience. The analysis method used in all cases was 1 µL injection of sample, an injection temp of 250 °C, and a 20:1 split ratio. The initial inlet pressure was 8.1 psi, but varied as the column flow was held constant at 1.0 mL/min for the duration of the run. The interface temperature was held at 275 °C, and the ion source (EI^{+}, 30 eV) was held at 200 °C. The initial oven temperature was held at 60 °C for 1 min with the detector off, followed by a temperature ramp, with the detector on, to 300 °C at 20 °C/min. Total run time was 13.00 min.

Supercritical Fluid Chromatography Mass Spectrometry (SFC/MS)
SFC/MS analyses were performed on a Waters ACQUITY UPC^2 equipped with ACQUITY UPC^2 PDA and ACQUITY QDa Detector. A Daicel Dcpack SFC-A column (3 mm ID × 150 mm L, 3 µm PS) was used for separations. The eluent was a mixture (97:3 CO_2/MeOH) with a flow rate of 2 mL/min at 40 °C with a ABPR at 1500 psi. We are grateful to Joe Barendt and Chiral Technologies for the donation of the SFC-A column used in this work.

Liquid Chromatography Mass Spectrometry (LC/MS)
UW-Madison: UPLC-MS analyses were performed on a Waters Acquity UHPLC using a BEH-C18 column (1.7 µm, 2.1 x 50 mm) with an Acquity QDA MS detector. MPA: 0.05% TFA in H_2O; MPB: 100% ACN, starts from 1% B to 40% B for 0.5 minutes, then hold for 1.25 minutes, then increase to 65% B for 0.5 min, then hold for 1.1 minutes, then increase to 95% B over 0.15 minutes.
Janssen: Analytical LCMS was obtained on an Agilent 1200 Series using an ACE-C18 column (3µm, 3.0 x 50 mm, T=50°C). MPA: 0.05% TFA in H_2O; MPB: 100% ACN, Gradient method starts from 5% B to 100% B in 2.3 mins at a flow rate of 2.0 mL/min. MS detector is an Agilent G6125B API-ESI set in positive mode.

Infrared Spectroscopy
ATR-FTIR spectroscopy data was obtained using a Jasco FT/IR-4700 instrument.

Chromatography
Chromatography was performed on silica gel (EMD, silica gel 60, particle size 0.040-0.063 mm) using standard flash techniques, on a Teledyne Isco CombiFlash instrument using pre-packaged cartidges, on a Teledyne Isco Rf-200 (detection at 210 nm and 280 nm), or on a Biotage Isolera One (detection at 210 nm and 400 nm, on Sfar Duo columns). Products were visualized by UV, PMA stain, or fractions were analyzed by GC. Purifications using an HPLC were performed using a Teledyne ACCQ Prep HPLC system using an XBridge C18 column (5 µm, 100×50 mm), mobile phase of 5-100% ACN in 20 mM NH4OH over 17 min and then hold at 100% ACN for 3 min, at a flow rate of 80 mL/min.

Elemental Analysis
Elemental analyses were performed by CENTC Elemental Analysis Facility at University of Rochester, funded by NSF CHE-0650456.

High Resolution Mass Spectrometry
UW-Madison: High resolution mass spectra (HRMS) Mass spectrometry data was collected on a Thermo Q Exactive™ Plus (thermofisher.com) via flow injection with electrosprayionization or via ASAPMS™ (asap -ms.com) by the chemistry mass spectrometry facility at the University of Wisconsin – Madison. The purchase of the Thermo Q Exactive Plus in 2015 was funded by NIH Award 1S10 OD020022 to the Department of Chemistry
Janssen: High-resolution mass spectra (HRMS) were measured on an Agilent Technologies 6200 series mass spectrometer using electrospray ionization (ESI) time-of-flight (TOF) or on an Agilent 5975C GC/MSD (EI) using MassWorks 4.0 from CERNO bioscience.

Flow Chemistry Equipment
Flow chemistry was performed on a Vapourtec R2+R4 instrument.

2. Supplemental Data

2.1 Figure S1. Commercial Availability of Strained Ring-Containing Building Blocks[a]

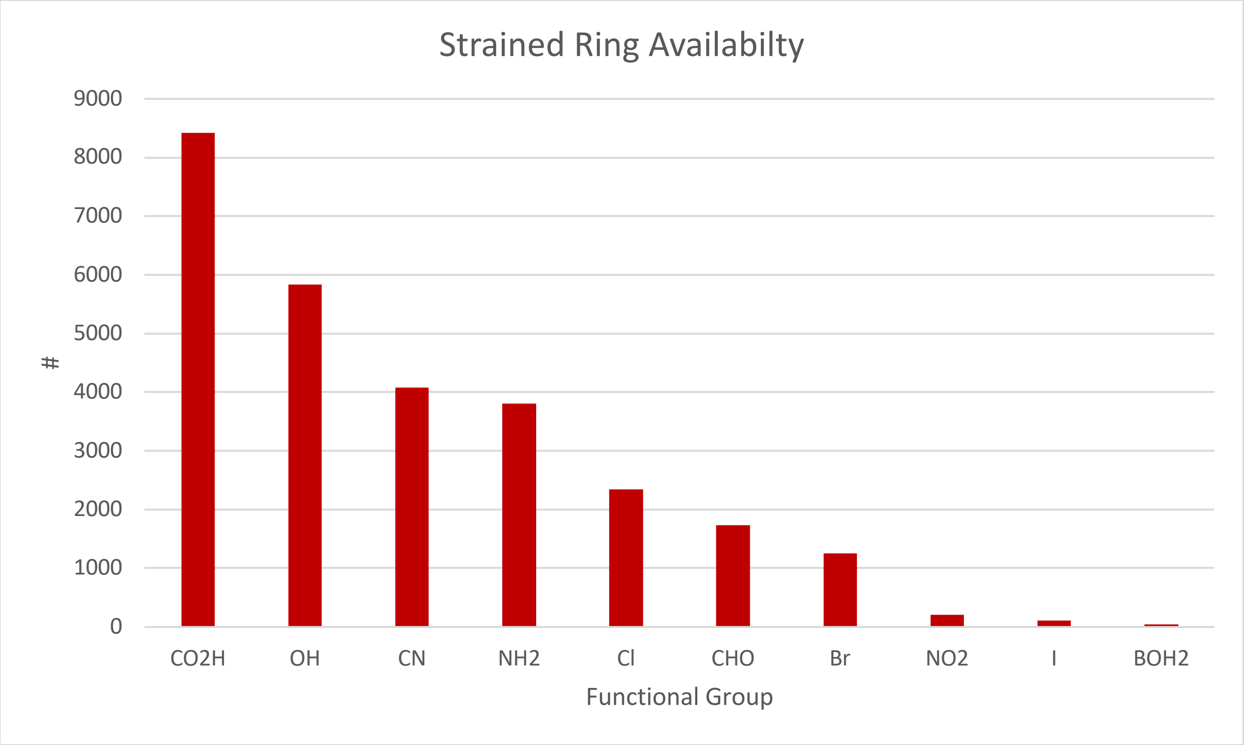

[a]3 and 4 membered carbocycle and heterocycle substrate commercial availability (Reaxys) as of November, 2021.

2.2 Cyclic Voltammograms of Substituted NHP Esters

[a]Cyclic voltammetry was performed using a NuVant EZStatPro Potentiostat at a sweep rate of 100 mV/s. Solutions were made to contain 5 mM of the analyte and 100 mM Bu_4NPF_6 in DMF. The sample was prepared in a vial equipped with a glassy carbon disk working electrode (3 mm diameter, purchased from BASi), Pt wire counter electrode (purchased from BASi) and a Ag/AgCl reference electrode in 3 M NaCl (purchased from BASi). Before data collection, each solution was sparged vigorously with nitrogen for 10 minutes. The reversible peak following reduction of the NHP esters is consistent with the reduction of benzyl radicals to benzyl anions.[2]

2.2.1 Figure S2. Cyclic Voltammogram of 1,3-dioxoisoindolin-2-yl 1-phenylcyclopropane-1-carboxylate

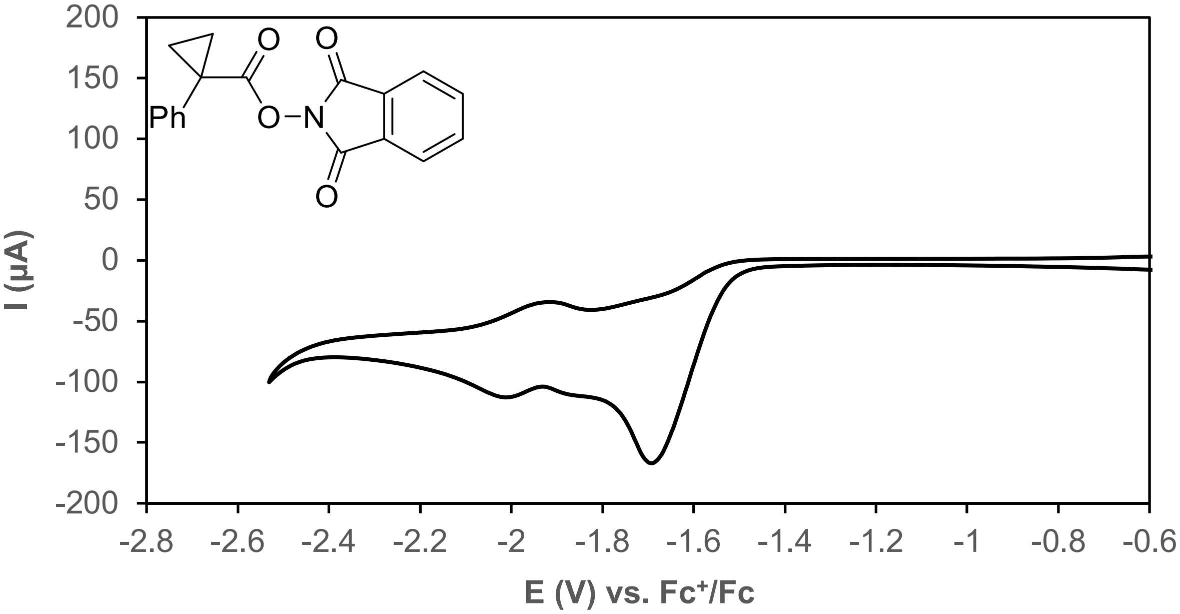

E_p=-1.690 vs Fc^+/Fc

2.2.2 Figure S3. Cyclic Voltammogram of 5-methyl-1,3-dioxoisoindolin-2-yl 1-phenylcyclopropane-1-carboxylate

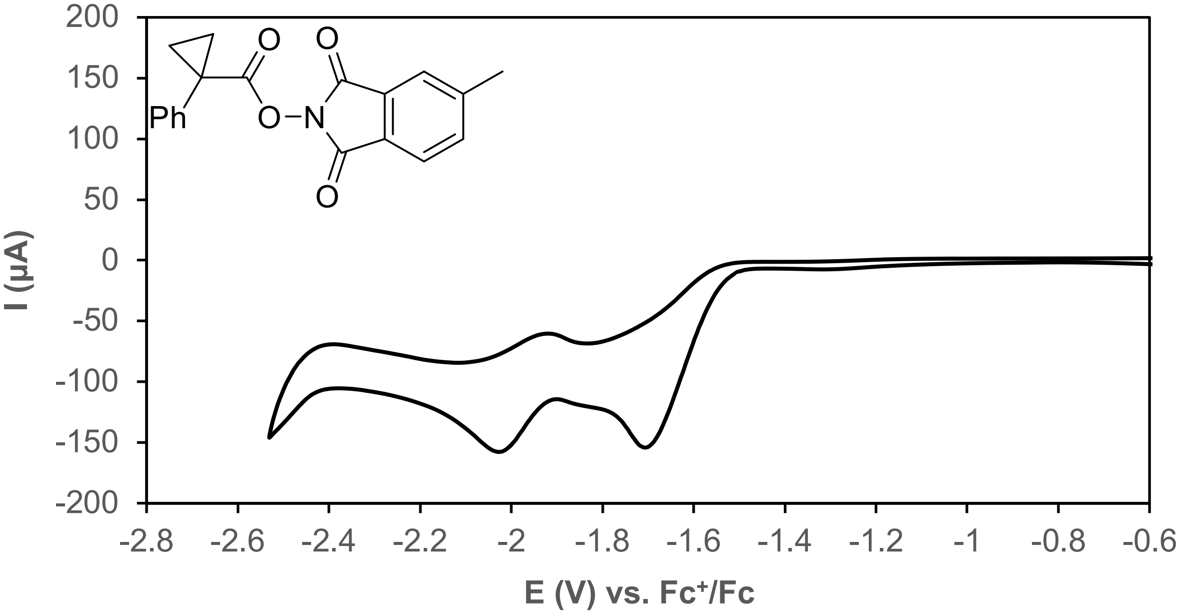

E_p=-1.704 vs Fc^+/Fc

2.2.3 Figure S4. Cyclic Voltammogram of 5-methoxy-1,3-dioxoisoindolin-2-yl 1-phenylcyclopropane-1-carboxylate

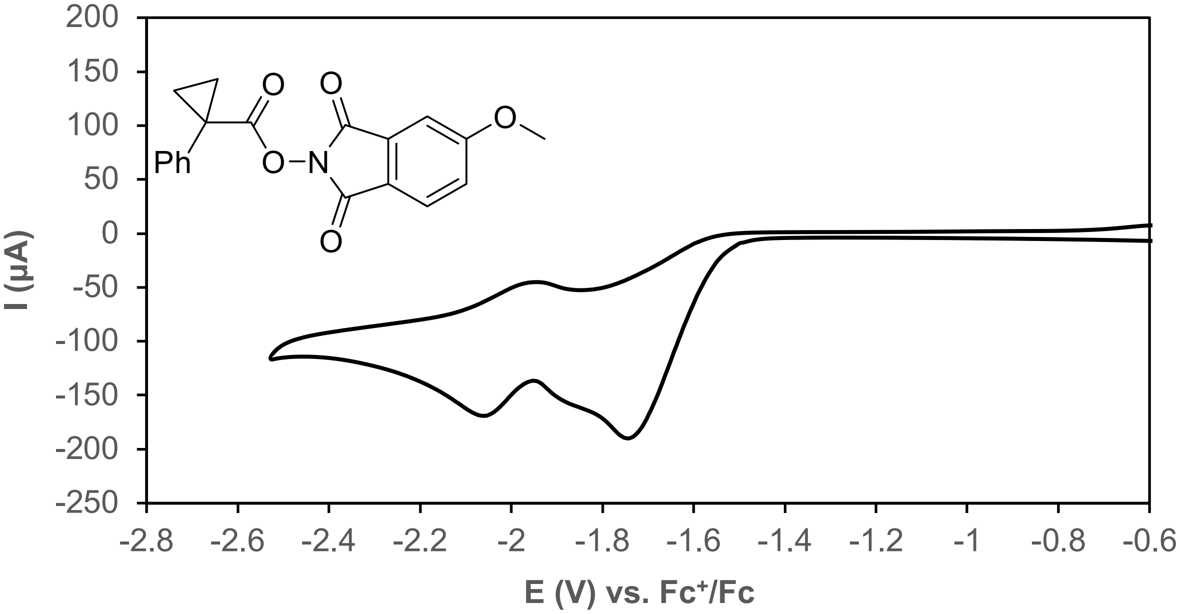

E_p= -1.737 vs Fc^+/Fc

2.2.4 Figure S5. Cyclic Voltammogram of 1,3-dioxo-1H-benzo[de]isoquinolin-2(3H)-yl 1-phenylcyclopropane-1-carboxylate

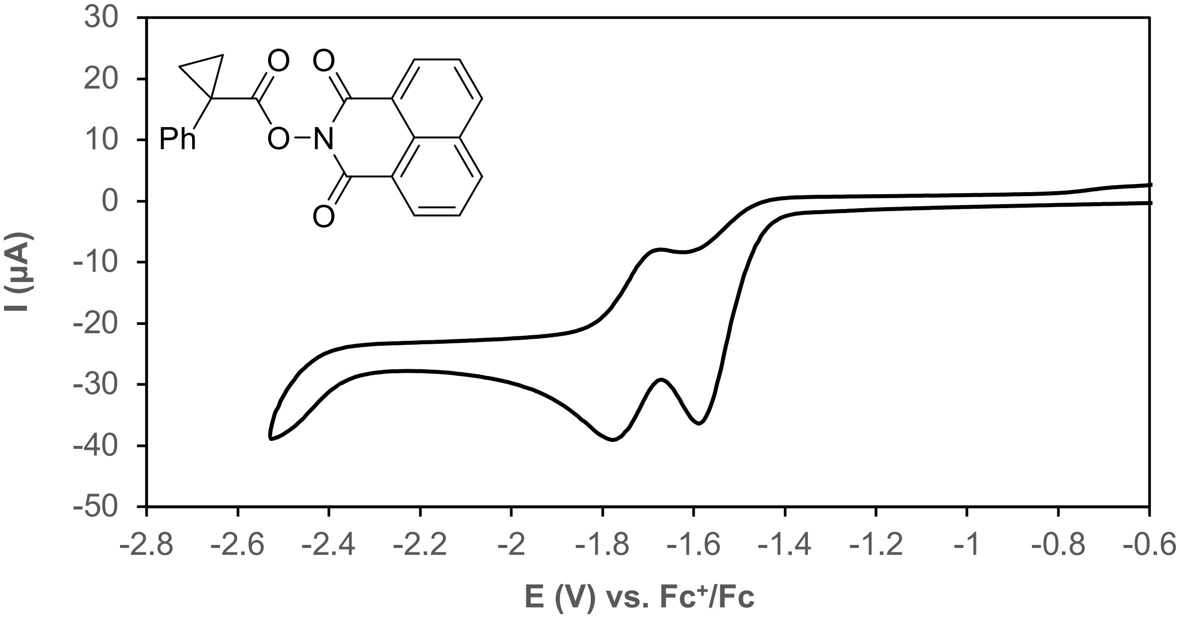

E_p= -1.589 vs Fc^+/Fc

2.2.5 Figure S6. Cyclic Voltammogram of 4,5,6,7-tetrachloro-1,3-dioxoisoindolin-2-yl 1-phenylcyclopropane-1-carboxylate

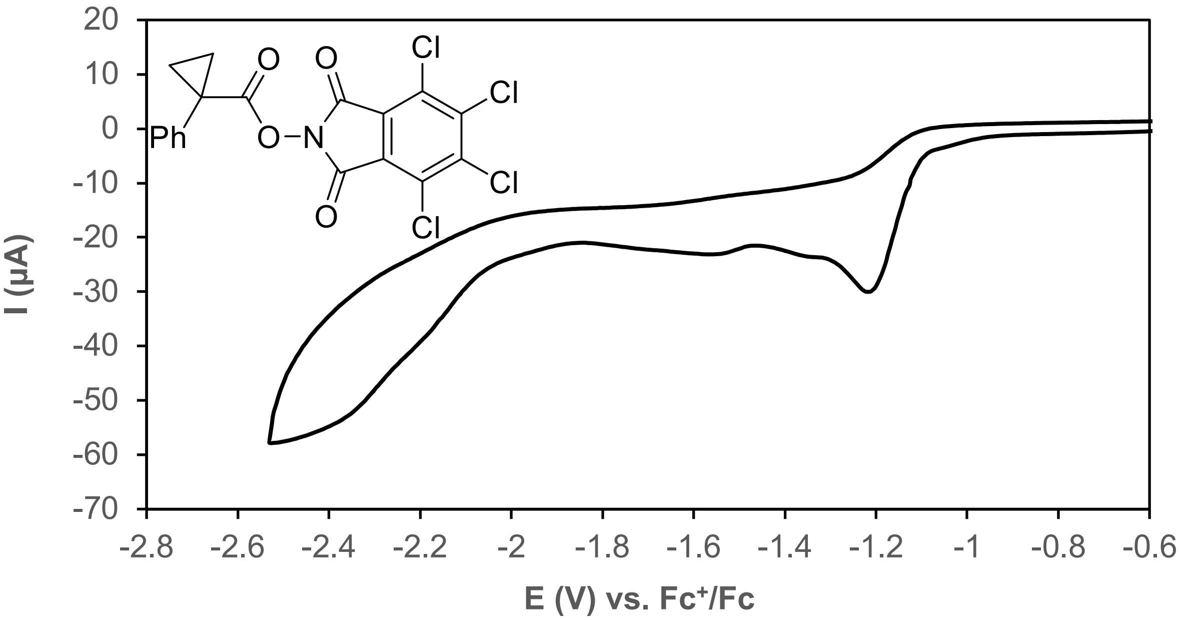

E_p= -1.213 vs Fc^+/Fc

2.3 Figure S7. The effect of NHP ester substitution on the rate of decarboxylation

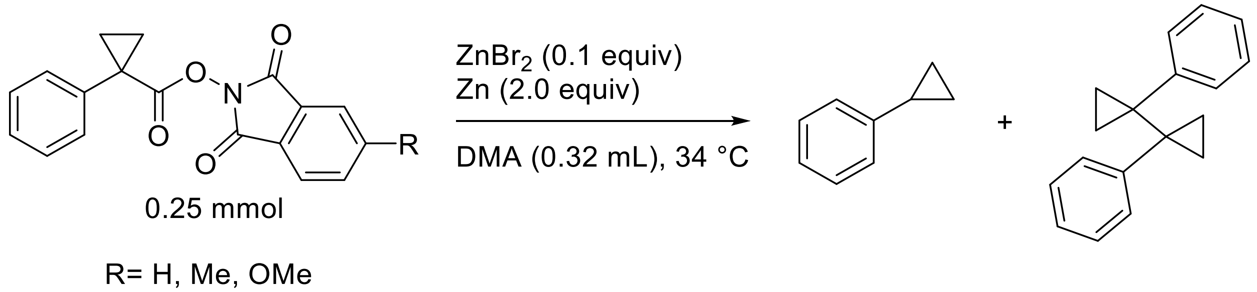

Reactions were setup in an N_2-filled glovebox for convenience. An oven-dried 1-dram vial with a PTFE-coated stirbar was charged with NHP ester (0.25 mmol, 1.0 equiv), zinc bromide (5.6 mg, 0.025 mmol, 0.10 equiv) and zinc (32.7 mg, 0.5 mmol, 2.0 equiv), and 1,3,5-trimethoxybenzene (21.1 mg, 0.125 mmol) internal standard, followed by the addition of DMA (0.32 mL). The reactions were sealed with a screw cap fitted with a PTFE-faced silicone septum and placed on a stir plate in the glovebox and were left to stir (1200 RPM) at 34 °C for 24 h. Aliquots of the reaction were taken, diluted with EtOAc, filtered through silica, and analyzed by SFC-MS.

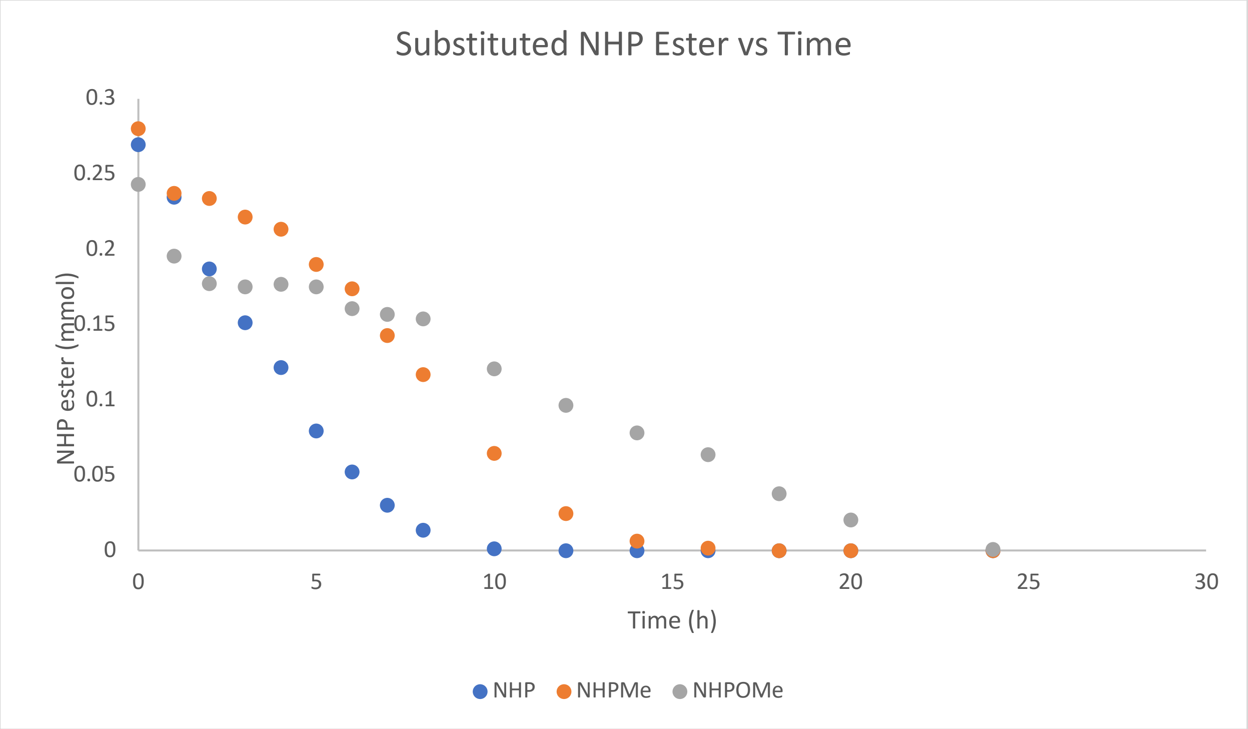

2.4 Figure S8. The effect of solvent on the rate of decarboxylation

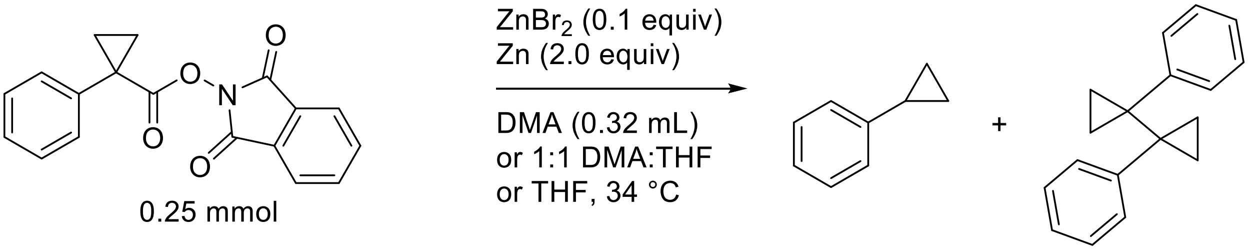

Reactions were setup in an N_2-filled glovebox for convenience. An oven-dried 1-dram vial with a PTFE-coated stirbar was charged with NHP ester (76.8 mg, 0.25 mmol, 1.0 equiv), zinc bromide (5.6 mg, 0.025 mmol, 0.10 equiv) and zinc (32.7 mg, 0.5 mmol, 2.0 equiv), and 1,3,5-trimethoxybenzene (21.1 mg, 0.125 mmol) internal standard, followed by the addition of solvent (0.32 mL). The reactions were sealed with a screw cap fitted with a PTFE-faced silicone septum and placed on a stir plate in the glovebox and were left to stir (1200 RPM) at 34 °C for 24 h. Aliquots of the reaction were taken, diluted with EtOAc, filtered through silica, and analyzed by SFC-MS.

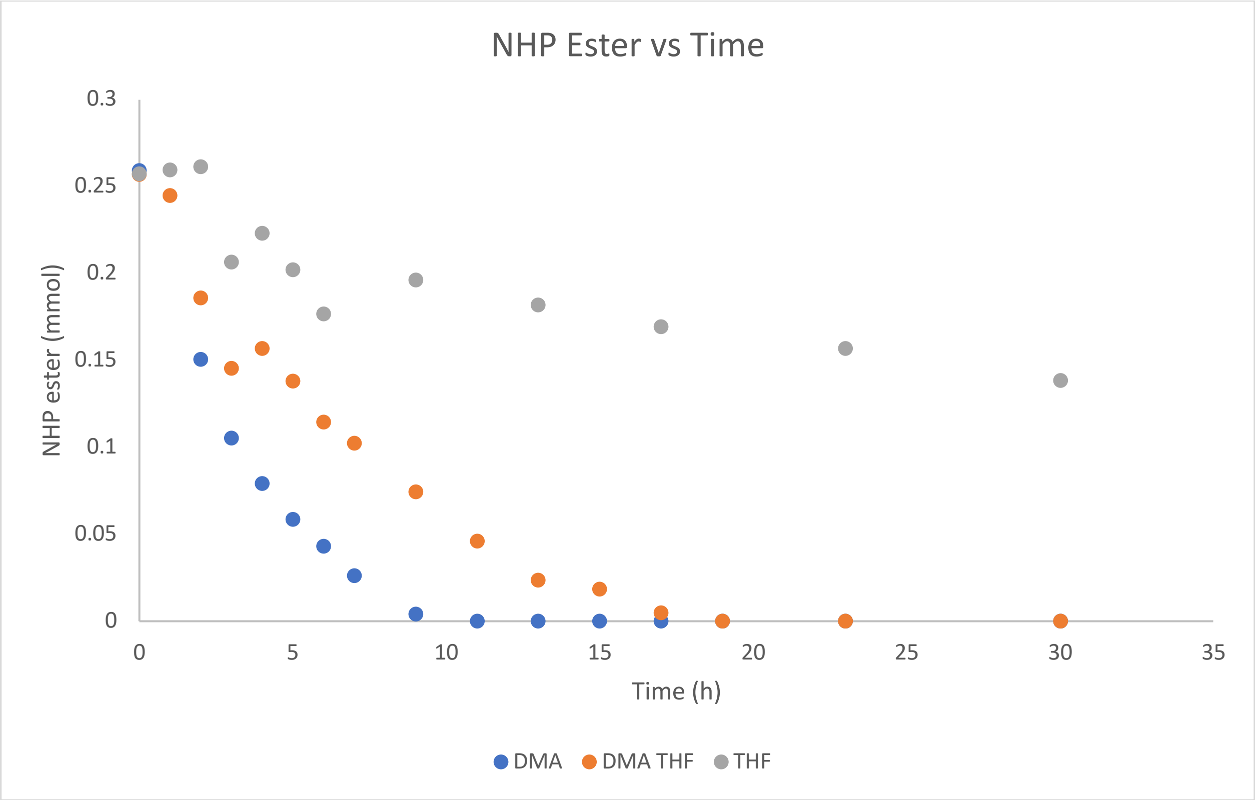

2.4 Figure S9. Time course for the model reaction

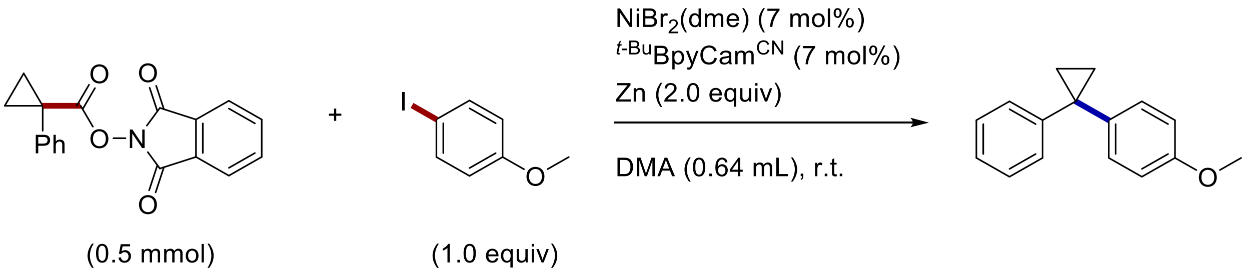

Reactions were setup in an N_2-filled glovebox for convenience. An oven-dried 1-dram vial with a PTFE-coated stirbar was charged with NHP ester (154 mg, 0.50 mmol, 1.0 equiv), aryl iodide (116 mg, 0.50 mmol, 1.0 equiv) and zinc (64.6 mg, 1.0 mmol, 2.0 equiv), and 1,3,5-trimethoxybenzene (7.4 mg, 0.044 mmol) internal standard, followed by the addition of solvent (0.32 mL). The reactions were sealed with a screw cap fitted with a PTFE-faced silicone septum , removed from the glovebox placed on a stir plate, and were left to stir (1200 RPM) at rt (20-22 °C) for 24 h. Aliquots of the reaction were taken, diluted with Et_2O, filtered through silica, and analyzed by GC.

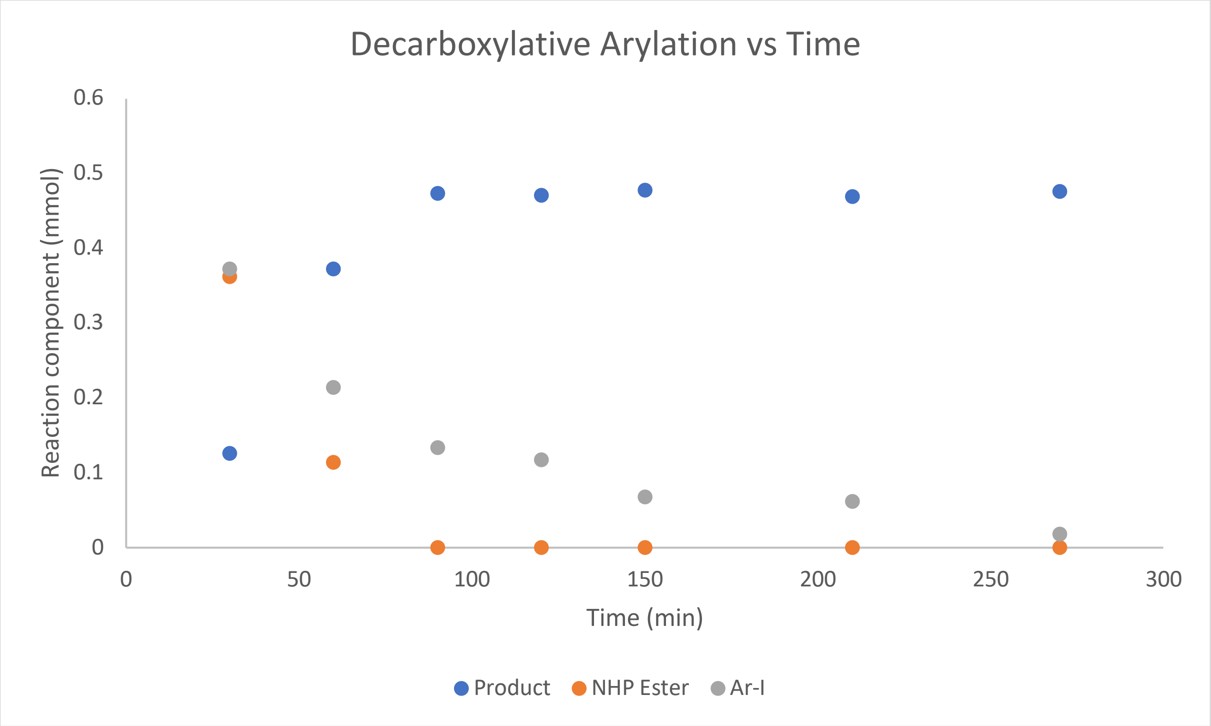

3. General Reaction Procedures

3.1 Synthesis of Tert-butyl Esters

Tert-butyl esters were prepared according to a previously reported procedure.[1]

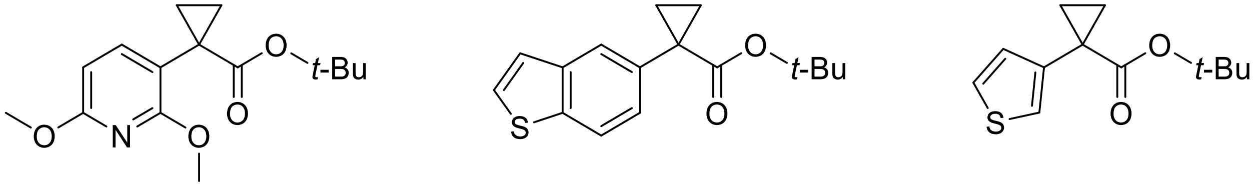

SI-1 **SI-2** **SI-3**

To a 20 mL scintillation vial containing solid $LiNCy_2$ (2.1 equiv) was added dropwise a solution of tert-butyl cyclopropanecarboxylate (511.9 mg, 3.60 mmol, 2.0 equiv) in toluene (0.67 M) while stirring for 15 minutes. Meanwhile, to a separate 20 mL scintillation vial was charged $AgBF_4$ (0.05 equiv), Pd(1-tBu-Indenyl)($PtBu_3$)(Cl) (0.05 equiv), aryl bromide (1 equiv), and toluene (1 M). The resulting mixture was shaken by hand for 30 seconds before transferring the enolate solution into the vial. The vial was sealed with a screw cap fitted with a PTFE-faced silicone septum and removed from the glovebox. The reaction mixture was left stirring at 65 °C for 12 hours. After the specified time, the reaction mixture was concentrated *in vacuo* and directly loaded onto silica gel for column chromatography to afford the product.

3.2 Synthesis of NHP Esters

NHP esters were prepared according to previously reported procedures using DIC[3–11] **(General Procedure A)** or PITU[9] **(General Procedure B)** as the coupling agent.

3.2.1 General Procedure A: Synthesis of NHP Esters Using DIC.

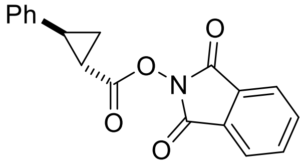

NHP ester 1
Ref. 5

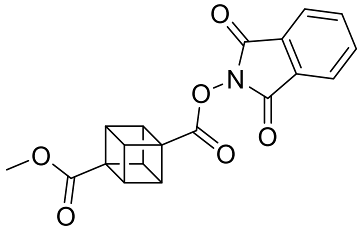

NHP ester 2
Ref. 10

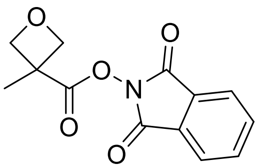

NHP ester 3
Ref. 9

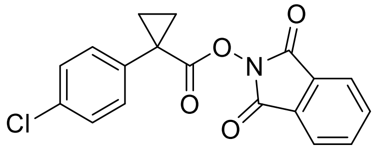

NHP ester 4
Ref. 4

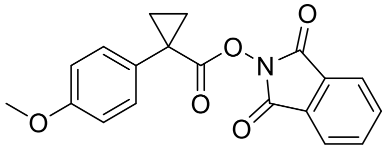

NHP ester 5
Ref. 4

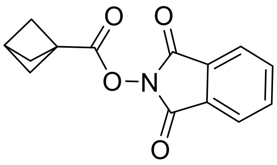

NHP ester 6
Ref. 11

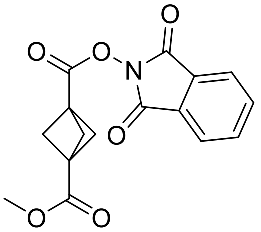

NHP ester 7
Ref. 8

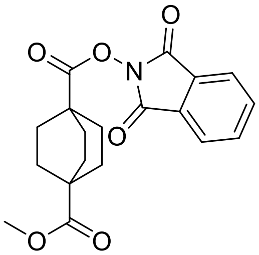

NHP ester 8
Ref. 7

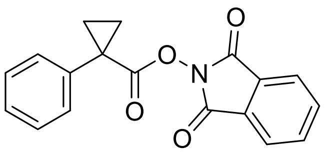

NHP ester 9
Ref. 3

To a round-bottom flask charged with a magnetic stir bar was added carboxylic acid (1.0 equiv), *N*-hydroxyphthalimide (1.0 equiv), *N,N*-dimethylaminopyridine (DMAP) (0.1 equiv), and dichloromethane (resulting in a solution 0.1 M in carboxylic acid). To this solution was added *N,N*-diisopropylcarbodiimide (DIC) (1.1 equiv) and the flask was capped with a rubber septum affixed with a vent needle. The resulting mixture was allowed to stir for at rt (20-22 °C)18 h. After this time, the reaction mixture was then filtered through a short pad of silica gel into a round bottom flask. The silica gel was rinsed with additional dichloromethane (~50 mL) into the flask. The solvent was removed under reduced pressure on a rotary evaporator. The crude material was recrystallized from hot methanol to afford the pure NHP ester. We and others have previously reported the synthesis of NHP esters shown below.

3.2.2 General Procedure B: Synthesis of NHP Esters Using PITU.

N-hydroxyphthalimide tetramethyluronium hexafluorophosphate[9] (PITU, 1.1 equiv) and N-methylmorpholine were added sequentially to a stirring 0.5 M solution of carboxylic acid (1.0 equiv) in N,N-dimethylformamide. The resulting mixture was stirred at room temperature (20-22 °C) for 16 h and then diluted with water (~0.2 M). Precipitated product, if formed, was collected

by filtration, washed with water (2× 10 mL) and dried under high vacuum to yield analytically pure product. Alternatively, the crude aqueous mixture was extracted with 1:1 EtOAc/Hexanes (×× 20 mL). The combined organic extracts were dried over Na_2SO_4, concentrated in vacuo and purified by flash column chromatography (FCC) to yield pure NHP ester.

3.3 General Procedures for Decarboxylative Cross-Electrophile Coupling

3.3.1 General Procedure C.

Reactions were set up in a N_2 filled glove box. For a preparative-scale benchtop procedure, see **3.3. Preparative-Scale Benchtop Procedure**. A catalyst solution was prepared by sequentially charging an oven dried scintillation vial with a PTFE-coated stirbar, $NiBr_2$(dme) (10.1 mg, 0.035 mmol, 7 mol%) and $^{t\text{-}Bu}$BpyCam^{CN} (11.6 mg, 0.035 mmol, 7 mol%). The solids were dissolved in DMA (0.64 mL) and the contents were stirred for 30 min, resulting in a homogeneous solution. A separate oven-dried 1-dram vial with a PTFE-coated stirbar was charged with NHP ester (0.50 mmol, 1.0 equiv), aryl halide (0.50 mmol, 1.0 equiv), zinc (65.4 mg, 1.0 mmol, 2.0 equiv), and 1,3,5-trimethoxybenzene (4.2 mg, 0.025 mmol) internal standard. To the vial containing NHP ester, aryl halide, zinc, and internal standard was added 0.64 mL of the prepared catalyst solution. The reactions were sealed with a screw cap fitted with a PTFE-faced silicone septum before being removed from the glovebox. The contents of the reaction vessel were stirred (1200 RPM) at r.t. (20-22 °C) for 24 h.

GC Analysis (modified below)
The reactions were monitored by GC analysis. Samples were prepared by the removal of a 25 μL aliquot of the crude reaction mixture with a gas-tight syringe. The aliquot was diluted with EtOAc (1.00 mL), then the resulting solution was filtered through a 2-cm celite plug in a Pasteur pipette into a 2 mL GC vial. The resulting solution was analyzed by GC and yields were determined based on the peak area of the analyte compared to 1,3,5-trimethoxybenzene as an internal standard.

Isolation and Purification (modified below)
Purification A. Unless otherwise indicated, reactions assembled for isolation were performed on a 0.5 mmol scale of NHP ester and aryl halide without the addition of an internal standard to avoid difficulties in separating 1,3,5-trimethoxybenzene from the cross-coupled product. Upon the completion of the reaction, the crude reaction mixture was diluted with DCM (5 mL) and slurried with 1–3 g of silica gel before the volatile solvents were removed by rotary evaporation. The adsorbed crude residue was purified by column chromatography on silica to provide the cross-coupled products.

3.3.2 General Procedure D. Ni-catalyzed Decarboxylative Cross-Electrophile Coupling without the use of a Glovebox.

Reactions were set up under an N_2 atmosphere using standard Schlenk techniques. A catalyst solution was prepared by sequentially charging an oven-dried 1 dram vial with a PTFE-coated stirbar, $NiBr_2$(dme (18.5 mg, 0.060 mmol, 20 mol%) and $^{t\text{-}Bu}$BpyCam^{CN} (20 mg, 0.060 mmol, 20 mol%). The vial was purged with N_2 (3×). DMA (50 µL) was added to give a blue-green slurry, followed immediately by THF (450 µL). The headspace was evacuated and purged again quickly with N_2 (3×) before being stirred at r.t. (20-22 °C) for 15 min, resulting in a homogeneous dark amber-colored solution.

A separate oven-dried 1-dram vial with a PTFE-coated stirbar was charged with NHP ester (0.30 mmol, 1.0 equiv), aryl halide (0.30 mmol, 1.0 equiv), zinc dust (Sigma-Aldrich, ≥98%, <10 μm, 39.2 mg, 600 μmol, 2.0 equiv). The vial was sealed with a screw cap fitted with a PTFE-faced silicone septum and purged with N_2 (3×). Finally, 0.50 mL of the prepared catalyst solution was added via syringe. The vial was immediately sealed with parafilm and the reaction mixture was stirred (1200 RPM) at r.t. (20-22 °C) for 24 h. (Note: Reactions can be run at higher dilution without affecting yields significantly).

GC/LCMS Analysis (combined with above)

The reactions were monitored by GC or LCMS analysis. GC Samples were prepared by the removal of a 25 μL aliquot of the crude reaction mixture with a gas-tight syringe. The aliquot was diluted with EtOAc (1.00 mL), then the resulting solution was filtered through a 2-cm celite plug in a Pasteur pipette into a 2 mL GC vial. The resulting solution was analyzed by GC and yields were determined based on the peak area of the analyte compared to 1,3,5-trimethoxybenzene as an internal standard.

LCMS samples were prepared by the removal of a 5 μL aliquot of the crude reaction mixture with a gas-tight syringe. The aliquot was diluted with MeOH (200 μL) and the resulting solution analyzed by LCMS. When required, triphenylamine was used as an internal standard (0.33 equiv) an added to the bulk of the reaction mixture prior to analysis.

Isolation and Purification (combine with above)

Purification Method A. Unless otherwise indicated, reactions assembled for isolation were performed without the addition of an internal standard to avoid difficulties in separating the internal standard from the cross-coupled product. Upon the completion of the reaction, the crude reaction mixture was diluted with DCM (5 mL) and slurried with 1–3 g of silica gel before the volatile solvents were removed by rotary evaporation. The adsorbed crude residue was purified by column chromatography on silica to provide the cross-coupled products. (Note: For highly basic compounds, extraction from dilute aq. NH_4OH $NH_4Cl/NaHCO_3$ may be used to decomplex zinc salts)

Purification Method B. Unless otherwise indicated, reactions assembled for isolation were performed without the addition of an internal standard to avoid difficulties in separating the internal standard from the cross-coupled product. Upon the completion of the reaction, the crude reaction mixture was quenched by the addition of 5 N aq. NH_4OH (750 μL) (Note: For sensitive substrates, sat'd. aq. NH_4Cl may be used, instead). The mixture was stirred at rt for 10 min, then diluted with DMA (2 mL), filtered through an Acrodisc® syringe filter (Note: Use of a centrifuge was employed as needed to avoid clogging), and purified by RP-HPLC (ACN/H_2O, 20 mM NH_4OH) to provide the cross-coupled products.

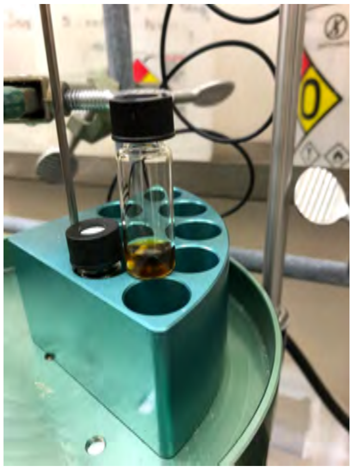

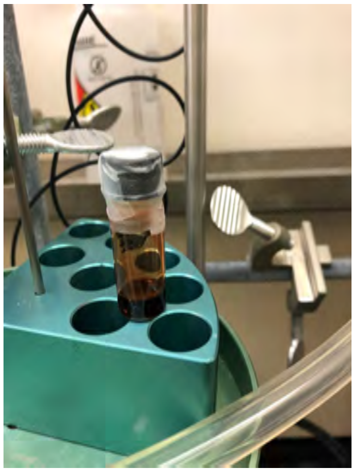

Formation of Ni catalyst solution in THF/DMA. **Final Reaction Mixture (t=24h).**

3.3.3 Preparative-Scale Benchtop Procedure

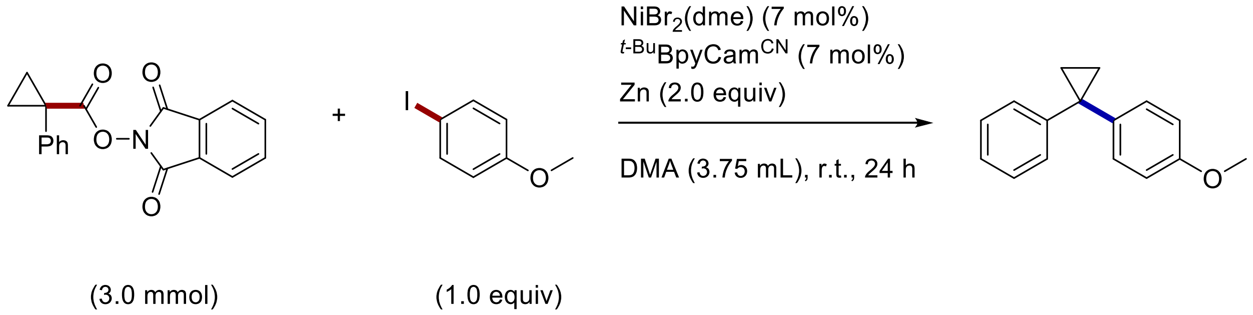

A catalyst solution was prepared on the benchtop by charging an oven-dried 20 mL scintillation vial with a PTFE-coated stirbar, $NiBr_2$•dme (65.1 mg, 0.21 mmol, 7 mol%), $^{t\text{-Bu}}$BpyCam^{CN} (70.4 mg, 0.21 mmol, 7 mol%). The scintillation vial sealed with a screw cap fitted with a PTFE-faced silicone septum. The scintillation vial was affixed with a N2 inlet line and a vent needle, and the headspace was purged with N2 for 10 min. Anhydrous DMA (3.75 mL) was added via syringe to the scintillation vial and the mixture was allowed to stir at rt for 30 min, resulting in a clear, homogeneous, dark orange solution. A separate oven dried 20 mL scintillation vial was charged with a PTFE-coated stirbar, 1,3-dioxoisoindolin-2-yl 1-phenylcyclopropane-1-carboxylate (922 mg, 3.0 mmol, 1.0 equiv), 4-iodoanisole (702 mg, 3.0 mmol, 1.0 equiv), and zinc (392 mg, 6.0 mmol, 2.0 equiv). The catalyst solution was transferred to the reaction vial via syringe, the vial containing the reaction mixture was affixed with a, N2 inlet line and a vent needle, the mixture was sparged with N_2 for 15 min, and the reaction mixture was allowed to stir at room temperature (20-22 °C) for 24 h.

Isolation and Purification
The reaction was diluted with DCM (60 mL) and the resulting solution was passed through a plug of silica gel and collected in a round bottom flask. The silica gel was washed twice with DCM (60 mL) and the resulting solution was slurried with silica gel (10 g). The slurry was concentrated under reduced pressure on a rotary evaporator. The resulting adsorbed crude residue was purified by column chromatography on silica to afford 1-methoxy-4-(1-phenylcyclopropyl)benzene as a clear, colorless oil (426 mg, 63% yield).

3.3.4 Decarboxylative Cross-Electrophile Coupling Under Continuous Flow Exemplified for the synthesis of 3-(4-methoxyphenyl)bicyclo[1.1.1]pentane-1-carboxylate (3w).

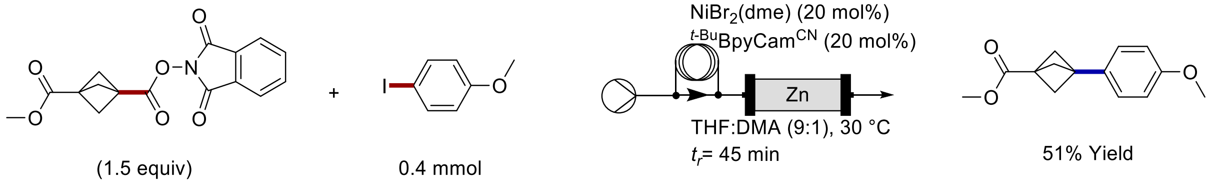

Note: This procedure was adapted from the literature[12] and modified for test-scale work using a syringe pump.

Step 1. Preparation of Zinc Column. Zinc ~30 mesh (Aldrich 565148) and Zinc ~325 mesh (Alfa-Aesar 13789) were throughly mixed in a 20 mL vial and then transferred to a 6 mm x 150 mm Omnifit column fitted with a fixed end piece at the bottom and a small amount of cotton wool at both ends. The column was capped with one fixed endpiece and one adjustable endpiece and attached to Vapourtec R2+R4 system. The column was flushed with THF (~2.5 mL column void volume) using a syringe pump. Note: A small amount of backpressure is observed.

Step 2. Activation of Zinc Column. An activating solution was prepared by adding chlorotrimethylsilane (800 µL) and 1-bromo-2-chloroethane (200 µL) to anhydrous THF (10 mL). The resulting solution was passed through the zinc column at 30 °C, at a flow rate of 1 mL/min. Once all of the activating solution had been passed through, anhydrous DMA (2 mL) was passed through the column to flush excess activating solution.

Step 3. Decarboxylative Cross-Electrophile Coupling in Flow. An oven-dried 2-dram vial was charged with a PTFE-coated stirbar, $NiBr_2$(dme) (24.7 mg, 0.080 mmol, 20 mol%), $^{t\text{-Bu}}$BpyCam^{CN} (29.5 mg, 0.088 mmol, 22 mol%). The vial was sealed with a screw cap fitted with a PTFE-faced silicone septum and purged with N2 (3×). DMA (50 µL) was added to give a blue-green slurry, followed promptly by THF (450 µL). The headspace was purged again quickly with N_2 (3×) before being stirred at r.t. (20-22 °C) for 15 min, resulting in a homogeneous dark amber-colored solution. To this solution was quickly added 4-iodoanisole (93.6 mg, 0.40 mmol, 1.0 equiv) and 1-(1,3-dioxoisoindolin-2-yl) 3-methyl bicyclo[1.1.1]pentane-1,3-dicarboxylate (189 mg, 0.60 mmol, 1.5 equiv) in one portion. After the end of the addition, the vial was immediately sealed with a PTFE-faced silicone septum and the headspace quickly purged with N_2 (3×). The mixture was stirred at room temperature for 10 min to give a homogenous solution. The reactant solution thus formed was passed through the activated column from Step 2 at 30 °C, at a flow rate of 0.1 mL/min. Product was observed as a red-colored solution which was collected after the first ~2 mL of reaction solution had been flowed through. After the end of the addition, DMA (3 mL) was flowed through the column to complete the elution of product. Product was collected until the eluting

solution became colorless (final product solution volume ~6 mL). This set-up gave a residence time through the zinc column, t_r, of approximately 45 min.

Step 4. Isolation of Cross-Coupled Product. The obtained product solution was diluted with sat'd aq. NH_4Cl (5 mL) and sat'd aq. $NaHCO_3$ (5 mL). The resulting aqueous mixture was extracted with 1:1 EtOAc/Hex (3×10 mL). The combined organic extracts were concentrated *in vacuo* and purified by flash chromatography (0-20% EtOAc/Hex) using 12 g silica to afford methyl 3-(4-methoxyphenyl)bicyclo[1.1.1]pentane-1-carboxylate (**3z**, 47.3 mg, 204 µmol, 51%) as a semi-crystalline white solid. Analytical data was in accordance with that reported in entry **3x**. Note: No further optimization of conditions was investigated at this time.

3.3.5 Decarboxylative Cross-Electrophile Coupling in a High-Throughput Experimentation (HTE) Mode

3.3.5.1 Coupling to X-Bromo *N*-methyl indazole

Catalyst Stock Solutions

Stock solutions of catalyst for HTE screening were prepared in a N_2-filled glovebox in separate dram vials. An oven dried dram vial equipped with a PTFE-coated stir bar was sequentially charged with $NiBr_2$dme, ligand, and THF (1.0 mL). The vial was capped with a Teflon-coated screw cap and the contents stirred at rt for 30 min to afford a (0.08 M) catalyst stock solution.

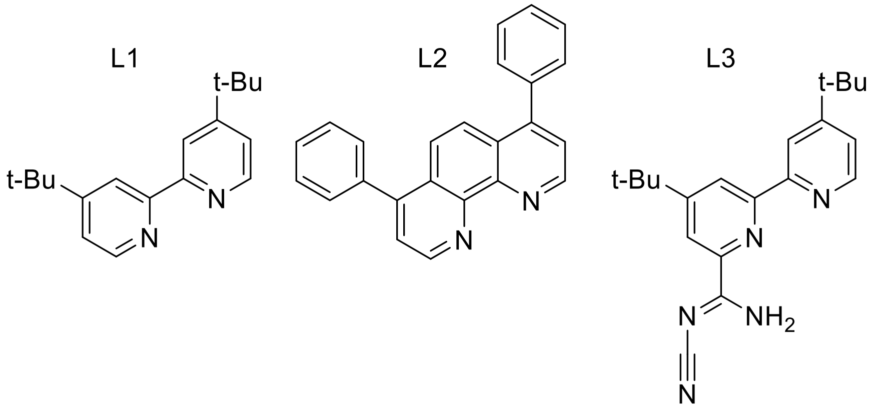

Solution 1: $NiBr_2$dme (23.4 mg, 0.08 mmol, 0.2 equiv) and dtbbpy (21.4 mg, 0.08 mmol, 0.2 equiv).

Solution 2: $NiBr_2$dme (23.4 mg, 0.08 mmol, 0.2 equiv) and bathophenanthroline (26.6 mg, 0.08 mmol, 0.2 equiv).

Solution 3: $NiBr_2$dme (23.4 mg, 0.08 mmol, 0.2 equiv), ^{tbu}bpyCAM^{CN} (26.8 mg, 0.08 mmol, 0.2 equiv)

Stock Solutions of NHP Esters

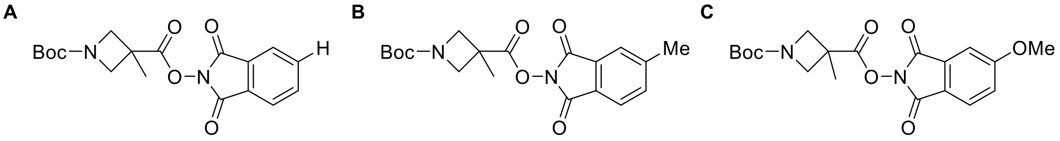

Note: Due to the poor solubility of these NHP esters in THF, the prepared stock solutions were made more dilute than the standard reaction conditions to afford homogeneous solutions. Eight NHP esters were chosen for high-throughput screening. Stock solutions of NHP esters **A-C**, sufficient for 40 reactions at a 0.01 mmol scale, were prepared in a N_2-filled glovebox by weighing each NHP ester (0.4 mmol) into a dram vial followed by addition of anhydrous THF (2.0 mL), before sealing the vial with a PTFE-lined cap and briefly shaking the mixture by hand for 30 seconds.

Stock Solutions of Bromo Indazoles

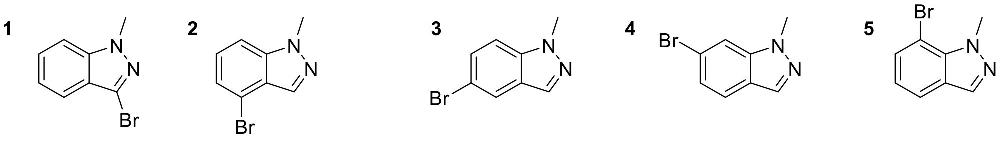

Note: Due to the poor solubility of these aryl bromides esters in THF, the prepared stock solutions were made more dilute than the standard reaction conditions to afford homogeneous solutions Five aryl bromide cores were chosen for high-throughput screening. In a N_2-filled glovebox, a stock solution of aryl bromides **1-3**, sufficient for 30 reactions at a 0.01 mmol scale, was prepared by weighing aryl bromide **1-3** (0.3 mmol) into a dram vial followed by the addition of anhydrous THF (1.5 mL) and briefly shaking the mixture by hand for 30 seconds until completely homogenous.

Preparation of Zinc-Coated ChemBeads

Zinc-coated ChemBeads (5% w/w) were prepared following a literature procedure. In a N_2-filled glovebox, to a 20-mL scintillation vial was charged 22.8 g of glass ChemBeads and 1.2 g (9.1 mmol) Zn. The vial was sealed, then removed from the glovebox. The vial was placed on a conical vortex mixer and agitated for 30 minutes to ensure even coating of the beads.

Preparation of Internal Standard Stock Solution

In a N_2-filled glovebox, an oven dried dram vial was sequentially charged with1,3,5-trimethoxybenzene (201.8 mg, 1.2 mmol) and THF (1.8 mL). The vial was capped with a Teflon-coated screw cap and the vial was shaken by hand for 30 seconds, resulting in a homogeneous solution

General Procedure for HTE Screening

All operations were performed in a N_2-filled glovebox. To each well of a 96-well (8 rows by 12 columns) aluminium block assembly equipped with 8 × 30 mm vials was dosed 30 mg of Zn-coated ChemBeads (5% loading wt/wt) using a calibrated scoop and a non-static funnel (Image 2). NHP Ester and aryl bromide substrates were dosed into each well by first transferring stock

solutions of each NHP ester and aryl bromide into separate channels of eight-channel polypropylene deep-well reservoirs followed by transferring 50 μL of each NHP ester stock solution to their respective wells (Image 3) and 50 μL of each aryl bromide stock solutions to their respective wells (Image 3) using a multi-channel pipette. To each well was added 15 μL of a stock solution of trimethoxybenzene internal standard and 25 uL of a stock solution of catalyst (Image 3). 15 uL of DMA were added to the appropriate wells (see table below). The well-plate vials were sealed with an electric screwdriver at torque setting 6 in a diagonal pattern (Image 4), using an aluminum lid, and the block was placed onto a heater/shaker (Torrey Pines Echotherm) set at 60 °C (actual temperature was found to be ~20 °C lower) and orbital speed at 8 to heat/shake for 36 h (Image 5).

	THF					THF:DMA					THF	THF:DMA	
	Col 1	Col 2	Col 3	Col 4	Col 5	Col 6	Col 7	Col 8	Col 9	Col 10	Col 11	Col 12	
Row 1	A, 1	A, 2	A, 3	A, 4	A, 5	A, 1	A, 2	A, 3	A, 4	A, 5	C, 1	C, 1	
Row 2	B, 1	B, 2	B, 3	B, 4	B, 5	B, 1	B, 2	B, 3	B, 4	B, 5	C, 2	C, 2	
Row 3	C, 1	C, 2	C, 3	C, 4	C, 5	C, 1	C, 2	C, 3	C, 4	C, 5	C, 3	C, 3	
Row 4	A, 1	A, 2	A, 3	A, 4	A, 5	A, 1	A, 2	A, 3	A, 4	A, 5	C, 4	C, 4	
Row 5	B, 1	B, 2	B, 3	B, 4	B, 5	B, 1	B, 2	B, 3	B, 4	B, 5	C, 5	C, 5	
Row 6	C, 1	C, 2	C, 3	C, 4	C, 5	C, 1	C, 2	C, 3	C, 4	C, 5			L1
Row 7	A, 1	A, 2	A, 3	A, 4	A, 5	A, 1	A, 2	A, 3	A, 4	A, 5			L2
Row 8	B, 1	B, 2	B, 3	B, 4	B, 5	B, 1	B, 2	B, 3	B, 4	B, 5			L3

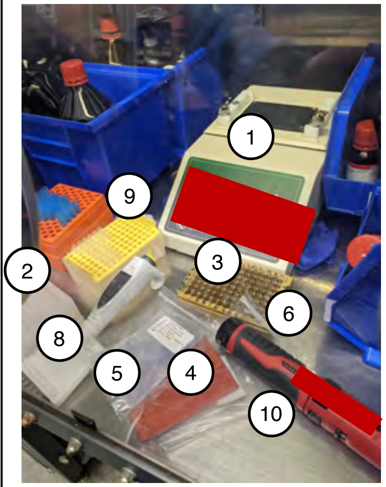

Image 1. HTE Equipment with prepared NHP ester and aryl bromide stock

Setup Parts (SKU#)

1. Torrey Pines Ectotherm Shaker/Heater
2. 8-Channel Deep Well Reservoir (32008)
3. Paradox 96-Well Photoredox Block Assembly (96973) loaded with 8×30mm shell vials (884001)
4. Rubber mat (96965)
5. Plastic film linings (96967)
6. Abbvie calibrated ChemBead scoops
7. Non-static funnel
8. Sartorius Picus electronic 8-channel pipette, 5 – 120 µL
9. Sartorius 0.5 – 200 µL pre-sterilized Optifit Tips (790200)
10. Milwaukee cordless screwdriver (2101-21)

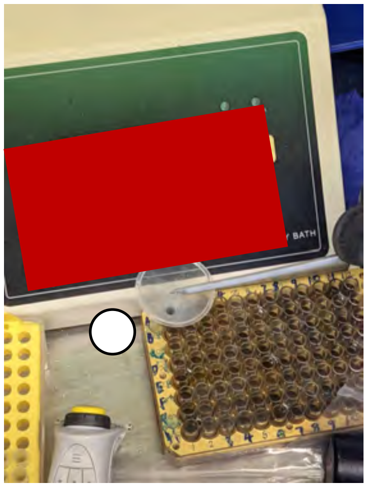

Image 2. Addition of zinc-coated ChemBeads

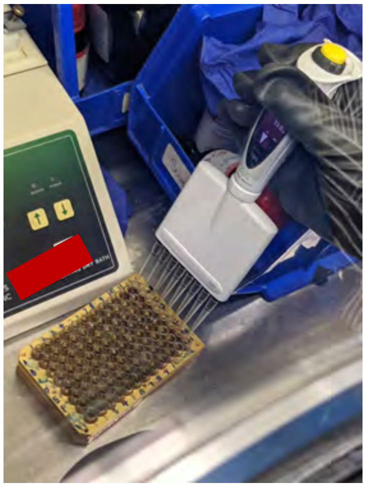

Image 3. Addition of stock solutions

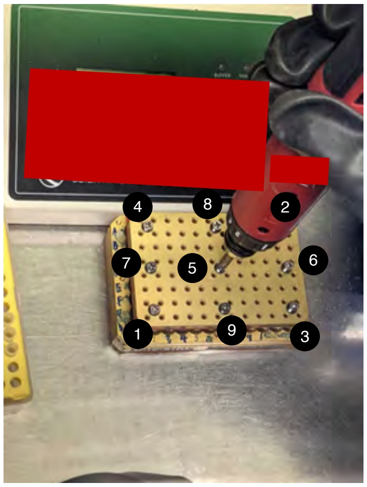

Image 4. Sealing plate in the pattern shown at torque setting of 6.

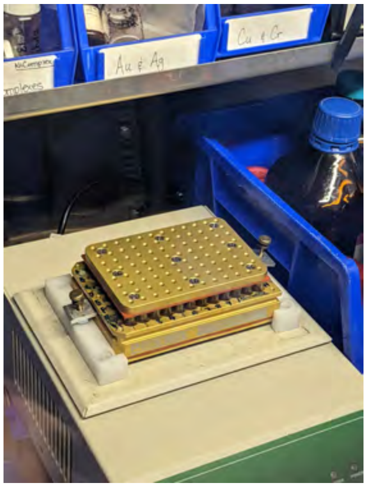

Image 5. Heating/shaking the block assembly in a glovebox for 36 hours.

Workup and Analysis

The reaction block was removed from the shaker apparatus and allowed to cool to rt. The aluminum block was then removed from the glovebox, the lid was removed, and 200 µL of MeOH was added to each well to dilute the reaction mixtures. 150 µL aliquots were taken from each well and were then filtered through a 0.2 µm filter plate into a 340 µL 96-well collection plate (Images 6 and 7). Each well of the filter plate was then washed with an additional 120 µL of MeOH The plate was then analyzed by UPLC-MS, product to internal standard ratios were determined by absorbance at 254 nm.

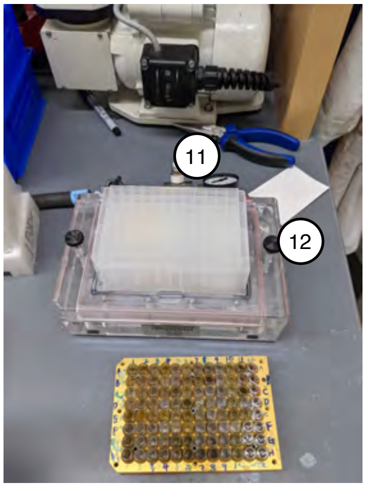

Image 6. Filtration setup into a 96-well collection plate.

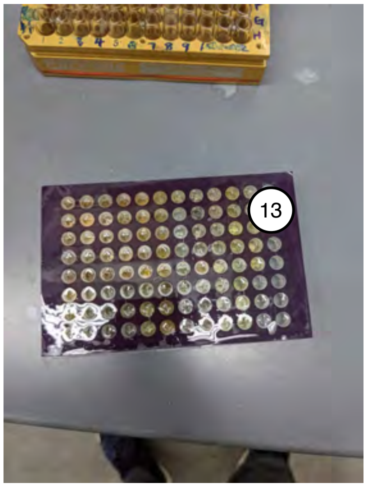

Image 7. Finished Plate sealed with a round well cap mat for SFC-MS analysis

Workup Parts (SKU#)

11. 2mL Hydrophilic filter plate, 0.2µm polypropylene membrane (96222-10)
12. Vacuum manifold filtration system (96844)
13. 340µL 96-well collection plate with V-shaped well bottoms and SiliGuard low binding coating (96340GC) with clear adhesive sealing film (Analytical Sales 961802)

3.3.5.2 HTE Aryl halide chemistry informer library compound X5

Catalyst Stock Solutions

Stock solutions of catalyst for HTE screening were prepared in a N_2-filled glovebox in separate dram vials. An oven dried dram vial equipped with a PTFE-coated stir bar was sequentially charged with $NiBr_2$dme, ligand, and THF (0.75 mL). The vial was capped with a Teflon-coated screw cap and the contents stirred at rt for 30 min to afford a (0.067 M) catalyst stock solution.

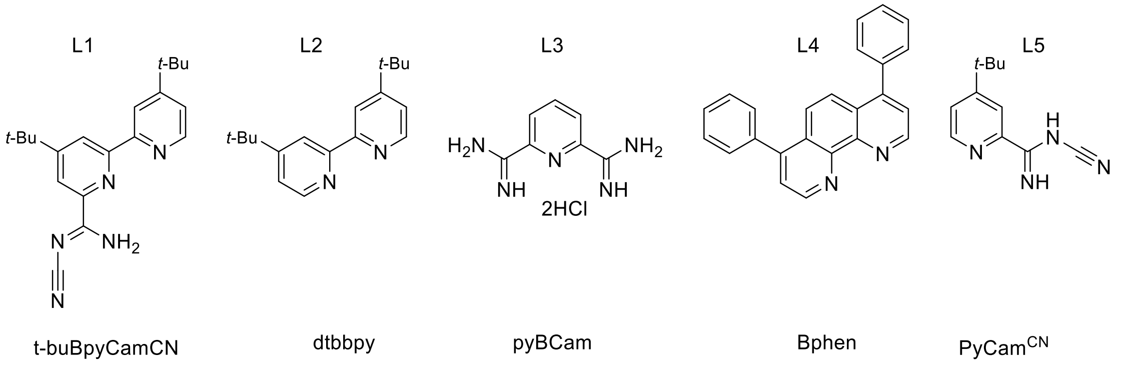

Solution 1: $NiBr_2$dme (14.6 mg, 0.05 mmol, 0.2 equiv), ^{tbu}bpyCAM^{CN} (16.8 mg, 0.05 mmol, 0.2 equiv)

Solution 2: $NiBr_2$dme (14.6 mg, 0.05 mmol, 0.2 equiv) and dtbbpy (13.4 mg, 0.05 mmol, 0.2 equiv).

Solution 3: $NiBr_2$dme (14.6 mg, 0.05 mmol, 0.2 equiv) and bathophenanthroline (16.6 mg, 0.05 mmol, 0.2 equiv).

Solution 4: $NiBr_2$dme (14.6 mg, 0.05 mmol, 0.2 equiv) and Pyridine-2,6-bis(carboximidamide) dihydrochloride (11.8 mg, 0.05 mmol, 0.2 equiv).

Solution 5: $NiBr_2$dme (14.6 mg, 0.05 mmol, 0.2 equiv) and 4-(tert-butyl)-N-cyanopicolinimidamide (10.1 mg, 0.05 mmol, 0.2 equiv).

Stock Solutions of NHP Esters

A

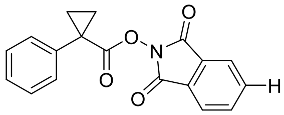

B

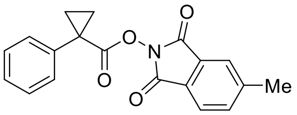

C

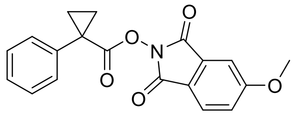

Stock solutions of NHP esters **A-C**, sufficient for 25 reactions at a 0.01 mmol scale, were prepared in a N_2-filled glovebox by weighing each NHP ester (0.25 mmol) into a dram vial followed by addition of anhydrous THF (2.25 mL), before sealing the vial with a PTFE-lined cap and briefly shaking the mixture by hand for 30 seconds. Stock solutions were prepared immediately prior to use in HTE screening studies.

Stock Solution of Aryl Bromide

1

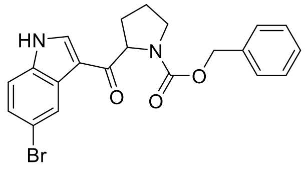

Note: Due to the poor solubility of this aryl bromide in THF, the prepared stock solutions were made more dilute than the standard reaction conditions to afford homogeneous solutions 5, sufficient for 20 reactions at a 0.01 mmol scale were prepared by weighing the aryl bromide (0.2 mmol) into a dram vial before addition of anhydrous THF (2.4 mL) and briefly shaking the mixture by hand for 30 seconds until completely homogenous.

Preparation of Zinc-Coated ChemBeads

Zinc-coated ChemBeads (5% w/w) were prepared following a literature procedure. In a N_2-filled glovebox, to a 20-mL scintillation vial was charged 22.8 g of glass ChemBeads and 1.2 g (9.1 mmol) Zn. The vial was sealed, then removed from the glovebox. The vial was placed on a conical vortex mixer and agitated for 30 minutes to ensure even coating of the beads.

Preparation of Internal Standard Stock Solution

In a N_2-filled glovebox, an oven dried dram vial was sequentially charged with1,3,5-trimethoxybenzene (201.8 mg, 1.2 mmol) and THF (1.8 mL). The vial was capped with a Teflon-coated screw cap and the vial was shaken by hand for 30 seconds, resulting in a homogeneous solution

General Procedure for HTE Screening

All operations were performed in a N_2-filled glovebox. To each well of a 96-well (8 rows by 12 columns) aluminium block assembly equipped with 8 × 30 mm vials was dosed 30 mg of Zn-coated ChemBeads (5% loading wt/wt) using a calibrated scoop and a non-static funnel (Image 2). The NHP esters and aryl bromide were dosed into each well by first transferring stock solutions of each NHP ester and aryl bromide into separate channels of eight-channel polypropylene deep-well reservoirs followed by transferring 90 µL of each NHP ester stock solution to their respective wells and 60 µL of the aryl bromide stock solutions to each well using a multi-channel pipette. To each well was added 15 µL of a stock solution of trimethoxybenzene internal standard (1.68 mg, 0.01 mmol, 1 equiv) in THF and 30 uL of a stock solution of catalyst. The well-plate vials were sealed with an electric screwdriver at torque setting 6 in a diagonal pattern (Image 4), using an aluminum lid, and the block was placed onto a heater/shaker (Torrey Pines Echotherm) set at 60 °C (actual temperature was found to be ~20 °C lower) and orbital speed at 8 to heat/shake overnight for 36 h.

<u>Workup and Analysis</u>

The reaction block was removed from the shaker apparatus and allowed to cool to rt. The aluminum block was then removed from the glovebox, the lid was removed, and 200 µL of MeOH was added to each well to dilute the reaction mixtures. 150 µL aliquots were taken from each well and were then filtered through a 0.2 µm filter plate into a 340 µL 96-well collection plate (Images 6 and 7). Each well of the filter plate was then washed with an additional 120 µL of MeOH The plate was then analyzed by UPLC-MS, product to internal standard ratios were determined by absorbance at 254 nm.

Figure S10. HTE coupling of Aryl halide chemistry informer library compound X5

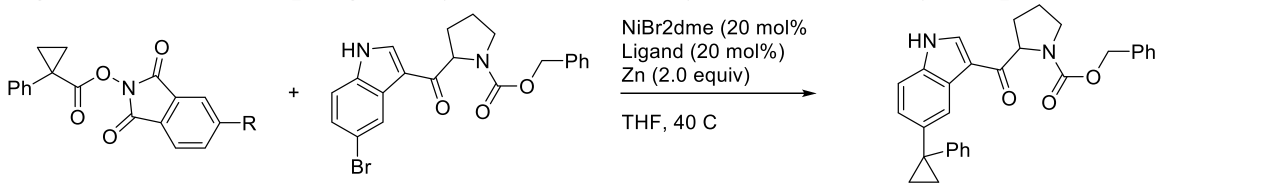

	$^{t\text{-bu}}$BpyCam^{CN}	dtbbpy	PyBCam	Bphen	PyCam^{CN}
R=H	7.96	0.82	2.06	0.30	0.46
R=Me	10.27	0.14	2.05	0.61	0.85
R=OMe	0.63	0.36	0.42	0.39	0.40

4. Specific Procedures and Product Characterization

4.1 Synthesis of 4,4'-di-*tert*-butyl-*N*-cyano-2,2'-bipyridine-6-carboximidamide (L7)

4,4'-di-*tert*-butyl-2,2'-bipyridine-1-oxide

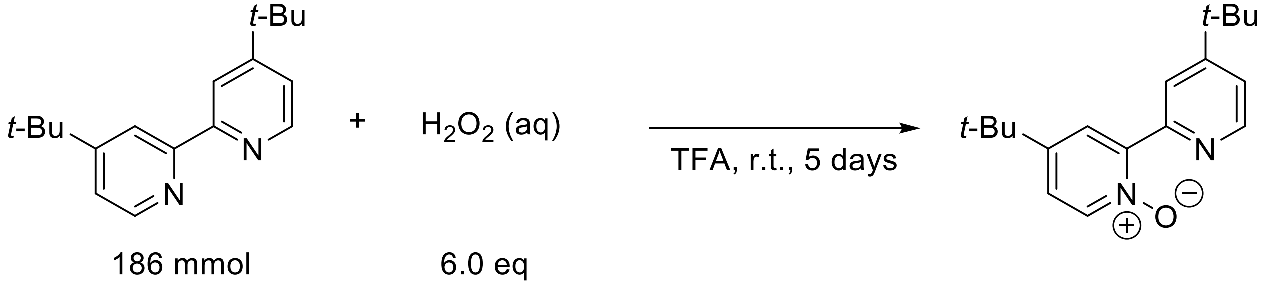

To a 1 L round-bottom flask equipped with a PTFE-coated stir bar was charged with 4,4′-di-tert-butyl-2,2′-bipyridine (50 g, 186 mmol, 1.0 equiv) and 125 mL of trifluoroacetic acid. The flask was placed in a water bath (to help control any potential exotherms) and the solution stirred. Aqueous H_2O_2 (30 mL of 30% solution, 279 mmol, 1.5 equiv) was added to the stirring solution in a steady stream and the resulting mixture was left to stir at r.t. (20-22 °C). Reaction progress was monitored via SFC-MS, and the presence of trifluoroperacetic acid was monitored using KI starch paper. Additional equivalents of H_2O_2 (30 mL of 30% solution, 279 mmol, 1.5 equiv) were added until the reaction was judged complete by SFC-MS as determined by the complete consumption of the 4,4′-di-tert-butyl-2,2′-bipyridine starting material. The reaction was quenched with the addition of $CHCl_3$ (400 mL). The resulting mixture was neutralized by the slow addition of aqueous 6 M NaOH (pH of 7). The resulting two layers were separated. The organic layer was washed with additional aqueous 6 M NaOH (2 × 200 mL). Finally, the organic layer was dried over Mg_2SO_4. After filtration to remove the drying agent, the filtrate was concentrated on a rotary evaporator under vacuum. A white powder was obtained which was further used in the next step without purification. Yield: 51 g (97%) of 95% pure material (95% *N*-oxide, 5% rsm).[13]

^{1}H NMR (500 MHz, $CDCl_3$) δ 8.91 (dd, *J* = 1.9, 0.8 Hz, 1H), 8.63 (dd, *J* = 5.3, 0.8 Hz, 1H), 8.23 (d, *J* = 6.9 Hz, 1H), 8.08 (d, *J* = 2.9 Hz, 1H), 7.33 (dd, *J* = 5.2, 2.0 Hz, 1H), 7.25 (dd, *J* = 6.9, 3.0 Hz, 1H), 1.37 (s, 9H), 1.37 (s, 9H).
^{13}C{^{1}H} NMR (126 MHz, $CDCl_3$) δ 160.2, 150.3, 150.1, 149.2, 146.7, 139.9, 124.7, 122.9, 122.5, 121.3, 35.0, 34.7, 30.6, 30.6.

4,4'-di-*tert*-butyl-2,2'-bipyridine-6-carbonitrile

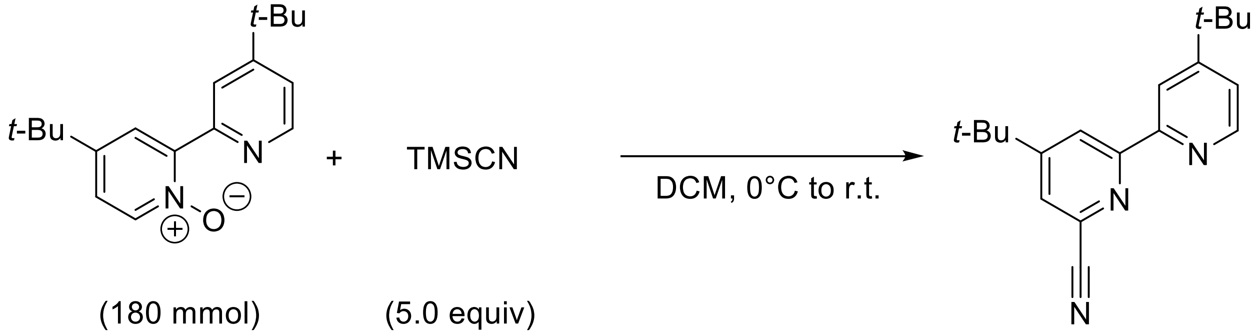

An oven-dried 500 mL 3-neck flask equipped with a PTFE-coated stirbar, an in situ thermometer, an addition funnel, was charged with 4,4′-di-tert-butyl2,2′-bipyridin-N-oxide (51 g, 180 mmol) and cooled under vacuum. The flask was placed under a N_2 atmosphere and dry, degassed CH_2Cl_2 (400 mL) was added via cannula, and the flask was lowered into an ice bath and cooled to 0 °C (determined by the in situ thermometer). Trimethylsilylcyanide (89 g, 900 mmol, 5.00 equiv) was added to the addition funnel via syringe and slowly added to this solution followed via the addition funnel. Benzoyl chloride (42 mL, 50.82 g, 361 mmol, 2.00 equiv) was added to the addition funnel, then added slowly to the reaction mixture to prevent any exotherms (as measured by the in situ thermometer. After complete addition, the mixture was warmed to rt (20-22 °C) and stirred for another 24 h. Then a 10% sodium hydrogencarbonate solution was added with caution until gas evolution has stopped. The resulting two-phase system was stirred for 24 h at rt. After this time, the mixture was transferred to a separatory funnel. Then, the two layers were separated and the aqueous layer was washed twice with CH_2Cl_2. Finally, the combined organic layers were dried over magnesium sulphate. After filtration, the solvent was removed in vacuum. The slightly brown residue was dried and recrystallized from hot *i*-PrOH. After drying under vacuum a white powder was obtained. Yield: 42 g (144 mmol, 80%).[13]

^{1}H NMR (500 MHz, CDCl3) δ 8.66 (d, J = 1.8 Hz, 1H), 8.59 (dd, J = 5.2, 0.8 Hz, 1H), 8.46 (d, J = 1.8 Hz, 1H), 7.69 (d, J = 1.8 Hz, 1H), 7.36 (dd, J = 5.2, 2.0 Hz, 1H), 1.41 (s, 9H), 1.40 (s, 9H). **^{13}C{^{1}H} NMR** (126 MHz, CDCl3) δ 162.5, 161.5, 158.0, 154.4, 149.2, 133.2, 125.6, 121.7, 121.5, 118.8, 118.0, 53.4, 35.4, 35.1, 30.6, 30.4.

4,4′-di-tert-butyl-6-N-cyanocarboxamidine-2,2-bipyridine ($^{t\text{-Bu}}$BpyCam^{CN})

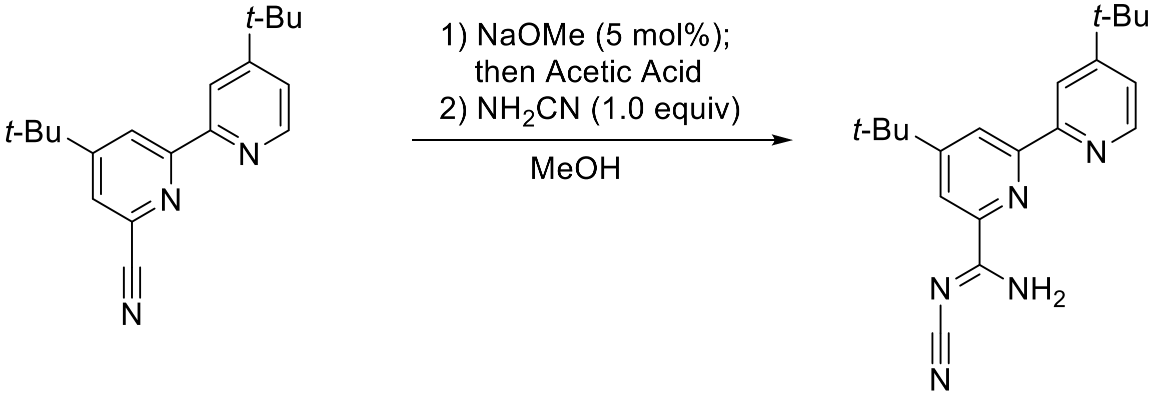

A flame-dried 1000 mL round-bottom flask equipped with a PTFE-coated stirbar was charged with 4,4'-di-tert-butyl-[2,2'-bipyridine]-6-carbonitrile (42.33 g, 144.3 mmol, 1.00 equiv) and sodium methoxide (42.33 g, 7.2 mmol, 0.05 equiv), and capped with a rubber suptem affixed with a N_2 inlet needle. The flask was evacuated and back-filled with N_2 three times, before addition of methanol (577 mL) via syringe. The reaction flask was lowered into an oil bath heated to 60 °C and left stirring for several hours until the formation of imidate was judged complete by SFC-MS. The oil bath was then set to 40 °C and the reaction mixture was stirred for an additional 2 hours, at which point a white precipitate formed. The mixture was quenched with acetic acid (0.41 mL, 7.2 mmol, 0.05 equiv) followed by addition of cyanamide (6.07 g, 144.3 mmol, 1.00 equiv) as a solution in 28.9 mL of methanol via syringe. The reaction mixture was then heated back to 60 °C and left stirring for several hours until the reaction was judged complete by SFC-MS. The reaction mixture was cooled to room temperature and filtered through a medium porosity fritted-glass funnel into a round bottom flask. The collected solids in the filter were analytically pure 4,4'-di-*tert*-butyl-*N*-cyano-2,2'-bipyridine-6-carboximidamide. The filtrate, containing additional product, was then concentrated in vacuo and the crude product recrystallized by completely dissolving the crude solid in THF followed by careful layering of hexanes. Characterization data matched those reported in the literature.[14] Yield: 35 g (104 mmol, 72% yield).[15]

^{1}H NMR (500 MHz, $CDCl_3$) δ 8.64 (d, *J* = 1.8 Hz, 1H), 8.63 (dd, *J* = 5.3, 0.8 Hz, 1H), 8.42 (s, 1H), 8.31 (d, *J* = 1.8 Hz, 1H), 8.30 (dd, *J* = 2.0, 0.7 Hz, 1H), 7.37 (dd, *J* = 5.2, 1.9 Hz, 1H), 6.75 (s, 1H), 1.43 (s, 9H), 1.41 (s, 9H).
^{13}C{^{1}H} NMR (126 MHz, $CDCl_3$) δ 166.5, 163.4, 161.2, 156.1, 155.0, 149.4, 146.5, 122.4, 121.5, 119.7, 118.0, 116.2, 35.5, 35.0, 30.6, 30.6.
HRMS (ESI) m/z calculated for $C_{20}H_{26}N_5$ $[M+H]^+$ 336.21827, found 336.2179.
M.P. (°C) 272 – 275.

tert-butyl 1-(3,5,5,8,8-pentamethyl-5,6,7,8-tetrahydronaphthalen-2-yl)cyclopropane-1-carboxylate (SI-4)

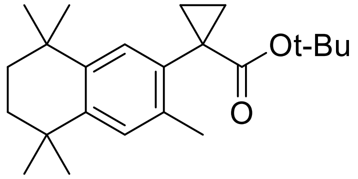

The following procedure was adapted from the method of Hartwig and coworkers. To a 20 mL scintillation vial containing solid $LiNCy_2$ (702 mg, 3.75 mmol, 2.1 equiv) was added dropwise a solution of tert-butyl cyclopropanecarboxylate (511.9 mg, 3.600 mmol, 2 equiv) in toluene (5.4 mL) while stirring for 15 minutes. Meanwhile, to a separate 20 mL scintillation vial was charged $AgBF_4$ (18 mg, 0.09 mmol, 0.05 equiv), Pd(1-tBu-Indenyl)($PtBu_3$)(Cl) (46.8 mg, 0.09 mmol, 0.05 equiv), 6-bromo-1,1,4,4,7-pentamethyl-1,2,3,4-tetrahydronaphthalene (506.4 mg, 1.800 mmol, 1 equiv), and toluene (1.8 mL). The resulting mixture was shaken by hand for 30 seconds before transferring the enolate solution into the vial. The vial was sealed with a screw cap fitted with a PTFE-faced silicone septum and removed from the glovebox. The reaction mixture was left stirring at 65 °C for 5 hours. After the specified time, the reaction mixture was concentrated *in vacuo* and directly loaded onto silica gel for column chromatography to afford the product as a white solid (0.45 g, 76% yield)
^{1}H NMR (500 MHz, $CDCl_3$) δ 7.10 (s, 1H), 7.03 (s, 1H), 2.26 (s, 3H), 1.64 (s, 4H), 1.57 – 151 (m, 2H), 1.35 (s, 9H), 1.25 (s, 6H), 1.24 (s, 6H), 1.11 – 1.08 (m, 2H).

$^{13}C\{^{1}H\}$ NMR (126 MHz, $CDCl_3$) δ 173.6, 143.2, 141.6, 135.5, 135.4, 128.2, 127.7, 80.0, 35.3, 35.3, 33.9, 33.9, 31.9, 31.8, 28.5, 27.9, 19.0, 16.4.
HRMS (ESI) m/z calculated for $C_{23}H_{35}O_2$ $[M+H]^+$ 343.2632, found 343.2624.
MP = 117 – 119 °C.

1-(3,5,5,8,8-pentamethyl-5,6,7,8-tetrahydronaphthalen-2-yl)cyclopropane-1-carboxylic acid (SI-5)

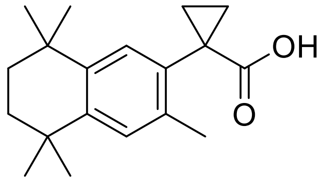

To a 20 mL scintillation vial under N_2 atmosphere was charged tert-butyl 1-(3,5,5,8,8-pentamethyl-5,6,7,8-tetrahydronaphthalen-2-yl)cyclopropane-1-carboxylate (814.1 mg, 2.47 mmol, 1.0 equiv) and anhydrous DCM (5.5 mL). To the vial was then added Et_3SiH (747 mg, 1.03 mL, 6.4 mmol, 2.6 equiv) followed by trifluoroacetic acid (3.67 g, 2.47 mL, 32.2 mmol, 13 equiv). The reaction mixture was left stirring at room temperature until complete conversion of the starting material was observed by TLC. The solvent was removed *in vacuo* to afford the product as a white solid (0.642 g, 91% yield).
^{1}H NMR (500 MHz, $CDCl_3$) δ 7.12 (s, 1H), 7.05 (s, 1H), 2.28 (s, 3H), 1.71 – 167 (m, 2H), 1.64 (s, 4H), 1.25 (s, 6H), 1.23 (s, 6H), 1.23 – 1.19 (m, 2H).
$^{13}C\{^{1}H\}$ NMR (126 MHz, $CDCl_3$) δ 180.4, 144.0, 142.0, 135.7, 134.0, 128.4, 128.0, 35.2, 33.9, 31.9, 31.8, 27.3, 19.0, 18.2.
HRMS (ESI) m/z calculated for $C_{19}H_{25}O_2$ $[M-H]^-$ 285.1860, found 285.1861.
MP = 228 – 230 °C.

1-(thiophen-3-yl)cyclopropane-1-carboxylic acid (SI-6)

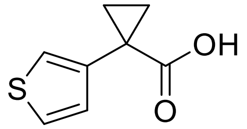

To a 20 mL scintillation vial under N_2 atmosphere was charged tert-butyl 1-(thiophen-3-yl)cyclopropane-1-carboxylate (164 mg, 0.73 mmol, 1.0 equiv) and anhydrous DCM (1.6 mL). To the vial was then added Et_3SiH (220.7 mg, 0.3 mL, 1.9 mmol, 2.6 equiv) followed by trifluoroacetic acid (1.08 g, 1.8 mL, 9.5 mmol, 13.0 equiv). The reaction mixture was left stirring at room temperature until complete conversion of the starting material was observed by TLC. The solvent was removed *in vacuo* to afford the product as a white solid (0.121 g, 99% yield). Characterization data matched those reported in the literature.[16]

1-(benzo[b]thiophen-5-yl)cyclopropane-1-carboxylic acid (SI-7)

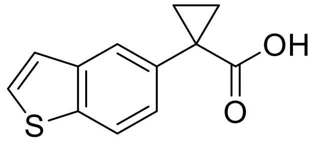

To a 20 mL scintillation vial under N_2 atmosphere was charged tert-butyl 1-(benzo[b]thiophen-5-yl)cyclopropane-1-carboxylate (419.6 mg, 1.9 mmol, 1.0 equiv) and anhydrous DCM (4.2 mL). To the vial was then added Et_3SiH (565.1 mg, 0.78 mL, 4.9 mmol, 2.6 equiv) followed by

trifluoroacetic acid (2.77 g, 1.86 mL, 24.3 mmol, 13.0 equiv). The reaction mixture was left stirring at room temperature until complete conversion of the starting material was observed by TLC. The solvent was removed *in vacuo* to afford the product as a white solid (0.360 g, 88% yield).
^{1}H NMR (500 MHz, $CDCl_3$) δ 7.82 (dd, J = 8.3, 0.8 Hz, 2H), 7.79 (d, J = 1.6 Hz, 1H), 7.43 (d, J = 5.4 Hz, 1H), 7.36 (dd, J = 8.3, 1.7 Hz, 1H), 7.29 (dd, J = 5.5, 0.6 Hz, 1H), 1.75 – 1.69 (m, 2H), 1.36 – 1.30 (m, 2H).
^{13}C{^{1}H} NMR (126 MHz, $CDCl_3$) δ 179.9, 139.6, 139.0, 134.9, 127.1, 127.0, 125.2, 123.7, 122.3, 28.7, 17.6.
HRMS (ESI) m/z calculated for $C_{12}H_9O_2S$ $[M-H]^-$ 217.0329, found 217.0329.
MP = 206 – 208 °C.

1-(2,6-dimethoxypyridin-3-yl)cyclopropane-1-carboxylic acid (SI-8)

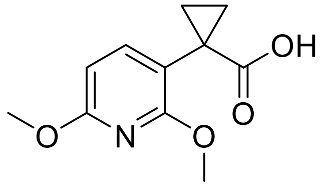

To a 20 mL scintillation vial under N_2 atmosphere was charged tert-butyl 1-(2,6-dimethoxypyridin-3-yl)cyclopropane-1-carboxylate (595.1 mg, 2.13 mmol, 1 equiv) and anhydrous DCM (4.73 mL). To the vial was then added Et_3SiH (644.0 mg, 0.884 mL, 5.5 mmol, 2.6 equiv) followed by trifluoroacetic acid (3.16 g, 2.12 mL, 27.7 mmol, 13.0 equiv). The reaction mixture was left stirring at room temperature until complete conversion of the starting material was observed by TLC. The solvent was removed *in vacuo* to afford the product as a white solid (0.470 g, 99% yield).
^{1}H NMR (500 MHz, $CDCl_3$) δ 7.37 (d, J = 8.0 Hz, 1H), 6.23 (d, J = 8.0 Hz, 1H), 3.94 (s, 3H), 3.89 (s, 3H), 1.67 – 1.61 (m, 2H), 1.14 – 1.08 (m, 2H).
^{13}C{^{1}H} NMR (126 MHz, $CDCl_3$) δ 180.3, 162.4, 161.8, 141.9, 112.4, 100.1, 53.5, 53.5, 23.5, 17.5.
HRMS (ESI) m/z calculated for $C_{11}H_{12}NO_4$ $[M-H]^-$ 222.0772, found 222.0771.
MP = 180 – 182 °C.

tert-butyl acetyl(4-iodophenyl)carbamate (SI-9)

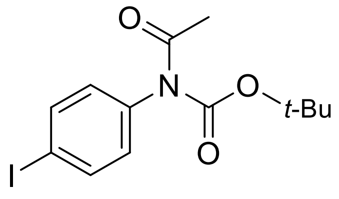

A solution of *N*-(4-iodophenyl)acetamide (500 mg, 1.192 mmol, 1.0 equiv), 4-dimethylaminopyridine (46.8 mg, 0.383 mmol, 0.2 equiv) and triethylamine (666 µL, 4.79 mmol, 2.5 equiv) was stirred at room temperature for 5 min. Di-*tert*-butyl dicarbonate (627 mg, 2.87 mmol, 1.5 equiv) was added subsequently and the resulting mixture was stirred at room temperature for 2.5 d. The reaction mixture was diluted with EtOAc (20 mL) and 1M aq. HCl (5 mL). The organic layer was separated and the aqueous layer was extracted with EtOAc (2×5 mL). The combined organic extracts were dried over Na_2SO_4, filtered, and concentrated *in vacuo* to give the crude product. Purification by silica gel flash chromatography afforded the target product (523

mg, 1.45 mmol) as a white solid. Analytical data was in accordance with that reported in the literature.[17]
^{1}H NMR (500 MHz, $CDCl_3$) δ 7.77 - 7.69 (m, 2H), 6.88 - 6.80 (m, 2H), 2.59 (s, 3H), 1.40 (s, 9H).
^{13}C{^{1}H} NMR (126 MHz, $CDCl_3$) δ 172.7, 152.3, 138.6, 138.1, 130.2, 93.2, 83.6, 27.8, 26.4.
HRMS (ESI) m/z calculated for $C_{13}H_{16}IO_2$ $[M+Na]^+$ 384.0067, found 384.0076.
MP = 115-118 °C.
FTIR (ATR, cm^{-1}) 1303, 1274, 1256, 1155, 1010, 710.

4.2 NHP Esters

5-methyl-1,3-dioxoisoindolin-2-yl 1-phenylcyclopropane-1-carboxylate (1a)

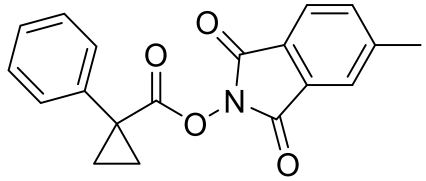

The title product was prepared according to General Procedure A using 1-phenylcyclopropane-1-carboxylic acid (0.81 g, 5.0 mmol, 1.0 equiv), 2-hydroxy-5-methylisoindoline-1,3-dione (0.89 g, 5.0 mmol, 1.0 equiv) *N,N*-dimethylaminopyridine (61 mg, 0.5 mmol, 0.1 equiv), and *N,N*-diisopropylcarbodiimide (0.69 g, 5.5 mmol. 1.1 equiv). Purification of the crude material by FCC (0-100% EtOAc/Hex) using 25 g silica afforded the title product (1.35 g, 4.20 mmol, 84%) as a pale yellow solid.
^{1}H NMR (500 MHz, $CDCl_3$) δ 7.72 (d, *J* = 7.6 Hz, 1H), 7.66 – 7.62 (m, 1H), 7.56 – 7.48 (m, 3H), 7.36 (tt, *J* = 7.3, 1.7 Hz, 2H), 7.31 (tt, *J* = 7.5, 1.4 Hz, 1H), 2.50 (s, 3H), 1.90 (m, 2H), 1.48 (m, 2H).
^{13}C{^{1}H} NMR (126 MHz, $CDCl_3$) δ 171.1, 162.1, 162.0, 146.1, 137.1, 135.1, 130.6, 129.2, 128.5, 127.9, 126.3, 124.4, 123.8, 77.0, 27.3, 22.1, 18.6.
HRMS (ESI) m/z calculated for $C_{19}H_{19}N_2O_4$ $[M+NH_4]+$ 339.1339, found 339.1335.
FTIR (ATR, cm^{-1})
MP = 185-191 °C.

5-methoxy-1,3-dioxoisoindolin-2-yl 1-phenylcyclopropane-1-carboxylate (1b)

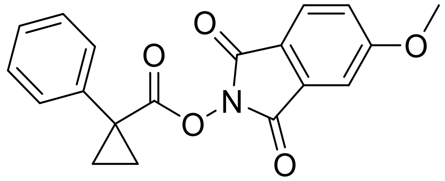

The title product was prepared according to General Procedure A using 1-phenylcyclopropane-1-carboxylic acid (0.81g, 5.0 mmol), 2-hydroxy-5-methoxyisoindoline-1,3-dione (0.97 g, 5.0 mmol) *N,N*-dimethylaminopyridine (61 mg, 0.50 mmol, 0.1 equiv), and *N,N*-diisopropylcarbodiimide (0.69 g, 5.5 mmol. 1.1 equiv). Purification of the crude material by FCC (0-100% EtOAc/Hex) using 25 g silica afforded the title product (1.39 g, 4.11 mmol, 82%) as a white solid.
^{1}H NMR (500 MHz, $CDCl_3$) δ 7.76 (d, *J* = 8.3 Hz, 1H), 7.54 – 7.48 (m, 2H), 7.38 – 7.28 (m, 4H), 7.18 (dd, *J* = 8.4, 2.4 Hz, 1H), 3.92 (s, 3H), 1.93 – 1.87 (m, 2H), 1.50 – 1.46 (m, 2H).
^{13}C{^{1}H} NMR (126 MHz, $CDCl_3$) δ 171.2, 165.1, 162.0, 161.9, 137.1, 131.5, 130.6, 128.5, 127.9, 125.8, 120.7, 120.2, 108.9, 77.0, 56.2, 27.3, 18.6.
HRMS (ESI) m/z calculated for $C_{19}H_{19}N_2O_5$ $[M+NH_4]+$ 355.1289, found 355.1285.

FTIR (ATR, cm^{-1})
MP = 154-157 °C.

4,5,6,7-tetrachloro-1,3-dioxoisoindolin-2-yl 1-phenylcyclopropane-1-carboxylate (1c)

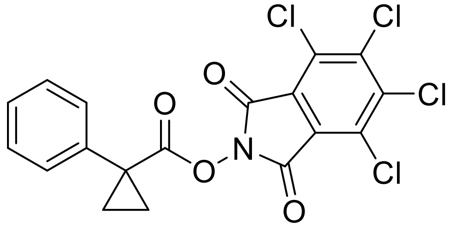

The title product was prepared according to General Procedure A using 1-phenylcyclopropane-1-carboxylic acid (0.81g, 5.0 mmol), *N*-hydroxytetrachlorophthalimide (1.50 g, 5.0 mmol) *N,N*-dimethylaminopyridine (61 mg, 0.50 mmol, 0.1 equiv), and *N,N*-diisopropylcarbodiimide (0.69 g, 5.5 mmol. 1.1 equiv). Purification of the crude material by FCC (0-100% EtOAc/Hex) using 25 g silica afforded the title product (1.78 g, 4.00 mmol, 80%) as an off-white solid, characterization data matched those reported in the literature.[18]
^{1}H NMR (500 MHz, $CDCl_3$) δ 7.51 – 7.48 (m, 2H), 7.37 (ddt, *J* = 8.1, 6.5, 1.1 Hz, 2H), 7.32 (dd, *J* = 7.2, 1.4 Hz, 1H), 1.90 (q, *J* = 4.3 Hz, 2H), 1.54 – 1.48 (m, 2H).
^{13}C{^{1}H} NMR (126 MHz, $CDCl_3$) δ 170.8, 157.6, 141.1, 136.7, 130.7, 130.5, 128.7, 128.3, 124.9, 27.4, 19.0.

1,3-dioxo-1H-benzo[de]isoquinolin-2(3H)-yl 1-phenylcyclopropane-1-carboxylate (1d)

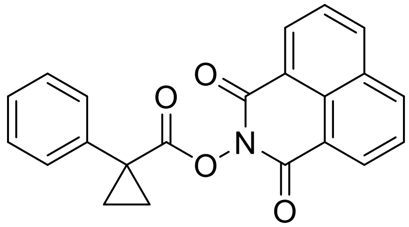

The title product was prepared according to General Procedure A using 1-phenylcyclopropane-1-carboxylic acid (0.81g, 5.0 mmol), *N*-hydroxynaphthalimide (1.06 g, 5.0 mmol) *N,N*-dimethylaminopyridine (61 mg, 0.50 mmol, 0.1 equiv), and *N,N*-diisopropylcarbodiimide (0.69 g, 5.5 mmol. 1.1 equiv). Purification of the crude material by FCC (0-100% EtOAc/Hex) using 25 g silica afforded the title product (1.78 g, 4.00 mmol, 80%) as an off-white solid.
^{1}H NMR (500 MHz, $CDCl_3$) δ 8.58 (dt, *J* = 7.5, 1.4 Hz, 2H), 8.26 – 8.20 (m, 2H), 7.80 – 7.72 (m, 2H), 7.64 – 7.57 (m, 2H), 7.38 (tt, *J* = 7.2, 1.8 Hz, 2H), 7.31 (dd, *J* = 7.2, 1.2 Hz, 1H), 1.98 (q, *J* = 4.3 Hz, 2H), 1.50 (q, *J* = 4.3 Hz, 2H).
^{13}C{^{1}H} NMR (126 MHz, $CDCl_3$) δ 171.1, 159.6, 137.7, 135.1, 132.0, 132.0, 130.8, 128.5, 127.9, 127.7, 127.2, 122.5, 27.8, 18.4.
HRMS (ESI) m/z calculated for $C_{22}H_{19}N2O_4$ [M+NH4]+ 375.1339, found 375.1337.
MP = 206-209 °C

1,3-dioxoisoindolin-2-yl 1-(thiophen-3-yl)cyclopropane-1-carboxylate (1e)

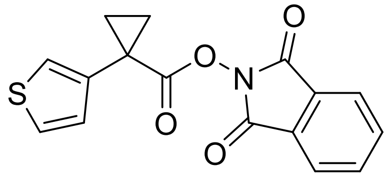

The title product was prepared according to General Procedure A using 1-(thiophen-3-yl)cyclopropane-1-carboxylic acid (0.260 g, 1.5 mmol, 1.0 equiv), *N*-hydroxyphthalimide (0.251 g, 1.5 mmol, 1.0 equiv), *N,N*-dimethylaminopyridine (0.019 g, 0.15 mmol, 0.1 equiv), and *N,N*-diisopropylcarbodiimide (0.214 g, 0.263 mL, 1.7 mmol, 1.1 equiv). Recrystallization from hot methanol afforded the product as a white solid (0.383 g, 79% yield).
^{1}H NMR (500 MHz, $CDCl_3$) δ 7.87 (m, 2H), 7.81 – 7.73 (m, 2H), 7.32 – 7.28 (m, 2H), 7.27 – 7.23 (m, 1H), 1.90 (m, 2H), 1.51 – 1.46 (m, 2H).
^{13}C{^{1}H} NMR (126 MHz, $CDCl_3$) δ 170.5, 161.9, 137.7, 134.7, 129.1, 129.0, 125.7, 124.1, 123.9, 22.4, 19.4.
HRMS (ESI) m/z calculated for $C_{16}H_{11}NNaO_4S$ [M+Na]+ 336.0301, found 336.0297.
MP = 169–171 °C.

1,3-dioxoisoindolin-2-yl 1-(2,6-dimethoxypyridin-3-yl)cyclopropane-1-carboxylate (1f)

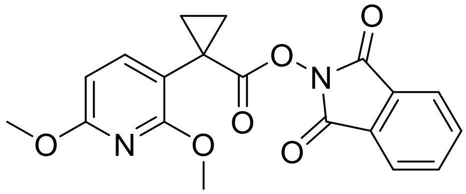

The title product was prepared according to General Procedure A using 1-(2,6-dimethoxypyridin-3-yl)cyclopropane-1-carboxylic acid (0.218 g, 1.0 mmol, 1.0 equiv), *N*-hydroxyphthalimide (0.159 g, 1.0 mmol, 1.0 equiv), *N,N*-dimethylaminopyridine (0.012 g, 0.1 mmol, 0.1 equiv), and *N,N*-diisopropylcarbodiimide (0.136 g, 0.167 mL, 1.1 mmol, 1.1 equiv). Recrystallization from hot methanol afforded the product as a white solid (0.206 g, 57% yield).
^{1}H NMR (500 MHz, $CDCl_3$) δ 7.84 (m, 2H), 7.75 (m, 2H), 7.49 (d, J = 8.0 Hz, 1H), 6.27 (d, J = 8.0 Hz, 1H), 4.07 (s, 3H), 3.92 (s, 3H), 1.85 (m, 2H), 1.33 (m, 2H).
^{13}C{^{1}H} NMR (126 MHz, $CDCl_3$) δ 171.0, 162.9, 162.2, 161.9, 141.5, 134.6, 129.1, 123.8, 110.7, 100.2, 53.7, 53.6, 22.1, 18.5.
HRMS (ESI) m/z calculated for $C_{19}H_{17}N_2O_6$ [M+H]+ 369.1081, found 369.1075.
MP = 130–131 °C.

1,3-dioxoisoindolin-2-yl 1-(benzo[b]thiophen-5-yl)cyclopropane-1-carboxylate (1g)

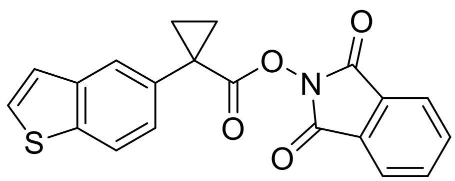

The title product was prepared according to General Procedure A using 1-(benzo[b]thiophen-5-yl)cyclopropane-1-carboxylic acid (0.600 g, 2.75 mmol, 1.0 equiv), *N*-hydroxyphthalimide (0.450

g, 2.75 mmol, 1.0 equiv), *N,N*-dimethylaminopyridine (0.0346 g, 0.28 mmol, 0.1 equiv), and *N,N*-diisopropylcarbodiimide (0.382 g, 0.470 mL, 3.03 mmol, 1.1 equiv). Recrystallization from hot methanol afforded the product as a white solid (0.768 g, 77% yield).
^{1}H NMR (500 MHz, $CDCl_3$) δ 7.96 (d, J = 1.7 Hz, 1H), 7.88 (s, 1H), 7.87 – 7.82 (m, 2H), 7.75 (m, 2H), 7.54 (dd, J = 8.4, 1.8 Hz, 1H), 7.46 (d, J = 5.5 Hz, 1H), 7.34 (dd, J = 5.5, 0.8 Hz, 1H), 1.96 (m, 2H), 1.55 (m, 2H).
^{13}C{^{1}H} NMR (126 MHz, $CDCl_3$) δ 171.2, 161.9, 139.7, 139.4, 134.6, 133.2, 129.0, 127.2, 127.1, 125.4, 123.9, 123.8, 122.5, 27.3, 19.0.
HRMS (ESI) m/z calculated for $C_{20}H_{13}NNaO_4S$ [M+Na]+ 386.0458, found 386.0452.
MP = 146–148 °C.

1,3-dioxoisoindolin-2-yl 1-methylcyclopropane-1-carboxylate (1h)

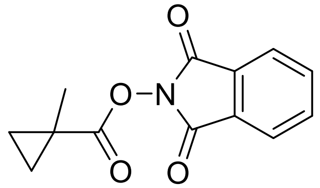

The title product was prepared according to General Procedure A using 1-phenylcyclopropane-1-carboxylic acid (0.50 g, 5.0 mmol, 1.0 equiv), *N*-hydroxyphthalimide (0.89 g, 5.0 mmol, 1.0 equiv) *N,N*-dimethylaminopyridine (61 mg, 0.5 mmol, 0.1 equiv), and *N,N*-diisopropylcarbodiimide (0.69 g, 5.5 mmol. 1.1 equiv). Purification of the crude material by FCC (0-100% EtOAc/Hex) using 12 g silica afforded the title product (0.97 g, 3.95 mmol, 79%) as a white powder.
^{1}H NMR (500 MHz, $CDCl_3$) δ 7.93 – 7.84 (m, 2H), 7.81 – 7.74 (m, 2H), 1.60 – 1.54 (m, 2H), 1.48 (s, 3H), 1.00 – 0.94 (m, 2H).
^{13}C{^{1}H} NMR (126 MHz, $CDCl_3$) δ 172.2, 162.2, 134.8, 129.1, 124.0, 77.2, 19.0, 18.7, 17.5.
HRMS (ESI) m/z calculated for $C_{13}H_{12}NO_4$[M+H]+ 246.0761, found 246.0758.
MP = 125-126 °C.

1,3-dioxoisoindolin-2-yl 1-(3,5,5,8,8-pentamethyl-5,6,7,8-tetrahydronaphthalen-2-yl)cyclopropane-1-carboxylate (1i)

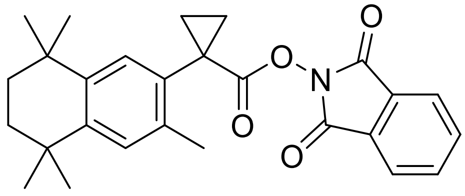

The title product was prepared according to General Procedure A using 1-(3,5,5,8,8-pentamethyl-5,6,7,8-tetrahydronaphthalen-2-yl)cyclopropane-1-carboxylic acid (0.411 g, 1.5 mmol, 1.0 equiv), *N*-hydroxyphthalimide (0.250 g, 1.5 mmol, 1.0 equiv), *N,N*-dimethylaminopyridine (0.019 g, 0.1 mmol, 0.1 equiv), and *N,N*-diisopropylcarbodiimide (0.212 g, 0.260 mL, 1.7 mmol, 1.1 equiv). Recrystallization from hot methanol afforded the product as a white solid (0.382 g, 58% yield).
^{1}H NMR (500 MHz, $CDCl_3$) δ 7.84 (m, 2H), 7.75 (m, 2H), 7.26 (s, 1H), 7.10 (s, 1H), 2.47 (s, 1H), 1.93 (m, 2H), 1.66 (s, 4H), 1.43 (m, 2H), 1.28 (s, 6H), 1.26 (s, 6H.
^{13}C{^{1}H} NMR (126 MHz, $CDCl_3$) δ 171.3, 162.0, 144.5, 142.2, 136.3, 134.6, 132.4, 129.1, 128.6, 128.2, 123.8, 35.2, 35.1, 34.0, 34.0, 31.9, 31.8, 25.8, 19.6, 19.1.
HRMS (ESI) m/z calculated for $C_{27}H_{33}N_2O_4$ [M+NH4]+ 449.2435, found 449.2433.

MP = 201–203 °C.

5-methoxy-1,3-dioxoisoindolin-2-yl bicyclo[1.1.1]pentane-1-carboxylate (1j)

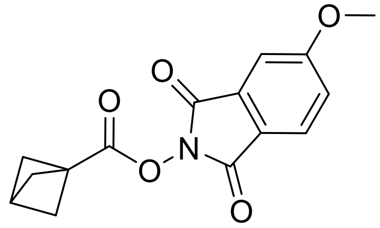

The title product was prepared according to General Procedure A using bicyclo[1.1.1]pentane-1-carboxylic acid (4.46 mmol), 2-hydroxy-5-methoxyisoindoline-1,3-dione (4.46 mmol) *N,N*-dimethylaminopyridine (55 mg, 0.446 mmol, 0.1 equiv), and *N,N*-diisopropylcarbodiimide (0.768 μL, 4.91 mmol, 1.1 equiv). Purification of the crude material by FCC (0-100% EtOAc/Hex) using 12 g silica afforded the title product (1.15 g, 4.00 mmol, 90%) as a white solid.
^{1}H NMR (600 MHz, $CDCl_3$) δ 7.80 (d, J = 8.3 Hz, 1H), 7.37 (d, J = 2.3 Hz, 1H), 7.22 (dd, J = 2.4, 8.4 Hz, 1H), 3.95 (s, 3H), 2.56 (s, 1H), 2.33 (s, 6H).
^{13}C{^{1}H} NMR (151 MHz, $CDCl_3$) δ 165.1, 164.7, 162.0, 161.8, 131.5, 125.9, 120.7, 120.3, 108.9, 56.2, 52.4, 39.9, 29.1.
HRMS (ESI) m/z calculated for $C_{15}H_{14}NO_5$ [M+H]+ 288.0867, found 288.0864.
FTIR (ATR, cm^{-1}) 2995, 1781, 1740, 1490, 1360, 1289, 1040.
MP = 116-124 °C.

1,3-dioxoisoindolin-2-yl 3-(dimethylcarbamoyl)bicyclo[1.1.1]pentane-1-carboxylate (1k)

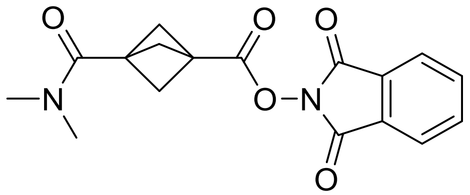

The title product was prepared according to General Procedure B using 3-(dimethylcarbamoyl)bicyclo[1.1.1]pentane-1-carboxylic acid (4.09 mmol). Collection of the precipitate resulting from dilution of the crude reaction mixture with water (~20 mL) afforded the title compound as a white solid (1.02 g, 3.11 mmol, 76% yield).
^{1}H NMR (600 MHz, $CDCl_3$) δ 7.94 - 7.86 (m, 2H), 7.83 - 7.77 (m, 2H), 3.12 (s, 3H), 2.96 (s, 3H), 2.64 (s, 6H).
^{13}C{^{1}H} NMR (151 MHz, $CDCl_3$) δ 167.7, 164.9, 161.7, 134.8, 128.9, 124.0, 54.5, 40.9, 37.2, 36.2, 36.1.
HRMS (ESI) m/z calculated for $C_{17}H_{18}N_2O_5$ [M+H]+ 329.1132, found 329.1134.
FTIR (ATR, cm^{-1}) 2925, 1778, 1739, 1628, 1181, 995, 913, 745.
MP =130-133 °C.

1-methyl 3-(5-methyl-1,3-dioxoisoindolin-2-yl) bicyclo[1.1.1]pentane-1,3- dicarboxylate (1l)

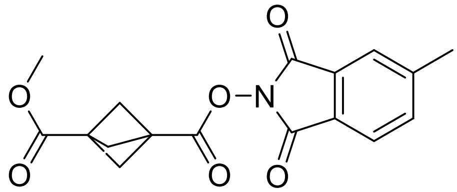

The title product was prepared according to General Procedure A using 3-(methoxycarbonyl)bicyclo[1.1.1]pentane-1-carboxylic acid (0.17 g, 1.0 mmol, 1.0 equiv), 2-hydroxy-5-methylisoindoline-1,3-dione (0.18 g, 1.0 mmol, 1.0 equiv) *N,N*-dimethylaminopyridine (12 mg, 0.1 mmol, 0.1 equiv), and *N,N*-diisopropylcarbodiimide (0.14 g, 1.1 mmol. 1.1 equiv). Purification of the crude material by FCC (0-100% EtOAc/Hex) using 12 g silica afforded the title product (286 mg, 0.87 mmol, 87%) as a white solid.

^{1}H NMR (500 MHz, $CDCl_3$) δ 7.77 (d, *J* = 7.7 Hz, 1H), 7.69 (dt, *J* = 1.5, 0.7 Hz, 1H), 7.58 (ddd, *J* = 7.7, 1.5, 0.8 Hz, 1H), 3.72 (s, 3H), 2.55 (s, 6H), 2.53 (s, 3H).

^{13}C{^{1}H} NMR δ 168.9, 164.8, 162.0, 161.9, 146.4, 135.3, 129.2, 126.2, 124.6, 124.0, 77.0, 53.6, 52.0, 38.6, 35.4, 22.2.

HRMS (ESI) m/z calculated for $C_{17}H_{15}NNaO_6$ [M+Na]+ 352.07916, found 352.0791.

MP =165-168 °C.

1-(5-methoxy-1,3-dioxoisoindolin-2-yl) 3-methyl bicyclo[1.1.1]pentane-1,3-dicarboxylate (1m)

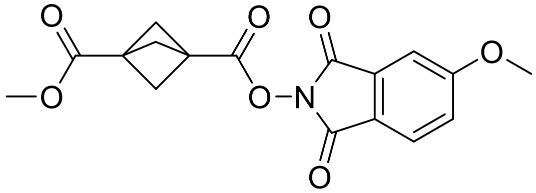

The title product was prepared according to General Procedure A using 3-(methoxycarbonyl)bicyclo[1.1.1]pentane-1-carboxylic acid (0.17 g, 1.0 mmol, 1.0 equiv), 2-hydroxy-5-methoxyisoindoline-1,3-dione (0.19 g, 1.0 mmol, 1.0 equiv) *N,N*-dimethylaminopyridine (12 mg, 0.1 mmol, 0.1 equiv), and *N,N*-diisopropylcarbodiimide (0.14 g, 1.1 mmol. 1.1 equiv). Purification of the crude material by FCC (0-100% EtOAc/Hex) using 12 g silica afforded the title (310 mg, 0.90 mmol, 90%) as a white solid.

^{1}H NMR (600 MHz, $CDCl_3$) δ 7.81 (d, J = 8.4 Hz, 1H), 7.37 (d, J = 2.3 Hz, 1H), 7.23 (dd, J = 2.3, 8.4 Hz, 1H), 3.95 (s, 3H), 3.73 (s, 3H), 2.56 (s, 6H).

^{13}C{^{1}H} NMR (151 MHz, $CDCl_3$) δ 168.9, 165.2, 164.8, 161.8, 161.6, 131.4, 126.0, 120.5, 120.4, 109.0, 56.2, 53.6, 52.0, 38.5, 35.4.

HRMS (ESI) m/z calculated for $C_{17}H_{16}NO_7$ [M+H]+ 346.0922, found 346.0916.

FTIR (ATR, cm^{-1}) 1778, 1738, 1488, 1354, 1238, 1213, 999, 913, 745.

MP = 191-193 °C.

1,3-dioxoisoindolin-2-yl bicyclo[1.1.1]pentane-1-carboxylate (1n)

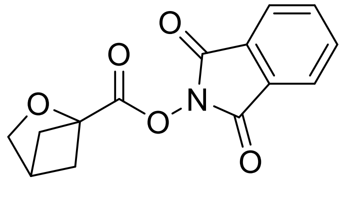

The title product was prepared according to General Procedure B using 2-oxabicyclo[2.1.1]hexane-1-carboxylic acid (2.03 mmol). After dilution of the crude reaction mixture with water (~20 mL), the resulting aqueous mixture was extracted with EtOAc/Hex (1:1, 3× 20 mL). The combined organic extracts were dried over Na_2SO_4, and concentrated in vacuo to give the crude NHP ester which was purified by FCC (0-50% EtOAc/Hex) using 12 g silica to yield the title product (301 mg, 1.10 mmol, 54%) as a white solid.
^{1}H NMR (600 MHz, $CDCl_3$) δ 7.93 - 7.88 (m, 2H), 7.82 - 7.78 (m, 2H), 4.00 - 3.97 (m, 2H), 3.10 (t, J = 3.2 Hz, 1H), 2.43 (ddd, J = 1.9, 3.1, 4.7 Hz, 2H), 2.02 - 1.98 (m, 2H).
^{13}C{^{1}H} NMR (151 MHz, $CDCl_3$) δ 164.3, 161.5, 134.8, 128.9, 124.1, 83.2, 70.0, 43.6, 38.6.
HRMS (ESI) m/z calculated for $C_{14}H_{13}NO_5$ [M+H]+ 274.0710, found 274.0716.
FTIR (ATR, cm^{-1}) 2963, 2894, 1816, 1784, 1738, 1031, 913, 745, 696.
MP = 125-128 °C.

1-(1,3-dioxoisoindolin-2-yl) 4-methyl bicyclo[2.1.1]hexane-1,4-dicarboxylate (1o)

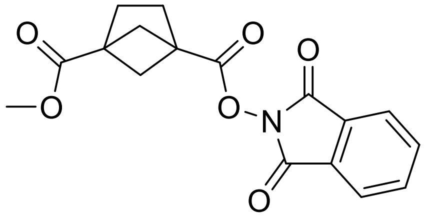

The title product was prepared according to General Procedure B using 4-(methoxycarbonyl)bicyclo[2.1.1]hexane-1-carboxylic acid (2.72 mmol). After dilution of the crude reaction mixture with water (~20 mL), the resulting aqueous mixture was extracted with EtOAc/Hex (1:1, 3× 20 mL). The combined organic extracts were dried over Na_2SO_4, and concentrated in vacuo to give the crude NHP ester which was purified by FCC (0-50% EtOAc/Hex) using 12 g silica to yield the title product (717 mg, 2.18 mmol, 80%) as a white solid.
^{1}H NMR (600 MHz, $CDCl_3$) δ 7.93 - 7.85 (m, 2H), 7.84 - 7.74 (m, 2H), 3.73 (s, 3H), 2.45 - 2.39 (m, 2H), 2.28 - 2.21 (m, 2H), 2.13 - 2.09 (m, 2H), 1.94 - 1.90 (m, 2H).
^{13}C{^{1}H} NMR (151 MHz, $CDCl_3$) δ 172.2, 168.2, 161.8, 134.7, 128.9, 124.0, 51.8, 49.7, 46.5, 44.9, 30.0, 29.5.
HRMS (ESI) m/z calculated for $C_{17}H_{16}NO_6$ [M+H]+ 330.0973, found 330.0972.
FTIR (ATR, cm^{-1}) 2950, 1779, 1732, 1263, 1141, 987, 693.
MP = 104-106 °C.

1-(tert-butyl) 3-(1,3-dioxoisoindolin-2-yl) 3-methylazetidine-1,3-dicarboxylate (1p)

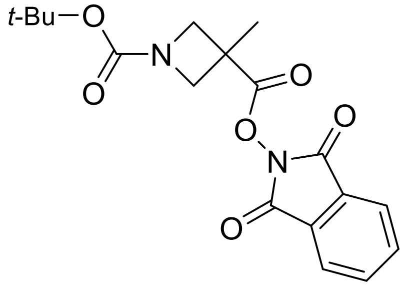

The title product was prepared according to General Procedure A using 1-(tert-butoxycarbonyl)-3-methylazetidine-3-carboxylic acid (0.500 g , 2.32 mmol), *N*-hydroxyphthalimide (0.379 g, 2.32 mmol, 1.0 equiv), *N,N*-dimethylaminopyridine (28 mg, 0.232 mmol, 0.1 equiv), and *N,N*-

diisopropylcarbodiimide (0.363 mL, 5.5 mmol. 1.0 equiv). Purification of the crude material by FCC (0-50% EtOAc/Hex) using 12 g silica afforded the title product (708 mg, 1.97 mmol, 85%) as a white solid.
^{1}H NMR (500 MHz, $CDCl_3$) δ 7.93 - 7.88 (m, 2H), 7.85 - 7.78 (m, 2H), 4.48 (m, 2H), 3.85 (m, 2H), 1.77 (s, 3H), 1.47 (s, 9H).
^{13}C{^{1}H} NMR (151 MHz, $CDCl_3$) δ 170.7, 161.7, 155.9, 134.8, 128.8, 124.0, 80.1, 58.1, 37.4, 28.3, 22.2.
HRMS (ESI) m/z calculated for $C_{18}H_{20}N_2O_6Na$ [M+Na]+ 383.1214, found 383.1216.
FTIR (ATR, cm^{-1}) 1746, 1698, 1684, 1052, 912, 743.
MP = 126-128 °C.

1-(tert-butyl) 3-(5-methyl-1,3-dioxoisoindolin-2-yl) 3-methylazetidine-1,3-dicarboxylate (1q)

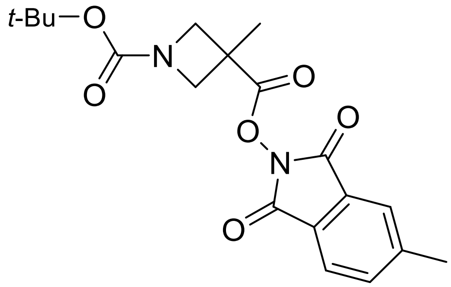

The title product was prepared according to General Procedure A using 1-(tert-butoxycarbonyl)-3-methylazetidine-3-carboxylic acid (1.08 g, 5.0 mmol), 2-hydroxy-5-methylisoindoline-1,3-dione (0.815 g, 5.0 mmol, 1.0 equiv), *N,N*-dimethylaminopyridine (60 mg, 0.50 mmol, 0.1 equiv), and *N,N*-diisopropylcarbodiimide (0.861 mL, 5.5 mmol. 1.0 equiv). Purification of the crude material by FCC (0-50% EtOAc/Hex) using 25 g silica afforded the title product (1.49 g, 4.0 mmol, 80%%) as a white solid.
^{1}H NMR (500 MHz, $CDCl_3$) δ 7.78 (d, *J* = 7.6 Hz, 1H), 7.72 – 7.68 (m, 1H), 7.62 – 7.56 (m, 1H), 4.47 (d, *J* = 8.7 Hz, 2H), 3.84 (d, *J* = 8.7 Hz, 2H), 2.54 (s, 3H), 1.76 (s, 3H), 1.46 (s, 9H).
^{13}C{^{1}H} NMR (126 MHz, $CDCl_3$) δ 170.8, 162.0, 161.9, 156.0, 146.4, 135.4, 129.2, 126.2, 124.6, 124.0, 80.2, 37.5, 28.3, 22.3, 22.2.
HRMS (ESI) m/z calculated for $C_{19}H_{22}N_2O_6Na$ [M+Na]+ 397.130, found 397.1361
MP = 97-99 °C.

1-(tert-butyl) 3-(5-methoxy-1,3-dioxoisoindolin-2-yl) 3-methylazetidine-1,3-dicarboxylate (1r)

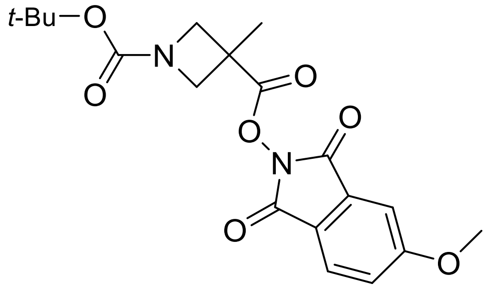

The title product was prepared according to General Procedure A using 1-(tert-butoxycarbonyl)-3-methylazetidine-3-carboxylic acid (1.08 g, 5.0 mmol), 2-hydroxy-5-methoxyisoindoline-1,3-dione (0.965 g, 5.0 mmol, 1.0 equiv), *N,N*-dimethylaminopyridine (60 mg, 0.5 mmol, 0.1 equiv),

and *N,N*-diisopropylcarbodiimide (0.861 mL, 5.5 mmol. 1.0 equiv). Purification of the crude material by FCC (0-50% EtOAc/Hex) using 25 g silica afforded the title product (1.65 g, 4.25 mmol, 85%) as a white solid.
^{1}H NMR (500 MHz, $CDCl_3$) δ 7.81 (d, *J* = 8.4 Hz, 1H), 7.37 (d, *J* = 2.3 Hz, 1H), 7.23 (dd, *J* = 8.4, 2.3 Hz, 1H), 4.47 (d, *J* = 8.7 Hz, 2H), 3.95 (s, 3H), 3.83 (d, *J* = 8.8 Hz, 2H), 1.76 (s, 3H), 1.46 (s, 9H).
^{13}C{^{1}H} NMR (126 MHz, $CDCl_3$) δ 170.9, 165.3, 161.9, 161.7, 156.0, 131.5, 126.0, 120.6, 120.5, 109.1, 80.2, 56.2, 37.5, 28.4, 22.3.
HRMS (ESI) m/z calculated for $C_{19}H_{22}N_2O_7Na$ [M+Na]+ 413.1319, found 413.1307
MP = 90-92 °C.

1-(1,3-dioxoisoindolin-2-yl) 1-ethyl cyclobutane-1,1-dicarboxylate (1s)

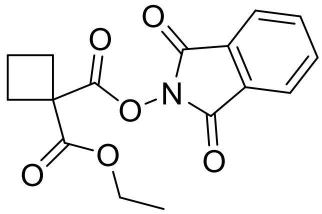

The title product was prepared according to General Procedure A using 1-(ethoxycarbonyl)cyclobutane-1-carboxylic acid (0.86 g, 5 mmol, 1.0 equiv), *N*-hydroxyphthalimide (0.82 g, 5.0 mmol, 1.0 equiv), *N,N*-dimethylaminopyridine (61 mg, 0.5 mmol, 0.1 equiv), and *N,N*-diisopropylcarbodiimide (0.69 g, 5.5 mmol. 1.1 equiv). Recrystallization from hot methanol afforded the product as a white solid (1.12g, 71% yield)
^{1}H NMR (500 MHz, $CDCl_3$) δ 7.89 (m, 2H), 7.80 (m, 2H), 4.32 (q, *J* = 7.2 Hz, 2H), 2.86 – 2.76 (m, 2H), 2.76 – 2.67 (m, 2H), 2.13 (ddqd, *J* = 13.6, 9.0, 4.4, 2.0 Hz, 2H), 1.37 (t, *J* = 7.1 Hz, 3H).
^{13}C{^{1}H} NMR (126 MHz, $CDCl_3$) δ 169.9, 168.4, 161.7, 134.8, 129.0, 124.0, 62.3, 53.5, 51.0, 29.1, 16.5, 13.9.
HRMS (ESI) m/z calculated for $C_{16}H_{15}NO_6Na$ [M+Na]+ 340.07916, found 340.0784
MP =165-168 °C.

5-methyl-1,3-dioxoisoindolin-2-yl 3-methyloxetane-3-carboxylate (1t)

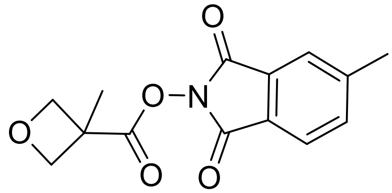

The title product was prepared according to General Procedure A using 3-methyloxetane-3-carboxylic acid (0.58 g, 5.0 mmol), 2-hydroxy-5-methylisoindoline-1,3-dione (0.815 g, 5.0 mmol, 1.0 equiv), *N,N*-dimethylaminopyridine (60 mg, 0.50 mmol, 0.1 equiv), and *N,N*-diisopropylcarbodiimide (0.861 mL, 5.5 mmol. 1.0 equiv). Purification of the crude material by FCC (0-50% EtOAc/Hex) using 25 g silica afforded the title product (1.49 g, 4.0 mmol, 80%%) as a white solid.
^{1}H NMR (500 MHz, $CDCl_3$) δ 7.71 (d, *J* = 7.6 Hz, 1H), 7.64 (s, 1H), 7.52 (d, *J* = 7.7 Hz, 1H), 5.10 (d, *J* = 6.2 Hz, 2H), 4.47 (d, *J* = 6.2 Hz, 2H), 2.47 (s, 3H), 1.77 (s, 3H).
^{13}C{^{1}H} NMR (126 MHz, $CDCl_3$) δ 170.5, 162.1, 162.0, 146.5, 135.4, 129.2, 126.2, 124.6, 124.0, 79.1, 43.3, 22.2, 21.4.
HRMS (ESI) m/z calculated for $C_{14}H_{14}NO_5$[M+H]+ 276.0867, found 276.0863

MP = 94-96 °C.

5-methoxy-1,3-dioxoisoindolin-2-yl 3-methyloxetane-3-carboxylate (1u)

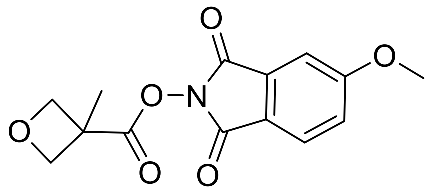

The title product was prepared according to General Procedure A using 3-methyloxetane-3-carboxylic acid (0.58 g, 5.0 mmol), 2-hydroxy-5-methoxyisoindoline-1,3-dione (0.965 g, 5.0 mmol, 1.0 equiv), *N,N*-dimethylaminopyridine (60 mg, 0.50 mmol, 0.1 equiv), and *N,N*-diisopropylcarbodiimide (0.861 mL, 5.5 mmol. 1.0 equiv). Purification of the crude material by FCC (0-50% EtOAc/Hex) using 25 g silica afforded the title product (1.49 g, 4.0 mmol, 80%%) as a white solid.
^{1}H NMR (500 MHz, $CDCl_3$) δ 7.81 (d, *J* = 8.3 Hz, 1H), 7.38 (d, *J* = 2.3 Hz, 1H), 7.23 (dd, *J* = 8.4, 2.3 Hz, 1H), 5.17 (d, *J* = 6.2 Hz, 2H), 4.54 (d, *J* = 6.3 Hz, 2H), 3.95 (s, 3H), 1.84 (s, 3H).
^{13}C{^{1}H} NMR (126 MHz, $CDCl_3$) δ 170.6, 165.3, 161.9, 161.8, 131.5, 126.0, 120.6, 120.5, 109.1, 79.1, 56.2, 43.3, 21.5.
HRMS (ESI) m/z calculated for $C_{14}H_{14}NO_6$ [M+H]+ 292.0816, found 292.0811
MP = 89-92 °C.

4.3 Products

1-methoxy-4-(1-phenylcyclopropyl)benzene (3a)

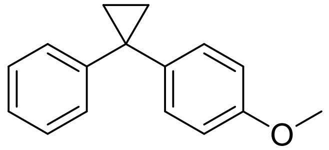

The title product was prepared according to General Procedure C at a 0.50 mmol scale using $NiBr_2$(dme) (10.8 mg, 0.035 mmol, 0.07 equiv), $^{t\text{-Bu}}$BpyCam^{CN} (11.8 mg, 0.035 mmol, 0.07 equiv), zinc flake (64.6 mg, 1.0 mmol, 2.0 equiv), and DMA (0.64 mL) employing 1,3-dioxoisoindolin-2-yl 1-phenylcyclopropane-1-carboxylate (154 mg, 0.50 mmol, 1.00 equiv) and 4-iodoanisole (116 mg, 0.50 mmol, 1.00 equiv) at r.t. (20-22 °C). Purification of the crude reaction mixture using Purification Method A afforded the title product as a colorless oil, 86 mg (77% yield). Characterization data matched those reported in the literature.[18]

The title product was prepared according to General Procedure C at a 0.50 mmol scale using $NiBr_2$(dme) (10.8 mg, 0.035 mmol, 0.07 equiv), $^{t\text{-Bu}}$BpyCam^{CN} (11.8 mg, 0.035 mmol, 0.07 equiv), zinc flake (64.6 mg, 1.0 mmol, 2.0 equiv), and THF (0.64 mL) employing 1,3-dioxoisoindolin-2-yl 1-phenylcyclopropane-1-carboxylate (154 mg, 0.5 mmol, 1.0 equiv) and 4-bromoanisole (91 mg, 0.5 mmol, 1.0 equiv) at 40 °C. Purification of the crude reaction mixture using Purification Method A afforded the title product as a colorless oil, 5 mg (5% yield). Modification of this procedure using 5-methyl-1,3-dioxoisoindolin-2-yl 1-phenylcyclopropane-1-carboxylate (161 mg, 0.5 mmol, 1.0 equiv) or 5-methoxy-1,3-dioxoisoindolin-2-yl 1-phenylcyclopropane-1-carboxylate (169 mg, 0.5 mmol, 1.0 equiv), 4,5,6,7-tetrachloro-1,3-dioxoisoindolin-2-yl 1-

phenylcyclopropane-1-carboxylate (222 mg, 0.5 mmol, 1.0 equiv), or 1,3-dioxo-1H-benzo[de]isoquinolin-2(3H)-yl 1-phenylcyclopropane-1-carboxylate (178 mg, 0.5 mmol, 1.0 equiv) afforded the title product, 20 mg (18% yield), 43 mg (38% yield), 12 mg (11% yield), and 0 mg (0%) respectively.

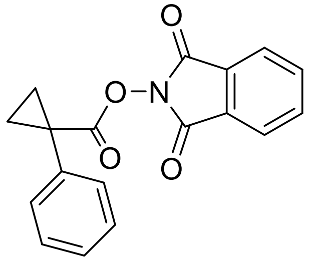

(154 mg, 0.50 mmol)

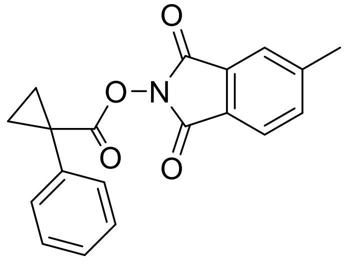

(161 mg, 0.50 mmol)

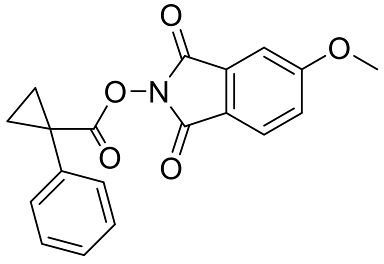

(169 mg, 0.50 mmol)

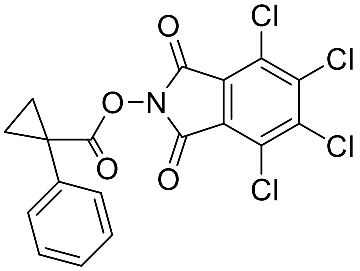

(222 mg, 0.50 mmol)

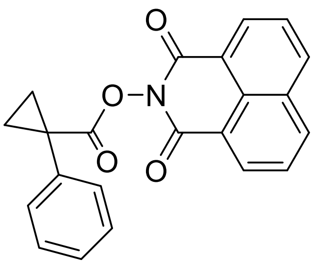

(178 mg, 0.50 mmol)

^{1}H NMR (500 MHz, $CDCl_3$) δ 7.27 – 7.10 (m, 7H), 6.84 – 6.78 (m, 2H), 3.75 (s, 3H), 1.25 (m, 4H).
^{13}C{^{1}H} NMR (126 MHz, $CDCl_3$) δ 157.9, 146.3, 137.8, 129.8, 128.2, 127.9, 125.7, 113.7, 55.2, 29.2, 16.3.
HRMS (ESI) m/z calculated for $C_{16}H_{17}O$ [M+H]+ 225.1274, found 225.1273

N,N-dimethyl-4-(1-phenylcyclopropyl)aniline (3b)

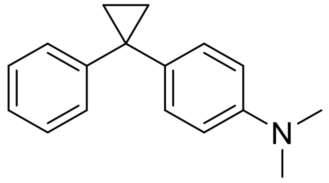

The title product was prepared according to General Procedure C at a 0.50 mmol scale using $NiBr_2$(dme) (10.8 mg, 0.035 mmol, 0.07 equiv), $^{t\text{-Bu}}$BpyCam^{CN} (11.8 mg, 0.035 mmol, 0.07 equiv), zinc flake (64.6 mg, 1.0 mmol, 2.0 equiv), and THF (0.64 mL) employing 1,3-dioxoisoindolin-2-yl 1-phenylcyclopropane-1-carboxylate (154 mg, 0.5 mmol, 1.0 equiv) and 4-bromo-N,N-dimethylaniline (100 mg, 0.5 mmol, 1.0 equiv) at 40 °C. Purification of the crude reaction mixture using Purification Method A afforded the title product as a colorless oil, 20 mg (16% yield). Modification of this procedure using 5-methyl-1,3-dioxoisoindolin-2-yl 1-phenylcyclopropane-1-carboxylate (161 mg, 0.5 mmol, 1.0 equiv) or 5-methoxy-1,3-dioxoisoindolin-2-yl 1-phenylcyclopropane-1-carboxylate (169 mg, 0.5 mmol, 1.0 equiv), 4,5,6,7-tetrachloro-1,3-dioxoisoindolin-2-yl 1-phenylcyclopropane-1-carboxylate (222 mg, 0.5 mmol, 1.0 equiv), or 1,3-

dioxo-1H-benzo[de]isoquinolin-2(3H)-yl 1-phenylcyclopropane-1-carboxylate (178 mg, 0.5 mmol, 1.0 equiv) afforded the title product, 26 mg (22% yield), 32 mg (27% yield), 0 mg (0% yield), and 7 mg (6% yield) respectively.

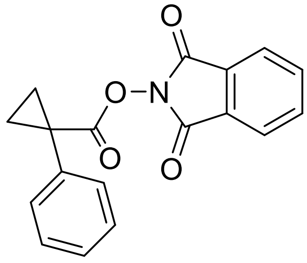

(154 mg, 0.50 mmol)

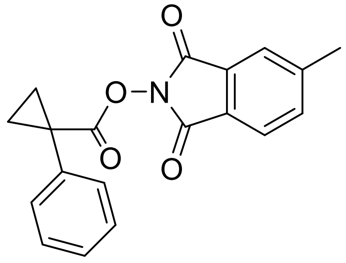

(161 mg, 0.50 mmol)

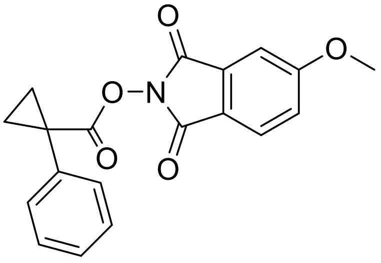

(169 mg, 0.50 mmol)

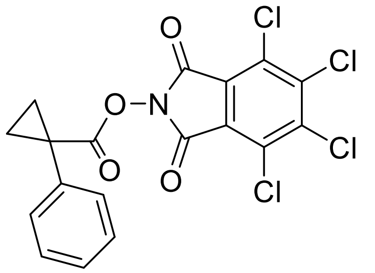

(222 mg, 0.50 mmol)

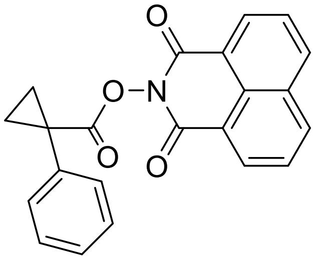

(178 mg, 0.50 mmol)

^{1}H NMR (500 MHz, $CDCl_3$) δ 7.26 – 7.08 (m, 6H), 6.72 – 6.64 (m, 2H), 2.90 (s, 6H), 1.23 (m, 4H).
^{13}C{^{1}H} NMR (126 MHz, $CDCl_3$) δ 149.1, 146.8, 133.6, 129.6, 128.1, 127.8, 125.5, 112.7, 40.8, 29.0, 16.3.
HRMS (ESI)) m/z calculated for $C_{17}H_{20}N$ [M+H]+ 238.15903, found 238.1588.

cyclopropyl(4-(1-phenylcyclopropyl)phenyl)methanone (3c)

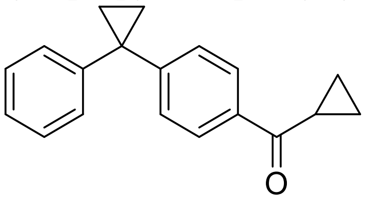

The title product was prepared according to General Procedure C at a 0.50 mmol scale using $NiBr_2$(dme) (10.8 mg, 0.035 mmol, 0.07 equiv), $^{t\text{-Bu}}$BpyCam^{CN} (11.8 mg, 0.035 mmol, 0.07 equiv), zinc flake (64.6 mg, 1.0 mmol, 2.0 equiv), and THF (0.64 mL) employing 1,3-dioxoisoindolin-2-yl 1-phenylcyclopropane-1-carboxylate (154 mg, 0.5 mmol, 1.0 equiv) and (4-bromophenyl)(cyclopropyl)methanone (112 mg, 0.5 mmol, 1.0 equiv) at 40 °C. Purification of the crude reaction mixture using Purification Method A afforded the title product as a colorless oil, 42 mg (isolated with 5% aryl dimer) (30% yield). Modification of this procedure using 5-methyl-1,3-dioxoisoindolin-2-yl 1-phenylcyclopropane-1-carboxylate (161 mg, 0.5 mmol, 1.0 equiv) or 5-methoxy-1,3-dioxoisoindolin-2-yl 1-phenylcyclopropane-1-carboxylate (169 mg, 0.5 mmol, 1.0 equiv), 4,5,6,7-tetrachloro-1,3-dioxoisoindolin-2-yl 1-phenylcyclopropane-1-carboxylate (222 mg, 0.5 mmol, 1.0 equiv), or 1,3-dioxo-1H-benzo[de]isoquinolin-2(3H)-yl 1-phenylcyclopropane-1-carboxylate (178 mg, 0.5 mmol, 1.0 equiv) afforded the title product, 65 mg (50% yield) and 78 mg (60% yield), 0 mg (0% yield), and 32 mg (24% yield) respectively.

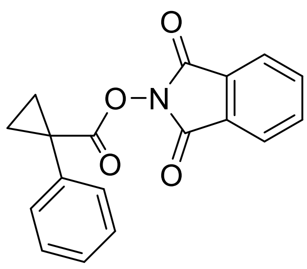

(154 mg, 0.50 mmol)

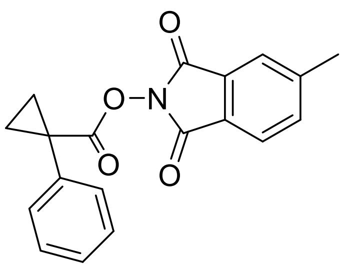

(161 mg, 0.50 mmol)

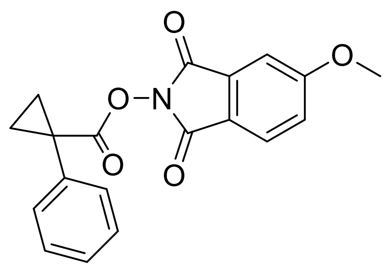

(169 mg, 0.50 mmol)

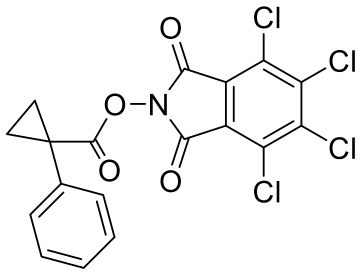

(222 mg, 0.50 mmol)

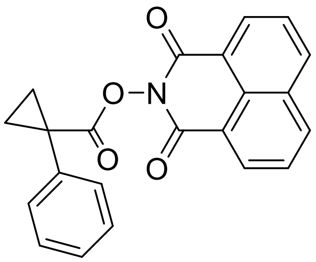

(178 mg, 0.50 mmol)

^{1}H NMR (400 MHz, $CDCl_3$) δ 7.95 – 7.87 (m, 2H), 7.34 – 7.17 (m, 7H), 2.63 (tt, J = 7.8, 4.6 Hz, 1H), 1.41 – 1.36 (m, 2H), 1.37 – 1.31 (m, 2H), 1.20 (dt, J = 4.6, 3.3 Hz, 2H), 1.00 (dq, J = 7.2, 3.6 Hz, 2H).
^{13}C{^{1}H} NMR (101 MHz, $CDCl_3$) δ 200.1, 151.3, 144.6, 135.7, 128.9, 128.5, 128.1, 127.9, 126.4, 77.1, 30.0, 17.1, 17.0, 11.5.
HRMS (ESI)) m/z calculated for $C_{19}H_{19}O$ [M+H]+ 263.1430, found 263.1428.

4-(1-phenylcyclopropyl)benzonitrile (3d)

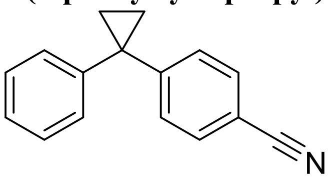

The title product was prepared according to General Procedure C at a 0.50 mmol scale using $NiBr_2$(dme) (10.8 mg, 0.035 mmol, 0.07 equiv), $^{t\text{-Bu}}$BpyCam^{CN} (11.8 mg, 0.035 mmol, 0.07 equiv), zinc flake (64.6 mg, 1.0 mmol, 2.0 equiv), and DMA (0.64 mL) employing 1,3-dioxoisoindolin-2-yl 1-phenylcyclopropane-1-carboxylate (154 mg, 0.5 mmol, 1.0 equiv) and 4-iodobenzonitrile (115 mg, 0.5 mmol, 1.0 equiv) at r.t. (20-22 °C). Purification of the crude reaction mixture using Purification Method A afforded the title product as a colorless oil, 69 mg (64% yield).

The title product was prepared according to General Procedure C at a 0.50 mmol scale using $NiBr_2$(dme) (10.8 mg, 0.035 mmol, 0.07 equiv), $^{t\text{-Bu}}$BpyCam^{CN} (11.8 mg, 0.035 mmol, 0.07 equiv), zinc flake (64.6 mg, 1.0 mmol, 2.0 equiv), and THF (0.64 mL) employing 1,3-dioxoisoindolin-2-yl 1-phenylcyclopropane-1-carboxylate (154 mg, 0.5 mmol, 1.0 equiv) and 4-bromobenzonitrile (91 mg, 0.5 mmol, 1.0 equiv) at 40 °C. Purification of the crude reaction mixture using Purification Method A afforded the title product as a colorless oil, 51 mg (47% yield). Modification of this procedure using 5-methyl-1,3-dioxoisoindolin-2-yl 1-phenylcyclopropane-1-carboxylate (161 mg, 0.5 mmol, 1.0 equiv) or 5-methoxy-1,3-dioxoisoindolin-2-yl 1-phenylcyclopropane-1-carboxylate (169 mg, 0.5 mmol, 1.0 equiv), 4,5,6,7-tetrachloro-1,3-dioxoisoindolin-2-yl 1-

phenylcyclopropane-1-carboxylate (222 mg, 0.5 mmol, 1.0 equiv), or 1,3-dioxo-1H-benzo[de]isoquinolin-2(3H)-yl 1-phenylcyclopropane-1-carboxylate (178 mg, 0.5 mmol, 1.0 equiv) afforded the title product, 68 mg (62% yield) 58 mg (53% yield), 0 mg (0% yield), and 21 mg (20% yield) respectively.

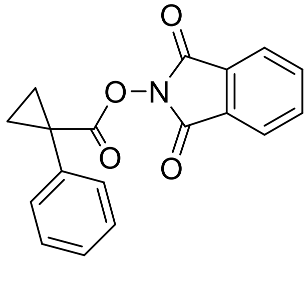

(154 mg, 0.50 mmol)

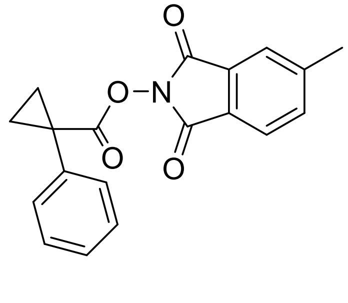

(161 mg, 0.50 mmol)

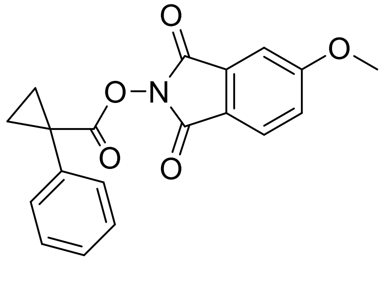

(169 mg, 0.50 mmol)

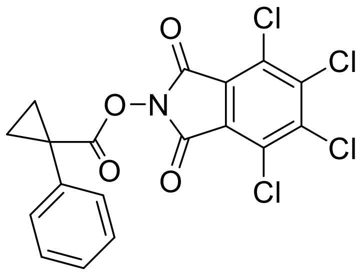

(222 mg, 0.50 mmol)

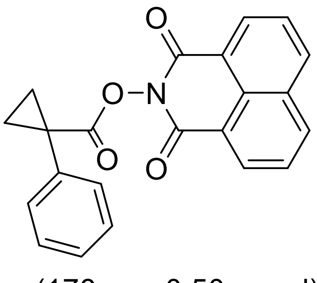

(178 mg, 0.50 mmol)

^{1}H NMR (400 MHz, $CDCl_3$) δ 7.56 – 7.50 (m, 2H), 7.38 – 7.28 (m, 2H), 7.26 – 7.18 (m, 5H), 1.44 – 1.38 (m, 2H), 1.37 – 1.28 (m, 2H).
^{13}C{^{1}H} NMR (101 MHz, $CDCl_3$) δ 151.7, 143.8, 132.2, 132.1, 129.1, 128.6, 128.2, 126.8, 119.1, 109.4, 30.1, 17.3.
HRMS (ESI)) m/z calculated for $C_{16}H_{14}N$ [M+H]+ 220.1121, found 220.1118.

1-(tert-butyl)-4-(1-phenylcyclopropyl)benzene (3e)

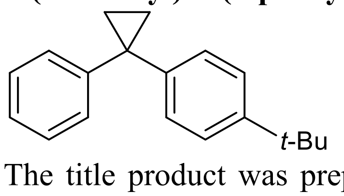

The title product was prepared according to General Procedure C at a 0.50 mmol scale using $NiBr_2$(dme) (10.8 mg, 0.035 mmol, 0.07 equiv), $^{t\text{-Bu}}$BpyCam^{CN} (11.8 mg, 0.035 mmol, 0.07 equiv), zinc flake (64.6 mg, 1.0 mmol, 2.0 equiv), and DMA (0.64 mL) employing 1,3-dioxoisoindolin-2-yl 1-phenylcyclopropane-1-carboxylate (154 mg, 0.5 mmol, 1.0 equiv) and 2-(4-iodophenyl)-4,4,5,5-tetramethyl-1,3,2-dioxaborolane (165 mg, 0.5 mmol, 1.0 equiv) at r.t. (20-22 °C). Purification of the crude reaction mixture using Purification Method A afforded the title product as a colorless oil, 90 mg (72% yield)
^{1}H NMR (500 MHz, $CDCl_3$) δ 7.29 – 7.28 (m, 1H), 7.27 – 7.23 (m, 5H), 7.17 (ddd, J = 6.0, 5.0, 2.7 Hz, 1H), 7.15 – 7.13 (m, 1H), 7.13 – 7.11 (m, 1H), 1.29 (s, 9H), 1.28 – 1.27 (m, 4H).

$^{13}C\{^1H\}$ NMR (126 MHz, $CDCl_3$) δ 148.6, 145.9, 142.7, 128.6, 128.2, 127.8, 125.9, 125.1, 34.3, 31.4, 29.4, 16.4.
HRMS (ESI) m/z calculated for $C_{19}H_{23}$ [M+H]+ 251.1794, found 251.1792.

4,4,5,5-tetramethyl-2-(4-(1-phenylcyclopropyl)phenyl)-1,3,2-dioxaborolane[19] (3f)

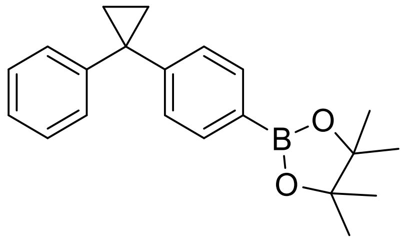

The title product was prepared according to General Procedure C at a 0.50 mmol scale using $NiBr_2$(dme) (10.8 mg, 0.035 mmol, 0.07 equiv), $^{t\text{-}Bu}$BpyCam^{CN} (11.8 mg, 0.035 mmol, 0.07 equiv), zinc flake (64.6 mg, 1.0 mmol, 2.0 equiv), and DMA (0.64 mL) employing 1,3-dioxoisoindolin-2-yl 1-phenylcyclopropane-1-carboxylate(154 mg, 0.5 mmol, 1.0 equiv) and 2-(4-iodophenyl)-4,4,5,5-tetramethyl-1,3,2-dioxaborolane (165 mg, 0.5 mmol, 1.0 equiv) at r.t. (20-22 °C). Purification of the crude reaction mixture using Purification Method A afforded the title product as a white powder, 99 mg (62% yield)
^1H NMR (500 MHz, $CDCl_3$) δ 7.71 (d, J = 8.2 Hz, 2H), 7.28 – 7.20 (m, 6H), 7.17 (tt, J = 7.1, 7.0, 1.3, 1.3 Hz, 1H), 1.32 (s, 12H), 1.31 (s, 4H).
$^{13}C\{^1H\}$ NMR (126 MHz, $CDCl_3$) δ 149.1, 145.4, 134.8, 128.5, 128.3, 127.7, 126.0, 83.7, 30.0, 24.9, 24.8, 16.6.
HRMS (ESI) m/z calculated for $C_{21}H_{26}BO_2$ [M+H]+ 320.2057, found 320.2054.
MP = 98-100 °C.

1-methoxy-2-(1-phenylcyclopropyl)benzene (3g)

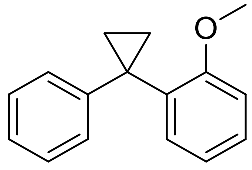

The title product was prepared according to General Procedure C at a 0.50 mmol scale using $NiBr_2$(dme) (10.8 mg, 0.035 mmol, 0.07 equiv), Bathophenanthroline (11.6 mg, 0.035 mmol, 0.07 equiv), zinc flake (64.6 mg, 1.0 mmol, 2.0 equiv), and DMA (0.64 mL) employing 1,3-dioxoisoindolin-2-yl 1-phenylcyclopropane-1-carboxylate (154 mg, 0.50 mmol, 1.0 equiv) and 2-iodoanisole (116 mg, 0.50 mmol, 1.0 equiv) at r.t. (20-22 °C). Purification of the crude reaction mixture using Purification Method A afforded the title product as a yellow oil, 46 mg (41% yield)
^1H NMR (400 MHz, $CDCl_3$) δ 7.41 (dd, J = 7.5, 1.8 Hz, 1H), 7.33 – 7.02 (m, 7H), 6.93 (td, J = 7.4, 1.2 Hz, 1H), 6.85 (dd, J = 8.3, 1.2 Hz, 1H), 3.76 (s, 3H), 1.28 (m, 2H), 1.22 (m, 2H).
$^{13}C\{^1H\}$ NMR (101 MHz, $CDCl_3$) δ 159.1, 146.1, 133.1, 131.5, 128.0, 127.9, 126.8, 125.2, 120.4, 110.9, 55.4, 26.1, 16.3.
HRMS (ESI) m/z calculated for $C_{16}H_{17}O$ [M+H]+ 225.1274, found 225.1273

1-methyl-5-(1-phenylcyclopropyl)-1H-indazole (3h)

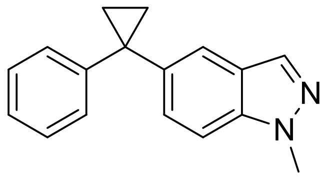

The title product was prepared according to General Procedure C at a 0.50 mmol scale using $NiBr_2$(dme) (5.4 mg, 0.0175 mmol, 0.07 equiv), $^{t\text{-Bu}}$BpyCam^{CN} (5.9 mg, 0.0175 mmol, 0.07 equiv), zinc flake (32.3 mg, 0.5 mmol, 2.0 equiv), and DMA (0.32 mL) employing 1,3-dioxoisoindolin-2-yl 1-phenylcyclopropane-1-carboxylate (77 mg, 0.25 mmol, 1.0 equiv) and 5-iodo-1-methyl-indazole (32.7 mg, 0.25 mmol, 1.0 equiv) at r.t. (20-22 °C). Purification of the crude reaction mixture using Purification Method A afforded the title product as a colorless oil, 27 mg (43% yield)

^{1}H NMR (500 MHz, $CDCl_3$) δ 7.90 (d, *J* = 1.0 Hz, 1H), 7.64 (dd, *J* = 1.6, 0.9 Hz, 1H), 7.36 (dd, *J* = 8.7, 1.6 Hz, 1H), 7.30 (dt, *J* = 8.7, 1.0 Hz, 1H), 7.27 – 7.22 (m, 2H), 7.21 – 7.18 (m, 2H), 7.15 (tt, *J* = 6.8, 1.3 Hz, 1H), 4.04 (s, 3H), 1.33 (m, 4H).

^{13}C{^{1}H} NMR (126 MHz, $CDCl_3$) δ 146.3, 138.8, 137.9, 132.6, 128.5, 128.2, 127.7, 125.8, 124.1, 120.6, 108.8, 35.6, 29.9, 16.3.

HRMS (ESI) m/z calculated for $C_{17}H_{17}N_2$ [M+H]+ 249.13863, found 249.1383

MP = 63-66 °C.

1-methoxy-4-(2-phenylcyclopropyl)benzene (3i)

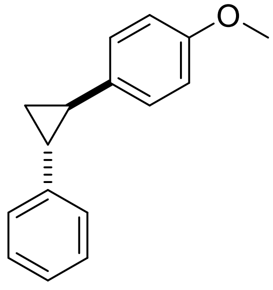

The title product was prepared according to General Procedure C at a 0.50 mmol scale using $NiBr_2$(dme) (10.8 mg, 0.035 mmol, 0.07 equiv), $^{t\text{-Bu}}$BpyCam^{CN} (11.8 mg, 0.035 mmol, 0.07 equiv), zinc flake (64.6 mg, 1.0 mmol, 2.0 equiv), and DMA (0.64 mL) employing 1,3-dioxoisoindolin-2-yl trans-2-phenylcyclopropane-1-carboxylate (154 mg, 0.5 mmol, 1.0 equiv) and 4-iodoanisole (116 mg, 0.50 mmol, 1.0 equiv) at r.t. (20-22 °C). Purification of the crude reaction mixture using Purification Method A afforded the title product as a colorless oil, 40 mg (36% yield). Characterization data matched those reported in the literature.[20]

^{1}H NMR (400 MHz, $CDCl_3$) δ 7.41 (dd, *J* = 7.5, 1.8 Hz, 1H), 7.33 – 7.02 (m, 7H), 6.93 (td, *J* = 7.4, 1.2 Hz, 1H), 6.85 (dd, *J* = 8.3, 1.2 Hz, 1H), 3.76 (s, 3H), 1.28 (m, 2H), 1.22 (m, 2H).

^{13}C{^{1}H} NMR (101 MHz, $CDCl_3$) δ 159.1, 146.1, 133.1, 131.5, 128.0, 127.9, 126.8, 125.2, 120.4, 110.9, 55.4, 26.1, 16.3.

HRMS (ESI) m/z calculated for $C_{16}H_{17}O$ [M+H]+ 225.1274, found 225.1272

4,4'-(cyclopropane-1,1-diyl)bis(methoxybenzene) (3j)

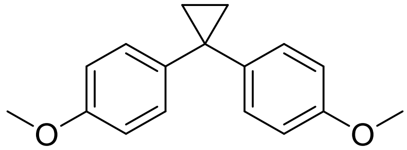

The title product was prepared according to General Procedure C at a 0.25 mmol scale using $NiBr_2$(dme) (5.4 mg, 0.0175 mmol, 0.07 equiv), $^{t\text{-Bu}}$BpyCam^{CN} (5.9 mg, 0.0175 mmol, 0.07 equiv), zinc flake (32.3 mg, 0.5 mmol, 2.0 equiv), and DMA (0.32 mL) employing 1,3-dioxoisoindolin-2-yl 1-(4-methoxyphenyl)cyclopropane-1-carboxylate (84.3 mg, 0.25 mmol, 1.0 equiv) and 1-iodo-4-methoxybenzene (58.5 mg, 0.25 mmol, 1.0 equiv). Purification Method A afforded the title product as a white solid, 45 mg (71% yield).

^{1}H NMR (500 MHz, $CDCl_3$) δ 7.18 – 7.10 (m, 4H), 6.83 – 6.76 (m, 4H), 3.76 (s, 6H), 1.20 (s, 4H).

^{13}C{^{1}H} NMR (126 MHz, $CDCl_3$) δ 157.7, 138.3, 129.3, 113.6, 55.2, 28.5, 16.0.

HRMS (ESI-MS) $[M+H]^+$ *m/z* calculated for $C_{17}H_{19}O_2^+$ 255.1380, found 255.1375.

MP = 56 – 58 °C.

1-chloro-4-(1-(4-methoxyphenyl)cyclopropyl)benzene (3k)

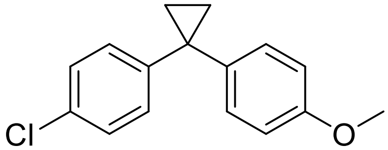

The title product was prepared according to General Procedure C at a 0.25 mmol scale using $NiBr_2$(dme) (5.4 mg, 0.0175 mmol, 0.07 equiv), $^{t\text{-Bu}}$BpyCam^{CN} (5.9 mg, 0.0175 mmol, 0.07 equiv), zinc flake (32.3 mg, 0.5 mmol, 2.0 equiv), and DMA (0.32 mL) employing 1,3-dioxoisoindolin-2-yl 1-(4-chlorophenyl)cyclopropane-1-carboxylate (85.4 mg, 0.25 mmol, 1.0 equiv) and 1-iodo-4-methoxybenzene (58.5 mg, 0.25 mmol, 1.0 equiv). Purification Method A afforded the title product as a colorless oil, 25.9 mg (40% yield).

^{1}H NMR (500 MHz, $CDCl_3$) δ 7.22 – 7.18 (m, 2H), 7.18 – 7.14 (m, 2H), 7.12 – 7.08 (m, 2H), 6.85 – 6.78 (m, 2H), 3.77 (s, 3H), 1.27 – 1.24 (m, 2H), 1.23 – 1.20 (m, 2H).

^{13}C{^{1}H} NMR (126 MHz, $CDCl_3$) δ 158.0, 144.8, 137.2, 131.4, 129.7, 129.3, 128.3, 113.7, 55.3, 28.7, 16.3.

HRMS (ESI-MS) $[M+H]^+$ *m/z* calculated for $C_{16}H_{16}ClO^+$ 259.0884, found 259.0879.

3-(1-(4-methoxyphenyl)cyclopropyl)thiophene (3l)

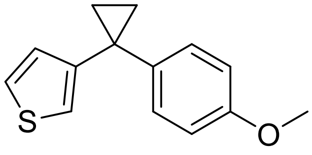

The title product was prepared according to General Procedure C at a 0.25 mmol scale using $NiBr_2$(dme) (5.4 mg, 0.0175 mmol, 0.07 equiv), $^{t\text{-Bu}}$BpyCam^{CN} (5.9 mg, 0.0175 mmol, 0.07 equiv), zinc flake (32.3 mg, 0.5 mmol, 2.0 equiv), and DMA (0.32 mL) employing 1,3-dioxoisoindolin-2-yl 1-(thiophen-3-yl)cyclopropane-1-carboxylate (78.3 mg, 0.25 mmol, 1.0 equiv) and 1-iodo-4-methoxybenzene (58.5 mg, 0.25 mmol, 1.0 equiv). Purification Method A afforded the title product as a colorless oil, 32.8 mg (57% yield).

^{1}H NMR (500 MHz, $CDCl_3$) δ 7.28 – 7.21 (m, 2H), 7.18 (dd, J = 5.0, 3.0 Hz, 1H), 6.87 – 6.80 (m, 2H), 6.78 (dd, J = 5.0, 1.4 Hz, 1H), 6.69 (dd, J = 3.0, 1.4 Hz, 1H), 3.79 (s, 3H), 1.26 – 1.18 (m, 4H).

^{13}C{^{1}H} NMR (126 MHz, $CDCl_3$) δ 158.1, 148.3, 137.1, 130.2, 126.6, 125.3, 119.9, 113.7, 55.3, 25.8, 16.6.

HRMS (ESI-MS) $[M+H]^+$ *m/z* calculated for $C_{14}H_{15}OS^+$ 231.0838, found 231.0834.

IR (cm^{-1})

2,6-dimethoxy-3-(1-(4-methoxyphenyl)cyclopropyl)pyridine (3m)

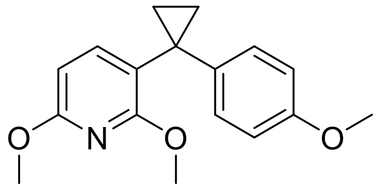

The title product was prepared according to General Procedure C at a 0.25 mmol scale using $NiBr_2$(dme) (5.4 mg, 0.0175 mmol, 0.07 equiv), $^{t\text{-Bu}}$BpyCam^{CN} (5.9 mg, 0.0175 mmol, 0.07 equiv), zinc flake (32.3 mg, 0.5 mmol, 2.0 equiv), and DMA (0.32 mL) employing 1,3-dioxoisoindolin-2-yl 1-(2,6-dimethoxypyridin-3-yl)cyclopropane-1-carboxylate (92.1 mg, 0.25 mmol, 1.0 equiv) and 1-iodo-4-methoxybenzene (58.5 mg, 0.25 mmol, 1.0 equiv). Purification Method A afforded the title product as a white solid, 67.1 mg (94% yield).

^{1}H NMR (500 MHz, $CDCl_3$) δ 7.56 (d, J = 7.9 Hz, 1H), 7.18 – 7.09 (m, 2H), 6.78 – 6.72 (m, 2H), 6.24 (d, J = 8.0 Hz, 1H), 3.91, (s, 3H), 3.88 (s, 3H), 3.74 (s, 3H), 1.22 – 1.14 (m, 2H), 1.14 – 1.06 (m, 2H).

^{13}C{^{1}H} NMR (126 MHz, $CDCl_3$) δ 161.6, 161.4, 157.5, 142.2, 137.9, 128.2, 118.7, 113.4, 100.0, 55.2, 53.4, 53.2, 24.2, 15.4.

HRMS (ESI-MS) $[M+H]^+$ *m/z* calculated for $C_{17}H_{20}NO_3^+$ 286.1438, found 286.1433.

MP = 79 – 81 °C.

5-(1-(4-methoxyphenyl)cyclopropyl)benzo[b]thiophene (3n)

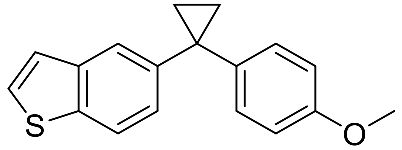

The title product was prepared according to General Procedure C at a 0.25 mmol scale using $NiBr_2$(dme) (5.4 mg, 0.0175 mmol, 0.07 equiv), $^{t\text{-Bu}}$BpyCam^{CN} (5.9 mg, 0.0175 mmol, 0.07 equiv), zinc flake (32.3 mg, 0.5 mmol, 2.0 equiv), and DMA (0.32 mL) employing 1,3-dioxoisoindolin-2-yl 1-(benzo[b]thiophen-5-yl)cyclopropane-1-carboxylate (90.8 mg, 0.25 mmol, 1.0 equiv) and 1-iodo-4-methoxybenzene (58.5 mg, 0.25 mmol, 1.0 equiv). Purification Method A afforded the title product as a colorless oil, 54 mg (77% yield).

^{1}H NMR (500 MHz, $CDCl_3$) δ 7.74 (d, J = 8.4 Hz, 1H), 7.67 (d, J = 1.7 Hz, 1H), 7.38 (d, J = 5.4 Hz, 1H), 7.26 – 7.21 (m, 2H), 7.21 – 7.16 (m, 2H), 6.84 – 6.77 (m, 2H), 3.76 (s, 3H), 1.33 – 1.26 (m, 4H).

^{13}C{^{1}H} NMR (126 MHz, $CDCl_3$) δ 157.8, 142.4, 139.7, 138.1, 137.4, 129.5, 126.6, 125.2, 123.8, 123.1, 122.2, 113.7, 55.2, 29.3, 16.2.

HRMS (ESI-MS) $[M+H]^+$ *m/z* calculated for $C_{18}H_{17}OS^+$ 281.0995, found 291.0988.

methyl 5-(1-phenylcyclopropyl)nicotinate (3o)

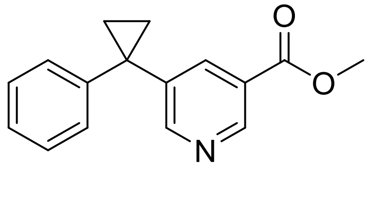

The title product was prepared according to General Procedure C at a 0.50 mmol scale using $NiBr_2$(dme) (10.8 mg, 0.035 mmol, 0.07 equiv), $^{t\text{-Bu}}$BpyCam^{CN} (11.8 mg, 0.035 mmol, 0.07 equiv), zinc flake (64.6 mg, 1.0 mmol, 2.0 equiv), and THF (0.64 mL) employing 1,3-dioxoisoindolin-2-yl 1-phenylcyclopropane-1-carboxylate (154 mg, 0.5 mmol, 1.0 equiv) and methyl 5-bromonicotinate (108 mg, 0.5 mmol, 1.0 equiv) at 40 °C. Purification of the crude reaction mixture using Purification Method A afforded the title product as a colorless oil, 69 mg (with 10% phthalimide) (50% yield)

^{1}H NMR (600 MHz, $CDCl_3$) δ 9.03 (d, J = 2.0 Hz, 1H), 8.65 (d, J = 2.3 Hz, 1H), 8.13 (t, J = 2.1 Hz, 1H), 7.35 – 7.27 (m, 2H), 7.26 – 7.18 (m, 3H), 3.93 (s, 3H), 1.42 – 1.39 (m, 2H), 1.35 – 1.32 (m, 2H).

^{13}C{^{1}H} NMR (151 MHz, $CDCl_3$) δ 165.9, 153.9, 148.3, 143.7, 141.3, 136.4, 128.7, 128.6, 126.7, 125.6, 52.4, 27.8, 16.0.

HRMS (ESI) m/z calculated for $C_{16}H_{16}NO_2$ [M+H]+ 254.11756, found 254.1171

methyl 5-(1-methylcyclopropyl)nicotinate (3p)

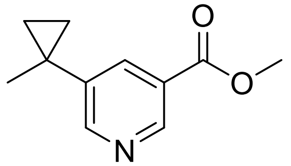

The title product was prepared according to General Procedure C at a 0.25 mmol scale using $NiBr_2$(dme) (15.4 mg, 0.05 mmol, 0.20 equiv), ^{tbu}bpyCam^{CN} (16.8 mg, 0.05 mmol, 0.2 equiv), zinc flake (33 mg, 0.50 mmol, 2.0 equiv), and THF (0.32 mL) employing 1,3-dioxoisoindolin-2-yl 1-methylcyclopropane-1-carboxylate (61.3 mg, 0.25 mmol, 1.0 equiv) and and methyl 5-bromonicotinate (54 mg, 0.25 mmol, 1.0 equiv) at 40 °C. Purification of the crude reaction mixture using Purification Method A afforded the title product as a colorless oil, 20 mg (42% yield).

^{1}H NMR (500 MHz, $CDCl_3$) δ 9.02 (d, J = 2.0 Hz, 1H), 8.68 (d, J = 2.3 Hz, 1H), 8.12 (t, J = 2.2 Hz, 1H), 3.95 (s, 3H), 1.46 (s, 3H), 0.95 – 0.91 (m, 2H), 0.87 – 0.83 (m, 2H).

^{13}C{^{1}H} NMR (126 MHz, $CDCl_3$) δ 166.2, 152.6, 148.1, 142.5, 135.3, 125.6, 77.2, 52.5, 25.2, 17.9, 15.7.

HRMS (ESI) m/z calculated for $C_{11}H_{14}NO_2$[M+H]+ 192.1019, found 192.1016

2-fluoro-5-(1-phenylcyclopropyl)pyridine (3q)

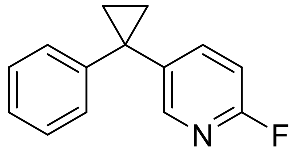

The title product was prepared according to General Procedure C at a 0.50 mmol scale using $NiBr_2$(dme) (10.8 mg, 0.035 mmol, 0.07 equiv), $^{t\text{-Bu}}$BpyCam^{CN} (11.8 mg, 0.035 mmol, 0.07 equiv), zinc flake (64.6 mg, 1.00 mmol, 2.00 equiv), and THF (0.64 mL) employing 1,3-dioxoisoindolin-2-yl 1-phenylcyclopropane-1-carboxylate (154 mg, 0.50 mmol, 1.00 equiv) and 5-bromo-2-fluoropyridine (88 mg, 0.5 mmol, 1.0 equiv) at 40 °C. Purification of the crude reaction mixture using Purification Method A afforded the title product as a pale yellow oil, 26 mg (25% yield)

^{1}H NMR (600 MHz, $CDCl_3$) δ 8.10 (dt, J = 1.8, 0.9 Hz, 1H), 7.64 (ddd, J = 8.4, 7.7, 2.6 Hz, 1H), 7.32 – 7.26 (m, 2H), 7.24 – 7.17 (m, 3H), 6.82 (ddd, J = 8.5, 3.1, 0.7 Hz, 1H), 1.37 – 1.35 (m, 2H), 1.29 – 1.26 (m, 2H).

$^{13}C\{^1H\}$ NMR (151 MHz, $CDCl_3$) δ 162.13 (d, J = 237.7 Hz), 147.47 (d, J = 14.7 Hz), 144.27, 141.55 (d, J = 7.9 Hz), 138.90 (d, J = 4.6 Hz), 128.59, 128.16, 126.51, 108.95 (d, J = 34.5 Hz), 27.16 (d, J = 1.7 Hz), 15.91.
$^{19}F\{^1H\}$ NMR (377 MHz, $CDCl_3$) δ -71.6.
HRMS (ESI) m/z calculated for $C_{14}H_{13}NF$ [M+H]+ 214.1027, found 214.1027

2-chloro-4-(1-phenylcyclopropyl)pyridine (3r)

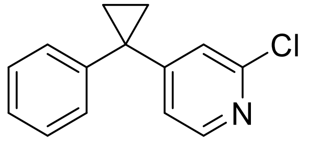

The title product was prepared according to General Procedure C at a 0.50 mmol scale using $NiBr_2$(dme) (10.8 mg, 0.035 mmol, 0.07 equiv), $^{t\text{-Bu}}$BpyCam^{CN} (11.8 mg, 0.035 mmol, 0.07 equiv), zinc flake (64.6 mg, 1.0 mmol, 2.0 equiv), and THF (0.64 mL) employing 1,3-dioxoisoindolin-2-yl 1-phenylcyclopropane-1-carboxylate (154 mg, 0.5 mmol, 1.0 equiv) and 4-bromo-2-chloropyridine (95 mg, 0.50 mmol, 1.0 equiv) at 40 °C. Purification of the crude reaction mixture using Purification Method A afforded the title product as a colorless oil, 64 mg (56% yield)
^1H NMR (500 MHz, $CDCl_3$) δ 8.18 (d, J = 5.3 Hz, 1H), 7.39 – 7.32 (m, 2H), 7.29 (dt, J = 5.8, 1.5 Hz, 3H), 6.93 (d, J = 1.7 Hz, 1H), 6.83 (dd, J = 5.3, 1.7 Hz, 1H), 1.48 – 1.42 (m, 2H), 1.38 – 1.32 (m, 2H).
$^{13}C\{^1H\}$ NMR (126 MHz, $CDCl_3$) δ 159.1, 151.7, 149.2, 142.2, 129.9, 128.8, 127.3, 122.1, 120.5, 29.2, 18.0.
HRMS (ESI) m/z calculated for $C_{15}H_{13}ClN$ [M+H]+ 230.0730, found 230.0731

2-(1-phenylcyclopropyl)quinoline (3s)

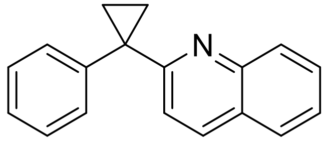

The title product was prepared according to General Procedure C at a 0.50 mmol scale using $NiBr_2$(dme) (10.8 mg, 0.035 mmol, 0.07 equiv), $^{t\text{-Bu}}$BpyCam^{CN} (11.8 mg, 0.035 mmol, 0.07 equiv), zinc flake (64.6 mg, 1.0 mmol, 2.0 equiv), and THF (0.64 mL) employing 1,3-dioxoisoindolin-2-yl 1-phenylcyclopropane-1-carboxylate (154 mg, 0.5 mmol, 1.0 equiv) and 5-bromo-2-fluoropyridine (88 mg, 0.5 mmol, 1.0 equiv) at r.t. (20-22 °C). Purification of the crude reaction mixture using Purification Method A afforded the title product as a pale yellow oil, 36 mg (29% yield)
^1H NMR (500 MHz, $CDCl_3$) δ 8.01 (d, J = 8.5 Hz, 1H), 7.89 (d, J = 8.5 Hz, 1H), 7.72 (dd, J = 8.2, 1.4 Hz, 1H), 7.66 (ddd, J = 8.4, 6.8, 1.5 Hz, 1H), 7.52 – 7.40 (m, 3H), 7.39 – 7.33 (m, 2H), 7.29 (1, 1H), 7.08 (1, J = 8.6 Hz, 1H), 1.85 (m, 2H), 1.38 (m, 2H).
$^{13}C\{^1H\}$ NMR (126 MHz, $CDCl_3$) δ 164.3, 147.8, 143.7, 135.4, 130.4, 129.2, 129.1, 128.6, 127.4, 126.8, 126.4, 125.5, 121.2, 32.3, 17.7, 0.0.
HRMS (ESI) m/z calculated for $C_{18}H_{16}N$ [M+H]+ 246.12773, found 246.1277

Methyl 6-(1-(3,5,5,8,8-pentamethyl-5,6,7,8-tetrahydronaphthalen-2-yl)cyclopropyl)nicotinate (3t)

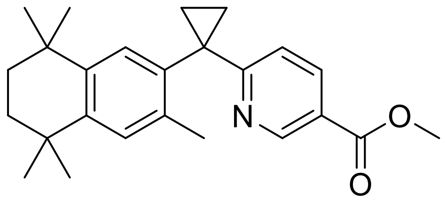

The title product was prepared according to General Procedure C at a 0.25 mmol scale using $NiBr_2$(dme) (5.4 mg, 0.0175 mmol, 0.07 equiv), $^{t\text{-Bu}}$BpyCam^{CN} (5.9 mg, 0.0175 mmol, 0.07 equiv), zinc flake (32.3 mg, 0.5 mmol, 2.0 equiv), and DMA (0.32 mL) employing 1,3-dioxoisoindolin-2-yl 1-(3,5,5,8,8-pentamethyl-5,6,7,8-tetrahydronaphthalen-2-yl)cyclopropane-1-carboxylate (92.1 mg, 0.25 mmol, 1.0 equiv) and methyl 6-bromonicotinate (54 mg, 0.25 mmol, 1.0 equiv). Purification Method A afforded the title product as a white solid, 20.1 mg (21% yield).

^{1}H NMR (500 MHz, $CDCl_3$) δ 9.08 (dd, J = 2.2, 0.8 Hz, 1H), 7.97 (dd, J = 8.3, 2.2 Hz, 1H), 7.27 (s, 1H), 7.11 (s, 1H), 6.74 (dd, J = 8.3, 0.8 Hz, 1H), 3.90 (s, 3H), 2.11 (s, 3H), 1.83 (m, 2H), 1.69 (s, 4H), 1.35 (m, 2H), 1.30 (s, 6H), 1.27 (s, 6H).

^{13}C{^{1}H} NMR (126 MHz, $CDCl_3$) δ 169.3, 166.1, 150.5, 143.8, 142.7, 137.2, 136.6, 135.8, 129.2, 128.3, 122.2, 120.7, 52.1, 35.2, 35.2, 34.0, 33.9, 32.0, 31.9, 30.3, 20.2, 19.3.

HRMS (ESI-MS) $[M+H]^+$ *m/z* calculated for $C_{25}H_{32}NO_2^+$ 378.2428, found 378.2421.

MP = 182 – 183 °C.

1-(4-methoxyphenyl)bicyclo[1.1.1]pentane (3u)

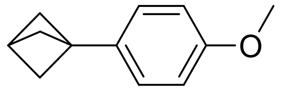

The title product was prepared according to General Procedure D at a 0.300 mmol scale using $NiBr_2$(dme) (18.5 mg, 0.060 mmol, 0.20 equiv), $^{t\text{-Bu}}$BpyCam^{CN} (20.1 mg, 0.060 mmol, 0.20 equiv), zinc dust (39.2 mg, 0.6 mmol, 2.0 equiv), and THF (0.450 mL)/DMA (0.050 mL), employing 1,3-dioxoisoindolin-2-yl bicyclo[1.1.1]pentane-1-carboxylate (77.1 mg, 0.300 mmol, 1.0 equiv) and 4-iodoanisole (70.2 mg, 0.30 mmol, 1.0 equiv). Purification of the crude reaction mixture using Purification Method A afforded the title product (5.8 mg, 11% yield) as a white solid. Modification of this procedure using 5-methoxy-1,3-dioxoisoindolin-2-yl bicyclo[1.1.1]pentane-1-carboxylate (86.2 mg, 0.300 mmol, 1.0 equiv) afforded the title product (22 mg, 42% yield)

^{1}H NMR (500 MHz, $CDCl_3$) δ 7.14 (d, J = 8.7 Hz, 2H), 6.83 (d, J = 8.7 Hz, 2H), 3.79 (s, 3H), 2.53 (s, 1H), 2.05 (s, 6H).

^{13}C{^{1}H} NMR (151 MHz, $CDCl_3$) δ 158.2, 134.2, 127.0, 113.5, 55.3, 52.2, 46.7, 26.5.

HRMS (ESI) m/z calculated for $C_{12}H_{15}O$ [M]+ 175.1117, found 175.1117

FTIR (ATR, cm^{-1}) 2966, 2906, 2869, 1737, 1519, 1501, 1246, 1207, 1174, 1032, 833.

MP = 48-50 °C.

3-(4-methoxyphenyl)-N,N-dimethylbicyclo[1.1.1]pentane-1-carboxamide (3v)

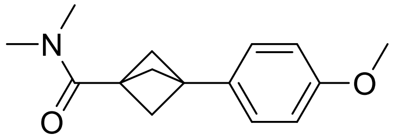

The title product was prepared according to General Procedure D at a 0.300 mmol scale using $NiBr_2$(dme) (18.5 mg, 0.060 mmol, 0.20 equiv), $^{t\text{-Bu}}$BpyCam^{CN} (20.1 mg, 0.060 mmol, 0.20 equiv),

zinc dust (39.2 mg, 0.6 mmol, 2.0 equiv), and THF (0.450 mL)/DMA (0.050 mL), employing 1,3-dioxoisoindolin-2-yl 3-(dimethylcarbamoyl)bicyclo[1.1.1]pentane-1-carboxylate (98.5 mg, 0.30 mmol, 1.0 equiv) and 4-iodoanisole (70.2 mg, 0.30 mmol, 1.0 equiv). Purification of the crude reaction mixture using Purification Method B afforded the title product (38.4 mg, 157 μmol, 52%) as a white solid.
^{1}H NMR (600 MHz, $CDCl_3$) δ 7.17 - 7.12 (m, 2H), 6.88 - 6.81 (m, 2H), 3.80 (s, 3H), 3.15 (s, 3H), 2.96 (s, 3H), 2.37 (s, 6H).
^{13}C{^{1}H} NMR (151 MHz, $CDCl_3$) δ 169.7, 158.5, 132.2, 127.1, 113.6, 55.3, 54.4, 42.1, 39.0, 37.3, 36.0.
HRMS (ESI) m/z calculated for $C_{15}H_{20}NO_2$ [M+H]+ 246.1489, found 246.1486.
FTIR (ATR, cm^{-1}) 1615, 1506, 1247, 1034, 912, 743, 646.
MP = 122-127 °C.

methyl 3-(4-methoxyphenyl)bicyclo[1.1.1]pentane-1-carboxylate (3w)

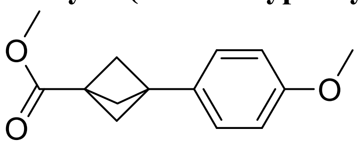

The title product was prepared according to General Procedure C at a 0.50 mmol scale using $NiBr_2$(dme) (10.8 mg, 0.035 mmol, 0.07 equiv), $^{t\text{-Bu}}$BpyCam^{CN} (11.8 mg, 0.035 mmol, 0.07 equiv), zinc flake (64.6 mg, 1.0 mmol, 2.0 equiv), DMA (0.064 mL) and THF (0.58 mL) employing 1-(1,3-dioxoisoindolin-2-yl) 3-methyl bicyclo[1.1.1]pentane-1,3-dicarboxylate (157 mg, 0.50 mmol, 1.0 equiv) and 4-iodoanisole (116 mg, 0.50 mmol, 1.0 equiv) at r.t. (20-22 °C). Purification of the crude reaction mixture using Purification Method A afforded the title product as a white powder, 58 mg (51% yield). Characterization data matched those reported in the literature.[21]
^{1}H NMR (500 MHz, CDCl3) δ 7.17 – 7.10 (m, 2H), 6.88 – 6.81 (m, 2H), 3.79 (s, 3H), 3.71 (s, 3H), 2.29 (s, 6H).
^{13}C{^{1}H} NMR (126 MHz, $CDCl_3$) δ 170.8, 158.7, 132.0, 127.2, 113.7, 55.3, 53.4, 51.7, 41.4, 36.9.
HRMS (ESI) m/z calculated for $C_{14}H_{17}O_3$ [M+H]+ 233.1172, found 233.1169.
MP = 113-116 °C.

methyl 3-(1-phenyl-1H-pyrazol-3-yl)bicyclo[1.1.1]pentane-1-carboxylate (3x)

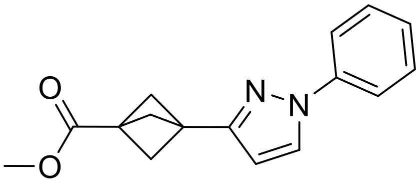

The title product was prepared according to General Procedure D at a 0.300 mmol scale using $NiBr_2$(dme) (18.5 mg, 0.060 mmol, 0.20 equiv), $^{t\text{-Bu}}$BpyCam^{CN} (20.1 mg, 0.060 mmol, 0.20 equiv), zinc dust (39.2 mg, 0.6 mmol, 2.0 equiv), and THF (0.450 mL)/DMA (0.050 mL),employing 1-(1,3-dioxoisoindolin-2-yl) 3-methyl bicyclo[1.1.1]pentane-1,3-dicarboxylate (94.6 mg, 0.30 mmol, 1.0 equiv) and 3-iodo-1-phenyl-1H-pyrazole (81.0 mg, 0.30 mmol, 1.0 equiv, 95% purity). Purification of the crude reaction mixture using Purification Method B afforded the title product (40.9 mg, 0.152 mmol, 51%) as a colorless oil.
^{1}H NMR (600 MHz, $CDCl_3$) δ 7.86 - 7.81 (m, 1H), 7.71 - 7.65 (m, 2H), 7.48 - 7.42 (m, 2H), 7.32 - 7.26 (m, 1H), 6.34 - 6.30 (m, 1H), 3.74 (s, 3H), 2.45 (s, 6H).

$^{13}C\{^1H\}$ **NMR** (151 MHz, $CDCl_3$) δ 170.5, 152.5, 140.0, 129.3, 127.6, 126.4, 119.4, 105.8, 53.8, 51.6, 38.2, 36.4.
HRMS (ESI) m/z calculated for $C_{16}H_{16}N_2O_2$ [M+H]+ 269.1285, found 269.1287.
FTIR (ATR, cm^{-1}) 2992, 1727, 1600, 1510, 1330, 1211, 1034, 760.

methyl 3-(1-methyl-1H-pyrrolo[2,3-b]pyridin-3-yl)bicyclo[1.1.1]pentane-1-carboxylate (3y)

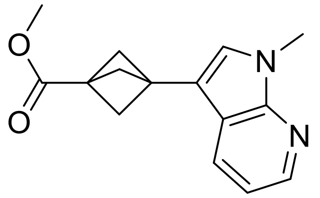

The title product was prepared according to General Procedure D at a 0.30 mmol scale using $NiBr_2$(dme) (18.5 mg, 0.060 mmol, 0.20 equiv), $^{t\text{-Bu}}$BpyCam^{CN} (20.1 mg, 0.060 mmol, 0.20 equiv), zinc dust (39.2 mg, 0.6 mmol, 2.0 equiv), and THF (0.450 mL)/DMA (0.050 mL),employing 1-(1,3-dioxoisoindolin-2-yl) 3-methyl bicyclo[1.1.1]pentane-1,3-dicarboxylate (94.6 mg, 0.30 mmol) and 3-iodo-1-methyl-1H-pyrrolo[2,3-b]pyridine (77.4 mg, 0.30 mmol, 1.0 equiv). Purification of the crude reaction mixture using Purification Method B afforded the title product (23.9 mg, 93.2 μmol, 31%) as a pale yellow solid.
^{1}**H NMR** (600 MHz, $CDCl_3$) δ 8.37 - 8.30 (m, 1H), 7.94 (dd, J = 1.5, 7.9 Hz, 1H), 7.11 - 7.04 (m, 1H), 6.99 (s, 1H), 3.86 (s, 3H), 3.72 (s, 3H), 2.44 (s, 6H).
$^{13}C\{^1H\}$ **NMR** (151 MHz, $CDCl_3$) δ 170.5, 147.7, 142.8, 127.8, 126.4, 119.6, 115.2, 112.4, 54.2, 51.7, 38.9, 36.4, 31.1.
HRMS (ESI) m/z calculated for $C_{15}H_{17}N_2O_2$ [M+H]+ 257.1285, found 257.1291.
FTIR (ATR, cm^{-1}) 2976, 2875, 1725, 1466, 1325, 1296, 1206, 1166, 772.
MP = 82-84 °C.

methyl 3-(1-methyl-1H-indazol-5-yl)bicyclo[1.1.1]pentane-1-carboxylate (3z)

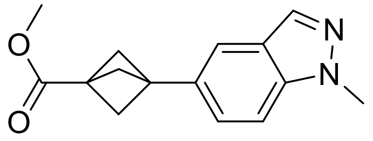

The title product was prepared according to General Procedure D at a 0.300 mmol scale using $NiBr_2$(dme) (18.5 mg, 0.060 mmol, 0.20 equiv), $^{t\text{-Bu}}$BpyCam^{CN} (20.1 mg, 0.060 mmol, 0.20 equiv), zinc dust (39.2 mg, 0.6 mmol, 2.0 equiv), and THF (0.450 mL)/DMA (0.050 mL),employing 1-(1,3-dioxoisoindolin-2-yl) 3-methyl bicyclo[1.1.1]pentane-1,3-dicarboxylate (94.6 mg, 0.30 mmol, 1.0 equiv) and 5-iodo-1-methyl-1H-indazole (77.4 mg, 0.30 mmol, 1.0 equiv). Purification of the crude reaction mixture using Purification Method B afforded the title product (30.8 mg, 0.120 mmol, 40%) as a semi-crystalline white solid.
^{1}**H NMR** (600 MHz, $CDCl_3$) δ 7.95 (s, 1H), 7.55 (dd, J = 0.9, 1.4 Hz, 1H), 7.37 - 7.34 (m, 1H), 7.32 - 7.27 (m, 1H), 4.08 (s, 3H), 3.75 (s, 3H), 2.39 (s, 6H).
$^{13}C\{^1H\}$ **NMR** (151 MHz, $CDCl_3$) δ 170.7, 139.1, 132.5, 132.1, 124.8, 123.9, 118.0, 108.8, 53.5, 51.7, 41.9, 36.9, 35.5.
HRMS (ESI) m/z calculated for $C_{15}H_{17}N_2O_2$ [M+H]+ 257.1285, found 257.1288.
FTIR (ATR, cm^{-1}) 2984, 2950, 2912, 2875, 1730, 1449, 1435, 1344, 1305, 1206, 1094, 986, 798.
MP = 137-139 °C.

methyl (R)-3-(4-(5-((1H-1,2,3-triazol-1-yl)methyl)-2-oxooxazolidin-3-yl)-2-fluorophenyl)bicyclo[1.1.1]pentane-1-carboxylate (3aa)

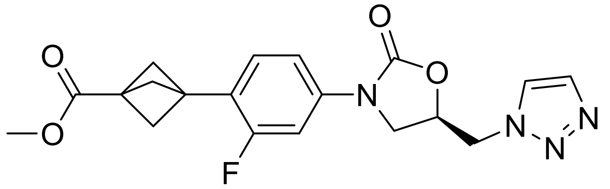

The title product was prepared according to General Procedure D at a 0.300 mmol scale using $NiBr_2$(dme) (18.5 mg, 0.060 mmol, 0.20 equiv), $^{t\text{-Bu}}$BpyCam^{CN} (20.1 mg, 0.060 mmol, 0.20 equiv), zinc dust (39.2 mg, 0.6 mmol, 2.0 equiv), and THF (0.810 mL)/DMA (0.090 mL),employing 1-(1,3-dioxoisoindolin-2-yl) 3-methyl bicyclo[1.1.1]pentane-1,3-dicarboxylate (94.6 mg, 0.30 mmol, 1.0 equiv), (R)-5-((1H-1,2,3-triazol-1-yl)methyl)-3-(3-fluoro-4-iodophenyl)oxazolidin-2-one (116.4 mg, 0.30 mmol, 1.0 equiv). Purification of the crude reaction mixture using Purification Method B afforded the title product (11.7 mg, 30.3 mol, 15%) as a white solid.

^{1}H NMR (600 MHz, $CDCl_3$) δ 7.92 - 7.66 (m, 2H), 7.25 (m, 1H), 7.10 - 7.04 (m, 1H), 7.03 - 6.98 (m, 1H), 5.14 - 5.01 (m, 1H), 4.86 - 4.71 (m, 2H), 4.22 - 4.09 (m, 1H), 3.98 - 3.87 (m, 1H), 3.75 - 3.67 (m, 3H), 2.40 - 2.34 (m, 6H).

^{13}C{^{1}H} NMR (151 MHz, $CDCl_3$) δ 170.4, 161.9 (d, *J* = 247.8 Hz), 153.2, 137.8 (d, *J* = 10.3 Hz), 134.7, 129.3 (d, *J* = 6.2 Hz), 125.3, 122.9 (d, *J* = 15.7 Hz), 113.4 (d, *J* = 3.3 Hz), 106.4 (d, *J* = 27.0 Hz), 70.5, 53.8, 52.2, 51.8, 47.4, 38.7, 38.3.

^{19}F{^{1}H} NMR (471 MHz, $CDCl_3$) δ -113.9.

HRMS (ESI) m/z calculated for $C_{19}H_{20}N_4O_4$ [M+H]+ 387.1463, found 387.1461.

FTIR (ATR, cm^{-1}) 1749, 1723, 1409, 1435, 1409, 1207, 1125.

MP = 162-166 °C.

methyl 3-(4-(N-(tert-butoxycarbonyl)acetamido)phenyl)bicyclo[1.1.1]pentane-1-carboxylate (3ab)

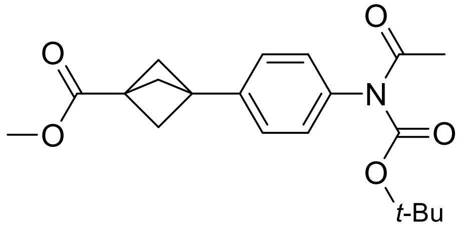

The title product was prepared according to General Procedure D at a 0.300 mmol scale using $NiBr_2$(dme) (18.5 mg, 0.060 mmol, 0.20 equiv), $^{t\text{-Bu}}$BpyCam^{CN} (20.1 mg, 0.060 mmol, 0.20 equiv), zinc dust (39.2 mg, 0.6 mmol, 2.0 equiv), and THF (0.450 mL)/DMA (0.050 mL),employing 1-(1,3-dioxoisoindolin-2-yl) 3-methyl bicyclo[1.1.1]pentane-1,3-dicarboxylate (94.6 mg, 0.30 mmol, 1.0 equiv), tert-butyl acetyl(4-iodophenyl)carbamate (108 mg, 0.30 mmol, 1.0 equiv). Purification of the crude reaction mixture using Purification Method A afforded the title product (71.8 mg, 0.20 mmol, 67%) as a white solid. Analytical data was in accordance with that reported in the literature.[8]

^{1}H NMR (600 MHz, $CDCl_3$) δ 7.24 - 7.20 (m, 2H), 7.05 - 6.99 (m, 2H), 3.71 (s, 3H), 2.56 (s, 3H), 2.33 (s, 6H), 1.40 (s, 9H).

^{13}C{^{1}H} NMR (151 MHz, $CDCl_3$) δ 172.9, 170.6, 152.8, 139.2, 137.6, 128.0, 126.8, 83.3, 53.4, 51.7, 41.6, 37.0, 27.9, 26.5.
HRMS (ESI) m/z calculated for $C_{20}H_{25}NO_5Na$ [M+Na]+ 382.1625, found 382.1630.

methyl 3-(4-acetamidophenyl)bicyclo[1.1.1]pentane-1-carboxylate (3ac)

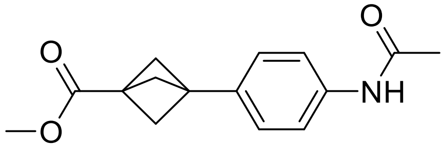

The title product was prepared according to General Procedure D at a 0.300 mmol scale using $NiBr_2$(dme) (18.5 mg, 0.060 mmol, 0.20 equiv), $^{t\text{-Bu}}$BpyCam^{CN} (20.1 mg, 0.060 mmol, 0.20 equiv), zinc dust (39.2 mg, 0.6 mmol, 2.0 equiv), and THF (0.450 mL)/DMA (0.050 mL),employing 1-(1,3-dioxoisoindolin-2-yl) 3-methyl bicyclo[1.1.1]pentane-1,3-dicarboxylate (94.6 mg, 0.30 mmol, 1.0 equiv), *N*-(4-iodophenyl)acetamide (78.3 mg, 0.30 mmol, 1.0 equiv). Purification of the crude reaction mixture using Purification Method B afforded the title product (50.4 mg, 0.194 mmol, 65%) as a white solid.
^{1}H NMR (600 MHz, $CDCl_3$) δ 7.80 (br s, 1H), 7.45 (d, J = 8.4 Hz, 1H), 7.14 (d, J = 8.2 Hz, 2H), 3.71 (s, 3H), 2.29 (s, 6H), 2.15 (s, 3H).
^{13}C{^{1}H} NMR (151 MHz, $CDCl_3$) δ 170.7, 168.6, 136.8, 135.6, 126.6, 119.9, 53.3, 51.6, 41.4, 36.8, 24.4.
HRMS (ESI) m/z calculated for $C_{15}H_{18}NO_3$ [M+H]+ 260.1281, found 260.1279.
MP = 172-175 °C.
FTIR (ATR, cm^{-1}) 2984, 1720, 1667, 1600, 1530, 1507, 1405, 1298, 1210, 911, 740.

methyl 3-(4-acetoxyphenyl)bicyclo[1.1.1]pentane-1-carboxylate (3ad)

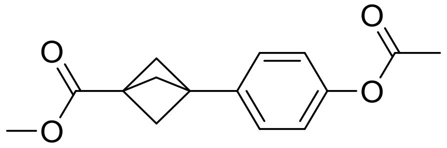

The title product was prepared according to General Procedure C at a 0.25 mmol scale using $NiBr_2$(dme) (14.6 mg, 0.05 mmol, 0.20 equiv), $^{t\text{-Bu}}$BpyCam^{CN} (16.7 mg, 0.05 mmol, 0.20 equiv), zinc flake (32.3 mg, 0.5 mmol, 2.0 equiv), and THF (0.32 mL) employing 1-(1,3-dioxoisoindolin-2-yl) 3-methyl bicyclo[1.1.1]pentane-1,3-dicarboxylate (79 mg, 0.25 mmol, 1.0 equiv) and 4-iodophenyl acetate (65 mg, 0.25 mmol, 1.0 equiv) at r.t. (20-22 °C). Purification of the crude reaction mixture using Purification Method A afforded the title product as a colorless oil, 27 mg (42% yield). Characterization data matched those reported in the literature.[8]
The title product was prepared according to General Procedure C at a 0.25 mmol scale using $NiBr_2$(dme) (14.6 mg, 0.05 mmol, 0.20 equiv), $^{t\text{-Bu}}$BpyCam^{CN} (16.7 mg, 0.05 mmol, 0.20 equiv), zinc flake (32.3 mg, 0.5 mmol, 2.0 equiv), and THF (0.32 mL) employing 1-(1,3-dioxoisoindolin-2-yl) 3-methyl bicyclo[1.1.1]pentane-1,3-dicarboxylate (79 mg, 0.25 mmol, 1.0 equiv) and 4-bromophenyl acetate (54 mg, 0.25 mmol, 1.0 equiv) at r.t. (20-22 °C). Purification of the crude reaction mixture using Purification Method A afforded the title product as a colorless oil, 1.3 mg (2% yield). Modification of this procedure using 5-methoxy-1,3-dioxoisoindolin-2-yl 3-methyl bicyclo[1.1.1]pentane-1,3-dicarboxylate (86.2 mg, 0.250 mmol, 1.0 equiv) afforded the title product (7 mg, 11% yield)

^{1}H NMR (500 MHz, $CDCl_3$) δ 7.24 – 7.18 (m, 2H), 7.04 – 6.99 (m, 2H), 3.71 (s, 3H), 2.31 (s, 6H), 2.29 (s, 3H).
^{13}C{^{1}H} NMR (126 MHz, $CDCl_3$) δ 170.6, 169.6, 149.6, 137.3, 127.2, 126.7, 121.4, 121.3, 53.5, 51.7, 41.4, 36.9, 21.1.
HRMS (ESI) m/z calculated for $C_{15}H_{17}O_4$ [M+H]+ 261.1121, found 261.1120.

methyl 3-(4-chlorophenyl)bicyclo[1.1.1]pentane-1-carboxylate (3ae)

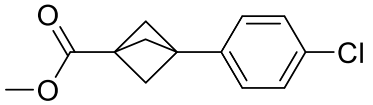

The title product was prepared according to General Procedure D at a 0.300 mmol scale using $NiBr_2$(dme) (18.5 mg, 0.060 mmol, 0.20 equiv), $^{t\text{-Bu}}$BpyCam^{CN} (20.1 mg, 0.060 mmol, 0.20 equiv), zinc dust (39.2 mg, 0.6 mmol, 2.0 equiv), and THF (0.450 mL)/DMA (0.050 mL),employing 1-(1,3-dioxoisoindolin-2-yl) 3-methyl bicyclo[1.1.1]pentane-1,3-dicarboxylate (94.6 mg, 0.30 mmol, 1.0 equiv), 1-chloro-4-iodobenzene (71.5 mg, 0.30 mmol, 1.0 equiv). Purification of the crude reaction mixture using Purification Method A afforded the title product (35.8 mg, 0.151 mmol, 50%) as a white solid. Analytical data was in accordance with that reported in the literature.[8]
^{1}H NMR (600 MHz, $CDCl_3$) δ 7.21 - 7.18 (m, 1H), 7.08 - 7.04 (m, 2H), 3.64 (s, 3H), 2.23 (s, 6H).
^{13}C{^{1}H} NMR (151 MHz, $CDCl_3$) δ 170.4, 138.1, 132.8, 128.4, 127.5, 53.4, 51.7, 41.3, 36.9.

Methyl 4-(4-methoxyphenyl)bicyclo[2.2.2]octane-1-carboxylate (3af)

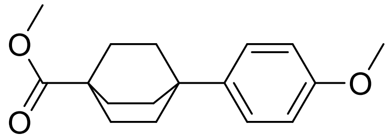

The title product was prepared according to General Procedure C at a 0.50 mmol scale using $NiBr_2$(dme) (10.8 mg, 0.035 mmol, 0.07 equiv), $^{t\text{-Bu}}$BpyCam^{CN} (11.8 mg, 0.035 mmol, 0.07 equiv), zinc flake (64.6 mg, 1.0 mmol, 2.0 equiv), and DMA (0.64 mL) employing 1-(1,3-dioxoisoindolin-2-yl) 4-methyl bicyclo[2.2.2]octane-1,4-dicarboxylate (179 mg, 0.50 mmol, 1.0 equiv) and 4-iodoanisole (116 mg, 0.50 mmol, 1.0 equiv) at r.t. (20-22 °C). Purification of the crude reaction mixture using Purification Method A afforded the title product as a white powder, 54 mg (39% yield). Characterization data matched those reported in the literature.[18]
^{1}H NMR (500 MHz, CDCl3) δ 7.25 – 7.19 (m, 2H), 6.87 – 6.80 (m, 2H), 3.78 (s, 3H), 3.66 (s, 3H), 1.91 (dd, J = 10.3, 4.9 Hz, 6H), 1.83 (dd, J = 10.4, 4.9 Hz, 6H).
^{13}C{^{1}H} NMR (126 MHz, $CDCl_3$) δ 178.47, 157.56, 141.33, 126.41, 113.49, 55.22, 51.68, 39.06, 33.96, 31.89, 28.84.
HRMS (ESI) m/z calculated for $C_{17}H_{23}O_3$ [M+H]+ 275.16417, found 275.1638.
MP = 115-116 °C.

methyl 4-(4-methoxyphenyl)bicyclo[2.1.1]hexane-1-carboxylate (3ag)

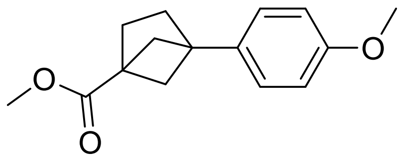

The title product was prepared according to General Procedure D at a 0.30 mmol scale using $NiBr_2$(dme) (18.5 mg, 0.060 mmol, 0.20 equiv), $^{t\text{-Bu}}$BpyCam^{CN} (20.1 mg, 0.060 mmol, 0.20 equiv), zinc dust (39.2 mg, 0.6 mmol, 2.0 equiv), and THF (0.450 mL)/DMA (0.050 mL), employing 1-(1,3-dioxoisoindolin-2-yl) 4-methyl bicyclo[2.1.1]hexane-1,4-dicarboxylate (98.8 mg, 0.30 mmol, 1.0 equiv) and 4-iodoanisole (70.2 mg, 0.30 mmol, 1.0 equiv). Purification of the crude reaction mixture using Purification Method B afforded the title product (16.1 mg, 65.4 µmol, 22%) as a colorless oil.

^{1}H NMR (600 MHz, $CDCl_3$) δ 7.21 - 7.15 (m, 2H), 6.91 - 6.84 (m, 2H), 3.79 (s, 3H), 3.72 (s, 3H), 2.11 - 2.04 (m, 4H), 2.02 - 1.96 (m, 2H), 1.84 - 1.77 (m, 2H).

^{13}C{^{1}H} NMR (151 MHz, $CDCl_3$) δ 174.0, 158.2, 135.0, 127.0, 113.7, 55.3, 51.5, 50.6, 48.9, 46.3, 33.5, 30.8.

HRMS (ESI) m/z calculated for $C_{15}H_{19}NO_3$ [M+H]+ 247.1329, found 247.1335.

FTIR (ATR, cm^{-1}) 2951, 1731, 1518, 1352, 1261, 1248, 1179, 1095.

3-(4-methoxyphenyl)-3-methyloxetane (3ah)

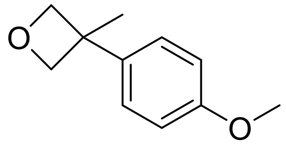

The title product was prepared according to General Procedure C at a 0.50 mmol scale using $NiBr_2$(dme) (10.8 mg, 0.035 mmol, 0.07 equiv), $^{t\text{-Bu}}$BpyCam^{CN} (11.8 mg, 0.035 mmol, 0.07 equiv), zinc flake (64.6 mg, 1.0 mmol, 2.0 equiv), and THF (0.64 mL) employing 1,3-dioxoisoindolin-2-yl 3-methyloxetane-3-carboxylate (130 mg, 0.5 mmol, 1.0 equiv) and 4-iodoanisole (116 mg, 0.5 mmol, 1.0 equiv) at r.t. (20-22 °C). Purification of the crude reaction mixture using Purification Method A afforded the title product as a colorless oil, 44 mg (49% yield). Characterization data matched those reported in the literature.[18]

Modification of this procedure using 5-methoxy-1,3-dioxoisoindolin-2-yl 3-methyloxetane-3-carboxylate (145 mg, 0.5 mmol, 1.0 equiv) afforded the title product as a colorless oil, 49 mg (55% yield)

^{1}H NMR (500 MHz, $CDCl_3$) δ 7.19 – 7.12 (m, 2H), 6.93 – 6.86 (m, 2H), 4.93 (d, J = 5.5 Hz, 2H), 4.61 (d, J = 5.5 Hz, 2H), 3.81 (s, 3H), 1.71 (s, 3H).

^{13}C{^{1}H} NMR (126 MHz, $CDCl_3$) δ 158.0, 138.6, 126.2, 113.9, 84.0, 65.9, 55.3, 42.8, 27.7, 15.3.

HRMS (ESI) m/z calculated for $C_{11}H_{15}O_2$ [M+H]+ 179.10666, found 179.1065

tert-butyl 3-(4-methoxyphenyl)-3-methylazetidine-1-carboxylate (3ai)

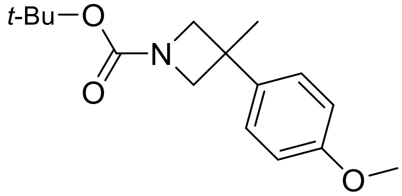

The title product was prepared according to General Procedure D at a 0.300 mmol scale using $NiBr_2$(dme) (18.5 mg, 0.060 mmol, 0.20 equiv), $^{t\text{-Bu}}$BpyCam^{CN} (20.1 mg, 0.060 mmol, 0.20 equiv),

zinc dust (39.2 mg, 0.6 mmol, 2.0 equiv), and THF (0.750 mL,employing 1-(tert-butyl) 3-(1,3-dioxoisoindolin-2-yl) 3-methylazetidine-1,3-dicarboxylate (108.1 mg, 0.30 mmol, 1.0 equiv) and 4-iodoanisole (70.2 mg, 0.30 mmol, 1.0 equiv). Purification of the crude reaction mixture using Purification Method A afforded the title product (50.1 mg, 0.181 mmol, 60%) as a colorless oil, which solidified on standing.
^{1}H NMR (600 MHz, $CDCl_3$) δ 7.17 - 7.11 (m, 2H), 6.90 - 6.86 (m, 2H), 4.16 (m, 2H), 3.90 (m, 2H), 3.80 (s, 3H), 1.60 (s, 3H), 1.46 (s, 9H).
^{13}C{^{1}H} NMR (151 MHz, $CDCl_3$) δ 157.9, 156.5, 138.9, 126.2, 113.8, 79.3, 61.8, 55.2, 37.1, 29.0, 28.3.
HRMS (ESI) m/z calculated for $C_{16}H_{23}NO_3Na$ [M+Na]+ 300.1570, found 300.1580.
FTIR (ATR, cm^{-1}) 1699, 1517, 1396, 1249, 1153, 913, 746.

ethyl 1-(4-methoxyphenyl)cyclobutane-1-carboxylate (3aj)

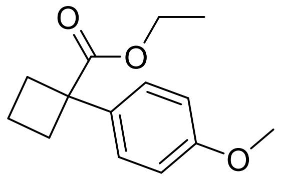

The title product was prepared according to General Procedure C at a 0.50 mmol scale using $NiBr_2$(dme) (10.8 mg, 0.035 mmol, 0.07 equiv), $^{t\text{-Bu}}$BpyCam^{CN} (11.8 mg, 0.035 mmol, 0.07 equiv), zinc flake (64.6 mg, 1.0 mmol, 2.0 equiv), and THF (0.64 mL) employing 1-(1,3-dioxoisoindolin-2-yl) 1-ethyl cyclobutane-1,1-dicarboxylate (159 mg, 0.5 mmol, 1.0 equiv)and 4-iodoanisole (116 mg, 0.5 mmol, 1.0 equiv) at r.t. (20-22 °C). Purification of the crude reaction mixture using Purification Method A afforded the title product as a colorless oil 45 mg (with 5% aryl dimer) (37% yield).
^{1}H NMR (400 MHz, $CDCl_3$) δ 7.27 – 7.20 (m, 2H), 6.89 – 6.82 (m, 2H), 4.09 (q, J = 7.1 Hz, 2H), 3.79 (s, 3H), 2.85 – 2.75 (m, 1H), 2.51 – 2.41 (m, 2H), 2.06 – 1.92 (m, 1H), 1.85 (dtt, J = 10.9, 9.1, 5.3 Hz, 1H), 1.17 (t, J = 7.1 Hz, 3H).
^{13}C{^{1}H} NMR (101 MHz, $CDCl_3$) δ 176.2, 158.2, 135.9, 127.4, 113.6, 60.8, 55.2, 51.7, 32.3, 16.5, 14.1.
HRMS (ESI) m/z calculated for $C_{14}H_{19}O_3$ [M+H]+ 235.1329, found 235.1325

tert-butyl 3-methyl-3-(1-phenyl-1H-pyrazol-3-yl)azetidine-1-carboxylate (3ak)

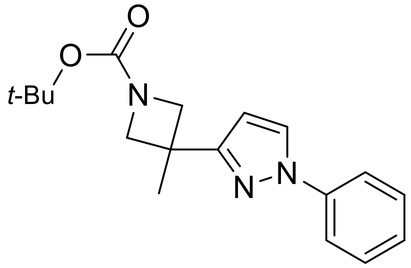

The title product was prepared according to General Procedure D at a 0.300 mmol scale using $NiBr_2$(dme) (18.5 mg, 0.060 mmol, 0.20 equiv), $^{t\text{-Bu}}$BpyCam^{CN} (20.1 mg, 0.060 mmol, 0.20 equiv), zinc dust (39.2 mg, 0.6 mmol, 2.0 equiv), and THF (0.810 mL)/DMA (0.090 mL),employing 1-(tert-butyl) 3-(1,3-dioxoisoindolin-2-yl) 3-methylazetidine-1,3-dicarboxylate (108.1 mg, 0.30 mmol, 1.0 equiv), 3-iodo-1-phenyl-1H-pyrazole (81.2 mg, 0.30 mmol, 1.0 equiv, 95% purity).

Purification of the crude reaction mixture using Purification Method A afforded the title product (66.5 mg, 0.212 mmol, 71%) as a colorless oil.
^{1}H NMR (600 MHz, $CDCl_3$) δ 7.90 - 7.86 (m, 1H), 7.73 - 7.67 (m, 2H), 7.49 - 7.43 (m, 2H), 7.31 - 7.26 (m, 1H), 6.38 (d, J = 2.4 Hz, 1H), 4.35 - 4.29 (m, 2H), 3.94 - 3.87 (m, 2H), 1.72 (s, 3H), 1.46 (s, 9H).
^{13}C{^{1}H} NMR (151 MHz, $CDCl_3$) δ 158.5, 156.7, 140.1, 129.4, 127.7, 126.2, 119.0, 104.5, 79.3, 61.4, 33.7, 28.4, 25.7.
HRMS (ESI) m/z calculated for $C_{18}H_{24}N_3O_2$ [M+H]+ 314.1863, found 314.1875.
FTIR (ATR, cm^{-1}) 2965, 1696, 1403, 1391, 1366, 1164, 1106, 913, 746.

Ethyl 4-(8-chloro-3-(1-phenylcyclopropyl)-5,6-dihydro-11H-benzo[5,6]cyclohepta[1,2-b]pyridin-11-ylidene)piperidine-1-carboxylate (3al)

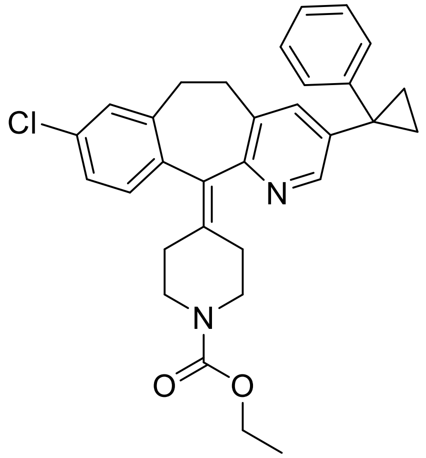

The title product was prepared according to General Procedure C at a 0.10 mmol scale using NiBr2(dme) (5.2 mg, 0.05 mmol, 0.20 equiv), ^{tbu}bpyCam^{CN} (5.8 mg, 0.0175 mmol, 0.2 equiv), zinc flake (13 mg, 0.2 mmol, 2.0 equiv), and THF (0.20 mL) employing 5-methyl-1,3-dioxoisoindolin-2-yl 1-phenylcyclopropane-1-carboxylate (32.1 mg, 0.10 mmol, 1.0 equiv) and and ethyl 4-(3 bromo-8-chloro-5,6-dihydro-11H-benzo[5,6]cyclohepta[1,2-b]pyridin-11-ylidene)piperidine-1-carboxylate (43 mg, 0.10 mmol, 1.0 equiv) at 40 °C. Purification of the crude reaction mixture using Purification Method A afforded the title product as a colorless oil, 29 mg (58% yield).
^{1}H NMR (500 MHz, $CDCl_3$) δ 8.26 (d, J = 2.2 Hz, 1H), 7.31 – 7.07 (m, 9H), 4.13 (q, J = 7.1 Hz, 2H), 3.79 (s, 2H), 3.41 – 3.21 (m, 2H), 3.13 (dddd, J = 13.1, 9.0, 4.1, 2.1 Hz, 2H), 2.83 – 2.71 (m, 2H), 2.54 – 2.44 (m, 1H), 2.32 (dt, J = 14.4, 5.0 Hz, 3H), 1.31 (s, 2H), 1.28 – 1.22 (m, 5H).
^{13}C{^{1}H} NMR (126 MHz, $CDCl_3$) δ 155.7, 146.6, 144.5, 139.9, 139.8, 137.8, 137.7, 134.1, 133.0, 132.7, 130.5, 129.0, 128.7, 128.6, 126.6, 126.3, 61.5, 45.0, 45.0, 31.8, 31.7, 30.7, 27.8, 15.9, 14.8.
HRMS (ESI) m/z calculated for $C_{31}H_{32}ClN_2O_2$ [M+H]+ 499.2147, found 499.2143

4.4 Preparation of ($^{t\text{-Bu}}$BpyCam^{CN})Ni(*o*-tol)

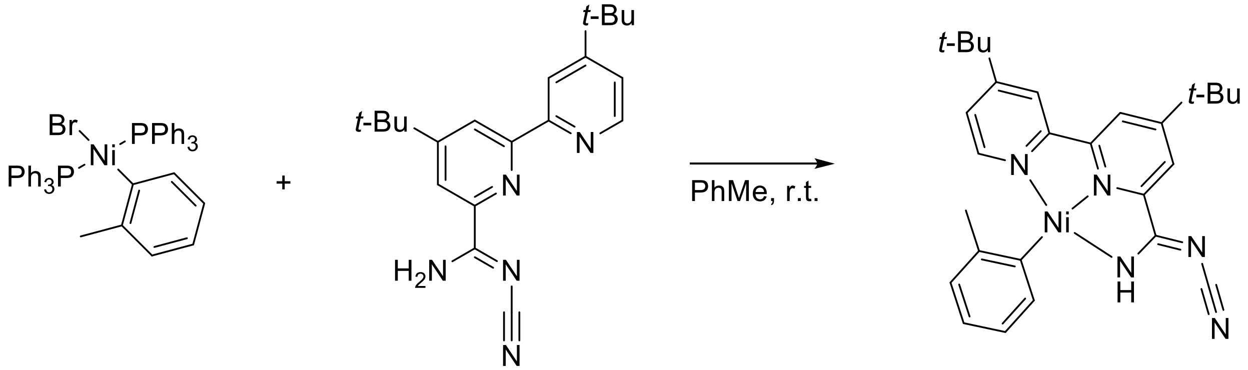

To a flame-dried 200 mL Schlenk flask equipped with a stir bar was charged 4,4′-di-tert-butyl-6-*N*-cyanocarboxamidine-2,2-bipyridine (120.8 mg, 0.36 mmol, 1.03 equiv) and dry toluene (100 mL). The mixture was heated gently, while stirring, using a heatgun until the solution was completely homogenous. The flask was then brought into a N_2-filled glovebox and *trans*-[$(PPh_3)_2$Ni(*o*-tol)]Br (264 mg, 0.35 mmol, 1.0 equiv) was added (*trans*-[$(PPh_3)_2$Ni(*o*-tol)]Br can also be added outside of a glovebox while maintaining positive nitrogen pressure in the reaction vessel). The flask was sealed, removed from the glovebox, and left to stir at room temperature (20-22 °C) for 26 h. After this time, the mixture was concentrated on a Schlenk line using an external solvent trap to approximately half its original volume. The flask then brought into a N_2-filled glovebox, and the mixture was filtered to afford an orange solid, which was subsequently washed multiple times with dry toluene. Pentane was added to the filtrate solution to afford the product as an orange solid. The product was washed several times with pentane to afford 162 mg of an impure orange solid, which was subsequently purified by layered recrystallization, carefully layering pentane onto a solution of the complex in DCM.

^{1}H NMR (500 MHz, CD_2Cl_2) δ 7.90 (d, *J* = 1.5 Hz, 1H), 7.88 (d, *J* = 1.5 Hz, 1H), 7.85 (d, *J* = 2.1 Hz, 1H), 7.62 – 7.56 (m, 1H), 7.51 (d, *J* = 6.0 Hz, 1H), 7.24 (dd, *J* = 6.0, 2.1 Hz, 1H), 6.97 – 6.92 (m, 1H), 6.91 – 6.86 (m, 2H), 2.87 (s, 3H), 1.46 (s, 9H), 1.37 (s, 9H).

^{13}C{^{1}H} NMR (126 MHz CD_2Cl_2) δ 166.3, 164.6, 156.0, 154.8, 153.1, 151.2, 144.5, 136.6, 127.8, 124.7, 123.9, 123.8, 120.3, 118.9, 118.1, 53.8, 36.8, 36.0, 30.7, 30.3, 25.6.

4.5 Crystallographic Data

Data Collection

A red crystal with approximate dimensions 0.129 × 0.028 × 0.028 mm^3 was selected under oil under ambient conditions and attached to the tip of a MiTeGen MicroMount©. The crystal was mounted in a stream of cold nitrogen at 100(1) K and centered in the X-ray beam by using a video camera.

The crystal evaluation and data collection were performed on a Bruker D8 VENTURE PhotonIII four-circle diffractometer with Cu Kα (λ = 1.54178 Å) radiation and the detector to crystal distance of 5.0 cm.[22]

The initial cell constants were obtained from a 180° φ scan conducted at a 2θ = 50° angle with the exposure time of 1 second per frame. The reflections were successfully indexed by an automated indexing routine built in the APEX3 program. The final cell constants were calculated from a set of 9840 strong reflections from the actual data collection.

The data were collected by using the data collection routine to survey the necessary portion of the reciprocal space to a resolution of 0.81 Å. A total of 126026 data were harvested by collecting 17 sets of frames with 0.9-1.0° scans in ω and φ with an exposure time 1–30 sec per frame. These highly redundant datasets were corrected for Lorentz and polarization effects. The absorption correction was based on fitting a function to the empirical transmission surface as sampled by multiple equivalent measurements.[23]

Structure Solution and Refinement

The systematic absences in the diffraction data were uniquely consistent for the space group $P2_1/c$ that yielded chemically reasonable and computationally stable results of refinement.[24-29]

A successful solution by intrinsic phasing provided most non-hydrogen atoms from the *E*-map. The remaining non-hydrogen atoms were located in an alternating series of least-squares cycles and difference Fourier maps. All non-hydrogen atoms were refined with anisotropic displacement coefficients. All hydrogen atoms were included in the structure factor calculation at idealized positions and were allowed to ride on the neighboring atoms with relative isotropic displacement coefficients.

The asymmetric unit contains two symmetry-independent Ni complexes and several unidentified solvent molecules.

The two Ni complexes have identical composition but minor geometrical differences (Figure 3). In each complex the tolyl ligand is disordered over two positions. The major disorder component has occupancy of 0.747(7) in the Ni1 complex and 0.766(6) in the Ni1A complex. The minor disorder components were refined with restraints.

There are several solvent-accessible voids (total volume ~ 1102 Å^3) in the unit cell. They contained two or more types of partially occupied solvent molecules. A significant amount of time was invested in identifying and refining the disordered molecules. Bond length restraints were applied to model the molecules but the resulting isotropic displacement coefficients suggested the molecules were mobile. In addition, the refinement was computationally unstable. Option Solvent Mask of program OLEX2[28] was used to correct the diffraction data for diffuse scattering effects and to identify the solvent molecules. Solvent Mask calculated 250 electrons in the unit cell for the diffuse species. The compound was crystallized from DCM, heptane, pentane and toluene, thus it is difficult to determine the composition of the solvent mixture is in the voids. Please note that all derived results in the following tables are based on the known contents. No data are given for the diffusely scattering species.

The final least-squares refinement of 739 parameters against 11647 data resulted in residuals R (based on F^2 for $I \geq 2\sigma$) and wR (based on F^2 for all data) of 0.0624 and 0.1735, respectively. The final difference Fourier map was featureless.

Summary

Crystal Data for $C_{27}H_{31}N_5Ni$ (M =484.28 g/mol): monoclinic, space group P2$_1$/c (no. 14), a = 24.446(3) Å, b = 11.886(2) Å, c = 21.571(3) Å, β = 114.747(7)°, V = 5692.3(15) Å^3, Z = 8, T =

100.00 K, μ(Cu Kα) = 1.135 mm^{-1}, *Dcalc* = 1.130 g/cm^3, 126026 reflections measured (3.98° ≤ 2Θ ≤ 149.478°), 11647 unique (R_{int} = 0.0879, R_{sigma} = 0.0377) which were used in all calculations. The final R_1 was 0.0624 (I > 2σ(I)) and wR_2 was 0.1735 (all data).

Acknowledgement

The purchase of the Bruker D8 VENTURE Photon III X-ray diffractometer was partially funded by NSF Award #CHE-1919350 to the UW–Madison Department of Chemistry.

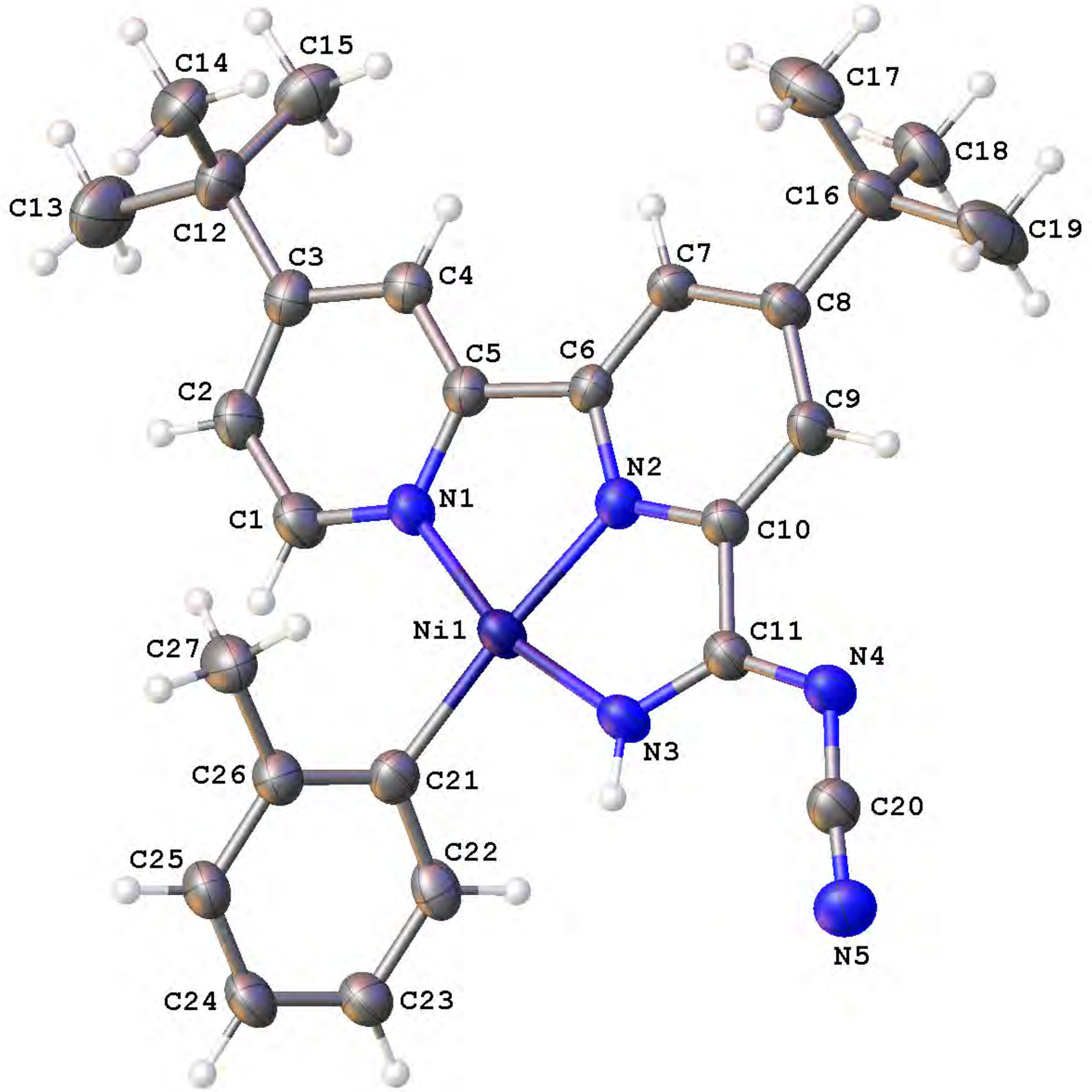

Figure S8. A molecular drawing of the first symmetry-independent complex in Weix10 shown with 50% probability ellipsoids. All H atoms are shown but the minor disorder components are omitted.

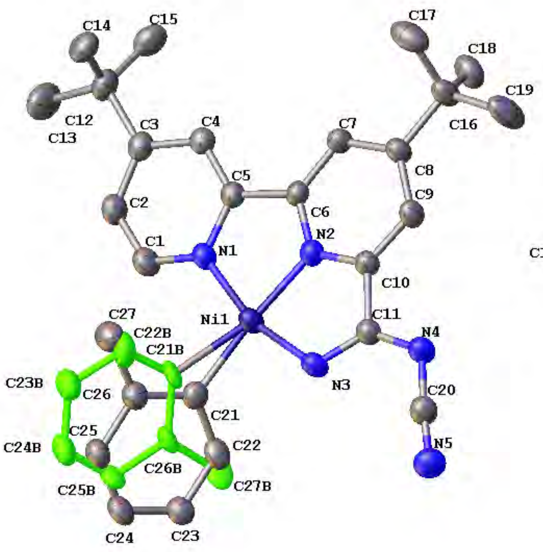

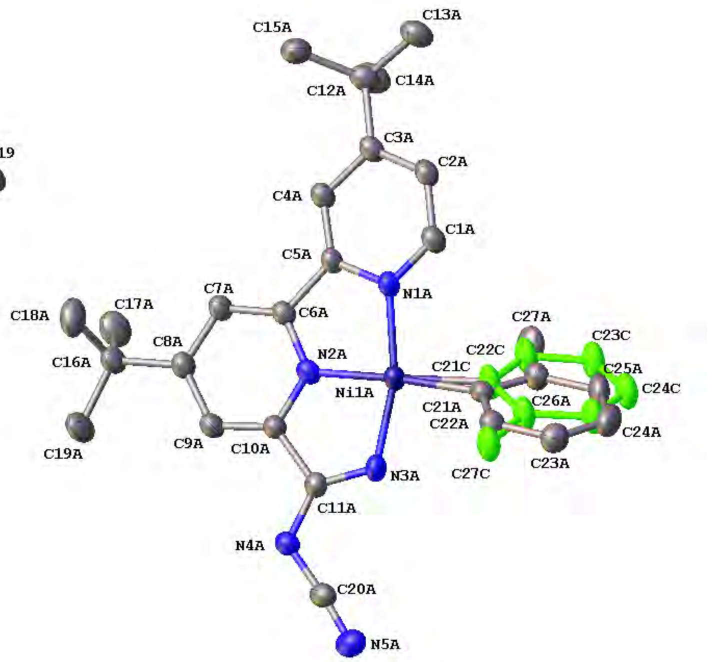

Figure S9. A molecular drawing of Weix10 shown with 50% probability ellipsoids. All H atoms are omitted but the minor disorder components are shown in green.

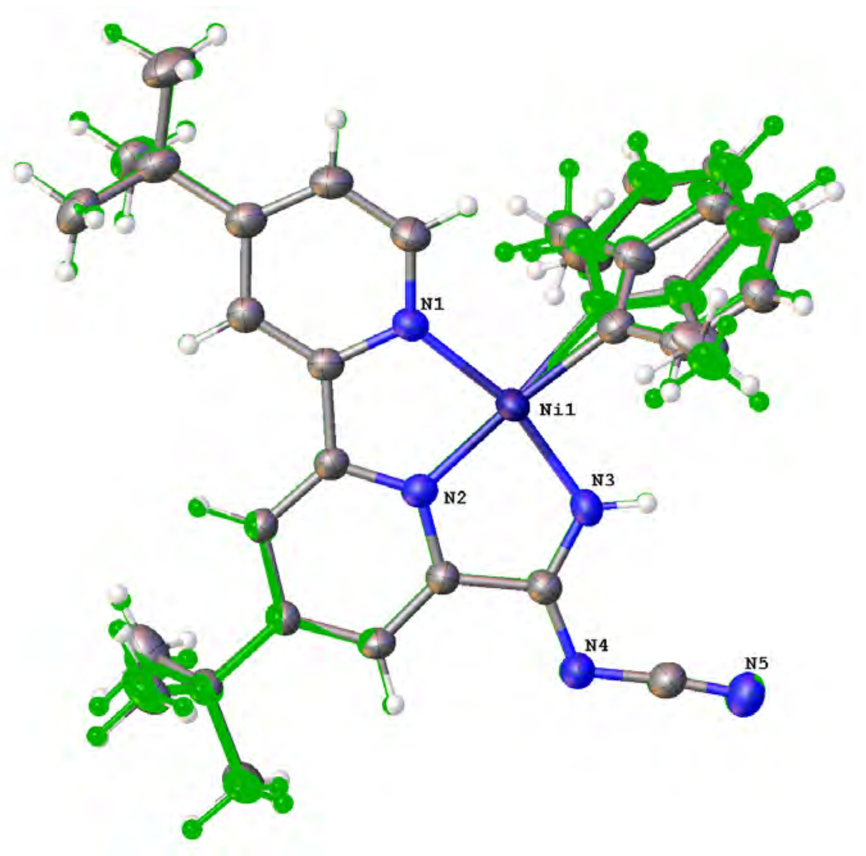

Figure S10. A superposition of the two Ni complexes shown with 50% probability ellipsoids. The Ni1A complex is shown in green.

Table 1 Crystal data and structure refinement for weix10.

Identification code	weix10
Empirical formula	$C_{27}H_{31}N_5Ni$·solvent
Formula weight	484.28
Temperature/K	100.00
Crystal system	monoclinic
Space group	$P2_1/c$
a/Å	24.446(3)
b/Å	11.886(2)
c/Å	21.571(3)
α/°	90
β/°	114.747(7)
γ/°	90
Volume/Å^3	5692.3(15)
Z	8
ρ_{calc}g/cm^3	1.130
μ/mm^{-1}	1.135
F(000)	2048.0
Crystal size/mm^3	0.129 × 0.028 × 0.028
Radiation	Cu Kα (λ = 1.54178)
2Θ range for data collection/°	3.98 to 149.478
Index ranges	$-30 \le h \le 30$, $-13 \le k \le 14$, $-26 \le l \le 26$
Reflections collected	126026
Independent reflections	11647 [R_{int} = 0.0879, R_{sigma} = 0.0377]
Data/restraints/parameters	11647/243/739
Goodness-of-fit on F^2	1.059
Final R indexes [I>=2σ (I)]	R_1 = 0.0624, wR_2 = 0.1654
Final R indexes [all data]	R_1 = 0.0744, wR_2 = 0.1735
Largest diff. peak/hole / e Å^{-3}	0.41/-0.67

Table 2 Fractional Atomic Coordinates (×10^4) and Equivalent Isotropic Displacement Parameters (Å^2×10^3) for weix10. U_{eq} is defined as 1/3 of the trace of the orthogonalised U_{IJ} tensor.

Atom	*x*	*y*	*z*	U(eq)
Ni1	10429.7(2)	1461.5(5)	4457.9(2)	36.50(14)
N1	10660.0(10)	1140(2)	5406.0(12)	36.1(5)
N2	9698.0(10)	1790(2)	4487.2(12)	34.1(5)
N3	9999.3(11)	1859(2)	3539.3(12)	38.3(6)
N4	9009.7(11)	2451(3)	2729.5(12)	41.3(6)
N5	9269.0(13)	2504(3)	1736.8(14)	47.5(7)

Table 2 Fractional Atomic Coordinates (×10^4) and Equivalent Isotropic Displacement Parameters (Å^2×10^3) for weix10. U_{eq} is defined as 1/3 of the trace of the orthogonalised U_{IJ} tensor.

**Atom**	***x***	***y***	***z***	**U(eq)**
C1	11189.8(14)	757(3)	5876.0(16)	42.9(7)
C2	11316.8(14)	662(3)	6563.4(16)	44.2(7)
C3	10899.1(13)	997(3)	6809.9(15)	39.6(7)
C4	10337.6(13)	1345(3)	6317.1(14)	35.3(6)
C5	10227.6(12)	1402(3)	5638.9(14)	33.5(6)
C6	9652.4(12)	1777(3)	5084.0(13)	32.5(6)
C7	9124.5(13)	2102(3)	5125.9(14)	35.5(6)
C8	8631.6(13)	2463(3)	4527.3(14)	36.7(6)
C9	8700.1(13)	2473(3)	3912.0(14)	38.5(7)
C10	9239.8(12)	2142(3)	3913.2(14)	33.9(6)
C11	9426.6(12)	2152(3)	3339.7(14)	35.7(6)
C12	11029.3(15)	1067(3)	7561.6(16)	47.4(8)
C13	11622(2)	476(5)	8010.9(19)	71.3(13)
C14	11075.6(17)	2327(4)	7748.6(18)	54.1(9)
C15	10520.1(17)	534(3)	7704.8(16)	52.1(8)
C16	8045.6(13)	2808(3)	4571.2(15)	43.4(7)
C17	8176.6(18)	3744(4)	5093(2)	61.2(10)
C18	7799.8(15)	1766(4)	4795(2)	53.3(9)
C19	7566.5(18)	3188(5)	3879(2)	71.5(14)
C20	9168.3(14)	2474(3)	2218.1(15)	40.2(7)
C21	11141.9(18)	1147(4)	4338(2)	37.1(10)
C22	11139(3)	328(7)	3878(4)	37.5(15)
C23	11628.1(18)	128(4)	3730(2)	41.6(10)
C24	12133.6(18)	795(5)	4031(3)	44.1(11)
C25	12152(2)	1621(6)	4483(3)	43.8(14)
C26	11655(2)	1812(4)	4641(2)	41.6(11)
C27	11685(3)	2772(8)	5102(5)	51.5(19)
C21B	11268(4)	1697(9)	4557(6)	38(3)
C22B	11601(8)	2610(20)	4923(17)	63(8)
C23B	12181(4)	2826(10)	5003(7)	48(3)
C24B	12422(4)	2176(10)	4649(6)	46(3)
C25B	12098(4)	1288(12)	4258(8)	34(4)
C26B	11515(4)	1048(8)	4200(5)	30(3)
C27B	11184(9)	57(19)	3787(17)	48(7)
Ni1A	4599.7(2)	3398.4(4)	-868.2(2)	32.27(14)
N1A	4365.3(10)	3869(2)	-163.5(11)	31.5(5)
N2A	5335.3(10)	3166(2)	-123.3(12)	33.4(5)
N3A	5034.8(10)	2890(2)	-1351.3(12)	36.5(5)

Table 2 Fractional Atomic Coordinates (×10⁴) and Equivalent Isotropic Displacement Parameters (Å²×10³) for weix10. U_{eq} is defined as 1/3 of the trace of the orthogonalised U_{IJ} tensor.

Atom	*x*	*y*	*z*	U(eq)
N4A	6038.2(10)	2322(2)	-1183.2(12)	38.9(6)
N5A	5781.0(13)	2065(3)	-2414.6(15)	53.9(8)
C1A	3836.4(12)	4283(3)	-220.8(15)	35.6(6)
C2A	3699.5(12)	4474(3)	331.2(14)	36.5(6)
C3A	4113.6(12)	4221(3)	992.4(14)	34.9(6)
C4A	4684.0(12)	3843(3)	1060.4(14)	35.1(6)
C5A	4796.8(11)	3697(3)	491.9(13)	30.8(6)
C6A	5377.7(12)	3307(3)	509.2(14)	31.8(6)
C7A	5918.5(12)	3093(3)	1071.7(14)	34.6(6)
C8A	6418.2(12)	2720(3)	963.5(14)	34.5(6)
C9A	6348.6(12)	2564(3)	291.7(14)	35.8(6)
C10A	5798.9(12)	2795(3)	-240.6(13)	32.7(6)
C11A	5610.3(12)	2656(3)	-989.0(14)	34.7(6)
C12A	3970.0(14)	4261(3)	1615.6(16)	42.5(7)
C13A	3373.9(16)	4850(4)	1460.8(18)	54.2(9)
C14A	3935.2(18)	3049(4)	1829(2)	57.4(9)
C15A	4474.3(15)	4876(4)	2214.3(16)	51.2(9)
C16A	7020.5(13)	2506(3)	1577.0(14)	40.1(7)
C17A	6933.3(16)	1570(4)	2020.8(19)	54.6(9)
C18A	7224.5(16)	3589(4)	1988.0(19)	56.8(9)
C19A	7512.8(15)	2139(4)	1355.3(17)	59.5(11)
C20A	5880.7(13)	2189(3)	-1842.6(16)	41.7(7)
C21A	3875.2(18)	3579(4)	-1668(2)	34.3(10)
C22A	3858.5(19)	4332(4)	-2170(2)	38.3(10)
C23A	3354.2(19)	4462(4)	-2785(2)	46.5(11)
C24A	2843(2)	3834(5)	-2910(3)	53.1(14)
C25A	2857(2)	3073(5)	-2424(2)	50.8(13)
C26A	3365.2(17)	2917(4)	-1809(2)	39.3(10)
C27A	3349(2)	2026(5)	-1317(3)	54.8(13)
C21C	3781(6)	3299(18)	-1588(7)	48(3)
C22C	3406(5)	2525(14)	-1477(6)	50(2)
C23C	2838(5)	2269(13)	-1963(6)	52(2)
C24C	2624(5)	2822(14)	-2582(6)	51(2)
C25C	2977(5)	3609(16)	-2708(7)	49(2)
C26C	3564(4)	3836(12)	-2219(5)	48(2)
C27C	3943(6)	4664(14)	-2385(7)	49(3)

Table 3 Anisotropic Displacement Parameters (Å^2×10^3) for weix10. The Anisotropic displacement factor exponent takes the form: $-2\pi^2[h^2a^{*2}U_{11}+2hka^*b^*U_{12}+…]$.

Atom	U_{11}	U_{22}	U_{33}	U_{23}	U_{13}	U_{12}
Ni1	25.2(2)	55.4(3)	30.0(3)	5.1(2)	12.59(19)	7.1(2)
N1	24.8(11)	51.1(15)	31.0(12)	1.3(10)	10.4(9)	4.9(10)
N2	26.9(11)	46.2(14)	26.9(11)	1.2(10)	9.1(9)	1.0(10)
N3	29.1(12)	57.7(16)	32.7(12)	4.3(11)	17.6(10)	5.3(11)
N4	30.9(12)	64.7(17)	29.8(12)	4.5(11)	14.1(10)	5.0(11)
N5	47.6(15)	62.4(18)	36.4(13)	5.6(12)	21.2(12)	-0.4(13)
C1	30.1(14)	59(2)	37.5(15)	4.7(14)	12.3(12)	11.4(13)
C2	29.9(14)	61(2)	35.0(15)	2.7(14)	6.6(12)	8.1(13)
C3	32.4(14)	49.0(18)	32.0(14)	-2.8(12)	8.2(12)	0.9(12)
C4	30.3(14)	43.3(16)	31.1(14)	-2.0(11)	11.7(11)	-0.3(11)
C5	26.8(13)	42.8(16)	28.6(13)	-1.1(11)	9.2(11)	1.7(11)
C6	26.0(13)	45.2(16)	23.9(12)	-3.4(11)	8.0(10)	-2.3(11)
C7	29.3(13)	53.1(18)	24.5(12)	2.3(12)	11.7(11)	4.2(12)
C8	27.1(13)	55.0(18)	29.9(13)	3.8(12)	14.0(11)	5.6(12)
C9	26.6(13)	58.1(19)	27.3(13)	6.7(12)	7.9(11)	7.1(12)
C10	26.8(13)	48.9(17)	26.4(13)	2.3(11)	11.5(10)	1.7(11)
C11	27.3(13)	50.7(17)	29.5(13)	1.5(12)	12.2(11)	2.5(12)
C12	36.7(16)	67(2)	29.0(14)	-2.0(14)	4.3(12)	3.1(15)
C13	60(2)	104(4)	34.5(18)	8(2)	4.2(16)	25(2)
C14	46.9(19)	74(3)	37.4(16)	-17.0(16)	13.7(14)	-8.6(17)
C15	59(2)	65(2)	29.6(15)	2.1(14)	15.9(14)	-0.2(17)
C16	30.5(14)	72(2)	31.4(14)	12.8(14)	16.5(12)	13.7(14)
C17	52(2)	66(2)	80(3)	-4(2)	42(2)	6.5(18)
C18	32.4(16)	73(3)	57(2)	2.6(18)	21.2(15)	-1.1(15)
C19	42.6(19)	126(4)	51(2)	32(2)	25.4(17)	39(2)
C20	33.5(14)	54.6(19)	31.5(14)	7.1(13)	12.4(12)	3.9(13)
C21	32(2)	50(3)	27(2)	5.1(17)	9.7(17)	2.8(19)
C22	27(3)	40(4)	41(3)	9(3)	10(2)	7(3)
C23	33(2)	54(3)	39(2)	0.2(18)	15.1(17)	6.4(17)
C24	26(2)	61(3)	45(3)	2(2)	14.1(18)	9(2)
C25	26(2)	62(5)	40(4)	-4(3)	12(2)	-2(2)
C26	30(2)	54(3)	38(2)	-3.2(19)	11.2(17)	4.6(18)
C27	39(3)	67(4)	50(5)	-14(3)	19(4)	-7(3)
C21B	11(6)	62(9)	38(7)	-1(6)	9(5)	2(5)
C22B	27(8)	100(16)	50(16)	-11(10)	5(8)	-4(8)
C23B	27(6)	64(8)	51(7)	-13(6)	13(5)	-1(5)
C24B	22(6)	63(9)	51(7)	-8(6)	11(5)	0(6)
C25B	22(6)	40(10)	41(11)	-6(7)	14(6)	11(5)
C26B	14(4)	45(6)	33(5)	7(5)	10(4)	2(4)

Table 3 Anisotropic Displacement Parameters (Å^2×10^3) for weix10. The Anisotropic displacement factor exponent takes the form: -2π^2[h^{2}a*^{2}U$_{11}$+2hka*b*U$_{12}$+…].

Atom	U$_{11}$	U$_{22}$	U$_{33}$	U$_{23}$	U$_{13}$	U$_{12}$
C27B	31(9)	29(9)	74(13)	-7(7)	13(7)	13(6)
Ni1A	20.0(2)	51.3(3)	23.1(2)	0.62(19)	6.65(18)	0.84(19)
N1A	20.1(10)	47.0(14)	24.2(10)	2.9(9)	6.2(8)	2.0(9)
N2A	23.9(11)	48.4(14)	29.4(11)	1.8(10)	12.7(9)	1.5(9)
N3A	22.1(11)	58.7(16)	23.7(11)	-2.2(10)	4.8(9)	-0.4(10)
N4A	25.4(11)	62.7(17)	29.9(12)	-5.9(11)	12.8(9)	0.9(11)
N5A	44.3(15)	82(2)	39.5(15)	-16.9(14)	21.3(12)	-10.7(15)
C1A	23.7(12)	50.4(17)	32.1(13)	5.1(12)	11.1(11)	5.0(11)
C2A	22.7(12)	53.2(18)	33.3(14)	2.3(12)	11.3(11)	4.1(11)
C3A	24.6(12)	49.2(17)	32.2(14)	5.8(12)	13.1(11)	4.7(11)
C4A	24.1(12)	52.8(17)	28.8(13)	4.9(12)	11.5(10)	4.4(12)
C5A	19.8(12)	46.4(16)	24.5(12)	5.9(11)	7.6(10)	3.9(10)
C6A	23.5(12)	45.6(16)	27.1(13)	3.1(11)	11.4(10)	4.5(11)
C7A	24.7(12)	52.4(17)	25.4(12)	6.0(12)	9.3(10)	6.6(11)
C8A	24.9(13)	49.9(17)	27.0(13)	5.1(11)	9.2(11)	4.1(11)
C9A	24.6(13)	54.0(18)	30.2(13)	1.0(12)	12.9(11)	3.6(12)
C10A	23.5(12)	50.9(17)	24.1(12)	-2.0(11)	10.3(10)	0.1(11)
C11A	24.2(12)	50.9(17)	27.2(13)	-3.4(12)	8.9(10)	-0.8(11)
C12A	35.6(15)	62(2)	36.0(15)	5.5(14)	21.1(13)	5.7(14)
C13A	40.2(17)	84(3)	46.8(18)	1.2(17)	26.5(15)	9.9(17)
C14A	56(2)	75(3)	56(2)	12.6(19)	37.6(18)	3.9(19)
C15A	41.3(17)	81(3)	34.8(15)	-4.5(16)	19.2(14)	7.2(16)
C16A	26.4(13)	64(2)	28.7(13)	8.5(13)	10.1(11)	11.6(13)
C17A	40.8(18)	71(2)	48.0(19)	19.8(17)	15.0(15)	14.6(16)
C18A	34.4(17)	71(3)	47.7(19)	2.1(17)	0.0(14)	7.1(16)
C19A	30.4(16)	107(3)	37.2(16)	6.3(18)	10.3(13)	24.2(18)
C20A	30.5(14)	62(2)	36.5(16)	-11.6(14)	17.6(12)	-5.0(13)
C21A	23.1(19)	54(3)	27.4(19)	-7.6(16)	12.0(15)	-0.1(16)
C22A	27(2)	53(3)	33(2)	-0.9(17)	10.7(16)	5.9(17)
C23A	38(2)	64(3)	33(2)	0.6(18)	10.5(17)	7.2(19)
C24A	34(2)	77(4)	33(2)	-7(2)	-0.9(18)	13(2)
C25A	23(2)	75(3)	44(3)	-13(2)	3.2(18)	-4(2)
C26A	27.4(19)	58(3)	31(2)	-0.5(18)	11.3(16)	-3.0(17)
C27A	42(2)	67(3)	48(3)	2(2)	11(2)	-19(2)
C21C	22(4)	76(5)	41(4)	3(3)	9(3)	-12(3)
C22C	23(3)	78(5)	42(4)	3(4)	8(3)	-14(3)
C23C	26(3)	79(5)	44(4)	4(3)	7(3)	-15(3)
C24C	24(4)	78(5)	43(4)	3(3)	8(3)	-13(3)
C25C	24(3)	77(5)	42(4)	2(3)	9(3)	-12(3)

Table 3 Anisotropic Displacement Parameters (Å^2×10^3) for weix10. The Anisotropic displacement factor exponent takes the form: $-2\pi^2[h^2a^{*2}U_{11}+2hka^*b^*U_{12}+\ldots]$.

Atom	U_{11}	U_{22}	U_{33}	U_{23}	U_{13}	U_{12}
C26C	22(3)	75(5)	40(4)	2(3)	9(3)	-11(3)
C27C	26(4)	74(6)	41(5)	1(4)	9(4)	-12(4)

Table 4 Bond Lengths for weix10.

Atom	Atom	Length/Å	Atom	Atom	Length/Å
Ni1	N1	1.919(2)	Ni1A	N1A	1.919(2)
Ni1	N2	1.857(2)	Ni1A	N2A	1.863(2)
Ni1	N3	1.874(2)	Ni1A	N3A	1.874(2)
Ni1	C21	1.901(4)	Ni1A	C21A	1.898(4)
Ni1	C21B	1.990(8)	Ni1A	C21C	1.958(9)
N1	C1	1.347(4)	N1A	C1A	1.340(4)
N1	C5	1.382(4)	N1A	C5A	1.380(3)
N2	C6	1.338(4)	N2A	C6A	1.335(4)
N2	C10	1.342(4)	N2A	C10A	1.335(4)
N3	C11	1.328(4)	N3A	C11A	1.322(4)
N4	C11	1.332(4)	N4A	C11A	1.339(4)
N4	C20	1.314(4)	N4A	C20A	1.318(4)
N5	C20	1.163(4)	N5A	C20A	1.163(4)
C1	C2	1.387(4)	C1A	C2A	1.384(4)
C2	C3	1.392(4)	C2A	C3A	1.393(4)
C3	C4	1.401(4)	C3A	C4A	1.413(4)
C3	C12	1.518(4)	C3A	C12A	1.526(4)
C4	C5	1.374(4)	C4A	C5A	1.376(4)
C5	C6	1.483(4)	C5A	C6A	1.479(4)
C6	C7	1.386(4)	C6A	C7A	1.393(4)
C7	C8	1.414(4)	C7A	C8A	1.408(4)
C8	C9	1.406(4)	C8A	C9A	1.399(4)
C8	C16	1.531(4)	C8A	C16A	1.534(4)
C9	C10	1.376(4)	C9A	C10A	1.381(4)
C10	C11	1.487(4)	C10A	C11A	1.491(4)
C12	C13	1.535(5)	C12A	C13A	1.523(4)
C12	C14	1.543(6)	C12A	C14A	1.526(5)
C12	C15	1.539(5)	C12A	C15A	1.545(5)
C16	C17	1.519(6)	C16A	C17A	1.540(5)
C16	C18	1.539(5)	C16A	C18A	1.523(5)
C16	C19	1.531(4)	C16A	C19A	1.533(4)
C21	C22	1.389(9)	C21A	C22A	1.393(6)
C21	C26	1.393(6)	C21A	C26A	1.396(6)
C22	C23	1.380(8)	C22A	C23A	1.390(6)

Table 4 Bond Lengths for weix10.

Atom	Atom	Length/Å	Atom	Atom	Length/Å
C23	C24	1.380(6)	C23A	C24A	1.381(7)
C24	C25	1.372(7)	C24A	C25A	1.373(8)
C25	C26	1.411(6)	C25A	C26A	1.400(6)
C26	C27	1.495(8)	C26A	C27A	1.510(7)
C21B	C22B	1.389(10)	C21C	C22C	1.389(9)
C21B	C26B	1.392(7)	C21C	C26C	1.392(7)
C22B	C23B	1.379(9)	C22C	C23C	1.379(9)
C23B	C24B	1.379(7)	C23C	C24C	1.378(7)
C24B	C25B	1.375(8)	C24C	C25C	1.374(8)
C25B	C26B	1.408(7)	C25C	C26C	1.406(7)
C26B	C27B	1.494(9)	C26C	C27C	1.495(8)

Table 5 Bond Angles for weix10.

Atom	Atom	Atom	Angle/°	Atom	Atom	Atom	Angle/°
N1	Ni1	C21B	94.8(4)	N1A	Ni1A	C21C	95.8(7)
N2	Ni1	N1	82.10(10)	N2A	Ni1A	N1A	81.98(10)
N2	Ni1	N3	81.99(10)	N2A	Ni1A	N3A	82.20(10)
N2	Ni1	C21	174.63(14)	N2A	Ni1A	C21A	175.66(16)
N2	Ni1	C21B	158.5(3)	N2A	Ni1A	C21C	166.5(4)
N3	Ni1	N1	164.09(10)	N3A	Ni1A	N1A	164.17(10)
N3	Ni1	C21	92.67(14)	N3A	Ni1A	C21A	93.52(15)
N3	Ni1	C21B	100.1(4)	N3A	Ni1A	C21C	99.8(7)
C21	Ni1	N1	103.24(14)	C21A	Ni1A	N1A	102.30(15)
C1	N1	Ni1	129.1(2)	C1A	N1A	Ni1A	129.10(19)
C1	N1	C5	116.3(2)	C1A	N1A	C5A	116.3(2)
C5	N1	Ni1	114.50(18)	C5A	N1A	Ni1A	114.48(18)
C6	N2	Ni1	119.97(19)	C6A	N2A	Ni1A	119.79(18)
C6	N2	C10	121.2(2)	C6A	N2A	C10A	121.7(2)
C10	N2	Ni1	118.53(19)	C10A	N2A	Ni1A	118.35(19)
C11	N3	Ni1	117.27(19)	C11A	N3A	Ni1A	116.78(19)
C20	N4	C11	117.5(3)	C20A	N4A	C11A	117.4(2)
N1	C1	C2	123.1(3)	N1A	C1A	C2A	123.5(3)
C1	C2	C3	120.9(3)	C1A	C2A	C3A	120.8(3)
C2	C3	C4	115.9(3)	C2A	C3A	C4A	115.9(3)
C2	C3	C12	124.3(3)	C2A	C3A	C12A	124.0(3)
C4	C3	C12	119.7(3)	C4A	C3A	C12A	120.0(2)
C5	C4	C3	121.0(3)	C5A	C4A	C3A	120.3(2)
N1	C5	C6	112.7(2)	N1A	C5A	C6A	112.7(2)
C4	C5	N1	122.5(3)	C4A	C5A	N1A	122.8(2)
C4	C5	C6	124.8(3)	C4A	C5A	C6A	124.4(2)

Table 5 Bond Angles for weix10.

Atom	Atom	Atom	Angle/°	Atom	Atom	Atom	Angle/°
N2	C6	C5	110.3(2)	N2A	C6A	C5A	110.5(2)
N2	C6	C7	120.9(3)	N2A	C6A	C7A	120.4(2)
C7	C6	C5	128.7(2)	C7A	C6A	C5A	129.0(2)
C6	C7	C8	119.1(2)	C6A	C7A	C8A	119.1(2)
C7	C8	C16	119.1(2)	C7A	C8A	C16A	119.7(2)
C9	C8	C7	118.2(3)	C9A	C8A	C7A	118.4(2)
C9	C8	C16	122.7(2)	C9A	C8A	C16A	121.9(2)
C10	C9	C8	119.3(3)	C10A	C9A	C8A	119.3(3)
N2	C10	C9	121.4(2)	N2A	C10A	C9A	121.1(2)
N2	C10	C11	110.0(2)	N2A	C10A	C11A	109.9(2)
C9	C10	C11	128.6(3)	C9A	C10A	C11A	129.1(3)
N3	C11	N4	131.1(3)	N3A	C11A	N4A	130.9(3)
N3	C11	C10	112.2(2)	N3A	C11A	C10A	112.8(2)
N4	C11	C10	116.7(2)	N4A	C11A	C10A	116.3(2)
C3	C12	C13	111.7(3)	C3A	C12A	C14A	107.4(3)
C3	C12	C14	106.9(3)	C3A	C12A	C15A	110.7(3)
C3	C12	C15	111.5(3)	C13A	C12A	C3A	111.9(3)
C13	C12	C14	109.4(3)	C13A	C12A	C14A	109.4(3)
C13	C12	C15	108.3(3)	C13A	C12A	C15A	108.7(3)
C15	C12	C14	108.9(3)	C14A	C12A	C15A	108.7(3)
C8	C16	C18	107.6(3)	C8A	C16A	C17A	108.9(3)
C17	C16	C8	109.3(3)	C18A	C16A	C8A	108.9(3)
C17	C16	C18	110.2(3)	C18A	C16A	C17A	110.2(3)
C17	C16	C19	110.2(4)	C18A	C16A	C19A	108.3(3)
C19	C16	C8	111.9(2)	C19A	C16A	C8A	111.9(2)
C19	C16	C18	107.7(3)	C19A	C16A	C17A	108.6(3)
N5	C20	N4	175.5(3)	N5A	C20A	N4A	175.6(3)
C22	C21	Ni1	120.3(4)	C22A	C21A	Ni1A	119.5(3)
C22	C21	C26	117.9(4)	C22A	C21A	C26A	117.4(4)
C26	C21	Ni1	121.3(3)	C26A	C21A	Ni1A	122.8(3)
C23	C22	C21	122.8(5)	C23A	C22A	C21A	122.6(4)
C22	C23	C24	118.9(5)	C24A	C23A	C22A	119.6(5)
C25	C24	C23	120.1(4)	C25A	C24A	C23A	118.4(4)
C24	C25	C26	120.8(5)	C24A	C25A	C26A	122.6(5)
C21	C26	C25	119.5(4)	C21A	C26A	C25A	119.2(4)
C21	C26	C27	122.0(4)	C21A	C26A	C27A	121.7(4)
C25	C26	C27	118.4(5)	C25A	C26A	C27A	119.0(4)
C22B	C21B	Ni1	120.2(6)	C22C	C21C	Ni1A	115.6(6)
C22B	C21B	C26B	117.5(6)	C22C	C21C	C26C	117.7(7)
C26B	C21B	Ni1	121.8(6)	C26C	C21C	Ni1A	126.3(6)

Table 5 Bond Angles for weix10.

Atom	Atom	Atom	Angle/°	Atom	Atom	Atom	Angle/°
C23B	C22B	C21B	122.8(8)	C23C	C22C	C21C	122.7(8)
C22B	C23B	C24B	118.9(7)	C24C	C23C	C22C	119.0(7)
C25B	C24B	C23B	120.0(6)	C25C	C24C	C23C	120.1(7)
C24B	C25B	C26B	120.7(6)	C24C	C25C	C26C	120.6(7)
C21B	C26B	C25B	119.7(6)	C21C	C26C	C25C	119.8(6)
C21B	C26B	C27B	121.0(7)	C21C	C26C	C27C	121.0(7)
C25B	C26B	C27B	119.2(7)	C25C	C26C	C27C	119.2(7)

Table 6 Torsion Angles for weix10.

A	B	C	D	Angle/°	A	B	C	D	Angle/°
Ni1	N1	C1	C2	173.6(3)	Ni1A	N1A	C5A	C6A	6.8(3)
Ni1	N1	C5	C4	-172.7(2)	Ni1A	N2A	C6A	C5A	-2.7(3)
Ni1	N1	C5	C6	5.5(3)	Ni1A	N2A	C6A	C7A	177.4(2)
Ni1	N2	C6	C5	-4.0(3)	Ni1A	N2A	C10A	C9A	-177.4(2)
Ni1	N2	C6	C7	175.0(2)	Ni1A	N2A	C10A	C11A	0.7(3)
Ni1	N2	C10	C9	-175.4(2)	Ni1A	N3A	C11A	N4A	-177.2(3)
Ni1	N2	C10	C11	2.1(3)	Ni1A	N3A	C11A	C10A	2.8(4)
Ni1	N3	C11	N4	-178.6(3)	Ni1A	C21A	C22A	C23A	176.4(3)
Ni1	N3	C11	C10	2.0(4)	Ni1A	C21A	C26A	C25A	-177.3(4)
Ni1	C21	C22	C23	174.6(5)	Ni1A	C21A	C26A	C27A	1.9(6)
Ni1	C21	C26	C25	-173.5(4)	Ni1A	C21C	C22C	C23C	-173.1(16)
Ni1	C21	C26	C27	3.0(8)	Ni1A	C21C	C26C	C25C	174.7(18)
Ni1	C21B	C22B	C23B	-179(2)	Ni1A	C21C	C26C	C27C	-6(3)
Ni1	C21B	C26B	C25B	176.4(11)	N1A	Ni1A	N2A	C6A	5.2(2)
Ni1	C21B	C26B	C27B	-7(2)	N1A	Ni1A	N2A	C10A	-178.8(2)
N1	Ni1	N2	C6	5.7(2)	N1A	Ni1A	N3A	C11A	0.2(6)
N1	Ni1	N2	C10	179.3(3)	N1A	Ni1A	C21A	C22A	118.2(4)
N1	Ni1	N3	C11	0.4(6)	N1A	Ni1A	C21A	C26A	-67.4(4)
N1	C1	C2	C3	-2.0(6)	N1A	C1A	C2A	C3A	-1.1(5)
N1	C5	C6	N2	-1.2(4)	N1A	C5A	C6A	N2A	-2.8(4)
N1	C5	C6	C7	179.9(3)	N1A	C5A	C6A	C7A	177.0(3)
N2	Ni1	N3	C11	-0.7(3)	N2A	Ni1A	N3A	C11A	-2.0(2)
N2	C6	C7	C8	-0.6(5)	N2A	C6A	C7A	C8A	-0.4(5)
N2	C10	C11	N3	-2.5(4)	N2A	C10A	C11A	N3A	-2.2(4)
N2	C10	C11	N4	177.9(3)	N2A	C10A	C11A	N4A	177.9(3)
N3	Ni1	N2	C6	-174.6(3)	N3A	Ni1A	N2A	C6A	-175.4(3)
N3	Ni1	N2	C10	-1.0(2)	N3A	Ni1A	N2A	C10A	0.6(2)
C1	N1	C5	C4	3.9(5)	N3A	Ni1A	C21A	C22A	-61.4(4)
C1	N1	C5	C6	-177.9(3)	N3A	Ni1A	C21A	C26A	113.0(4)
C1	C2	C3	C4	4.8(5)	C1A	N1A	C5A	C4A	5.4(4)

Table 6 Torsion Angles for weix10.

A	B	C	D	Angle/°	A	B	C	D	Angle/°
C1	C2	C3	C12	-171.7(3)	C1A	N1A	C5A	C6A	-176.4(3)
C2	C3	C4	C5	-3.3(5)	C1A	C2A	C3A	C4A	4.4(5)
C2	C3	C12	C13	-12.9(5)	C1A	C2A	C3A	C12A	-171.8(3)
C2	C3	C12	C14	106.8(4)	C2A	C3A	C4A	C5A	-2.8(5)
C2	C3	C12	C15	-134.3(4)	C2A	C3A	C12A	C13A	-12.5(5)
C3	C4	C5	N1	-1.0(5)	C2A	C3A	C12A	C14A	107.5(4)
C3	C4	C5	C6	-179.0(3)	C2A	C3A	C12A	C15A	-134.0(3)
C4	C3	C12	C13	170.8(4)	C3A	C4A	C5A	N1A	-2.1(5)
C4	C3	C12	C14	-69.5(4)	C3A	C4A	C5A	C6A	179.9(3)
C4	C3	C12	C15	49.4(4)	C4A	C3A	C12A	C13A	171.5(3)
C4	C5	C6	N2	177.0(3)	C4A	C3A	C12A	C14A	-68.5(4)
C4	C5	C6	C7	-1.9(5)	C4A	C3A	C12A	C15A	50.0(4)
C5	N1	C1	C2	-2.4(5)	C4A	C5A	C6A	N2A	175.3(3)
C5	C6	C7	C8	178.1(3)	C4A	C5A	C6A	C7A	-4.9(5)
C6	N2	C10	C9	-1.8(5)	C5A	N1A	C1A	C2A	-3.8(5)
C6	N2	C10	C11	175.7(3)	C5A	C6A	C7A	C8A	179.8(3)
C6	C7	C8	C9	0.1(5)	C6A	N2A	C10A	C9A	-1.4(5)
C6	C7	C8	C16	179.2(3)	C6A	N2A	C10A	C11A	176.6(3)
C7	C8	C9	C10	-0.4(5)	C6A	C7A	C8A	C9A	-0.8(5)
C7	C8	C16	C17	56.9(4)	C6A	C7A	C8A	C16A	178.6(3)
C7	C8	C16	C18	-62.7(4)	C7A	C8A	C9A	C10A	0.9(5)
C7	C8	C16	C19	179.2(4)	C7A	C8A	C16A	C17A	61.6(4)
C8	C9	C10	N2	1.2(5)	C7A	C8A	C16A	C18A	-58.6(4)
C8	C9	C10	C11	-175.8(3)	C7A	C8A	C16A	C19A	-178.3(3)
C9	C8	C16	C17	-124.0(4)	C8A	C9A	C10A	N2A	0.2(5)
C9	C8	C16	C18	116.4(4)	C8A	C9A	C10A	C11A	-177.5(3)
C9	C8	C16	C19	-1.7(5)	C9A	C8A	C16A	C17A	-119.0(3)
C9	C10	C11	N3	174.8(3)	C9A	C8A	C16A	C18A	120.8(3)
C9	C10	C11	N4	-4.8(5)	C9A	C8A	C16A	C19A	1.1(5)
C10	N2	C6	C5	-177.5(3)	C9A	C10A	C11A	N3A	175.6(3)
C10	N2	C6	C7	1.5(5)	C9A	C10A	C11A	N4A	-4.3(5)
C12	C3	C4	C5	173.2(3)	C10A	N2A	C6A	C5A	-178.6(3)
C16	C8	C9	C10	-179.5(3)	C10A	N2A	C6A	C7A	1.6(5)
C20	N4	C11	N3	0.0(6)	C12A	C3A	C4A	C5A	173.5(3)
C20	N4	C11	C10	179.5(3)	C16A	C8A	C9A	C10A	-178.5(3)
C21	Ni1	N3	C11	178.8(3)	C20A	N4A	C11A	N3A	-0.2(5)
C21	C22	C23	C24	-2.5(10)	C20A	N4A	C11A	C10A	179.7(3)
C22	C21	C26	C25	-1.2(8)	C21A	Ni1A	N3A	C11A	178.7(3)
C22	C21	C26	C27	175.3(8)	C21A	C22A	C23A	C24A	0.6(7)
C22	C23	C24	C25	1.8(9)	C22A	C21A	C26A	C25A	-2.9(7)

Table 6 Torsion Angles for weix10.

A	B	C	D	Angle/°	A	B	C	D	Angle/°
C23	C24	C25	C26	-0.9(10)	C22A	C21A	C26A	C27A	176.3(5)
C24	C25	C26	C21	0.6(9)	C22A	C23A	C24A	C25A	-1.7(8)
C24	C25	C26	C27	-176.0(8)	C23A	C24A	C25A	C26A	0.5(8)
C26	C21	C22	C23	2.2(10)	C24A	C25A	C26A	C21A	1.8(8)
C21B	Ni1	N2	C6	-77.3(11)	C24A	C25A	C26A	C27A	-177.4(5)
C21B	Ni1	N2	C10	96.3(10)	C26A	C21A	C22A	C23A	1.7(7)
C21B	Ni1	N3	C11	-159.0(4)	C21C	Ni1A	N2A	C6A	-76(3)
C21B	C22B	C23B	C24B	7(4)	C21C	Ni1A	N2A	C10A	100(3)
C22B	C21B	C26B	C25B	4(3)	C21C	Ni1A	N3A	C11A	-168.5(5)
C22B	C21B	C26B	C27B	-179(3)	C21C	C22C	C23C	C24C	-2(3)
C22B	C23B	C24B	C25B	-4(3)	C22C	C21C	C26C	C25C	2(3)
C23B	C24B	C25B	C26B	2(2)	C22C	C21C	C26C	C27C	-179(2)
C24B	C25B	C26B	C21B	-2(2)	C22C	C23C	C24C	C25C	0(3)
C24B	C25B	C26B	C27B	-178(2)	C23C	C24C	C25C	C26C	2(3)
C26B	C21B	C22B	C23B	-7(4)	C24C	C25C	C26C	C21C	-3(3)
Ni1A	N1A	C1A	C2A	172.3(2)	C24C	C25C	C26C	C27C	177.5(19)
Ni1A	N1A	C5A	C4A	-171.3(2)	C26C	C21C	C22C	C23C	1(3)

Table 7 Hydrogen Atom Coordinates (Å×10^4) and Isotropic Displacement Parameters (Å^2×10^3) for weix10.

Atom	*x*	*y*	*z*	U(eq)
H3	10169.9	1851.82	3251.93	46
H1	11491.14	542.03	5729.58	51
H2	11693.85	364.47	6870.39	53
H4	10028.23	1544.21	6454.09	42
H7	9094.1	2083.59	5550.64	43
H9	8377.29	2706.77	3499.88	46
H13A	11598.82	-318.46	7881.21	107
H13B	11690.76	534.35	8491.06	107
H13C	11955.95	835.93	7947.32	107
H14A	11396.24	2675.1	7654.31	81
H14B	11168.51	2407.43	8234.54	81
H14C	10691.54	2698.33	7475.61	81
H15A	10145.49	952.15	7458.53	78
H15B	10626.03	561.29	8195.82	78
H15C	10464.34	-250.64	7551.19	78
H17A	8439.91	3458.24	5547.05	92
H17B	7797.92	4009.36	5095.52	92
H17C	8376.43	4367.86	4973.89	92
H18A	7730.87	1159.52	4462.66	80

Table 7 Hydrogen Atom Coordinates (Å×10[4]) and Isotropic Displacement Parameters (Å[2]×10[3]) for weix10.

**Atom**	***x***	***y***	***z***	**U(eq)**
H18B	7419.25	1958.02	4818.75	80
H18C	8092.62	1518.14	5244.8	80
H19A	7708.89	3858.36	3726.29	107
H19B	7192.7	3365.68	3922.38	107
H19C	7491.02	2582.94	3544.2	107
H22	10786.45	-112.75	3655.04	45
H23	11616.8	-459.33	3425.75	50
H24	12469.4	681.92	3924.06	53
H25	12503.82	2069.22	4693.12	53
H27A	11312.83	3212.29	4899.62	77
H27B	12029.44	3252.64	5162.3	77
H27C	11732.82	2479.9	5546.84	77
H22B	11420.79	3112.6	5126.98	75
H23B	12410.95	3410.27	5296.26	58
H24B	12811.07	2341.25	4674.76	55
H25B	12270.05	832.24	4024.07	41
H27D	11045.64	228.03	3300.64	72
H27E	10836.71	-112.15	3886.38	72
H27F	11454.02	-595.63	3903.94	72
H3A	4864.14	2810.19	-1797.89	44
H1A	3539.35	4454.85	-664.29	43
H2A	3318.59	4780.8	258.43	44
H4A	4991.23	3689.42	1500.81	42
H7A	5949.61	3197.83	1521.89	41
H9A	6675.79	2302.45	202.44	43
H13D	3380.91	5604.11	1280.12	81
H13E	3313.18	4912.54	1880.7	81
H13F	3043.95	4413.91	1122.01	81
H14D	3623.12	2644.18	1449.61	86
H14E	3835.12	3044.1	2224.14	86
H14F	4324.94	2679.01	1950.77	86
H15D	4855.73	4473.23	2339.18	77
H15E	4372.21	4898.22	2607.72	77
H15F	4514.82	5645.08	2075.81	77
H17D	6634.02	1810.39	2185.4	82
H17E	7317.35	1420.56	2411.23	82
H17F	6793.04	883.74	1748.11	82
H18D	7268.59	4183.16	1697.25	85
H18E	7612.15	3462.74	2377.71	85

Table 7 Hydrogen Atom Coordinates (Å×10^4) and Isotropic Displacement Parameters (Å^2×10^3) for weix10.

Atom	*x*	*y*	*z*	U(eq)
H18F	6923.89	3817.48	2153.35	85
H19D	7401.89	1415.96	1116.54	89
H19E	7895.7	2059.51	1758.66	89
H19F	7555.4	2706.51	1048.93	89
H22A	4205.26	4775.46	-2089.32	46
H23A	3360.89	4979.79	-3117.04	56
H24A	2491.03	3926.11	-3321.39	64
H25A	2507.76	2634.66	-2509.81	61
H27G	3615.42	2246.96	-849.63	82
H27H	3485.92	1307.18	-1425.32	82
H27I	2936.73	1945.04	-1358.17	82
H22C	3546.28	2156.06	-1046.96	60
H23C	2597.74	1720.52	-1874.03	63
H24C	2232.73	2659.22	-2920.6	61
H25C	2822.2	4002.75	-3130.02	59
H27J	4306.44	4832.05	-1972.22	73
H27K	3713.73	5357.38	-2560.79	73
H27L	4058.47	4343.28	-2732.29	73

Table 8 Atomic Occupancy for weix10.

Atom	*Occupancy*	Atom	*Occupancy*	Atom	*Occupancy*
C21	0.747(6)	C22	0.747(6)	H22	0.747(6)
C23	0.747(6)	H23	0.747(6)	C24	0.747(6)
H24	0.747(6)	C25	0.747(6)	H25	0.747(6)
C26	0.747(6)	C27	0.747(6)	H27A	0.747(6)
H27B	0.747(6)	H27C	0.747(6)	C21B	0.253(6)
C22B	0.253(6)	H22B	0.253(6)	C23B	0.253(6)
H23B	0.253(6)	C24B	0.253(6)	H24B	0.253(6)
C25B	0.253(6)	H25B	0.253(6)	C26B	0.253(6)
C27B	0.253(6)	H27D	0.253(6)	H27E	0.253(6)
H27F	0.253(6)	C21A	0.766(6)	C22A	0.766(6)
H22A	0.766(6)	C23A	0.766(6)	H23A	0.766(6)
C24A	0.766(6)	H24A	0.766(6)	C25A	0.766(6)
H25A	0.766(6)	C26A	0.766(6)	C27A	0.766(6)
H27G	0.766(6)	H27H	0.766(6)	H27I	0.766(6)
C21C	0.234(6)	C22C	0.234(6)	H22C	0.234(6)
C23C	0.234(6)	H23C	0.234(6)	C24C	0.234(6)
H24C	0.234(6)	C25C	0.234(6)	H25C	0.234(6)
C26C	0.234(6)	C27C	0.234(6)	H27J	0.234(6)

Table 8 Atomic Occupancy for weix10.

Atom	*Occupancy*	Atom	*Occupancy*	Atom	*Occupancy*
H27K	0.234(6)	H27L	0.234(6)		

Table 9 Solvent masks information for weix10.

Number	X	Y	Z	Volume	Electron count	Content
1	0.000	0.000	0.000	257.1	53.3	?
2	0.000	0.500	0.500	257.1	53.3	?
3	0.500	0.000	0.000	293.9	71.6	?
4	0.500	0.500	0.500	293.9	71.6	?

5. References

(1) He, Z.-T.; Hartwig, J. F. Palladium-Catalyzed α-Arylation for the Addition of Small Rings to Aromatic Compounds. *Nat. Commun.* **2019**, *10* (1), 4083. https://doi.org/10.1038/s41467-019-12090-z.

(2) Sim, B. A.; Griller, D.; Wayner, D. D. M. Reduction Potentials for Substituted Benzyl Radicals: PKa Values for the Corresponding Toluenes. *J. Am. Chem. Soc.* **1989**, *111* (2), 754–755. https://doi.org/10.1021/ja00184a066.

(3) Barzanò, G.; Mao, R.; Garreau, M.; Waser, J.; Hu, X. Tandem Photoredox and Copper-Catalyzed Decarboxylative C(Sp3)–N Coupling of Anilines and Imines Using an Organic Photocatalyst. *Org Lett* **2020**, 5.

(4) Mao, R.; Balon, J.; Hu, X. Decarboxylative C(Sp3)−O Cross-Coupling. *Angew. Chem. Int. Ed.* **2018**, *57* (41), 13624–13628. https://doi.org/10.1002/anie.201808024.

(5) Wang, J.; Cary, B. P.; Beyer, P. D.; Gellman, S. H.; Weix, D. J. Ketones from Nickel-Catalyzed Decarboxylative, Non-Symmetric Cross-Electrophile Coupling of Carboxylic Acid Esters. *Angew. Chem. Int. Ed.* **2019**, *58* (35), 12081–12085. https://doi.org/10.1002/anie.201906000.

(6) Gao, L.; Wang, G.; Cao, J.; Chen, H.; Gu, Y.; Liu, X.; Cheng, X.; Ma, J.; Li, S. Lewis Acid-Catalyzed Selective Reductive Decarboxylative Pyridylation of *N* - Hydroxyphthalimide Esters: Synthesis of Congested Pyridine-Substituted Quaternary Carbons. *ACS Catal.* **2019**, *9* (11), 10142–10151. https://doi.org/10.1021/acscatal.9b03798.

(7) Qin, T.; Malins, L. R.; Edwards, J. T.; Merchant, R. R.; Novak, A. J. E.; Zhong, J. Z.; Mills, R. B.; Yan, M.; Yuan, C.; Eastgate, M. D.; Baran, P. S. Nickel-Catalyzed Barton Decarboxylation and Giese Reactions: A Practical Take on Classic Transforms. *Angew. Chem. Int. Ed.* **2017**, *56* (1), 260–265. https://doi.org/10.1002/anie.201609662.

(8) Polites, V. C.; Badir, S. O.; Keess, S.; Jolit, A.; Molander, G. A. Nickel-Catalyzed Decarboxylative Cross-Coupling of Bicyclo[1.1.1]Pentyl Radicals Enabled by Electron Donor–Acceptor Complex Photoactivation. *Org. Lett.* **2021**, *23* (12), 4828–4833. https://doi.org/10.1021/acs.orglett.1c01558.

(9) Ni, S.; Padial, N. M.; Kingston, C.; Vantourout, J. C.; Schmitt, D. C.; Edwards, J. T.; Kruszyk, M. M.; Merchant, R. R.; Mykhailiuk, P. K.; Sanchez, B. B.; Yang, S.; Perry, M. A.; Gallego, G. M.; Mousseau, J. J.; Collins, M. R.; Cherney, R. J.; Lebed, P. S.; Chen, J. S.; Qin, T.; Baran, P. S. A Radical Approach to Anionic Chemistry: Synthesis of Ketones,

Alcohols, and Amines. *J. Am. Chem. Soc.* **2019**, *141* (16), 6726–6739. https://doi.org/10.1021/jacs.9b02238.
(10) Toriyama, F.; Cornella, J.; Wimmer, L.; Chen, T.-G.; Dixon, D. D.; Creech, G.; Baran, P. S. Redox-Active Esters in Fe-Catalyzed C–C Coupling. *J. Am. Chem. Soc.* **2016**, *138* (35), 11132–11135. https://doi.org/10.1021/jacs.6b07172.
(11) Fawcett, A.; Pradeilles, J.; Wang, Y.; Mutsuga, T.; Myers, E. L.; Aggarwal, V. K. Photoinduced Decarboxylative Borylation of Carboxylic Acids. *Science* **2017**, *357* (6348), 283–286. https://doi.org/10.1126/science.aan3679.
(12) Watanabe, E.; Chen, Y.; May, O.; Ley, S. V. A Practical Method for Continuous Production of Sp3-Rich Compounds from (Hetero)Aryl Halides and Redox-Active Esters. *Chem. – Eur. J.* **2020**, *26* (1), 186–191. https://doi.org/10.1002/chem.201905048.
(13) M. Maiwald, M.; T. Wagner, A.; Kratsch, J.; Skerencak-Frech, A.; Trumm, M.; Geist, A.; W. Roesky, P.; J. Panak, P. 4,4′-Di- Tert -Butyl-6-(1 H -Tetrazol-5-Yl)-2,2′-Bipyridine: Modification of a Highly Selective N-Donor Ligand for the Separation of Trivalent Actinides from Lanthanides. *Dalton Trans.* **2017**, *46* (30), 9981–9994. https://doi.org/10.1039/C7DT01864A.
(14) Chi, B. K.; Widness, J. K.; Gilbert, M. M.; Salgueiro, D. C.; Garcia, K. J.; Weix, D. J. In-Situ Bromination Enables Formal Cross-Electrophile Coupling of Alcohols with Aryl and Alkenyl Halides. *ACS Catal.* **2022**, *12* (1), 580–586. https://doi.org/10.1021/acscatal.1c05208.
(15) Huffman, K. R.; Schaefer, F. C. Preparation and Reactions of N-Cyanoamidines. *J. Org. Chem.* **1963**, *28* (7), 1812–1816. https://doi.org/10.1021/jo01042a017.
(16) Huang, A.; Moretto, A.; Janz, K.; Lowe, M.; Bedard, P. W.; Tam, S.; Di, L.; Clerin, V.; Sushkova, N.; Tchernychev, B.; Tsao, D. H. H.; Keith, J. C.; Shaw, G. D.; Schaub, R. G.; Wang, Q.; Kaila, N. Discovery of 2-[1-(4-Chlorophenyl)Cyclopropyl]-3-Hydroxy-8-(Trifluoromethyl)Quinoline-4-Carboxylic Acid (PSI-421), a P-Selectin Inhibitor with Improved Pharmacokinetic Properties and Oral Efficacy in Models of Vascular Injury. *J. Med. Chem.* **2010**, *53* (16), 6003–6017. https://doi.org/10.1021/jm9013696.
(17) Brandstätter, M.; Huwyler, N.; Carreira, E. M. Gold(I)-Catalyzed Stereoselective Cyclization of 1,3-Enyne Aldehydes by a 1,3-Acyloxy Migration/Nazarov Cyclization/Aldol Addition Cascade. *Chem. Sci.* **2019**, *10* (35), 8219–8223. https://doi.org/10.1039/C9SC02828E.
(18) Chen, T.-G.; Zhang, H.; Mykhailiuk, P. K.; Merchant, R. R.; Smith, C. A.; Qin, T.; Baran, P. S. Quaternary Centers by Nickel-Catalyzed Cross-Coupling of Tertiary Carboxylic Acids and (Hetero)Aryl Zinc Reagents. *Angew. Chem. Int. Ed.* **2019**, *58* (8), 2454–2458. https://doi.org/10.1002/anie.201814524.
(19) Wrackmeyer, B. Carbon-13 NMR Spectroscopy of Boron Compounds. *Prog. Nucl. Magn. Reson. Spectrosc.* **1979**, *12* (4), 227–259. https://doi.org/10.1016/0079-6565(79)80003-5.
(20) Lévesque, É.; Goudreau, S. R.; Charette, A. B. Improved Zinc-Catalyzed Simmons–Smith Reaction: Access to Various 1,2,3-Trisubstituted Cyclopropanes. *Org. Lett.* **2014**, *16* (5), 1490–1493. https://doi.org/10.1021/ol500267w.
(21) Wiberg, K. B.; McMurdie, N. Formation and Reactions of Bicyclo[1.1.1]Pentyl-1 Cations. *J. Am. Chem. Soc.* **1994**, *116* (26), 11990–11998. https://doi.org/10.1021/ja00105a046.
(22) Bruker-AXS (2019). *APEX3.* Version 2019.11-0. Madison, Wisconsin, USA.

(23) Krause, L.; Herbst-Irmer, R.; Sheldrick, G. M.; Stalke, D. Comparison of Silver and Molybdenum Microfocus X-Ray Sources for Single-Crystal Structure Determination. *J Appl Crystallogr* **2015**, *48*, 3–10.

(24) Sheldrick, G. M. (2013b). *XPREP*. Version 2013/1. Georg-August-Universität Göttingen, Göttingen, Germany.

(25) Sheldrick, G. M. (2013a). The *SHELX* homepage, http://shelx.uni-ac.gwdg.de/SHELX/.

(26) Sheldrick, G. M. SHELXT – Integrated Space-Group and Crystal-Structure Determination. *Acta Cryst A* **2015**, *71*, 3–8.

(27) Sheldrick, G. M. Crystal Structure Refinement with SHELXL. *Acta Cryst C* **2015**, *71*, 3–8.

(28) Dolomanov, O. V.; Bourhis, L. J.; Gildea, R. J.; Howard, J. a. K.; Puschmann, H. OLEX2: A Complete Structure Solution, Refinement and Analysis Program. *J. Appl. Crystallogr.* **2009**, *42*, 339–341

(29) Guzei, I. A. (2007-2013). Programs *Gn*. University of Wisconsin-Madison, Madison, Wisconsin, USA.

6. NMR Spectra

^{1}H NMR spectrum of $^{t\text{-Bu}}$BpyCam^{CN} (500 MHz, $CDCl_3$)

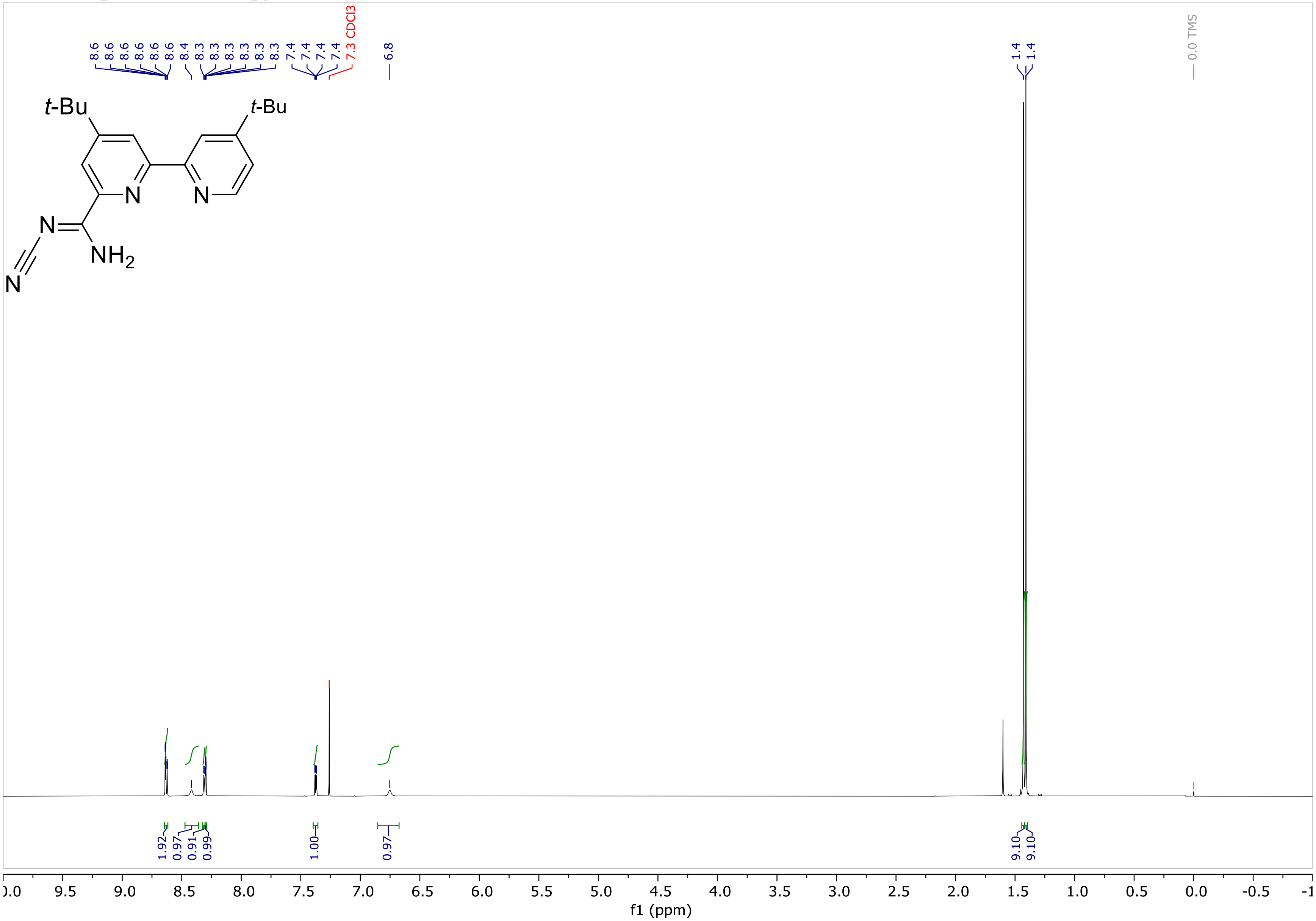

$^{13}C\{^1H\}$ NMR spectrum of $^{t\text{-Bu}}BpyCam^{CN}$ (126 MHz, $CDCl_3$)

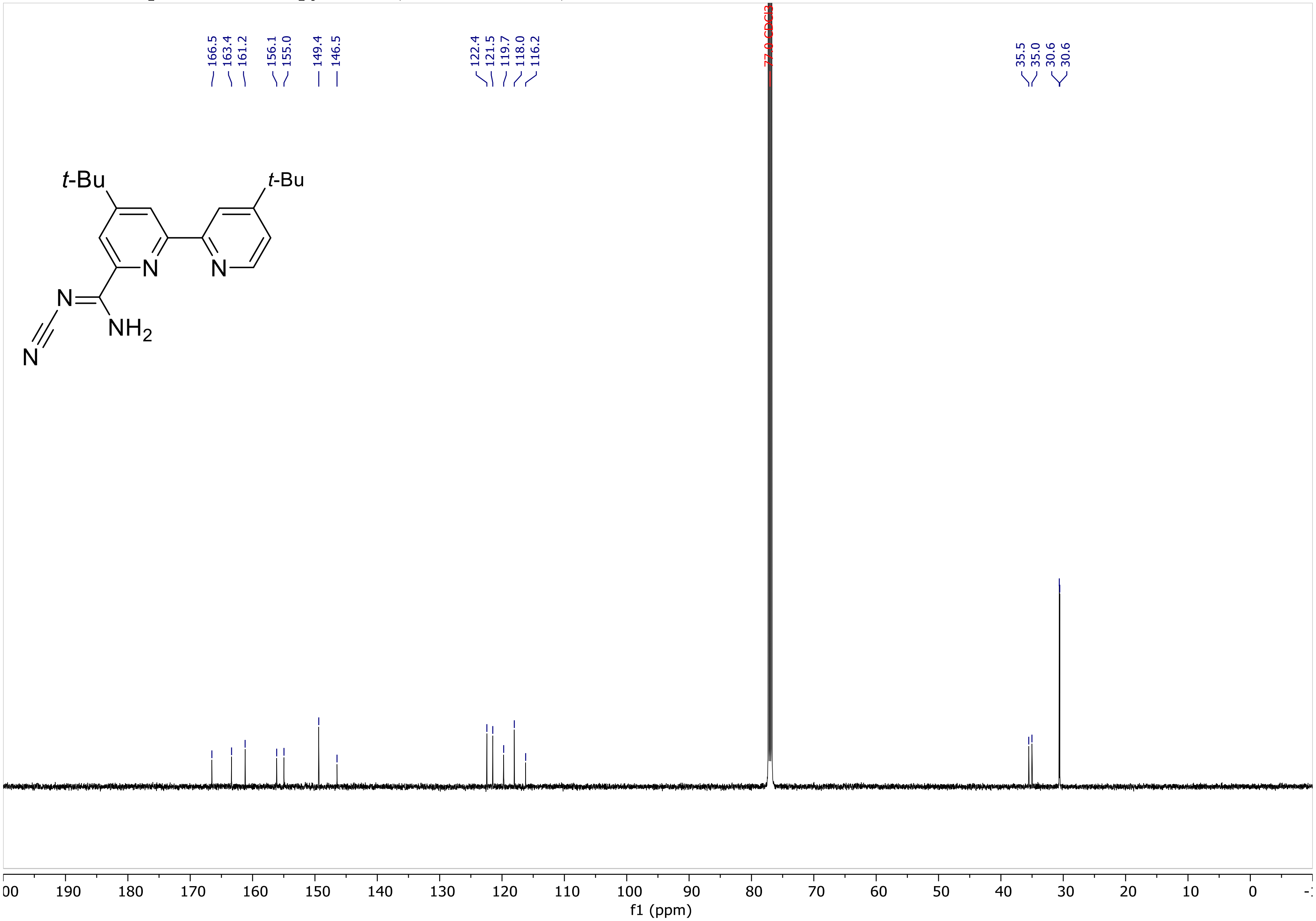

^{1}H NMR spectrum of compound SI-4 (500 MHz, $CDCl_3$)

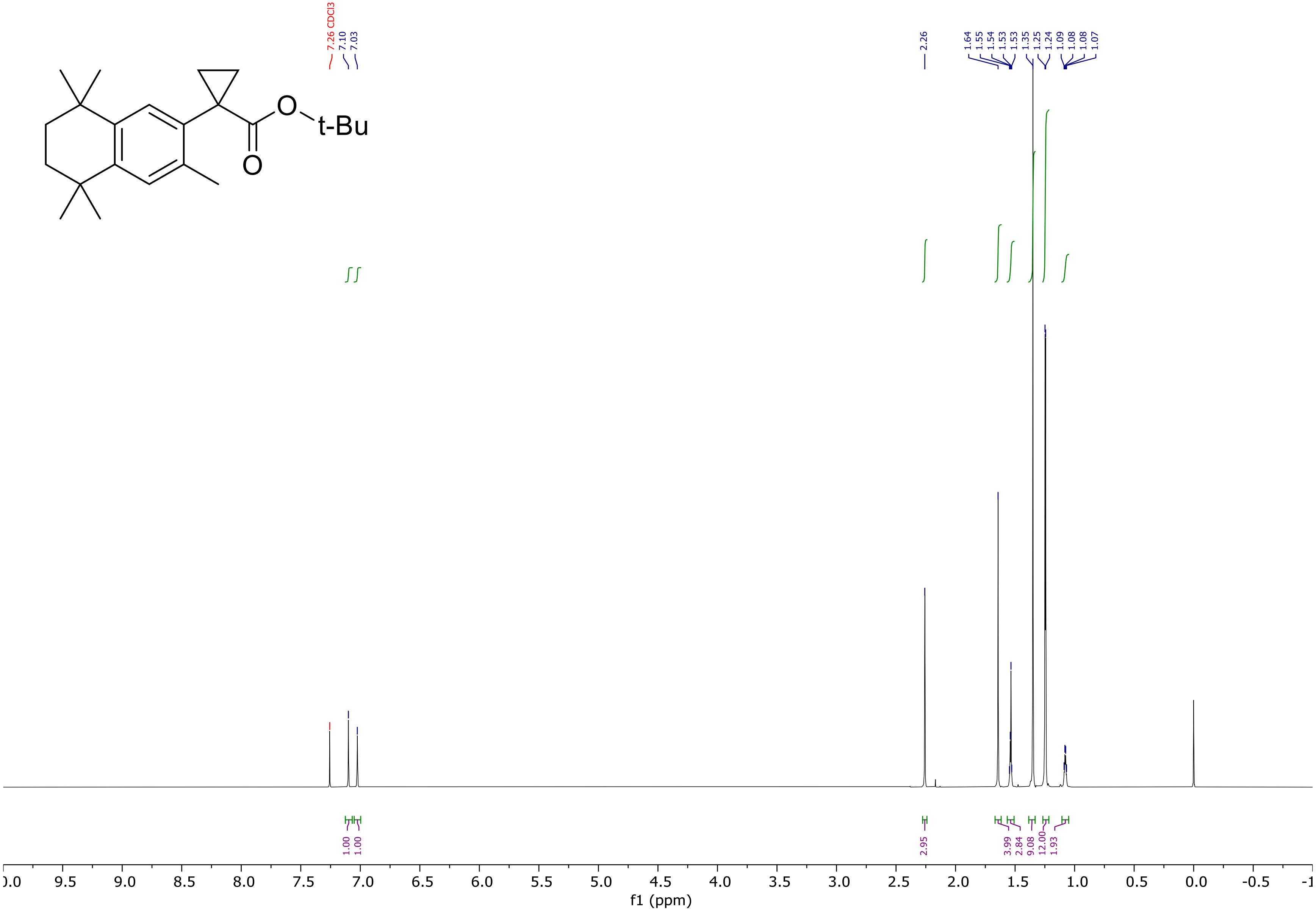

$^{13}C\{^{1}H\}$ NMR spectrum of compound SI-4 (126 MHz, $CDCl_3$)

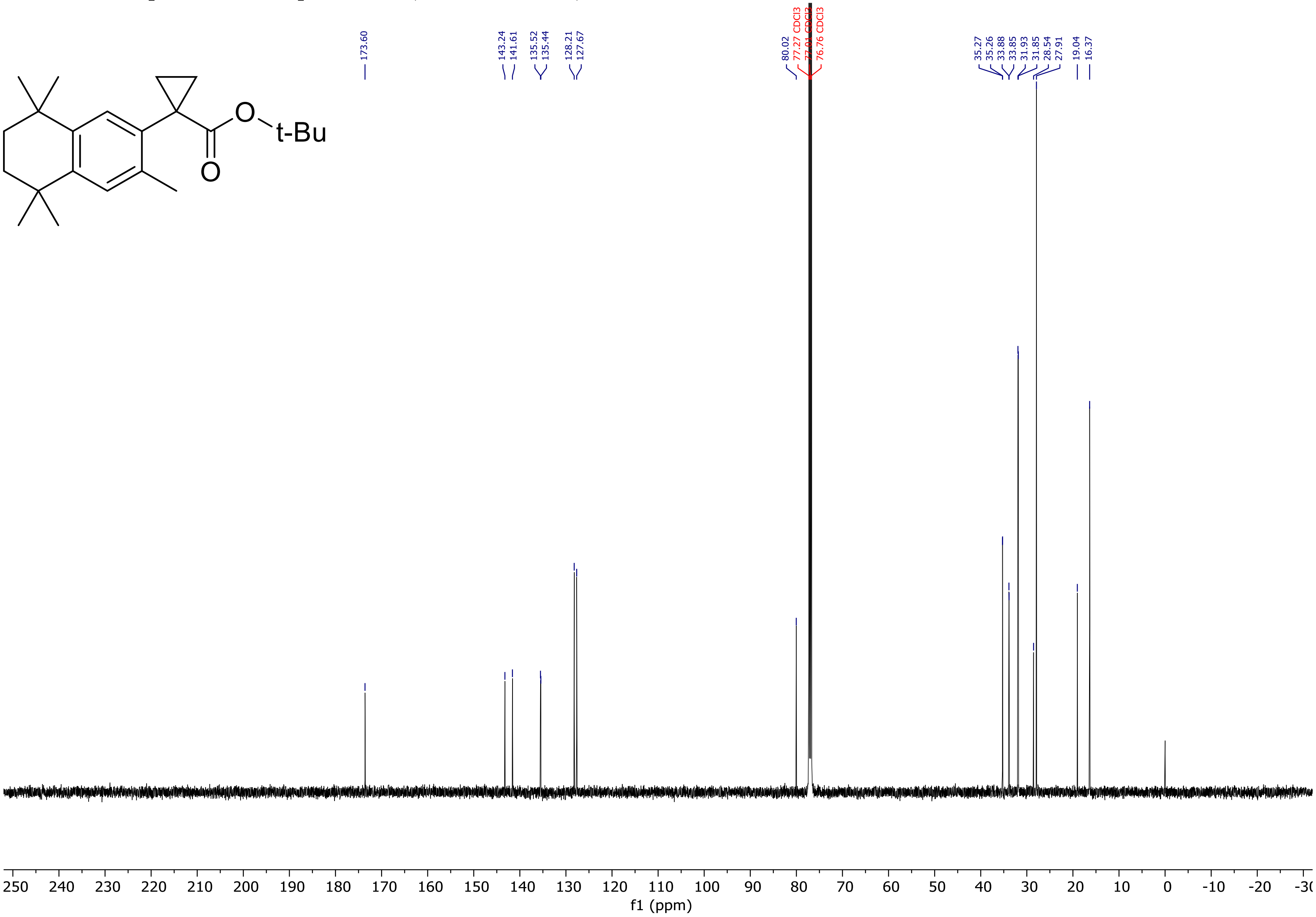

^{1}H NMR spectrum of compound SI-5 (500 MHz, $CDCl_3$)

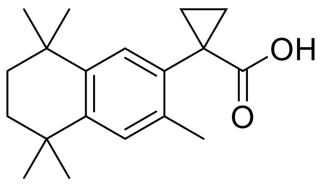

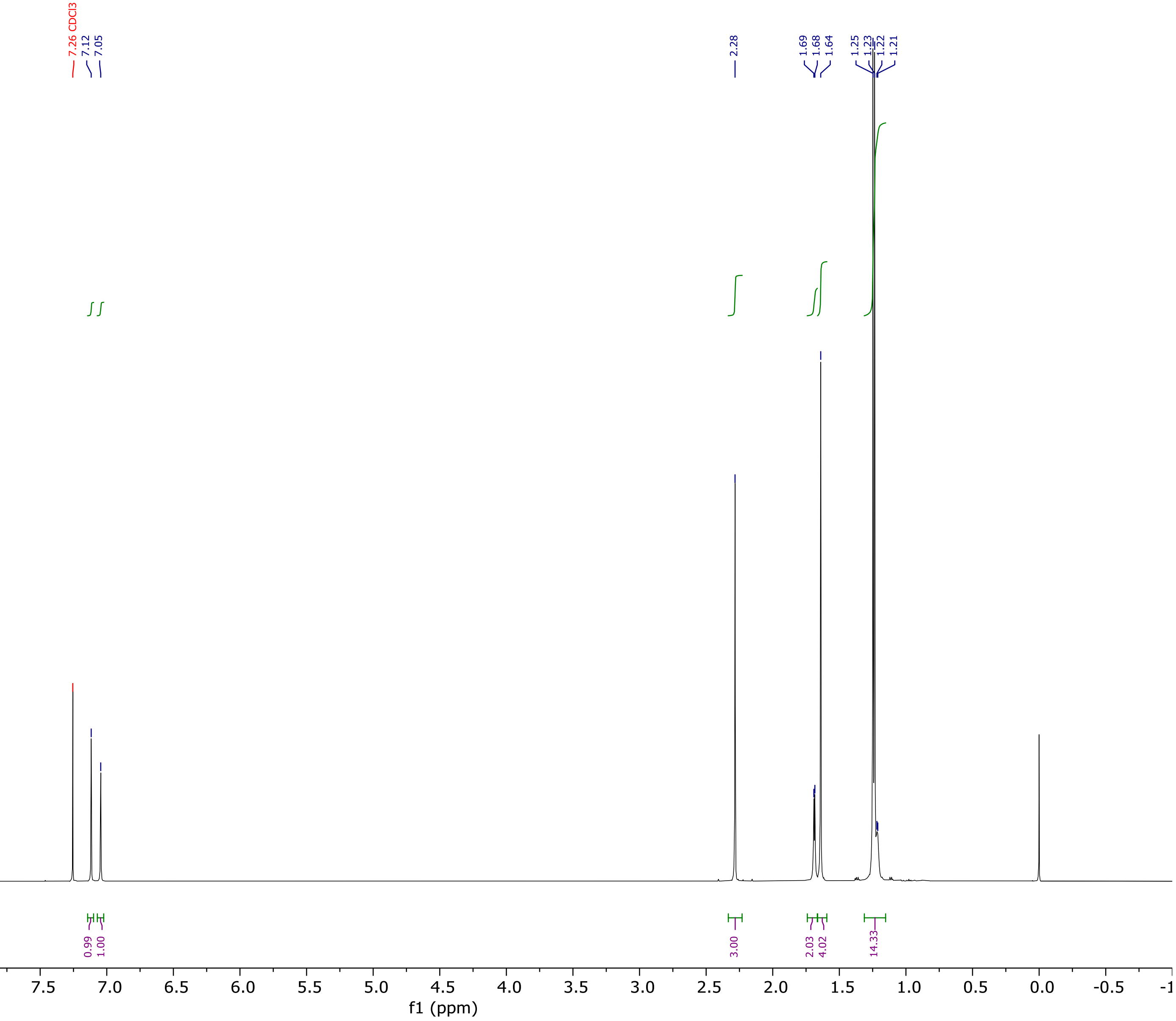

$^{13}C\{^1H\}$ **NMR spectrum of compound SI-5 (126 MHz, $CDCl_3$)**

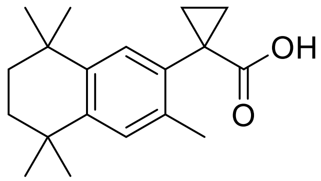

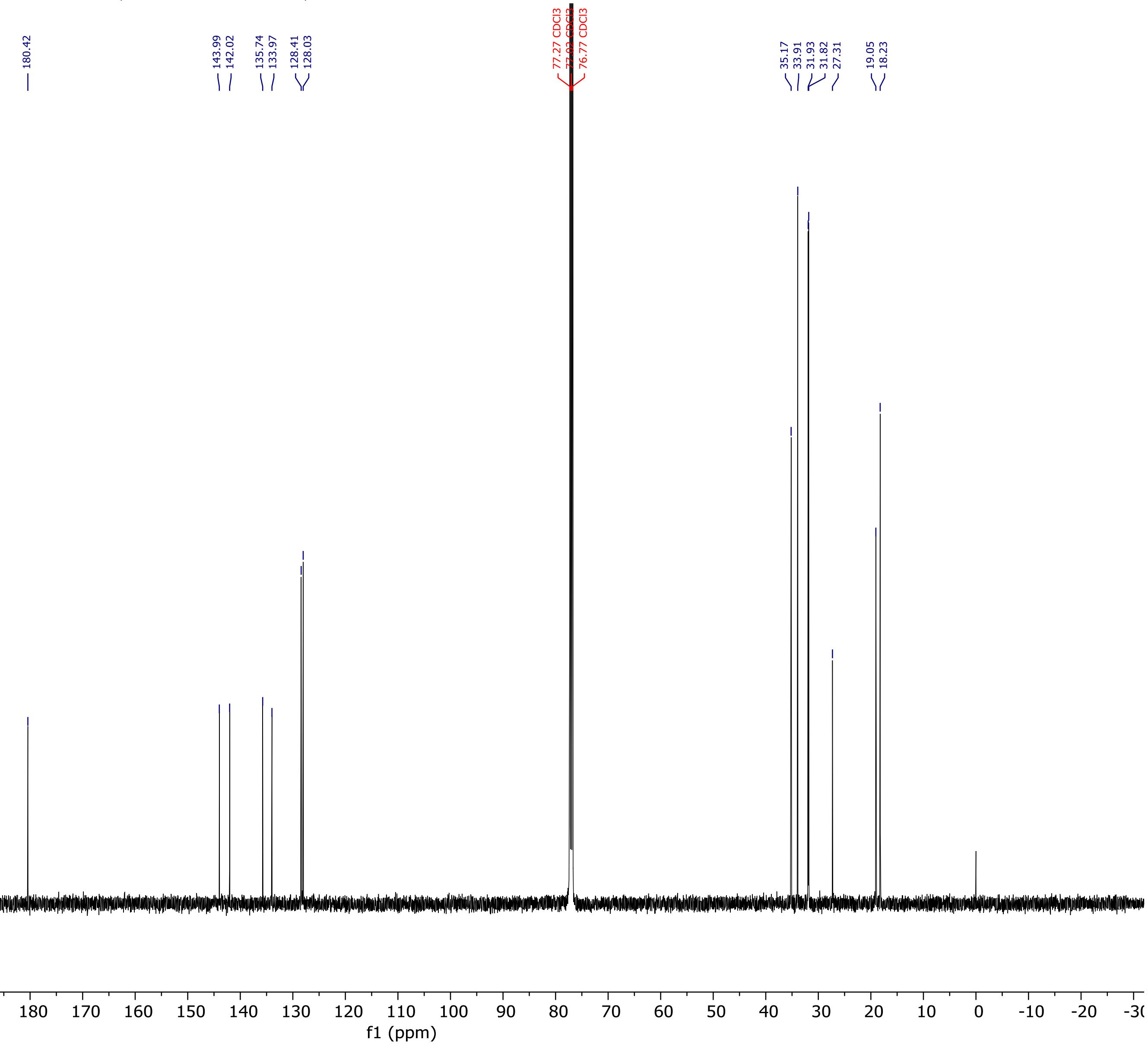

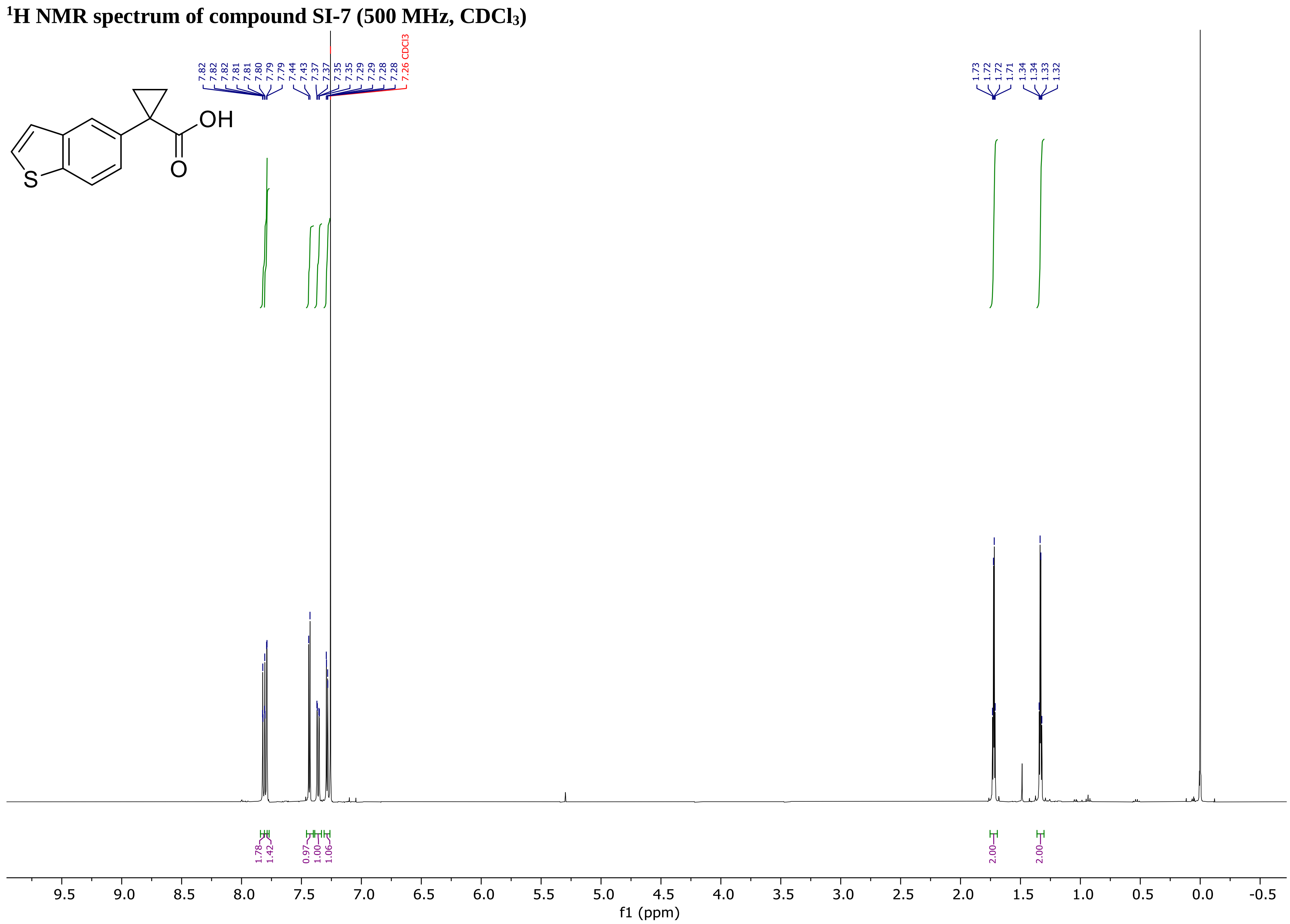

1H NMR spectrum of compound SI-7 (500 MHz, CDCl3)
S
OH
O
7.82
7.82
7.82
7.81
7.81
7.80
7.79
7.79
7.44
7.43
7.37
7.37
7.35
7.35
7.29
7.29
7.28
7.28
7.26 CDCl3
1.73
1.72
1.72
1.71
1.34
1.34
1.33
1.32
1.78
1.42
0.97
1.00
1.06
2.00
2.00
9.5
9.0
8.5
8.0
7.5
7.0
6.5
6.0
5.5
5.0
4.5
4.0
3.5
3.0
2.5
2.0
1.5
1.0
0.5
0.0
-0.5
f1 (ppm)

$^{13}C\{^1H\}$ **NMR spectrum of compound SI-7 (126 MHz, $CDCl_3$)**

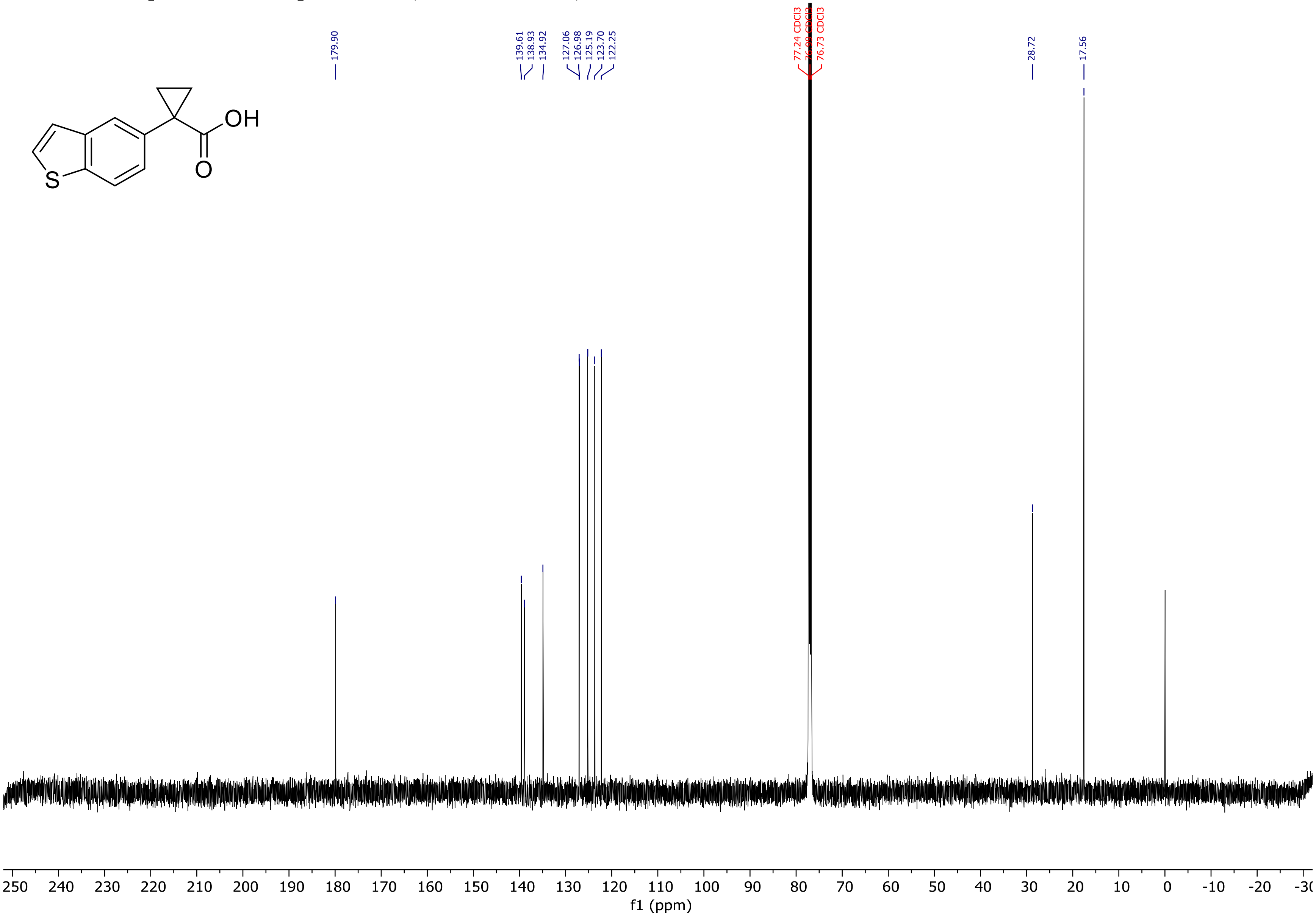

^{1}H NMR spectrum of compound SI-8 (500 MHz, CDCl$_3$)

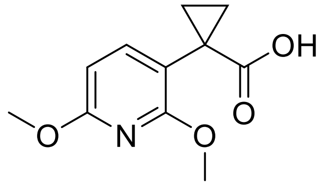

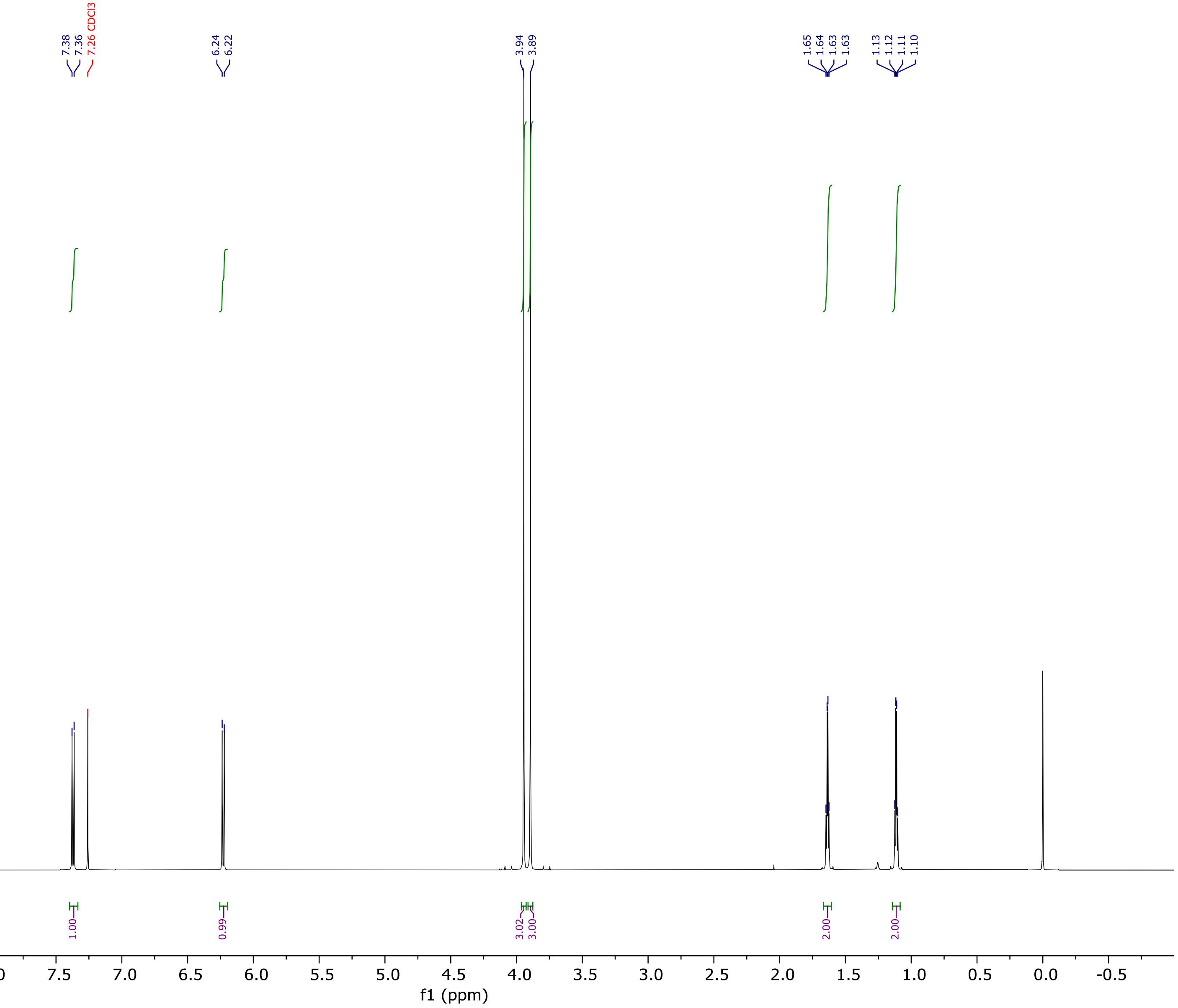

$^{13}C\{^1H\}$ NMR spectrum of compound SI-8 (126 MHz, $CDCl_3$)

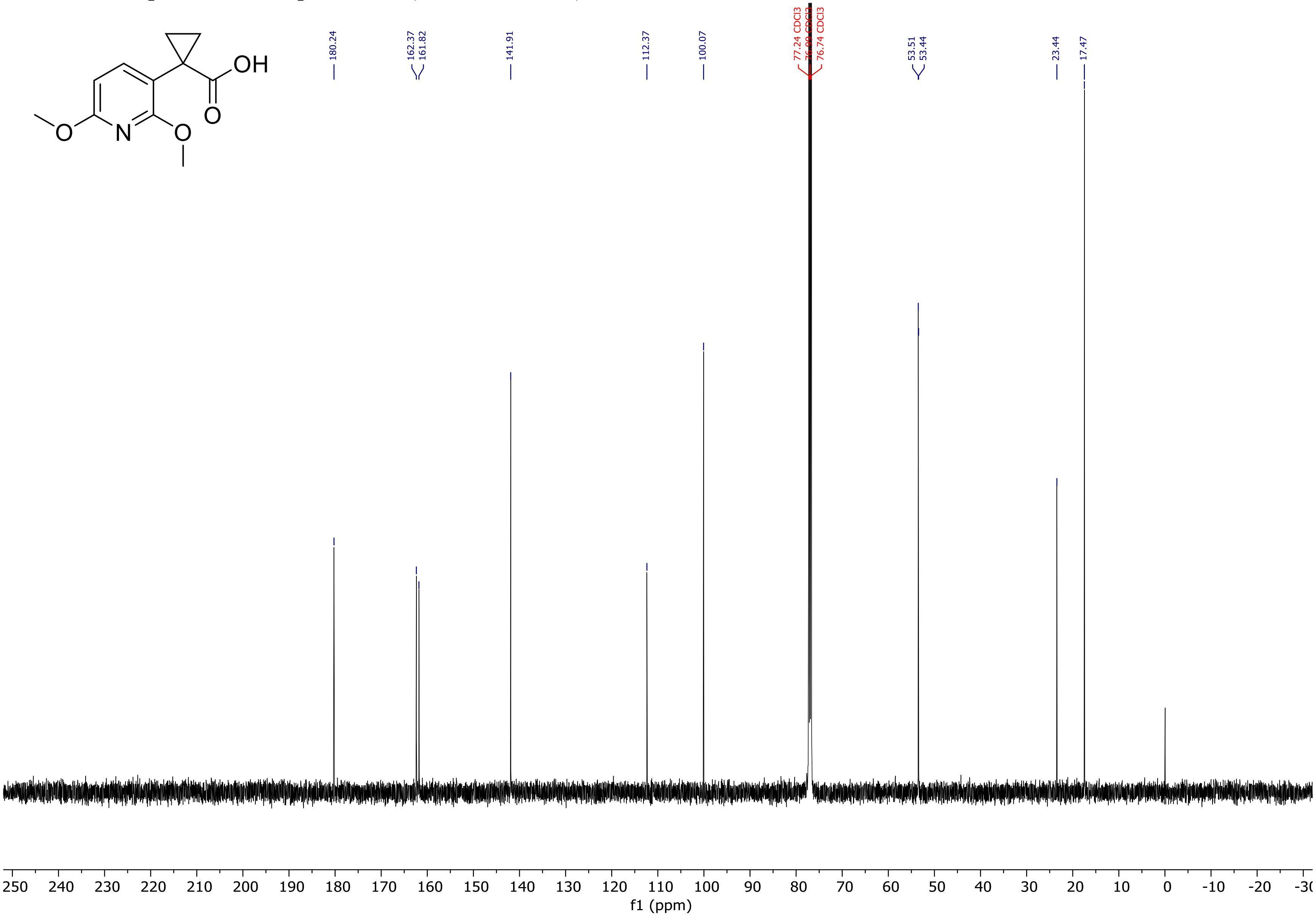

^{1}H NMR spectrum of compound 1a (500 MHz, $CDCl_3$)

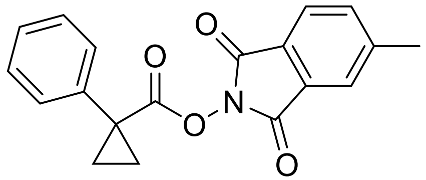

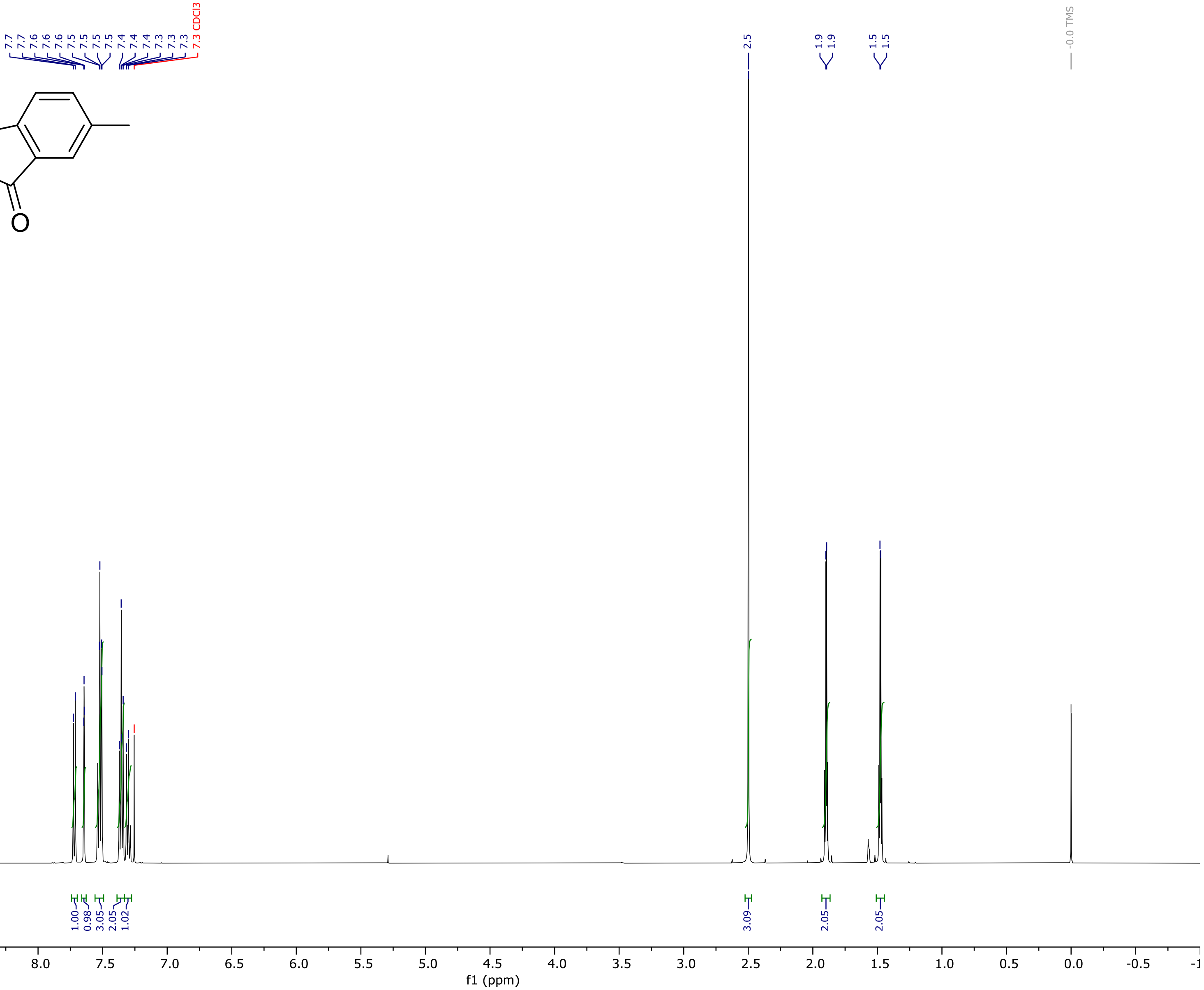

$^{13}C\{^{1}H\}$ NMR spectrum of compound 1a (126 MHz, CDCl$_3$)

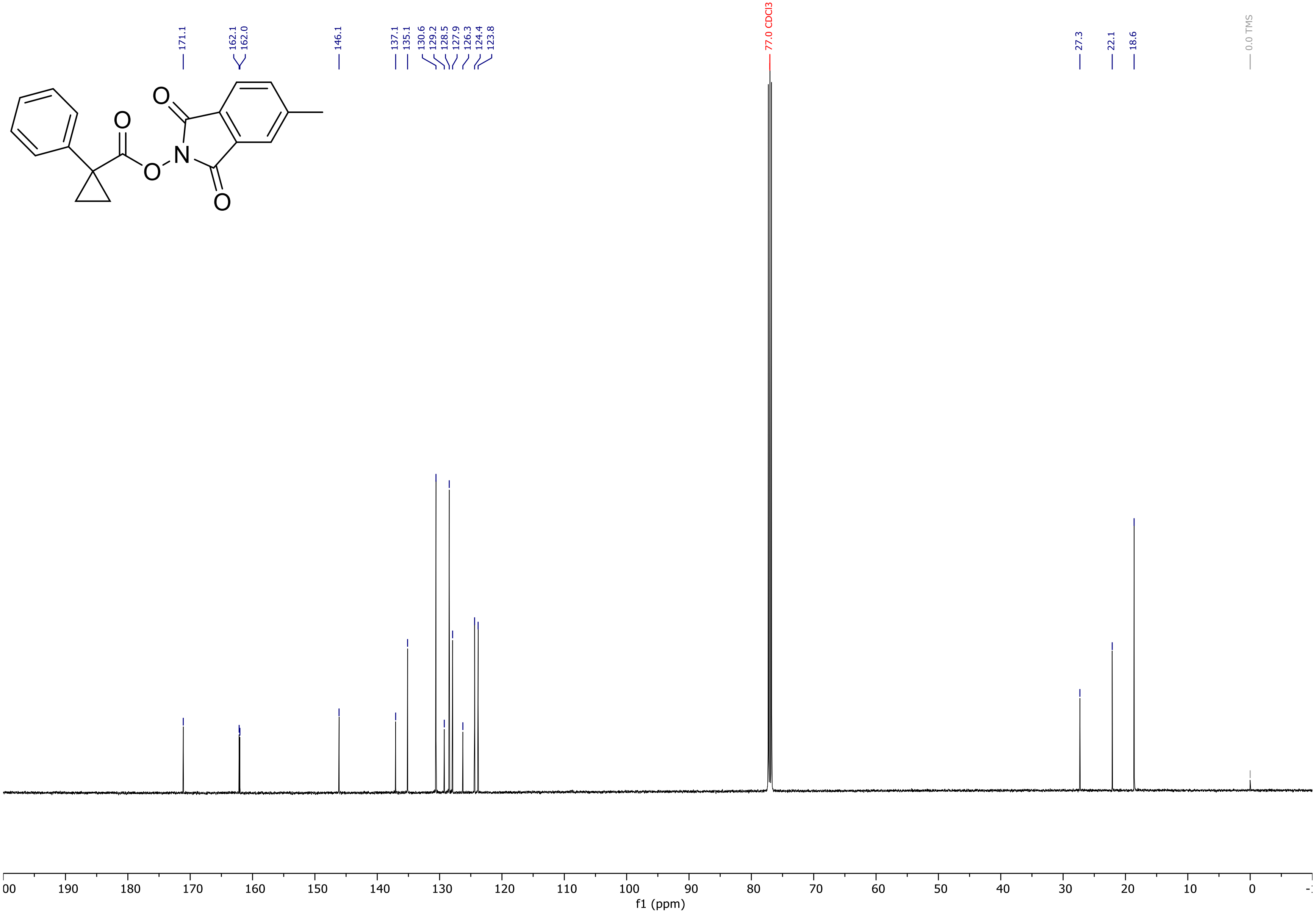

^{1}H NMR spectrum of compound 1b (500 MHz, $CDCl_3$)

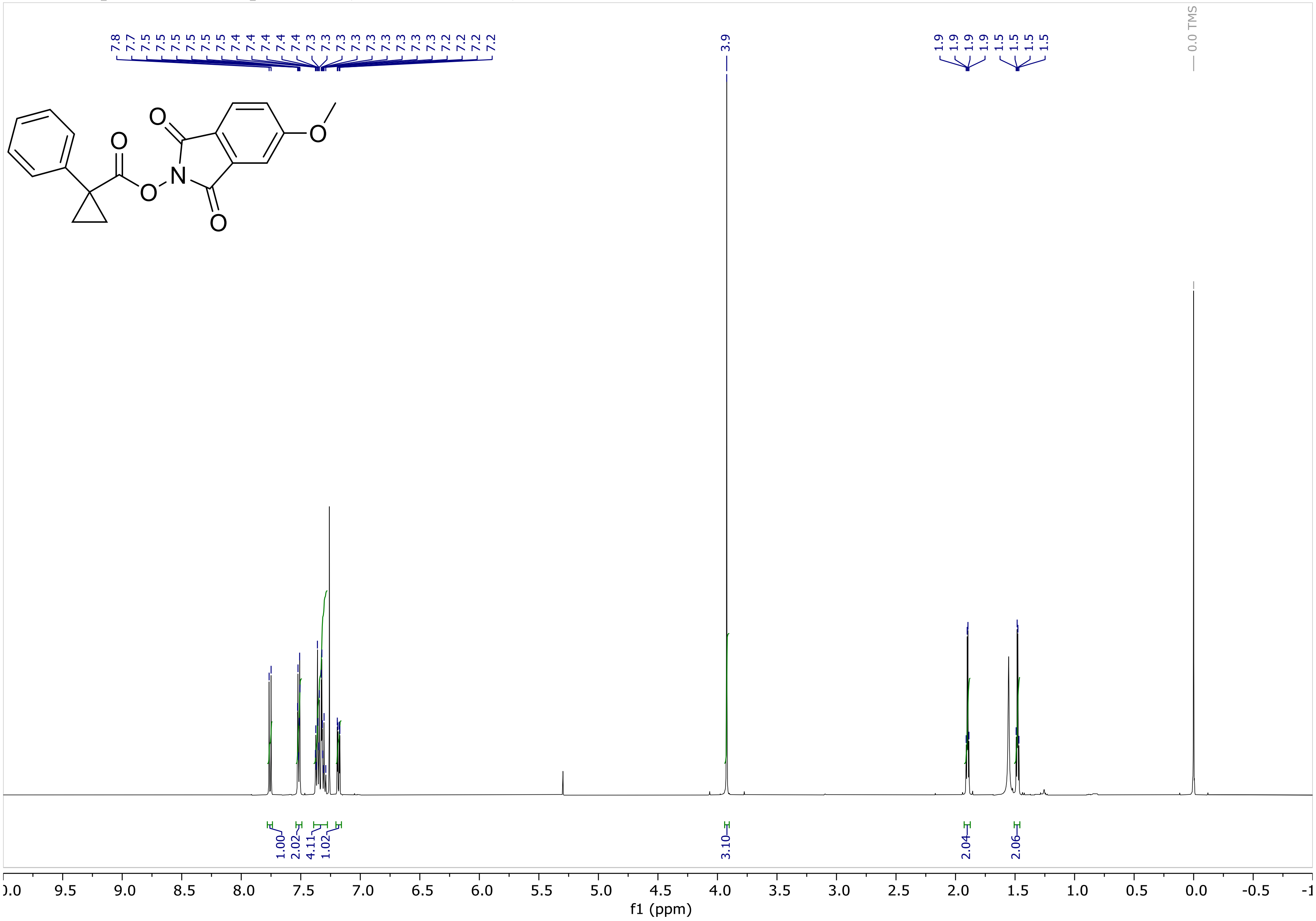

$^{13}C\{^1H\}$ NMR spectrum of compound 1b (126 MHz, $CDCl_3$)

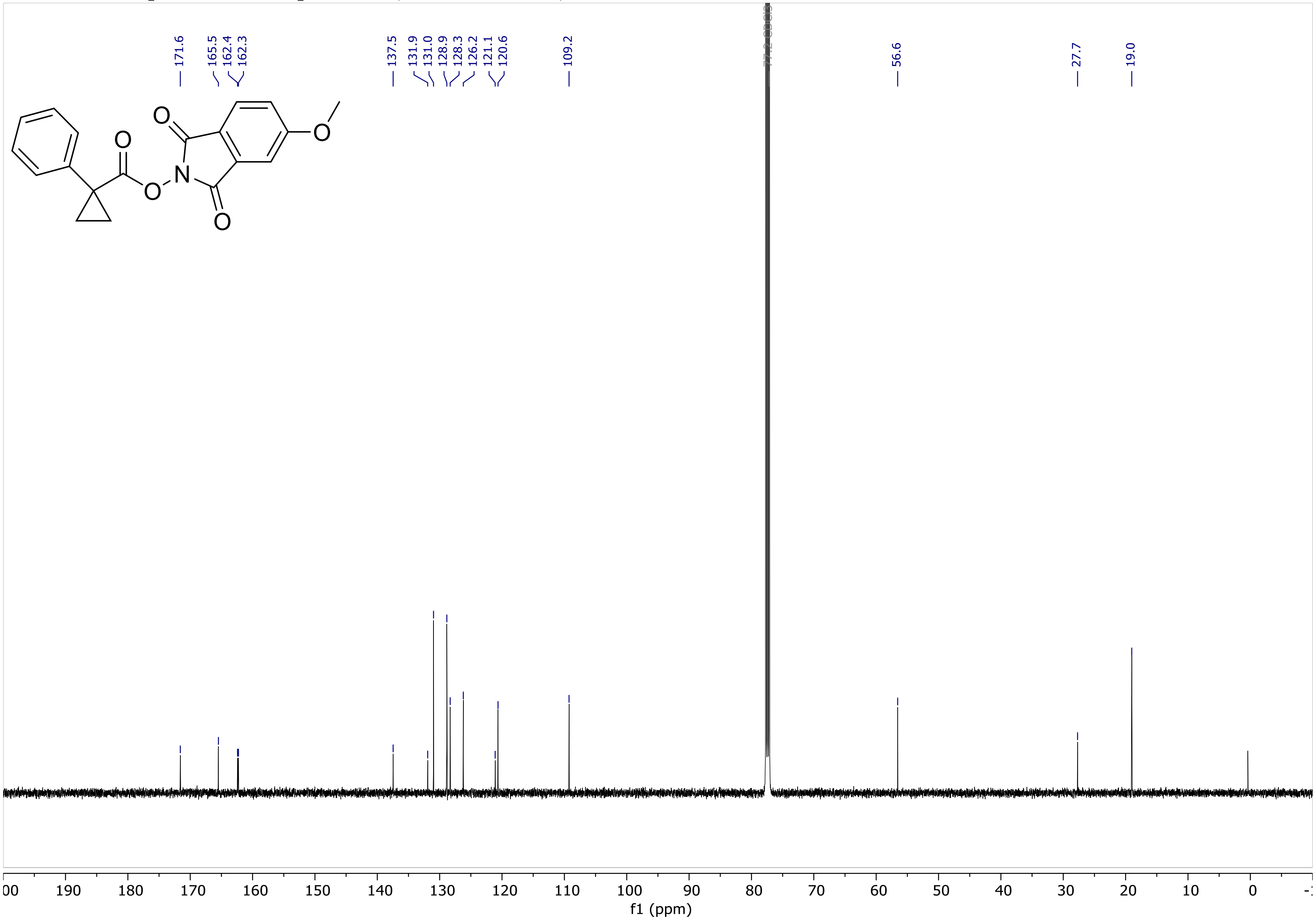

^{1}H NMR spectrum of compound 1c (500 MHz, CDCl$_3$)

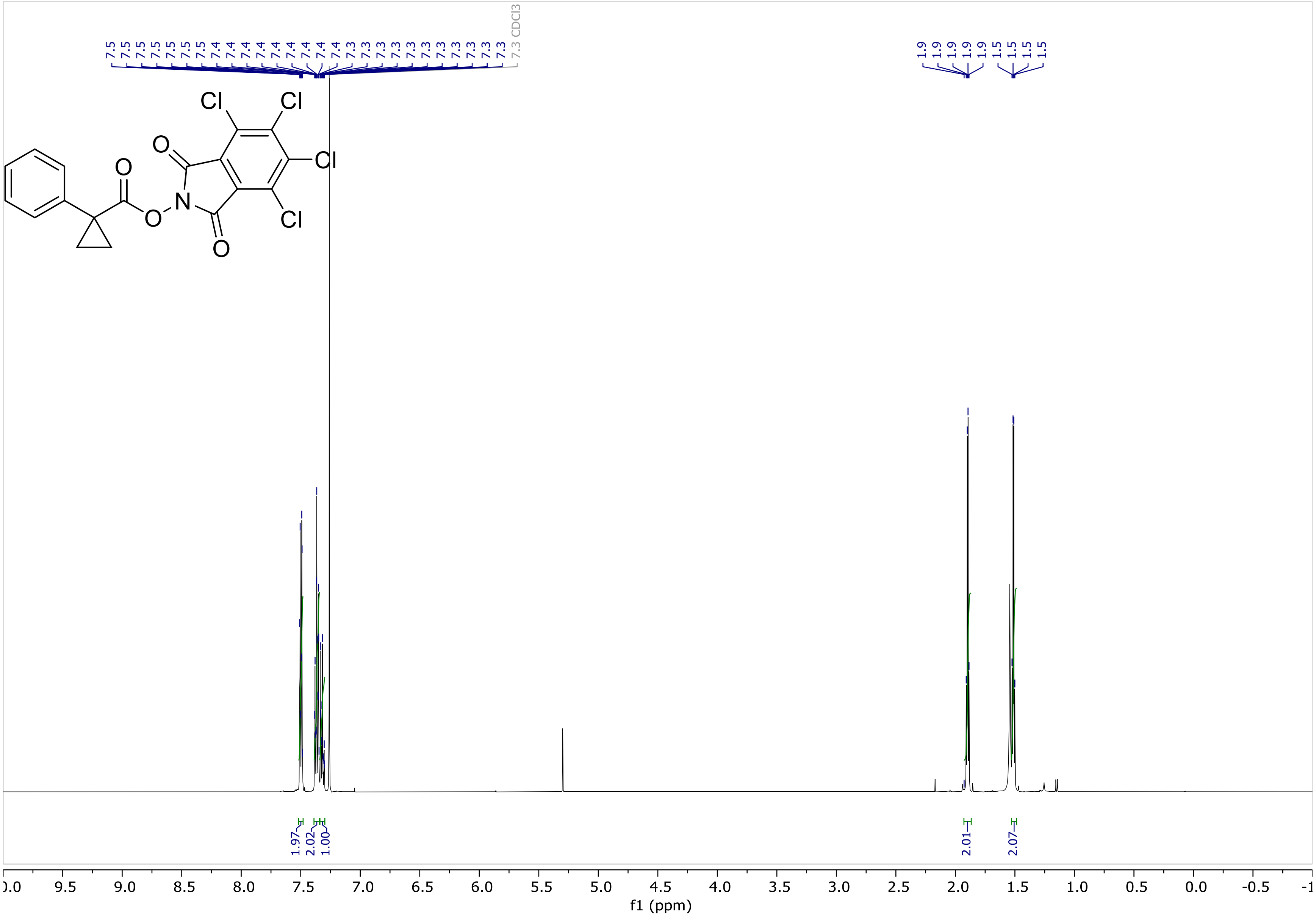

$^{13}C\{^{1}H\}$ NMR spectrum of compound 1c (126 MHz, $CDCl_3$)

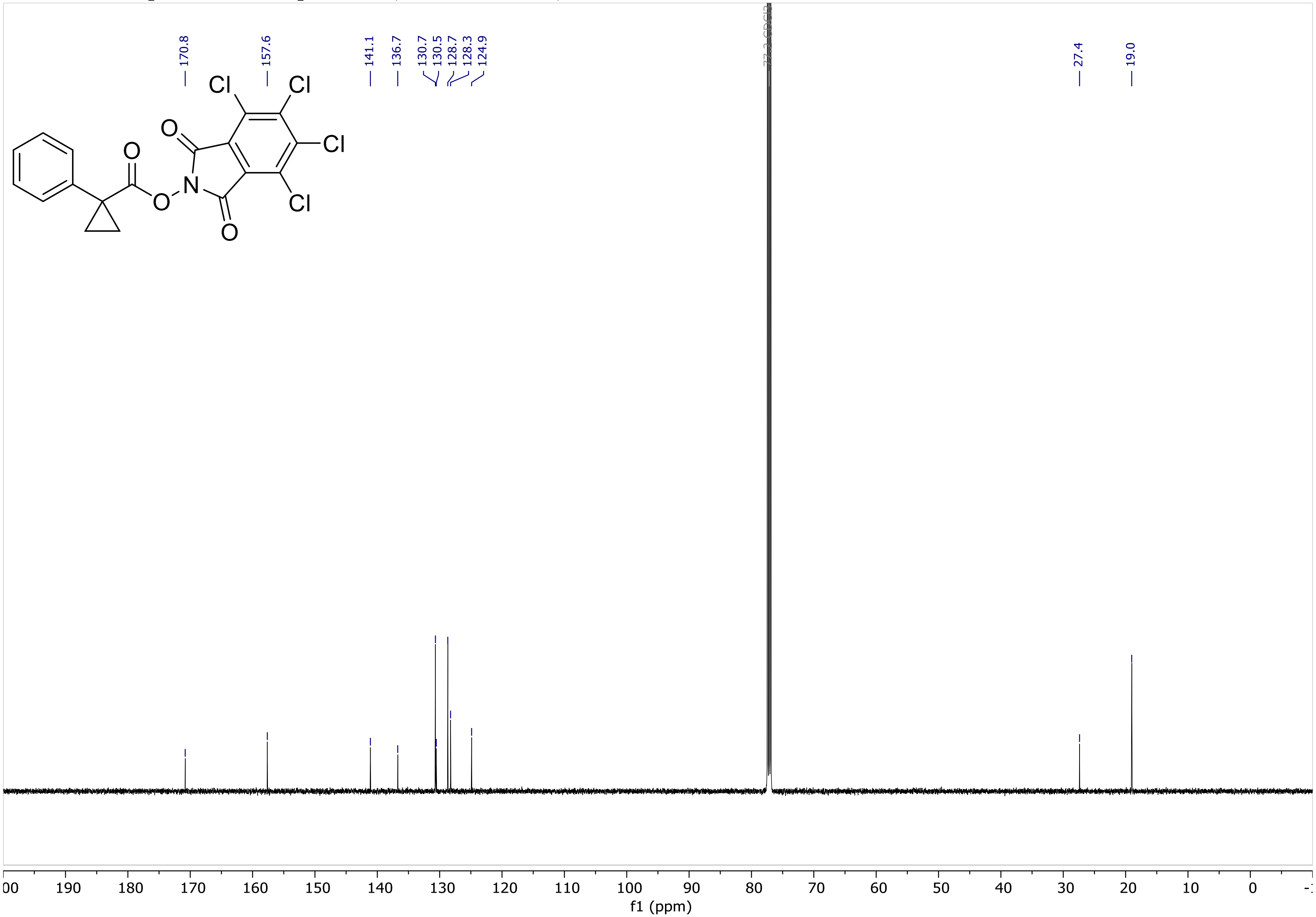

^{1}H NMR spectrum of compound 1d (500 MHz, CDCl$_3$)

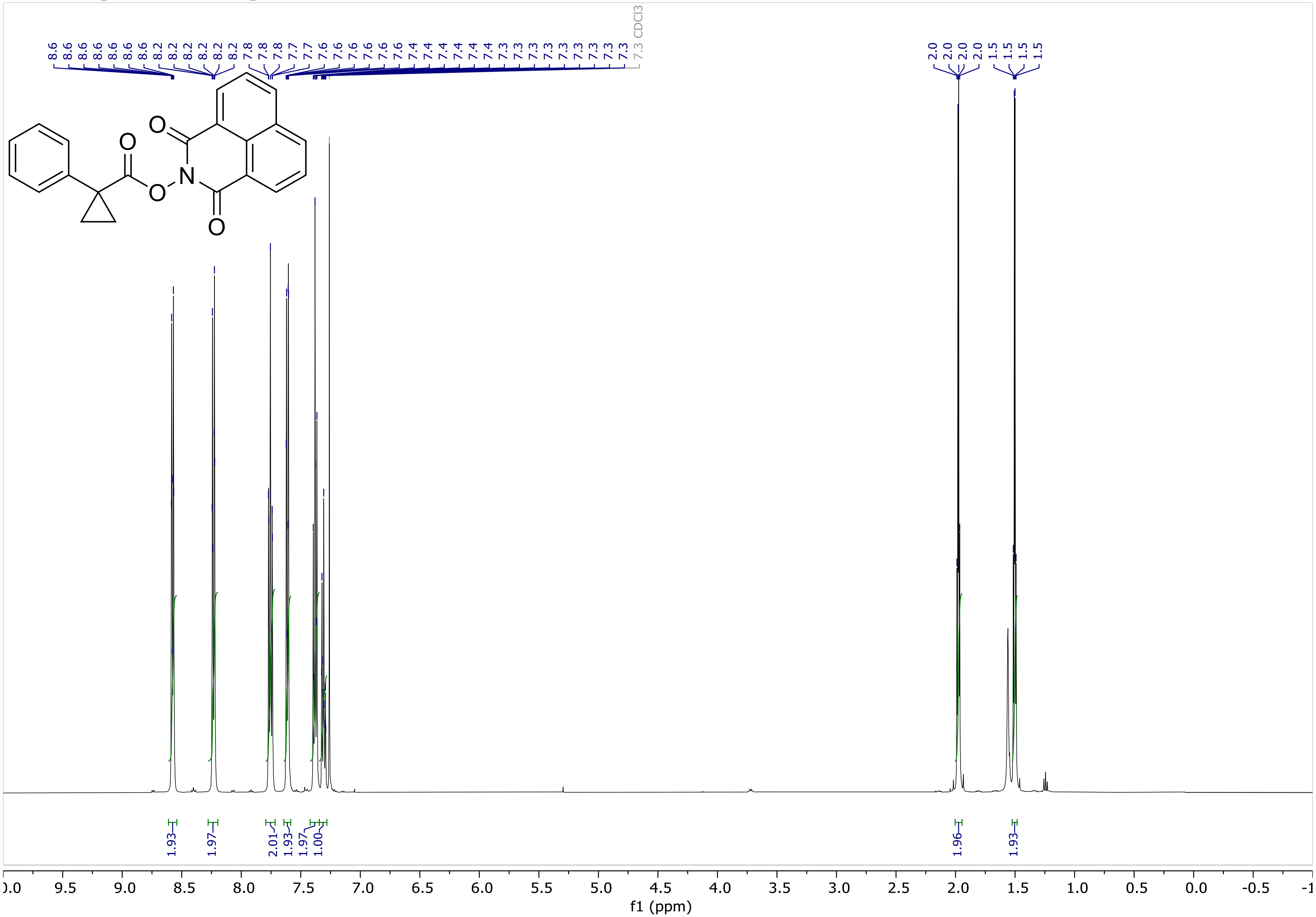

$^{13}C\{^{1}H\}$ NMR spectrum of compound 1d (126 MHz, $CDCl_3$)

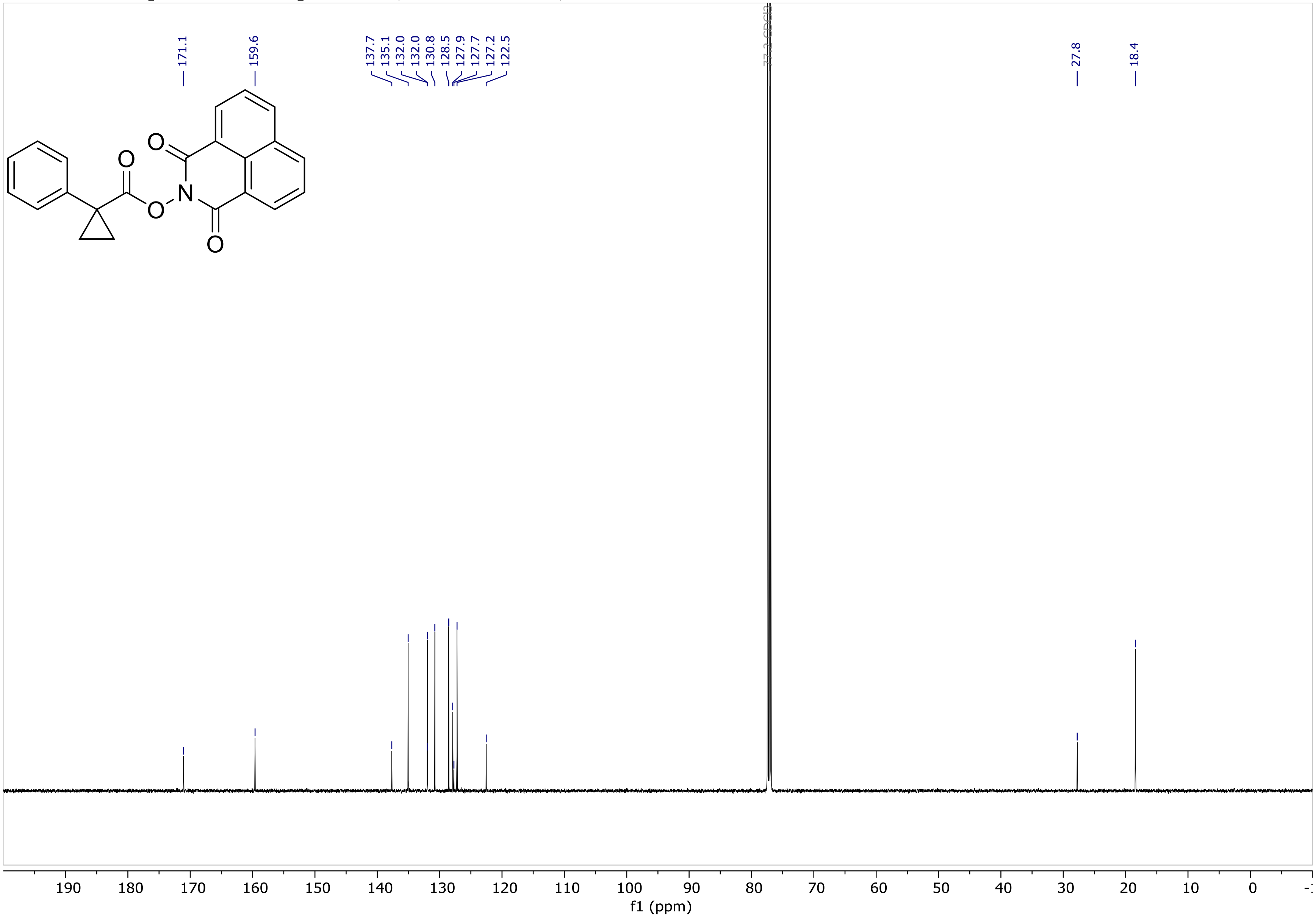

^{1}H NMR spectrum of compound 1e (500 MHz, $CDCl_3$)

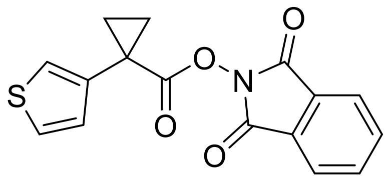

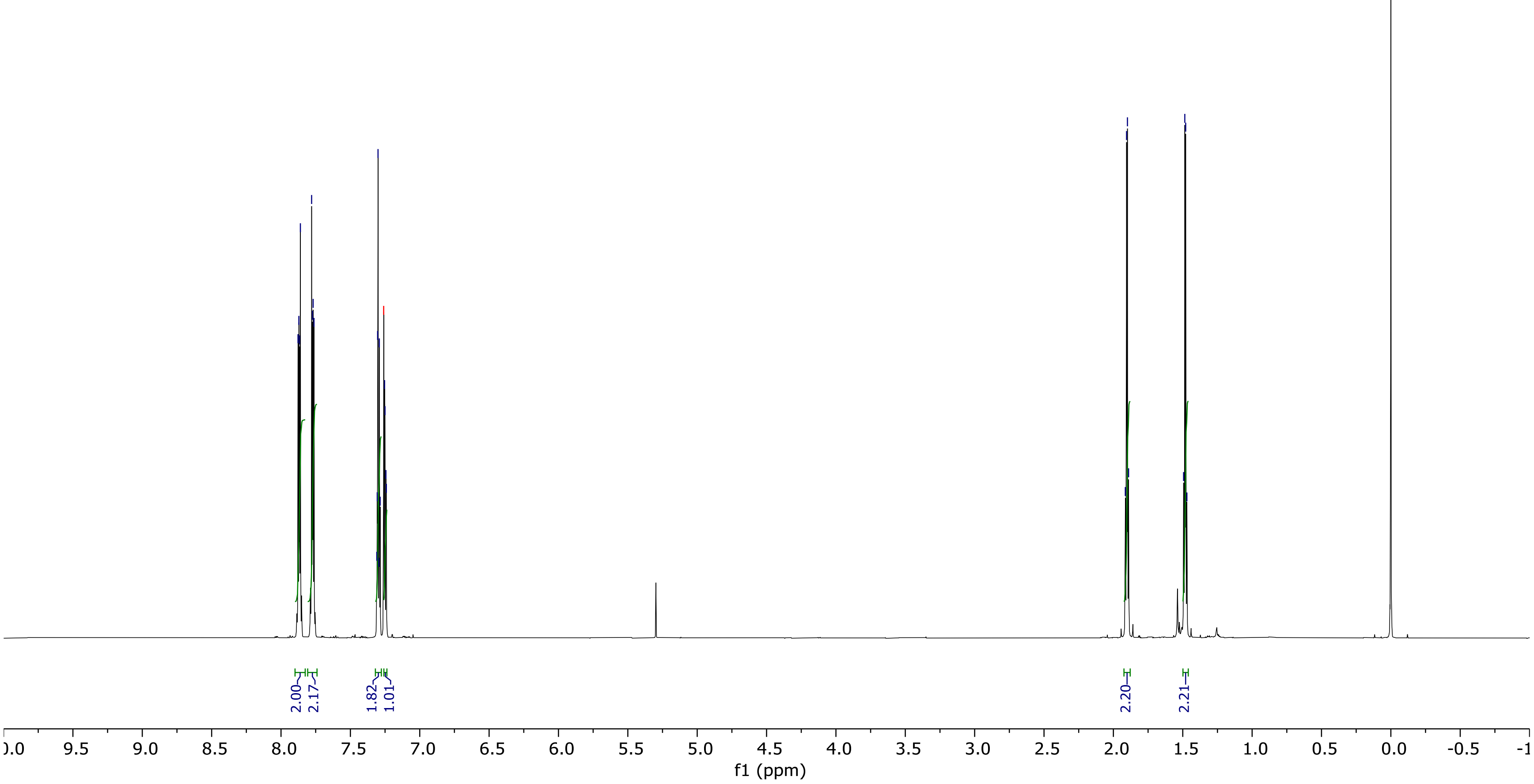

$^{13}C\{^1H\}$ NMR spectrum of compound 1e (126 MHz, $CDCl_3$)

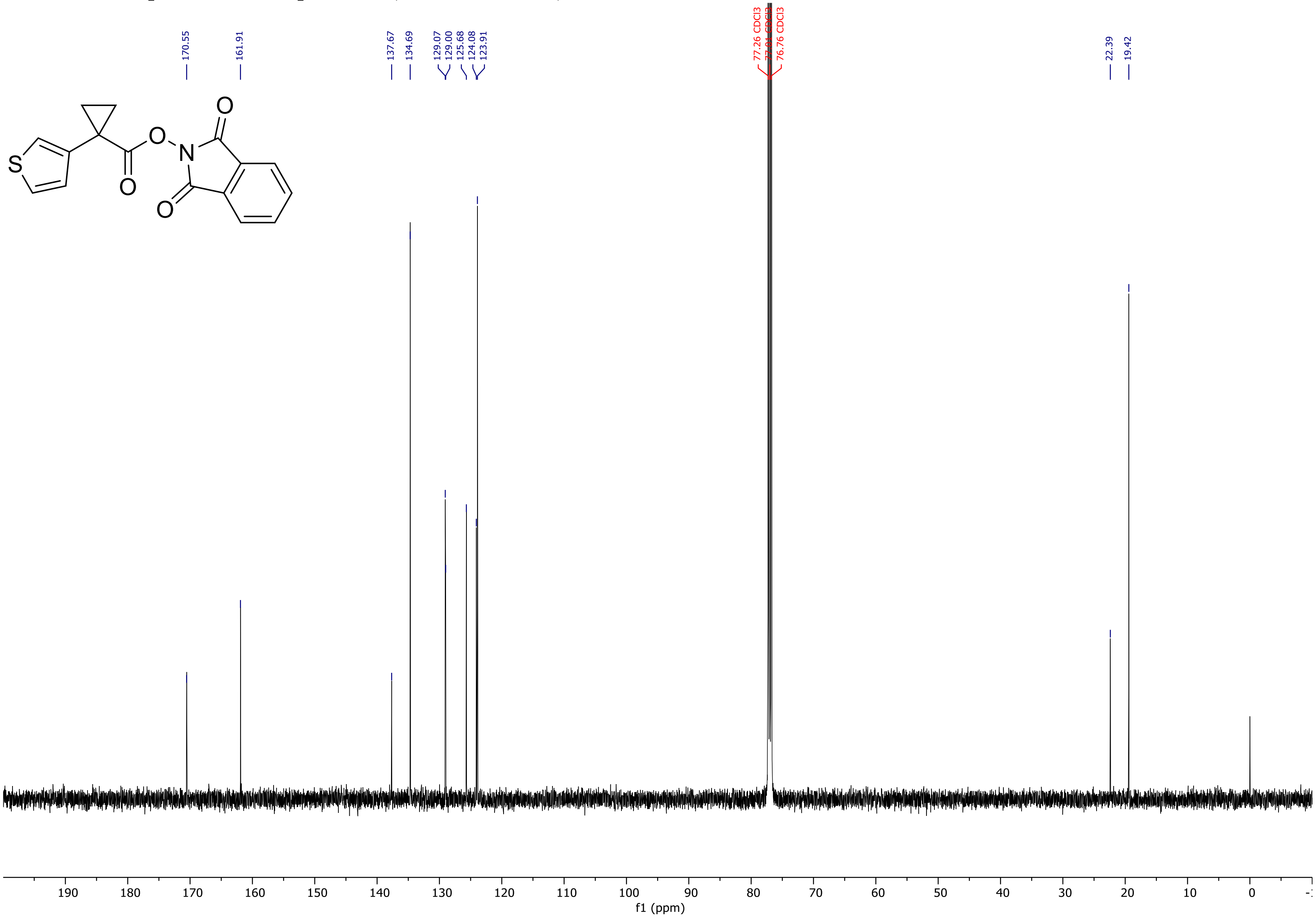

^{1}H NMR spectrum of compound 1f (500 MHz, $CDCl_3$)

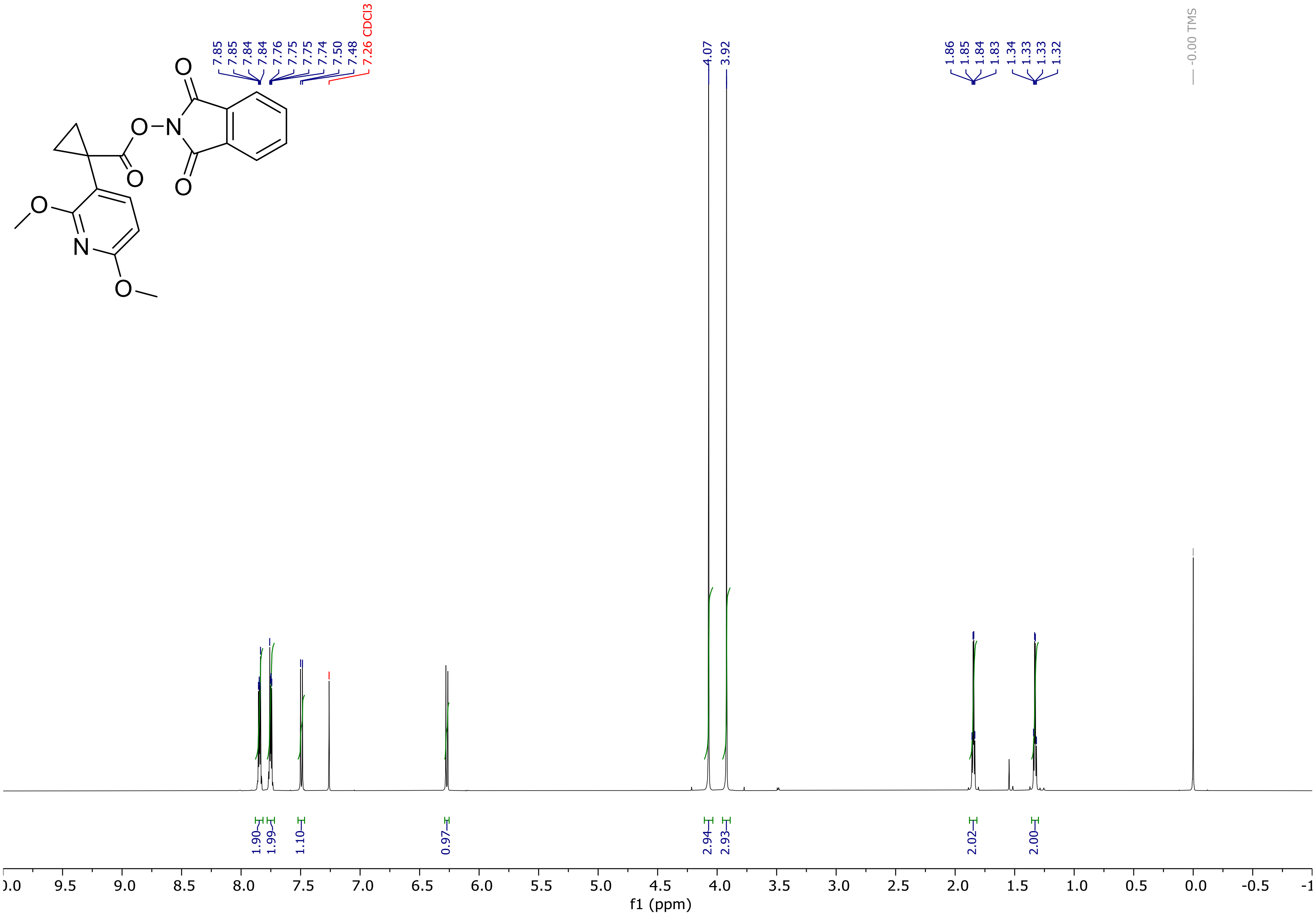

$^{13}C\{^1H\}$ **NMR spectrum of compound 1f (126 MHz, $CDCl_3$)**

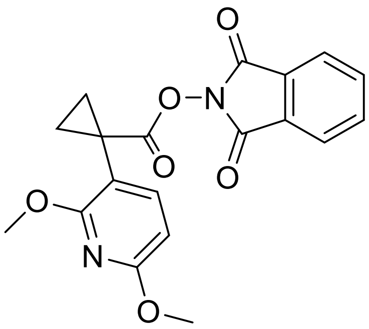

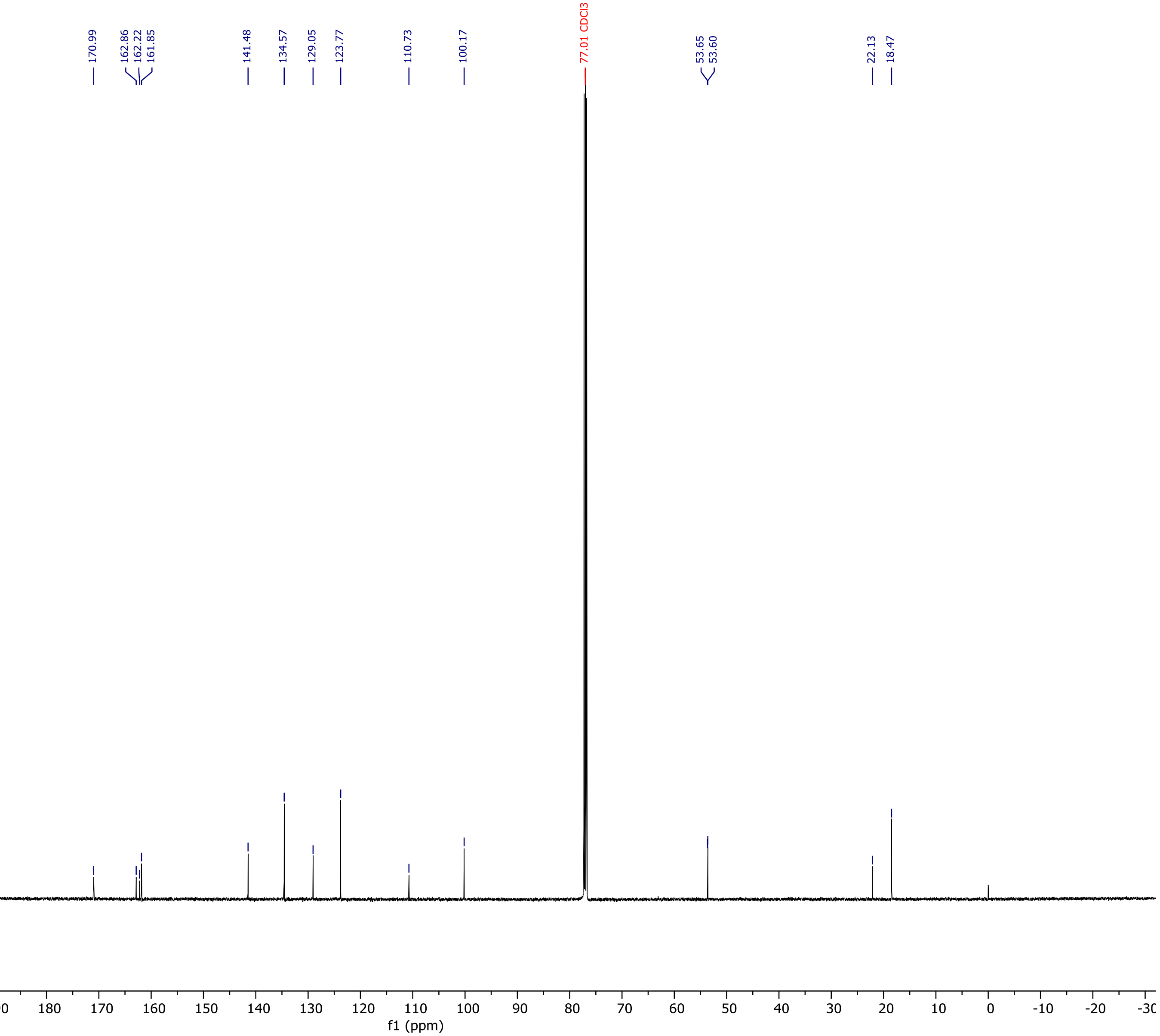

^{1}H NMR spectrum of compound 1g (500 MHz, $CDCl_3$)

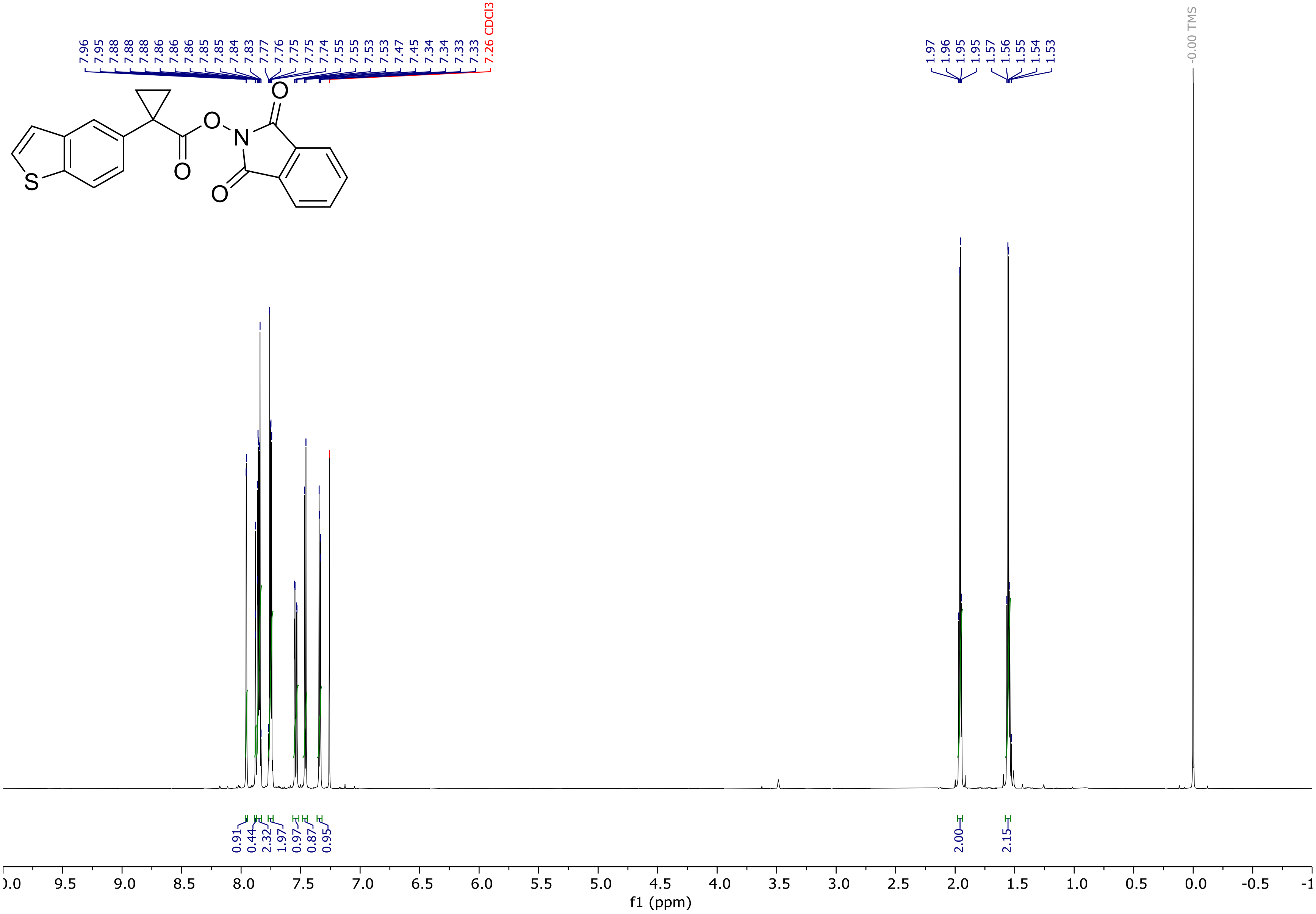

$^{13}C\{^{1}H\}$ NMR spectrum of compound 1g (126 MHz, $CDCl_3$)

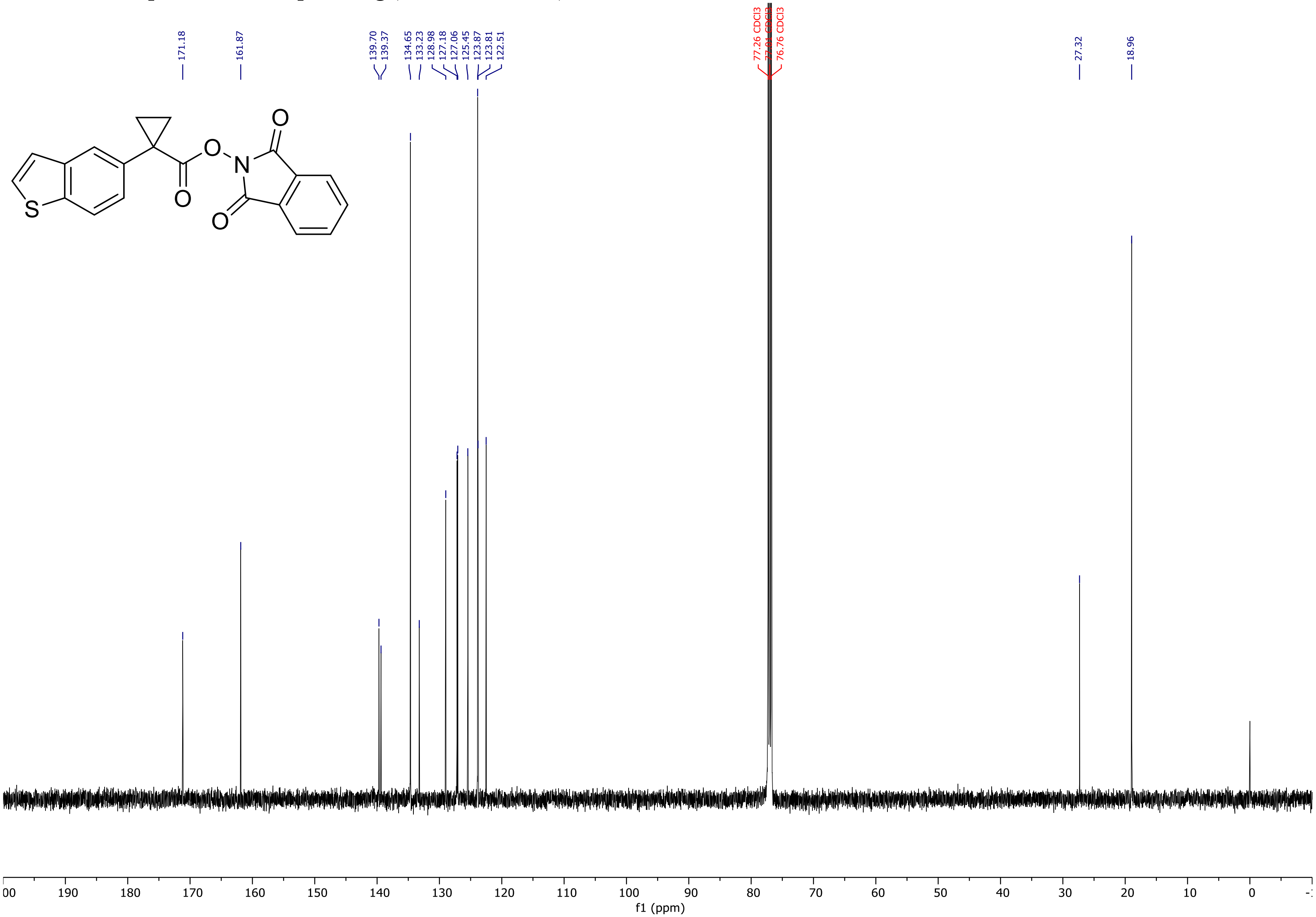

^{1}H NMR spectrum of compound 1h (500 MHz, $CDCl_3$)

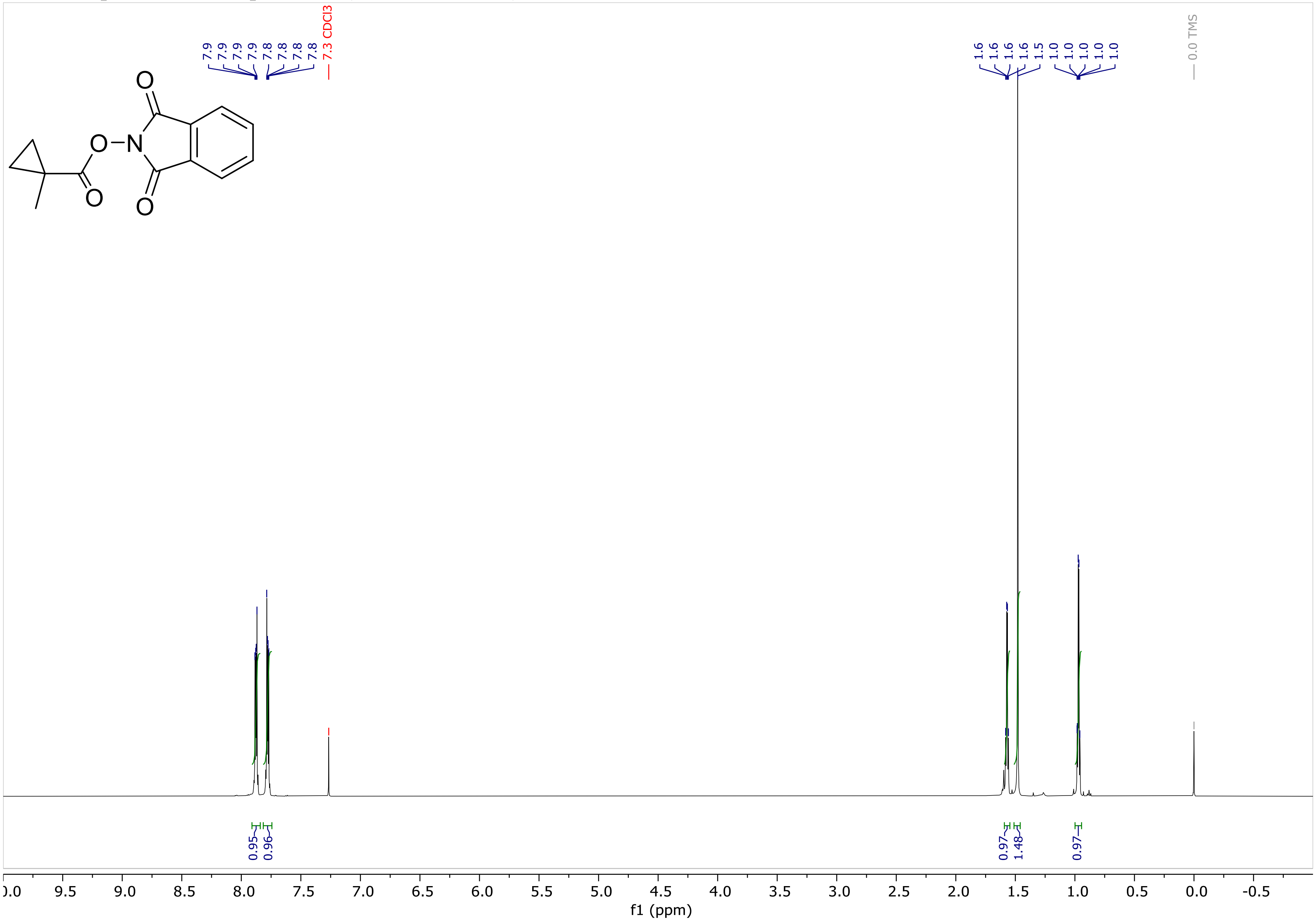

$^{13}C\{^1H\}$ NMR spectrum of compound 1h (126 MHz, $CDCl_3$)

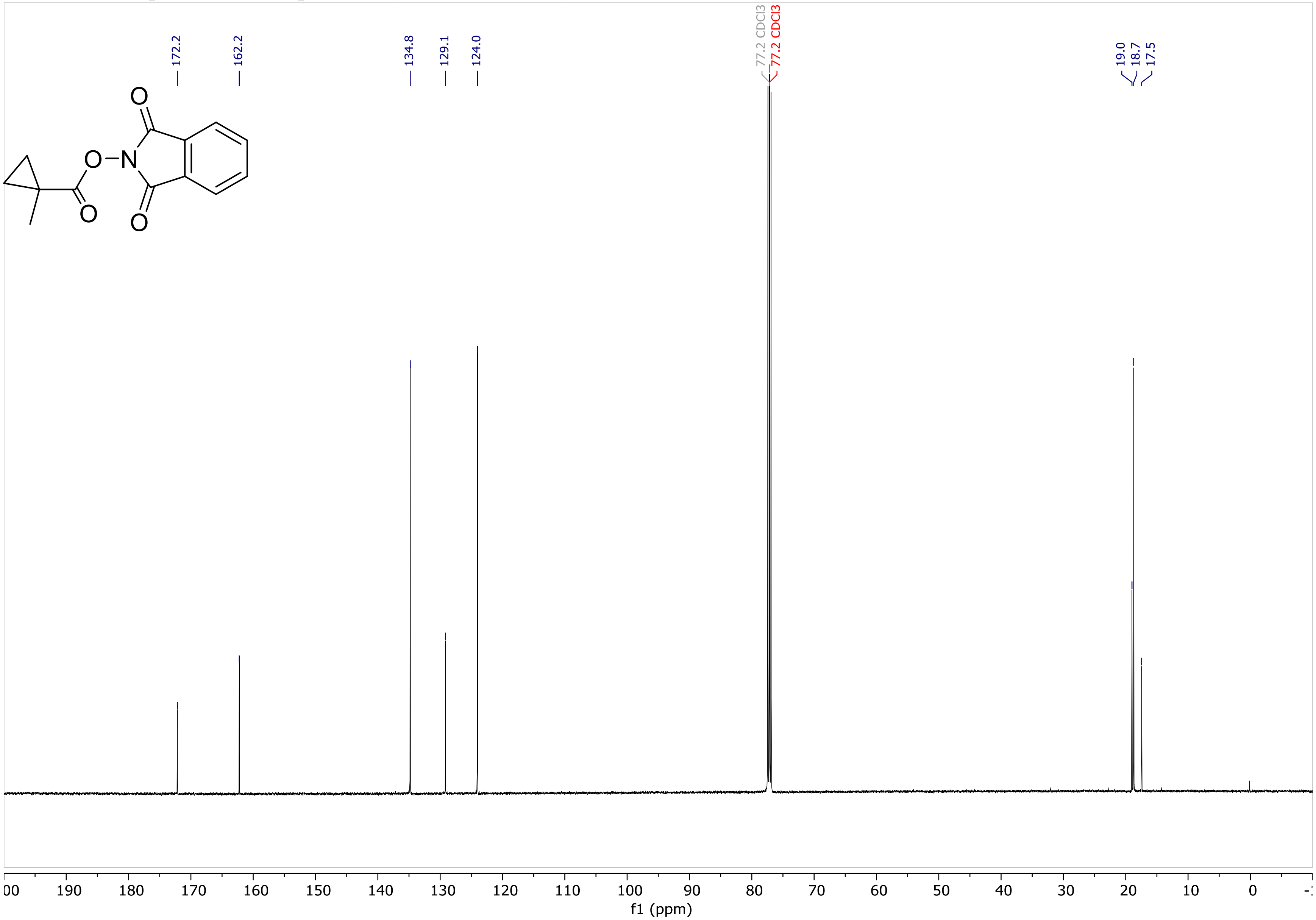

^{1}H NMR spectrum of compound 1i (500 MHz, $CDCl_3$)

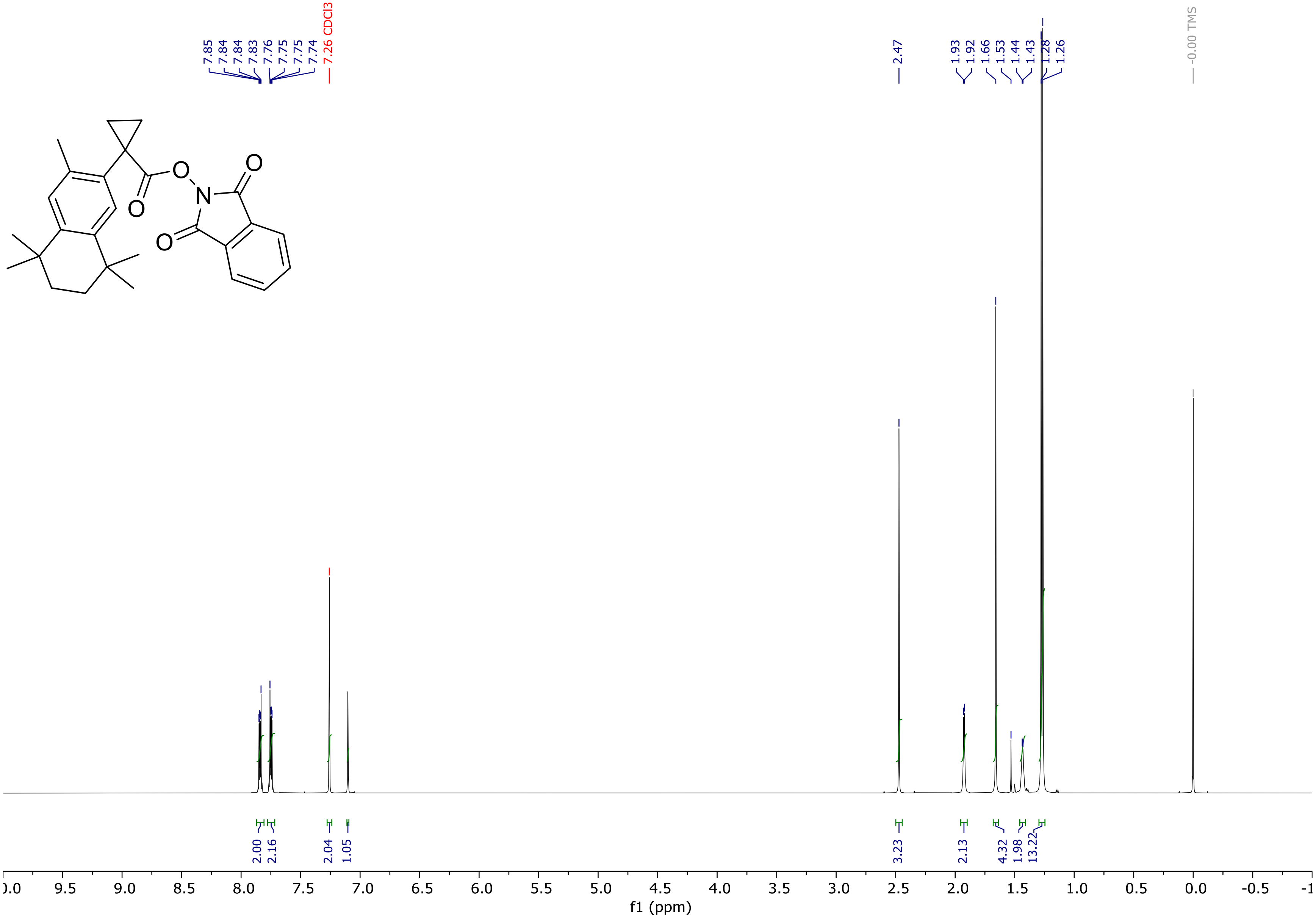

$^{13}C\{^1H\}$ NMR spectrum of compound 1i (126 MHz, $CDCl_3$)

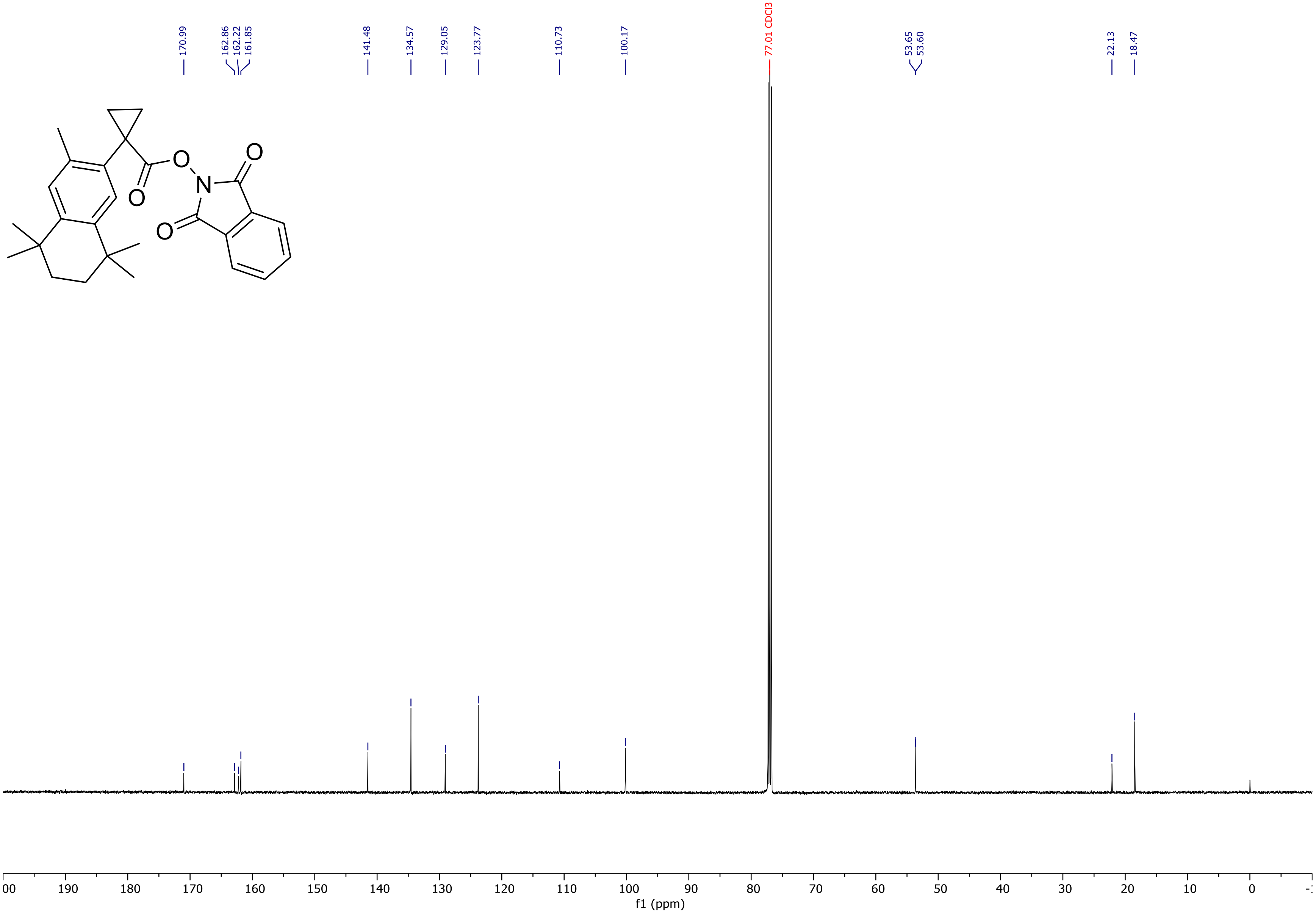

^{1}H NMR spectrum of compound 1j (600 MHz, $CDCl_3$)

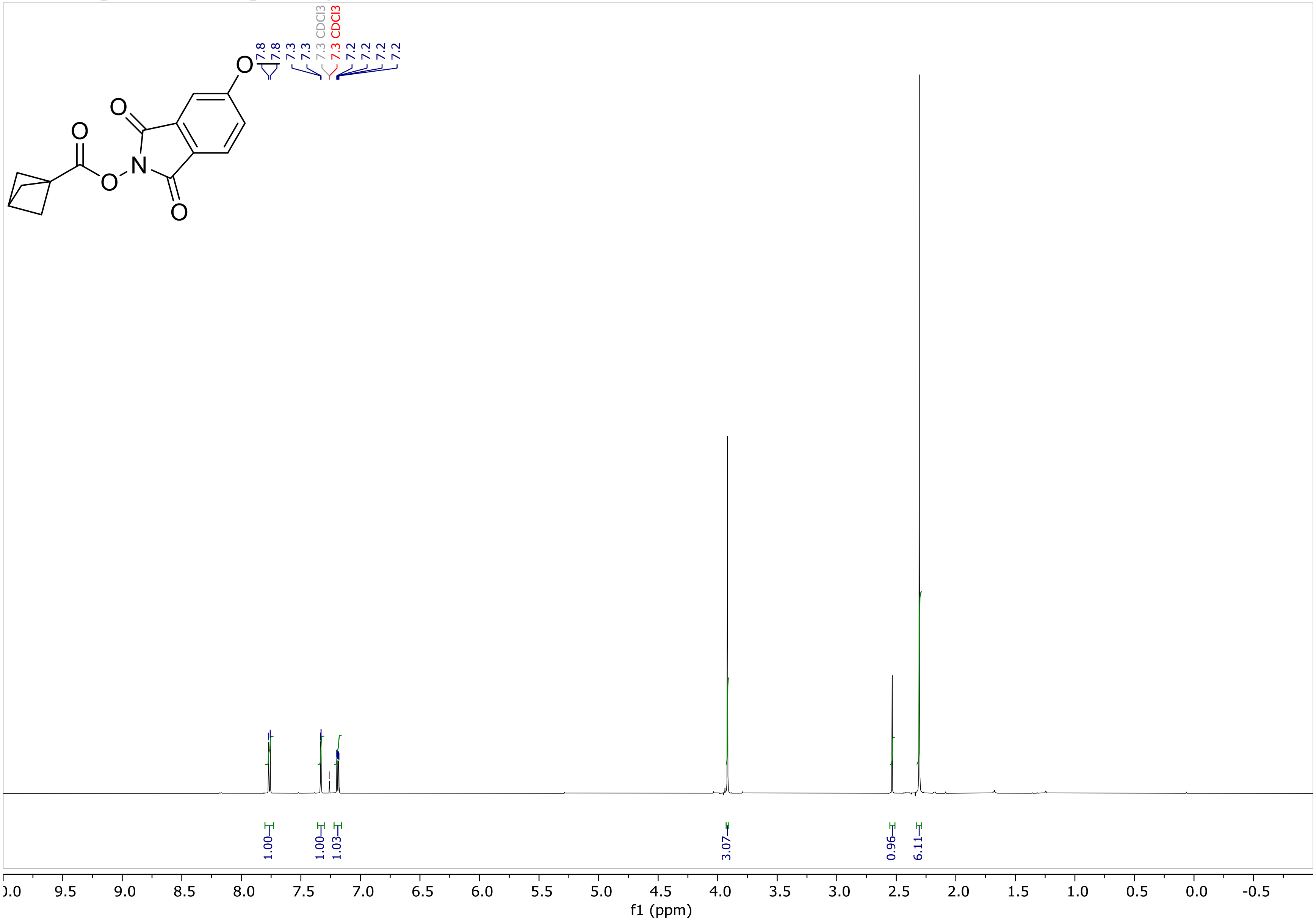

$^{13}C\{^{1}H\}$ NMR spectrum of compound 1j (151 MHz, $CDCl_3$)

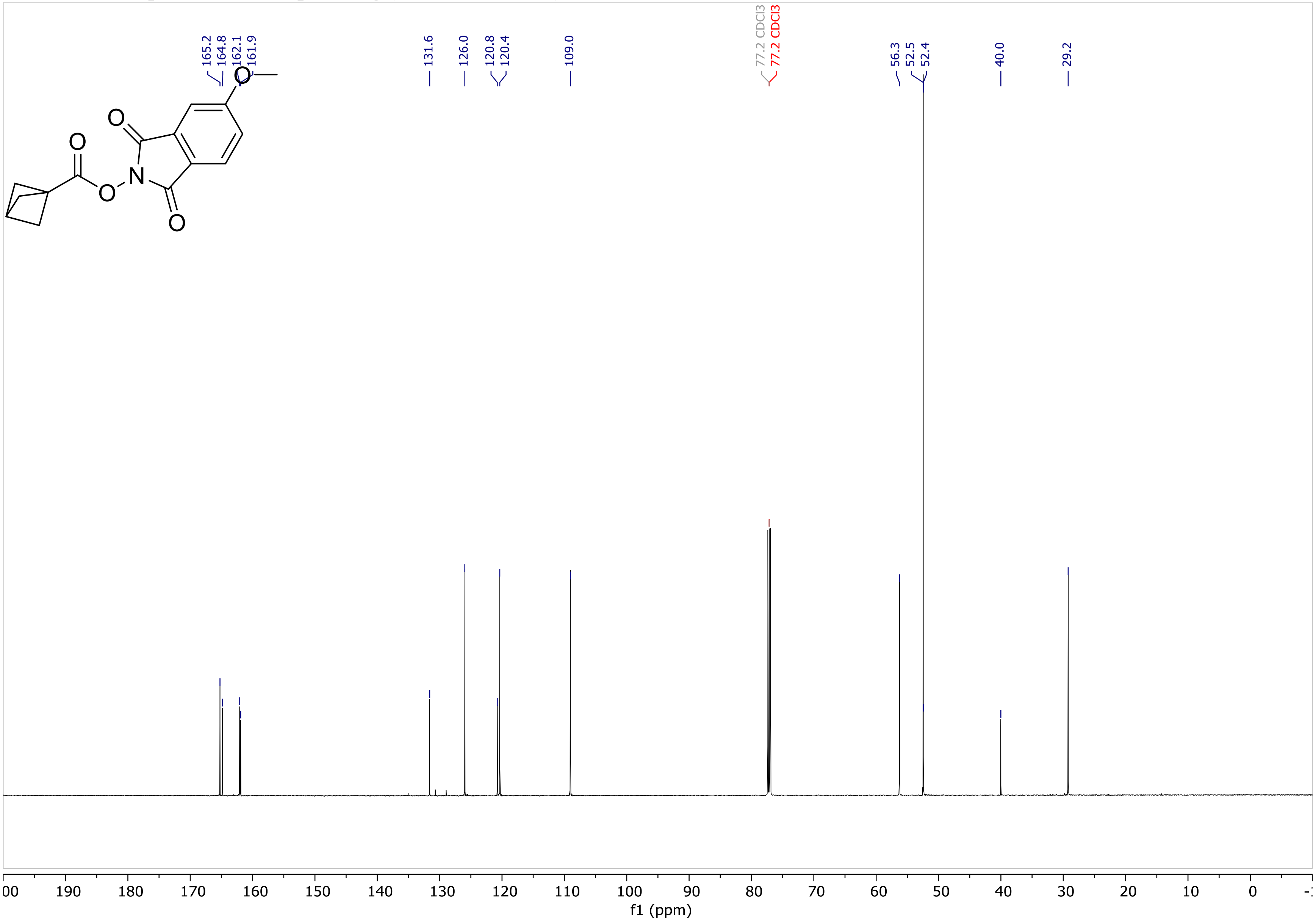

^{1}H NMR spectrum of compound 1k (600 MHz, $CDCl_3$)

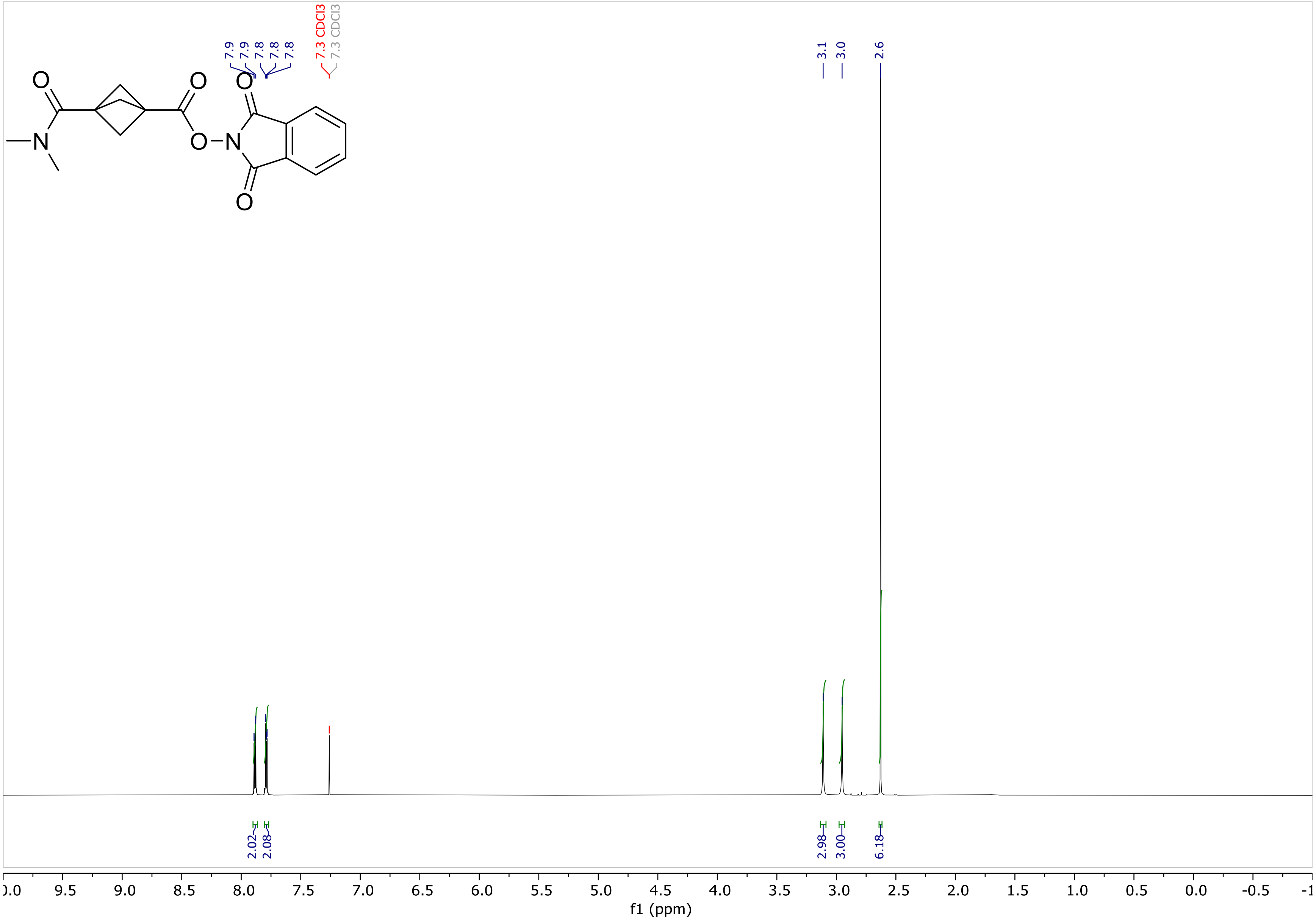

$^{13}C\{^1H\}$ NMR spectrum of compound 1k (151 MHz, $CDCl_3$)

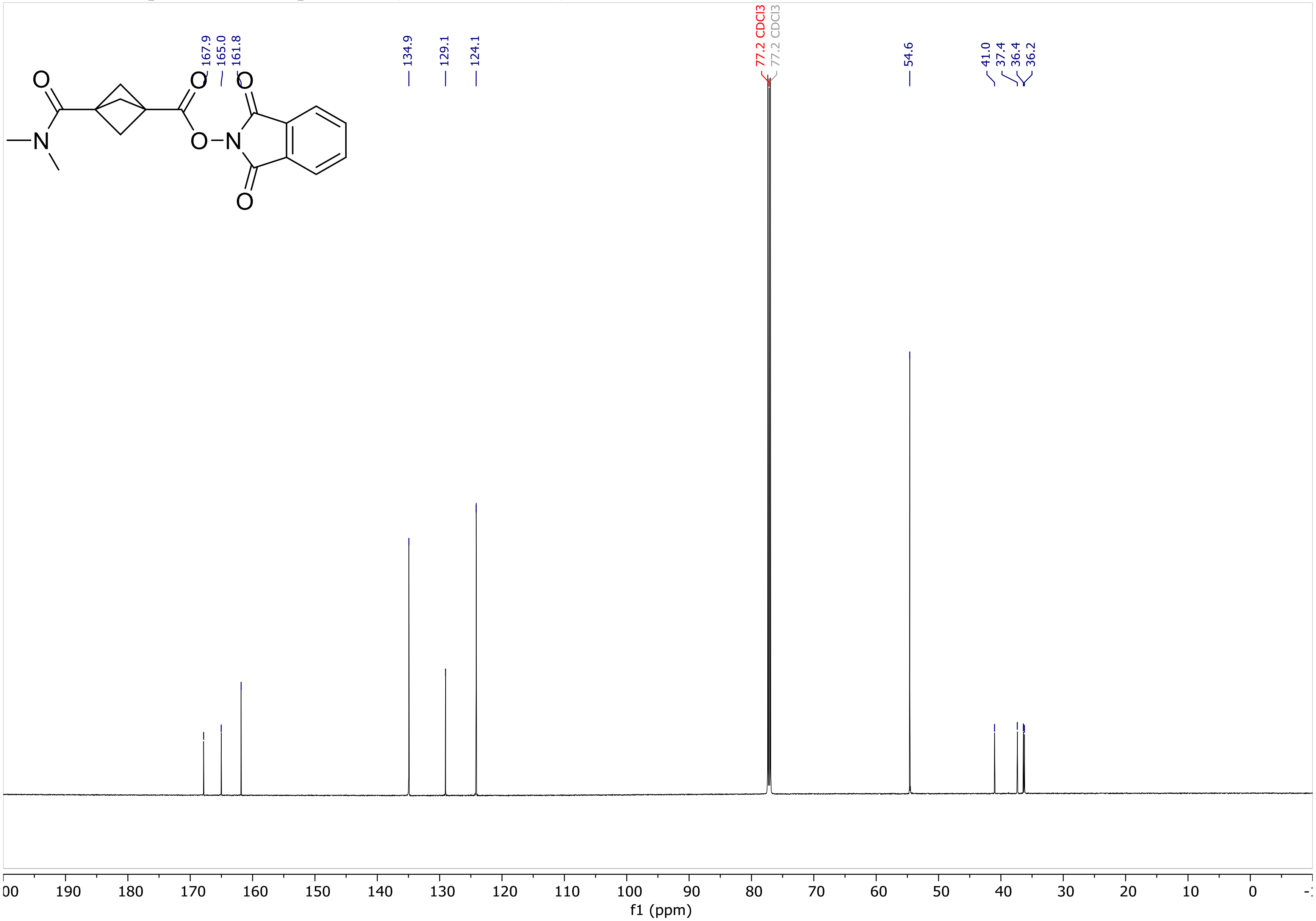

^{1}H NMR spectrum of compound 1l (500 MHz, $CDCl_3$)

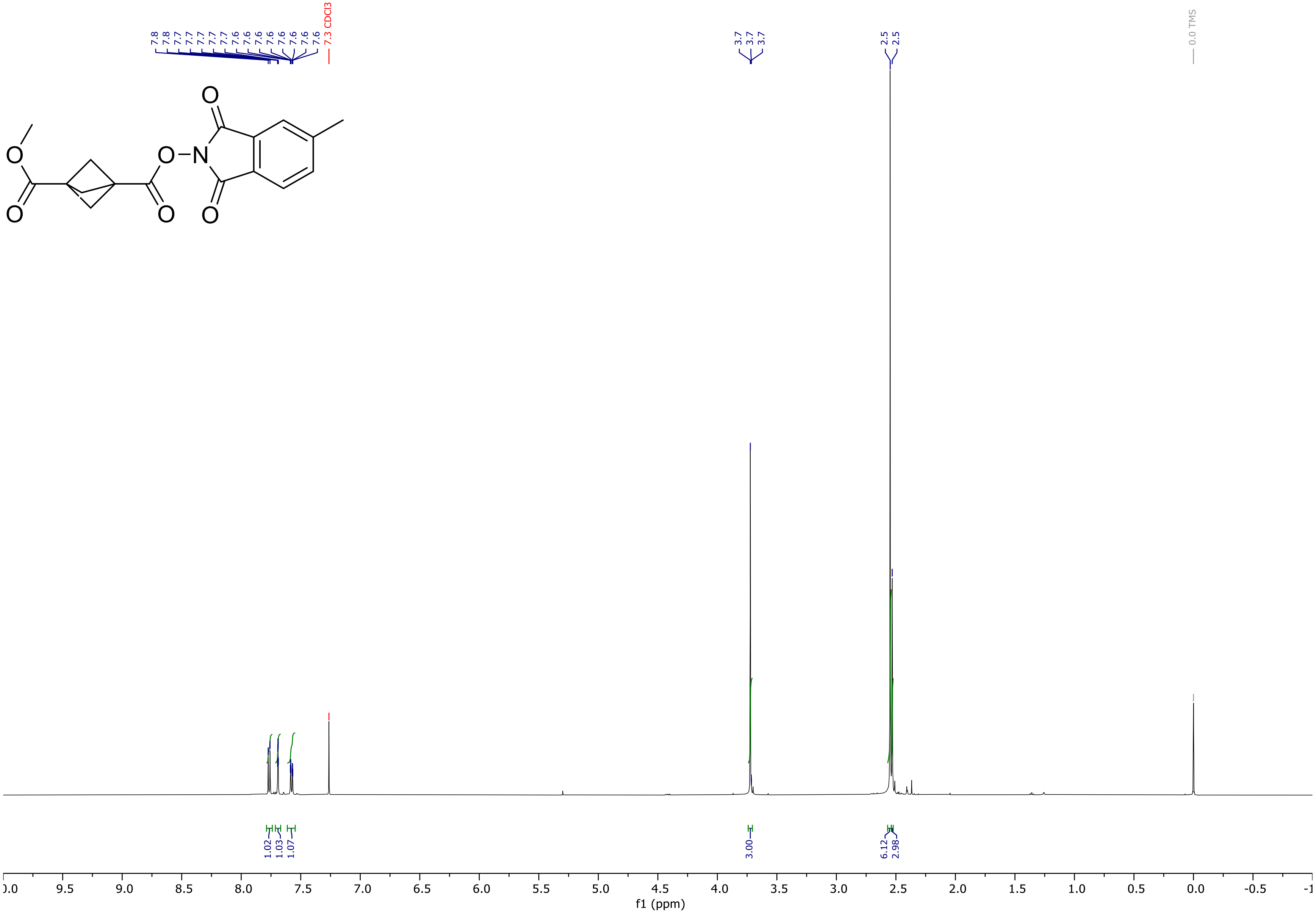

$^{13}C\{^1H\}$ NMR spectrum of compound 1l (126 MHz, $CDCl_3$)

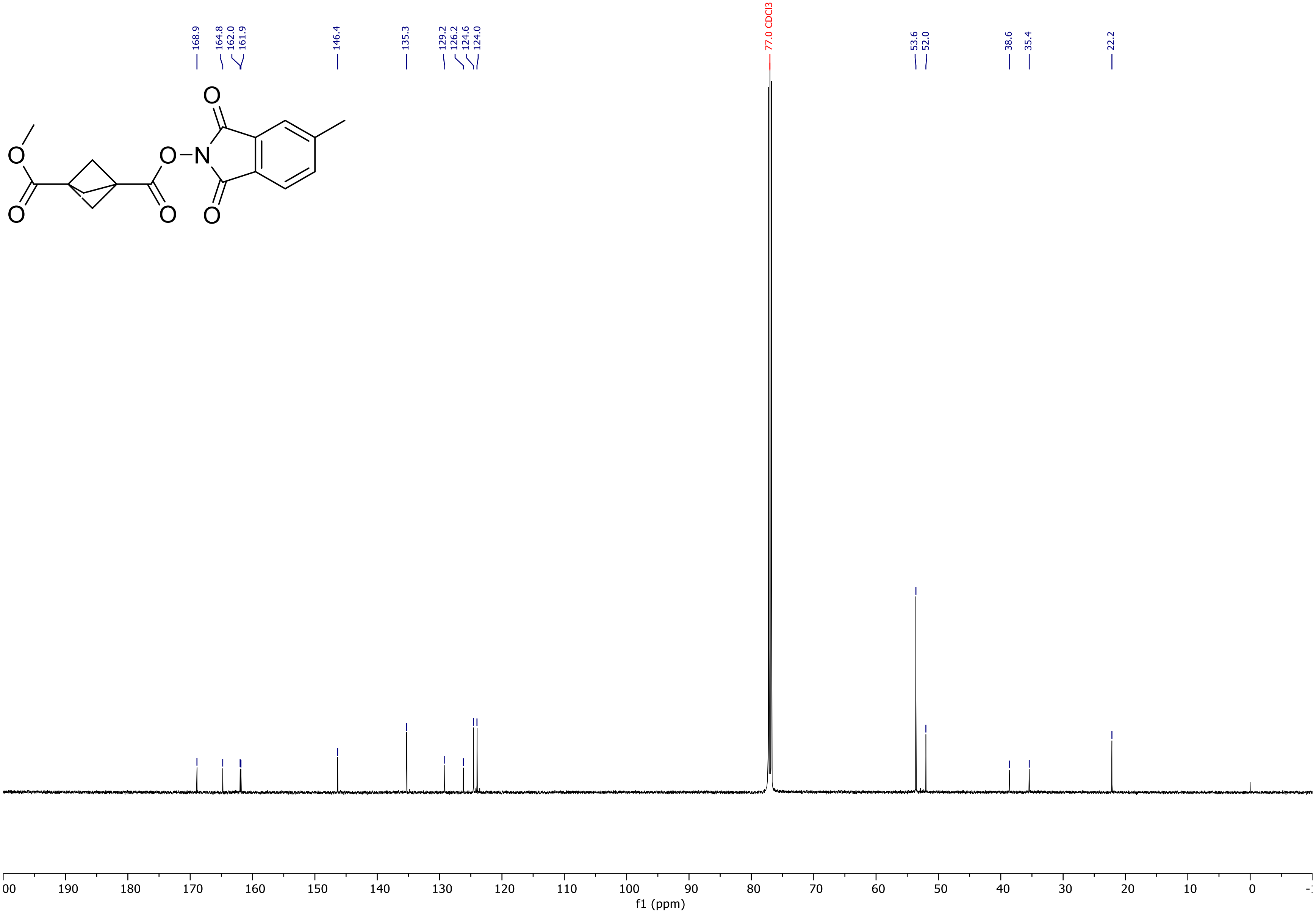

^{1}H NMR spectrum of compound 1m (500 MHz, CDCl$_3$)

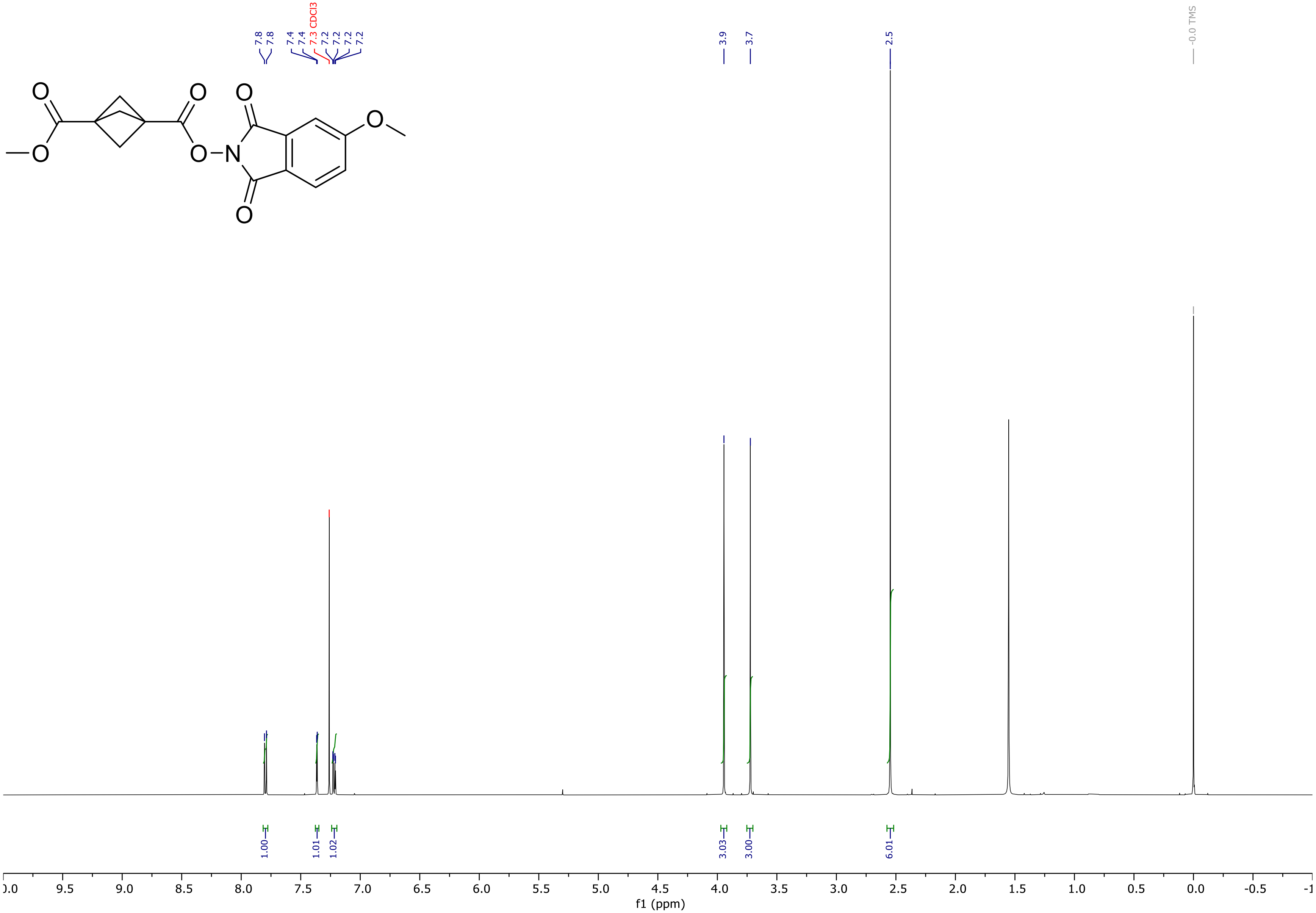

$^{13}C\{^{1}H\}$ NMR spectrum of compound 1m (126 MHz, $CDCl_3$)

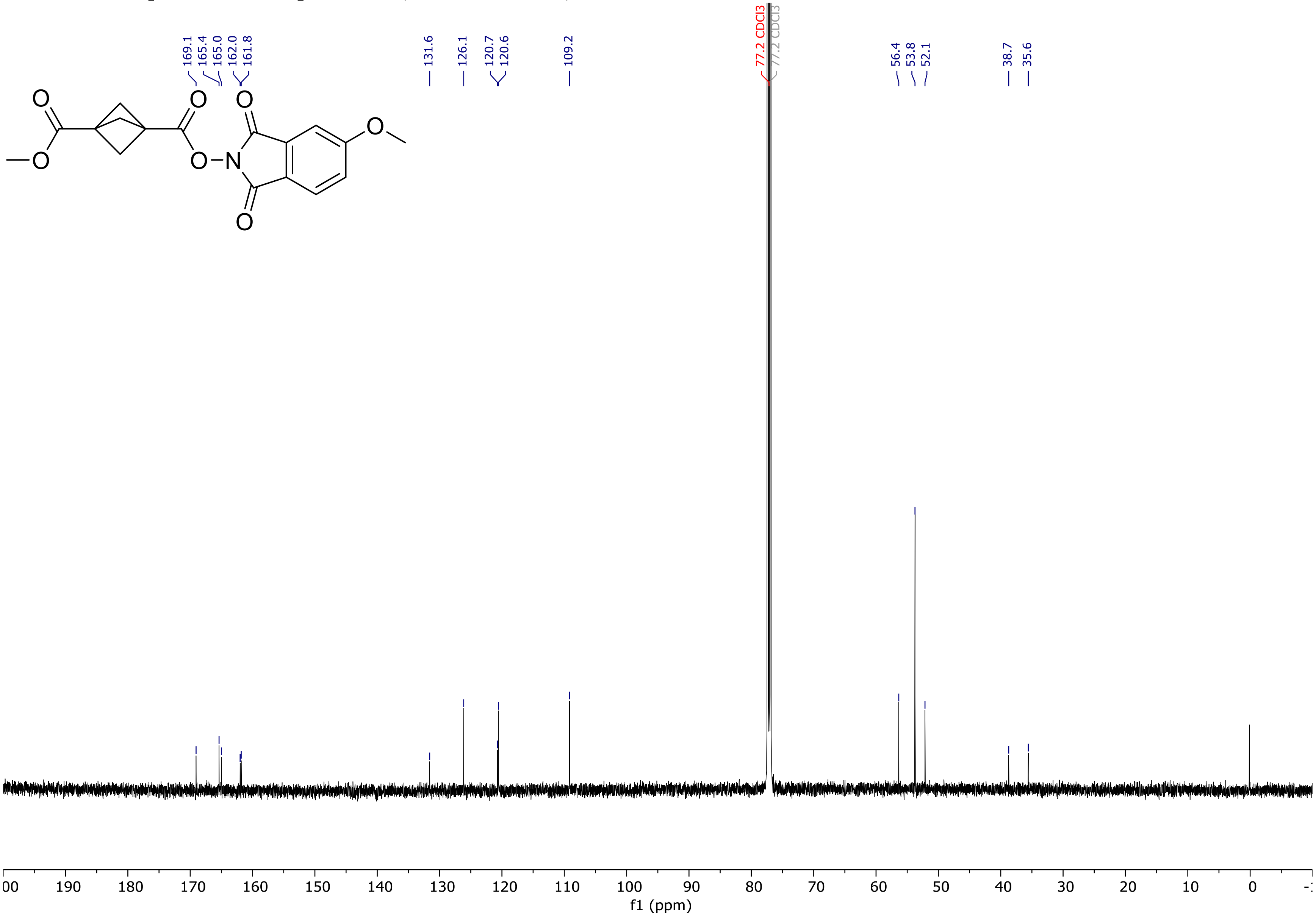

^{1}H NMR spectrum of compound 1n (600 MHz, $CDCl_3$)

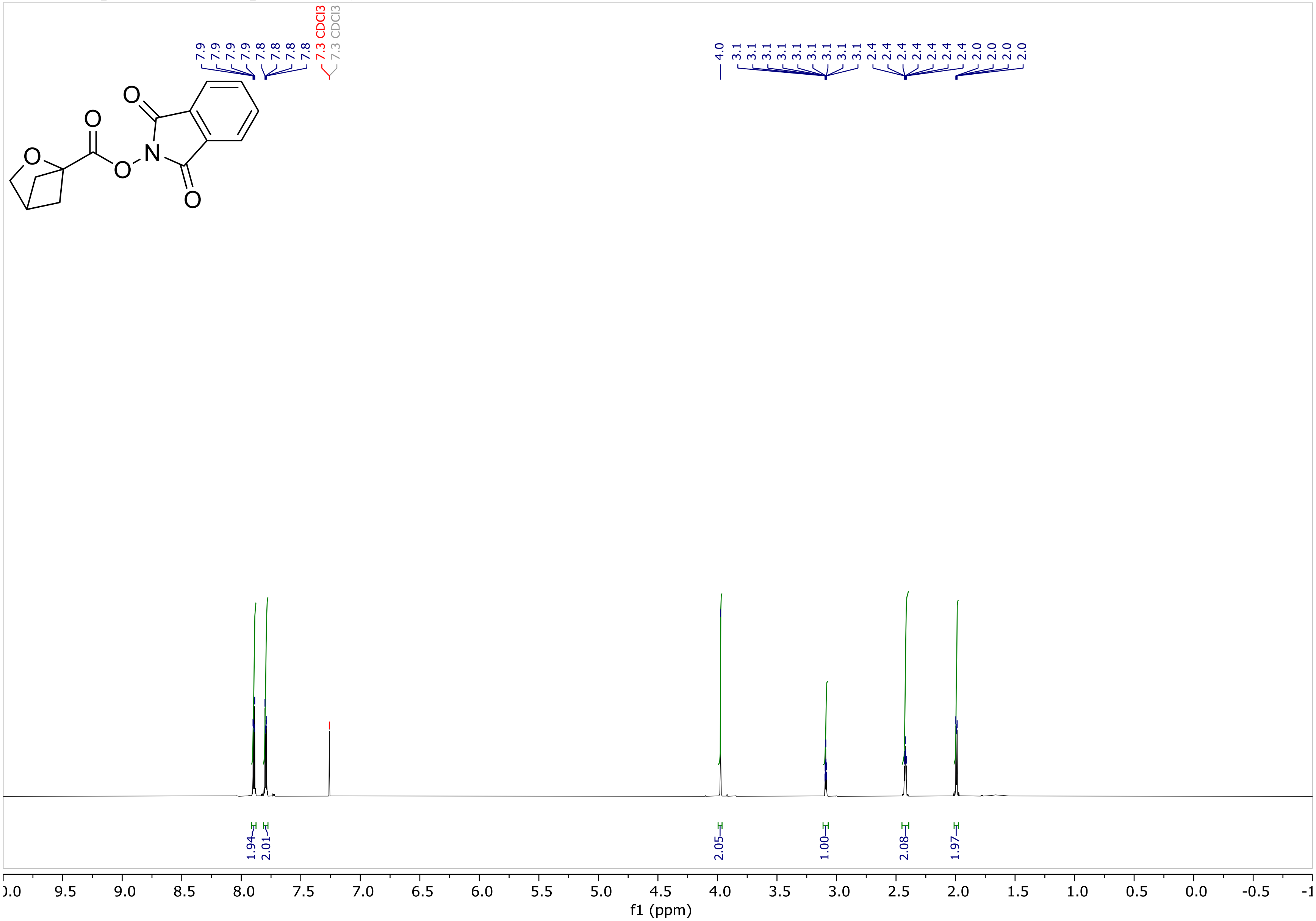

$^{13}C\{^{1}H\}$ NMR spectrum of compound 1n (151 MHz, $CDCl_3$)

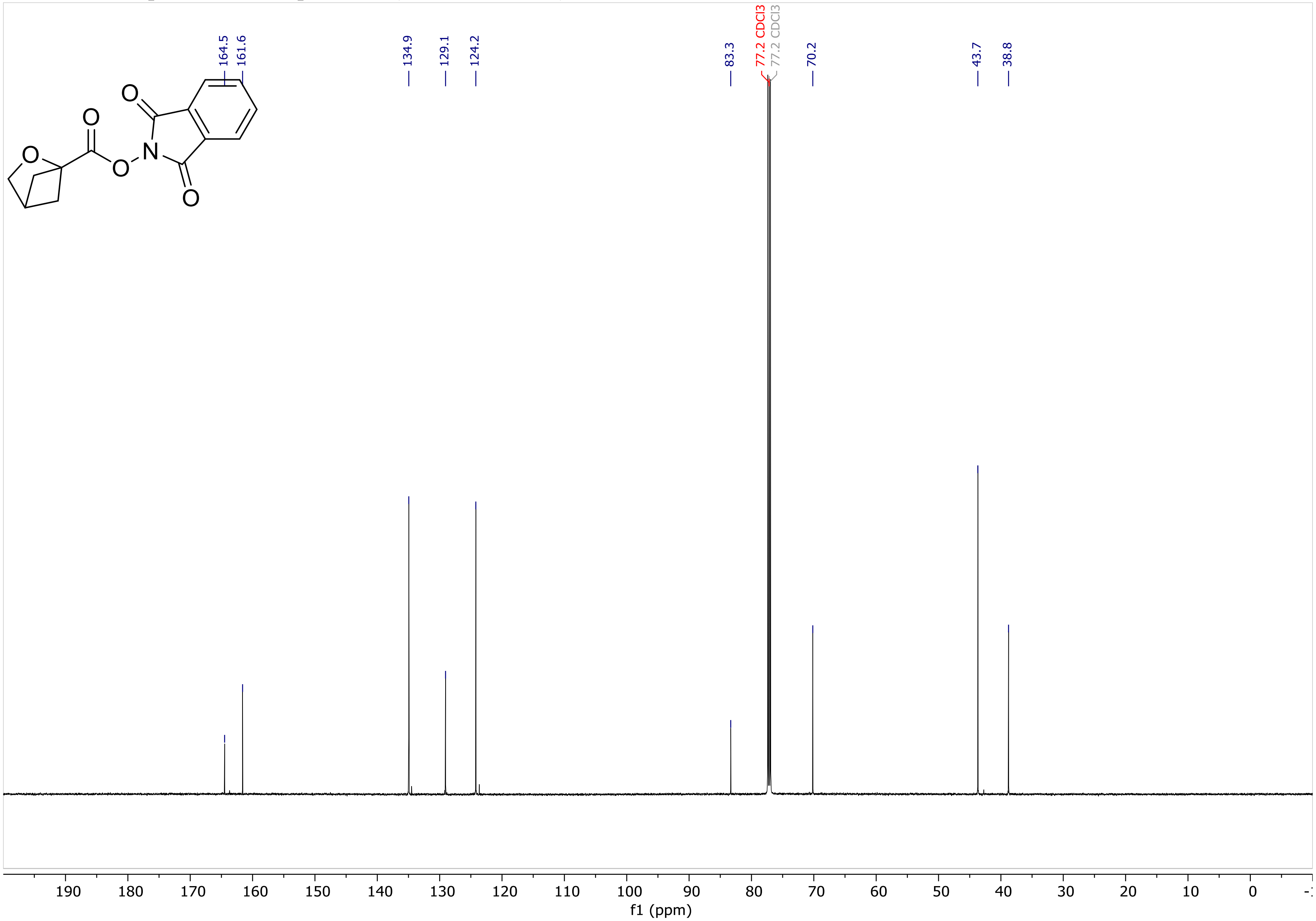

^{1}H NMR spectrum of compound 1o (600 MHz, $CDCl_3$)

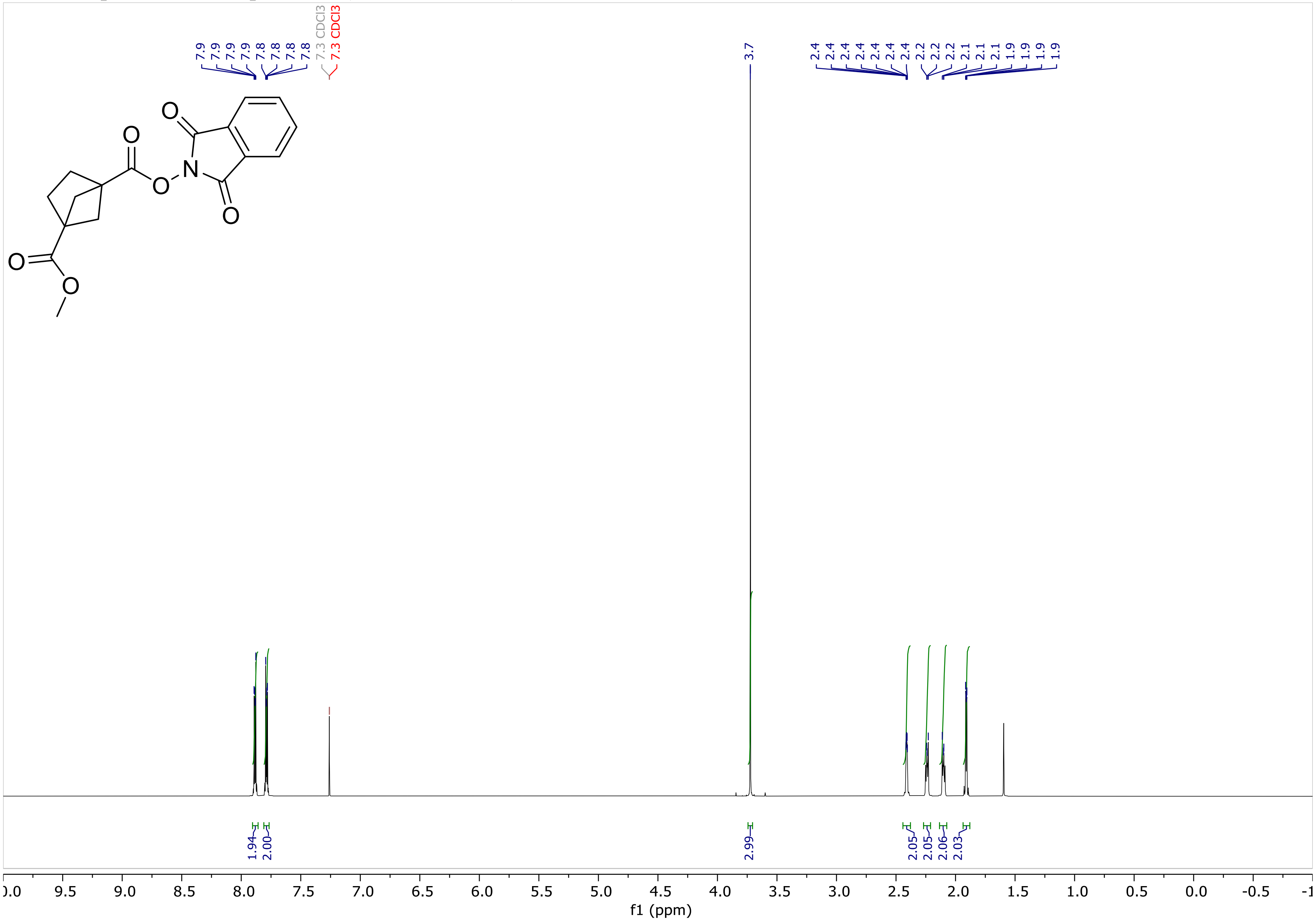

$^{13}C\{^{1}H\}$ NMR spectrum of compound 1o (151 MHz, $CDCl_3$)

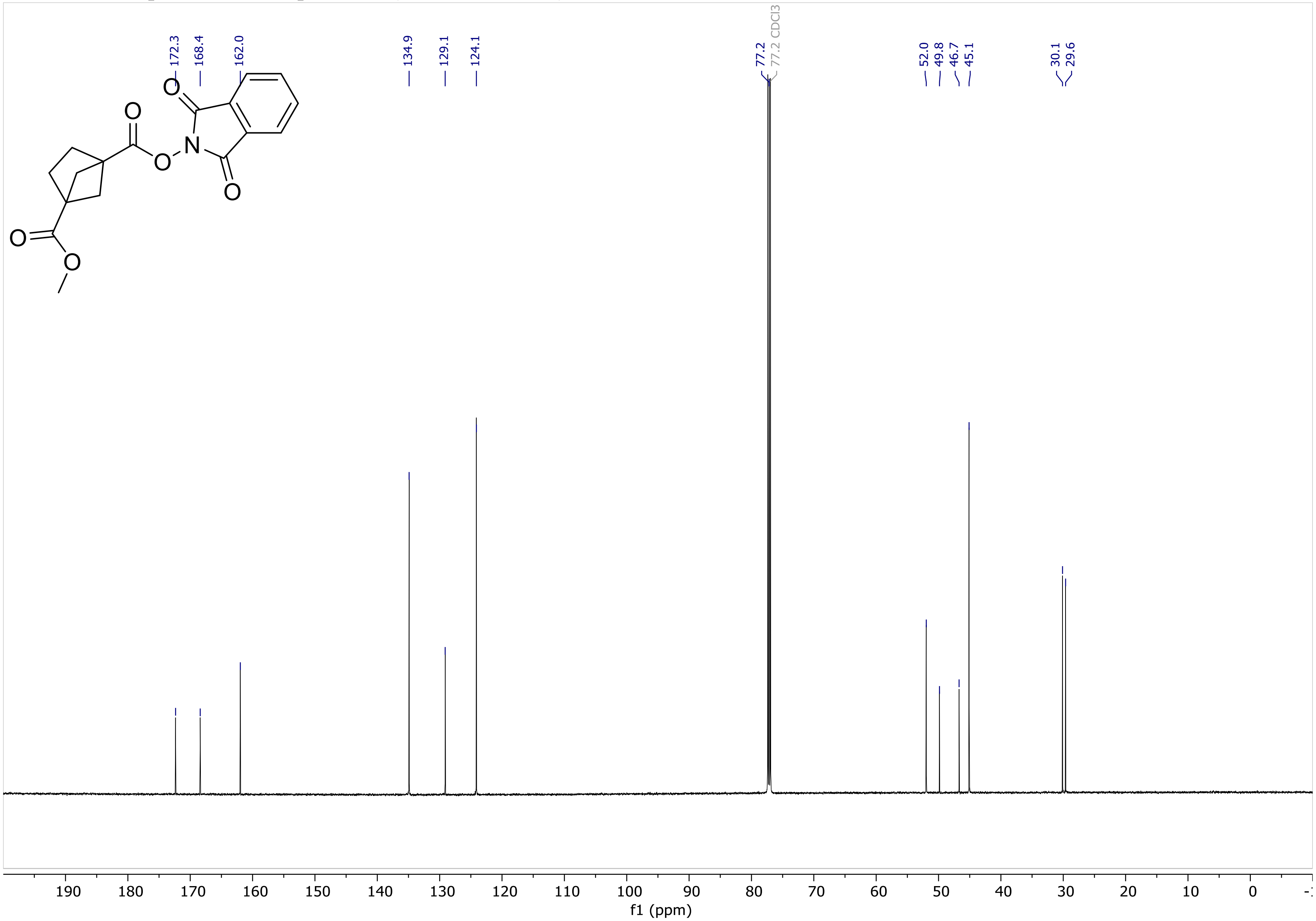

^{1}H NMR spectrum of compound 1p (600 MHz, $CDCl_3$)

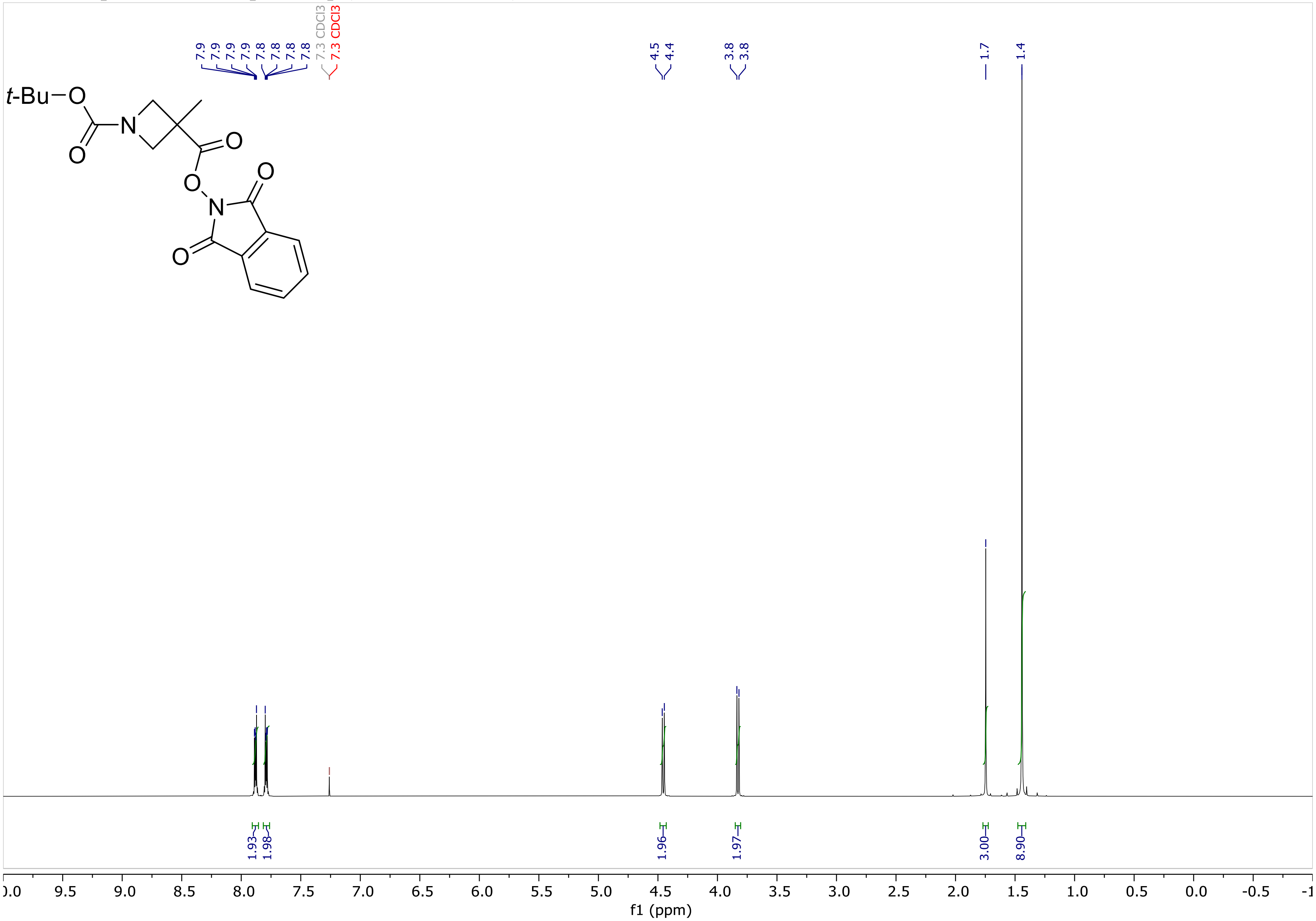

$^{13}C\{^{1}H\}$ NMR spectrum of compound 1p (151 MHz, $CDCl_3$)

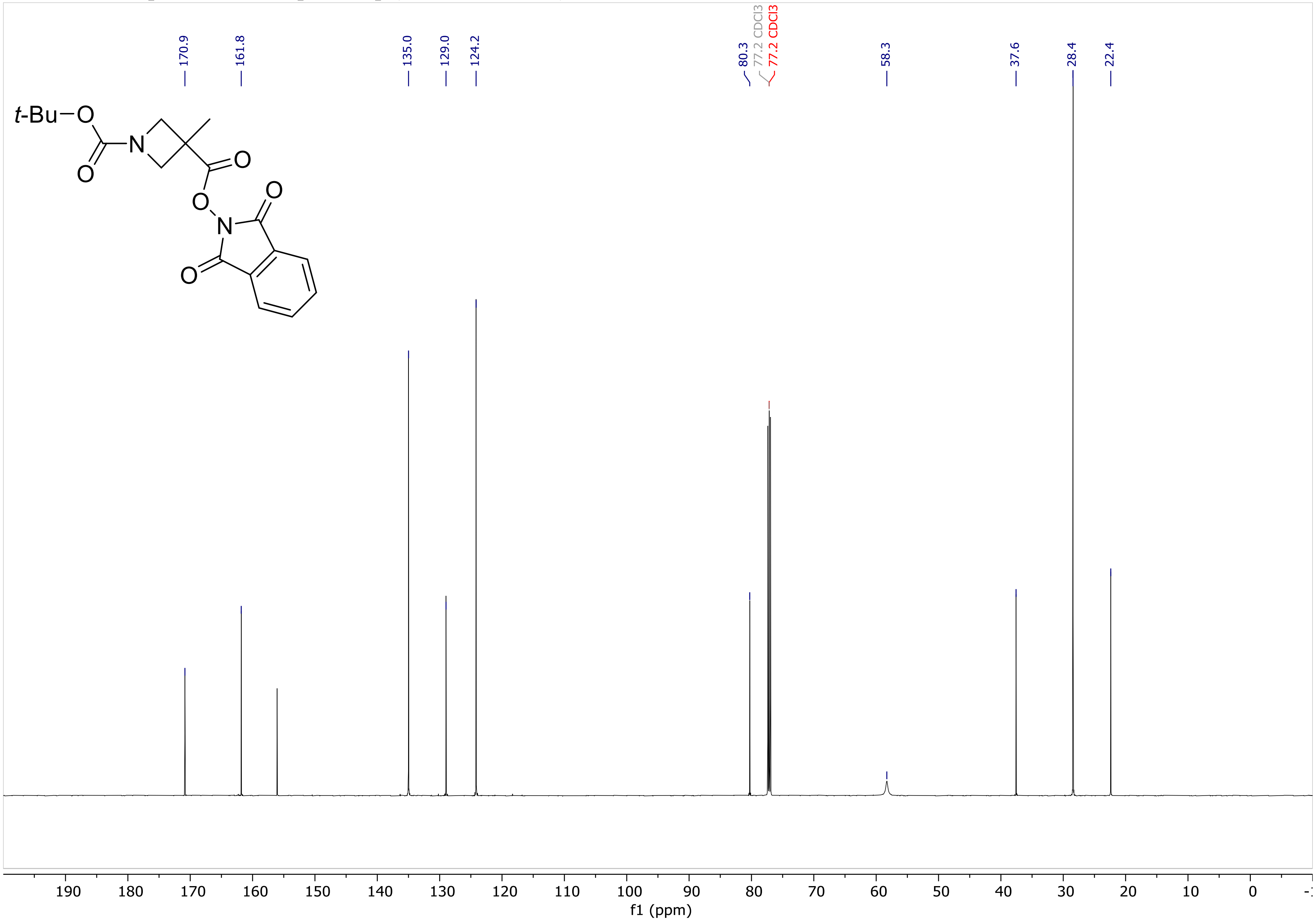

^{1}H NMR spectrum of compound 1q (500 MHz, $CDCl_3$)

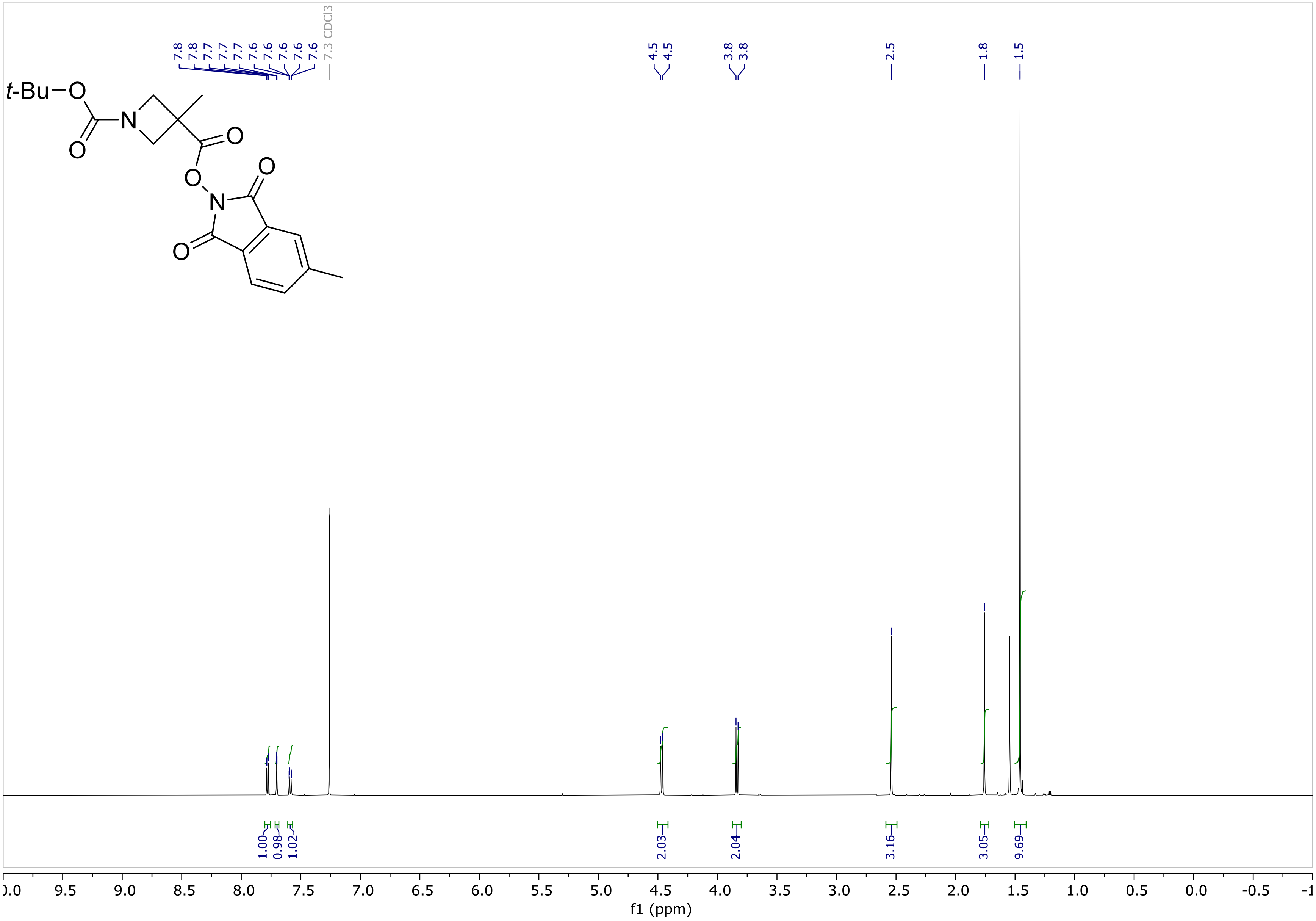

$^{13}C\{^1H\}$ NMR spectrum of compound 1q (126 MHz, $CDCl_3$)

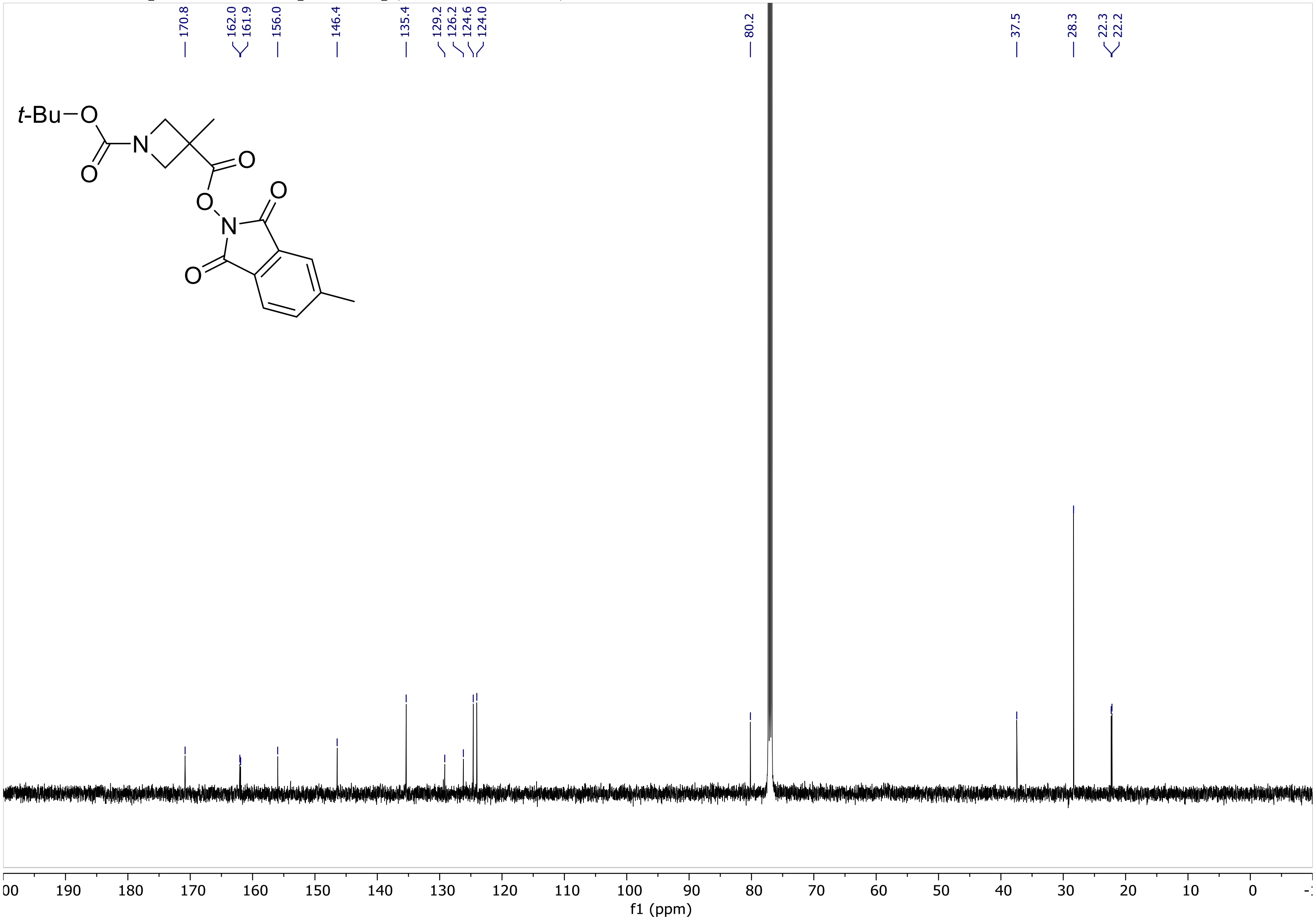

^{1}H NMR spectrum of compound 1r (500 MHz, $CDCl_3$)

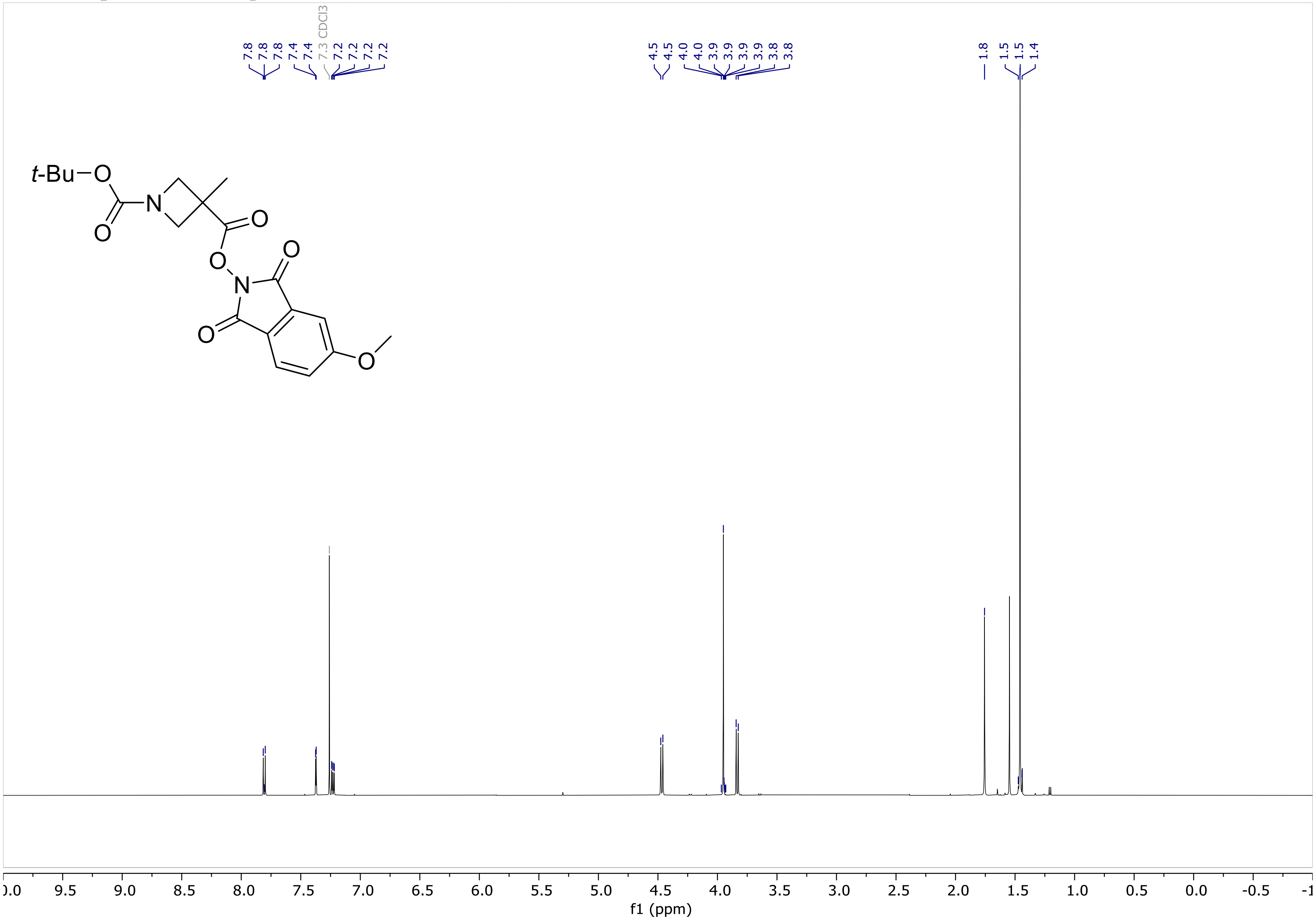

$^{13}C\{^{1}H\}$ NMR spectrum of compound 1r (126 MHz, $CDCl_3$)

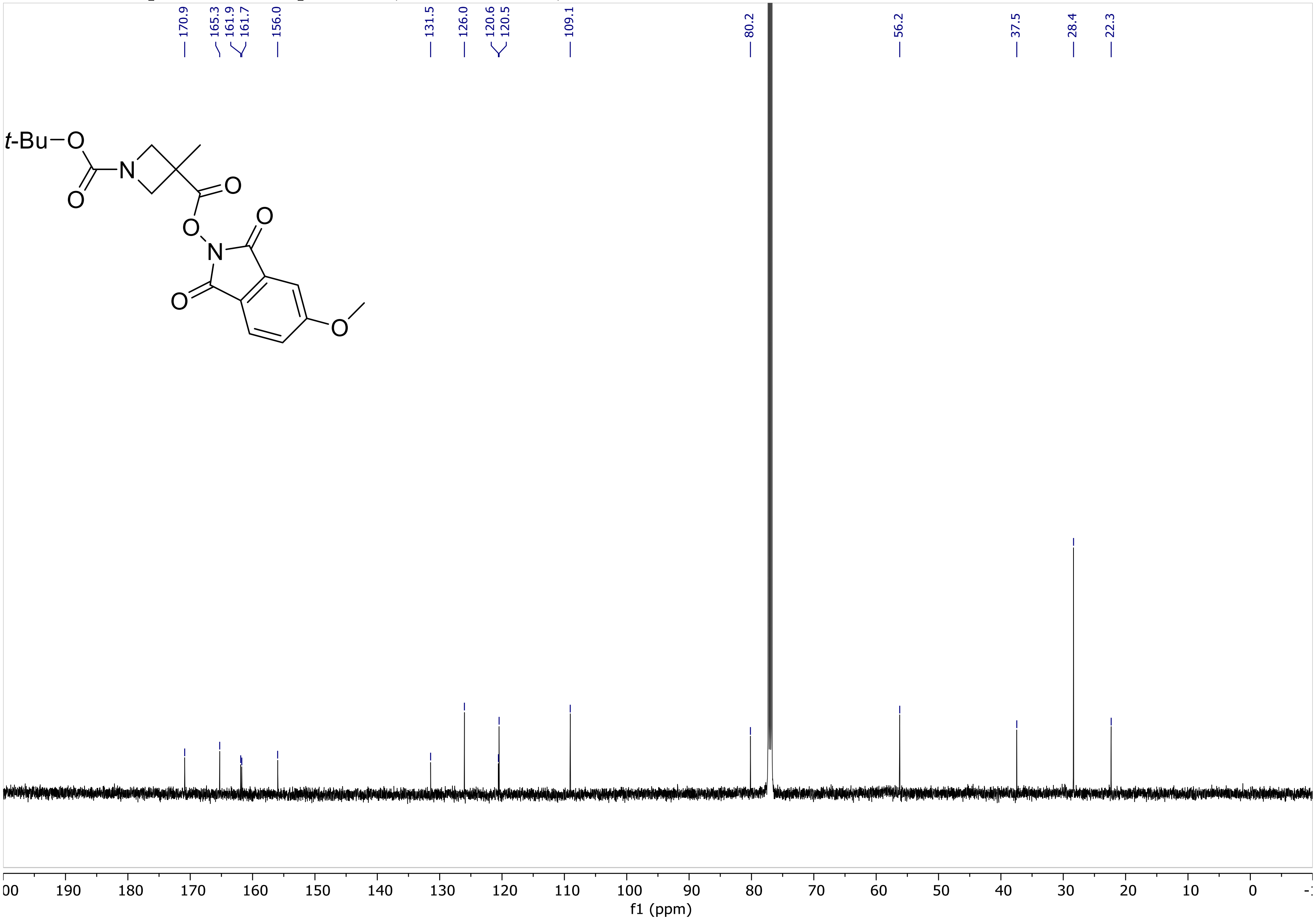

^{1}H NMR spectrum of compound 1s (500 MHz, CDCl$_3$)

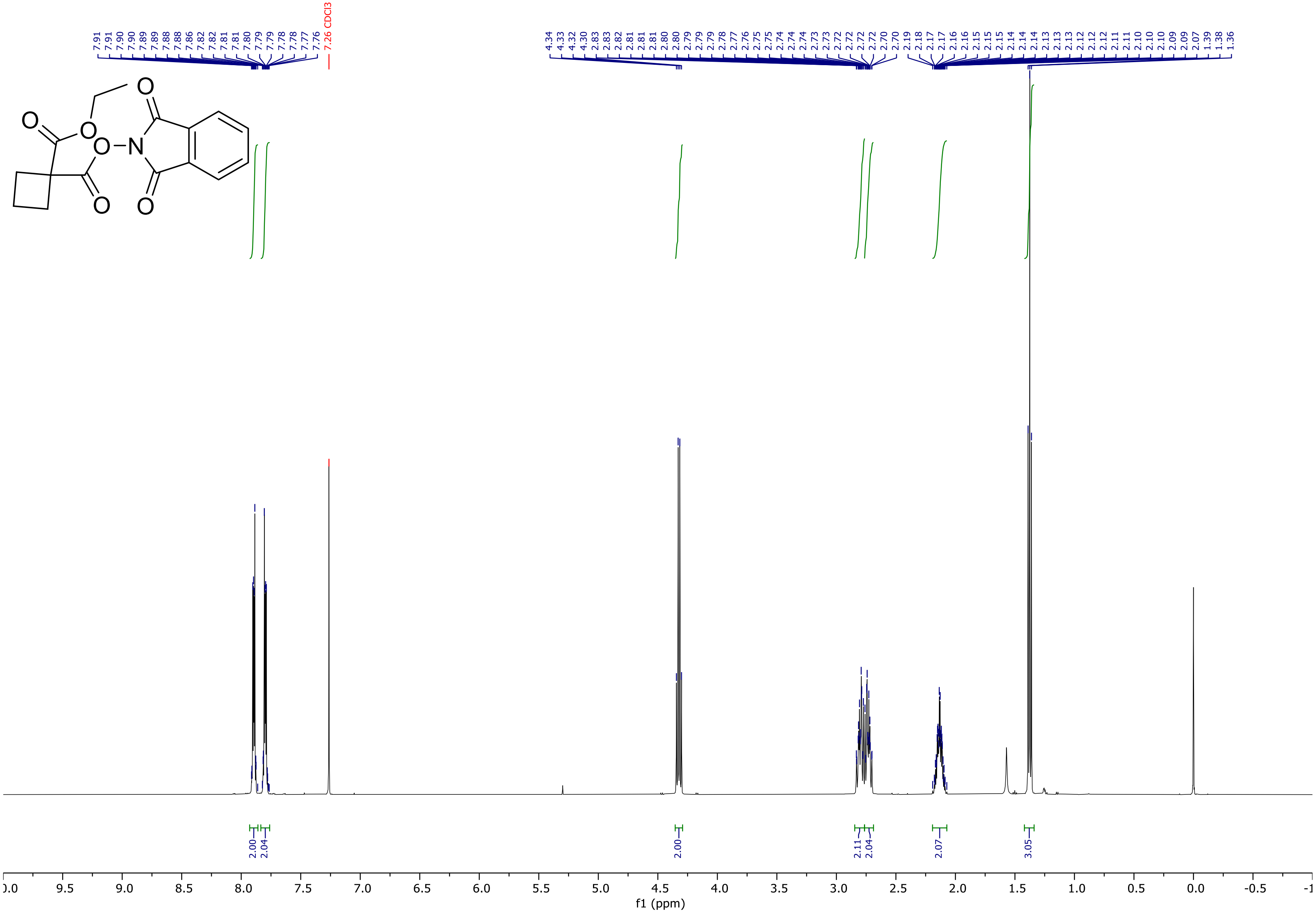

$^{13}C\{^1H\}$ NMR spectrum of compound 1s (126 MHz, $CDCl_3$)

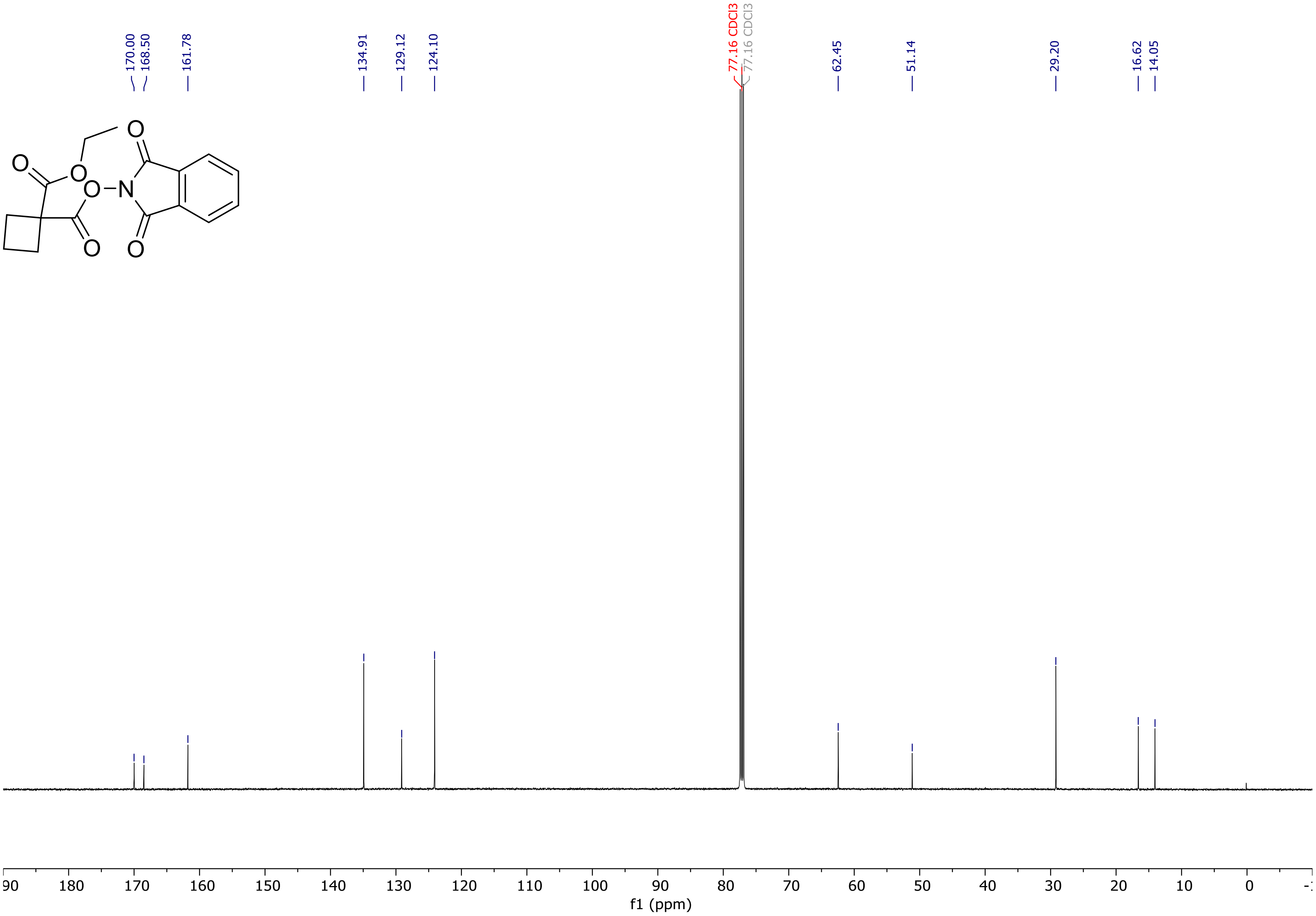

^{1}H NMR spectrum of compound 1t (500 MHz, CDCl$_3$)

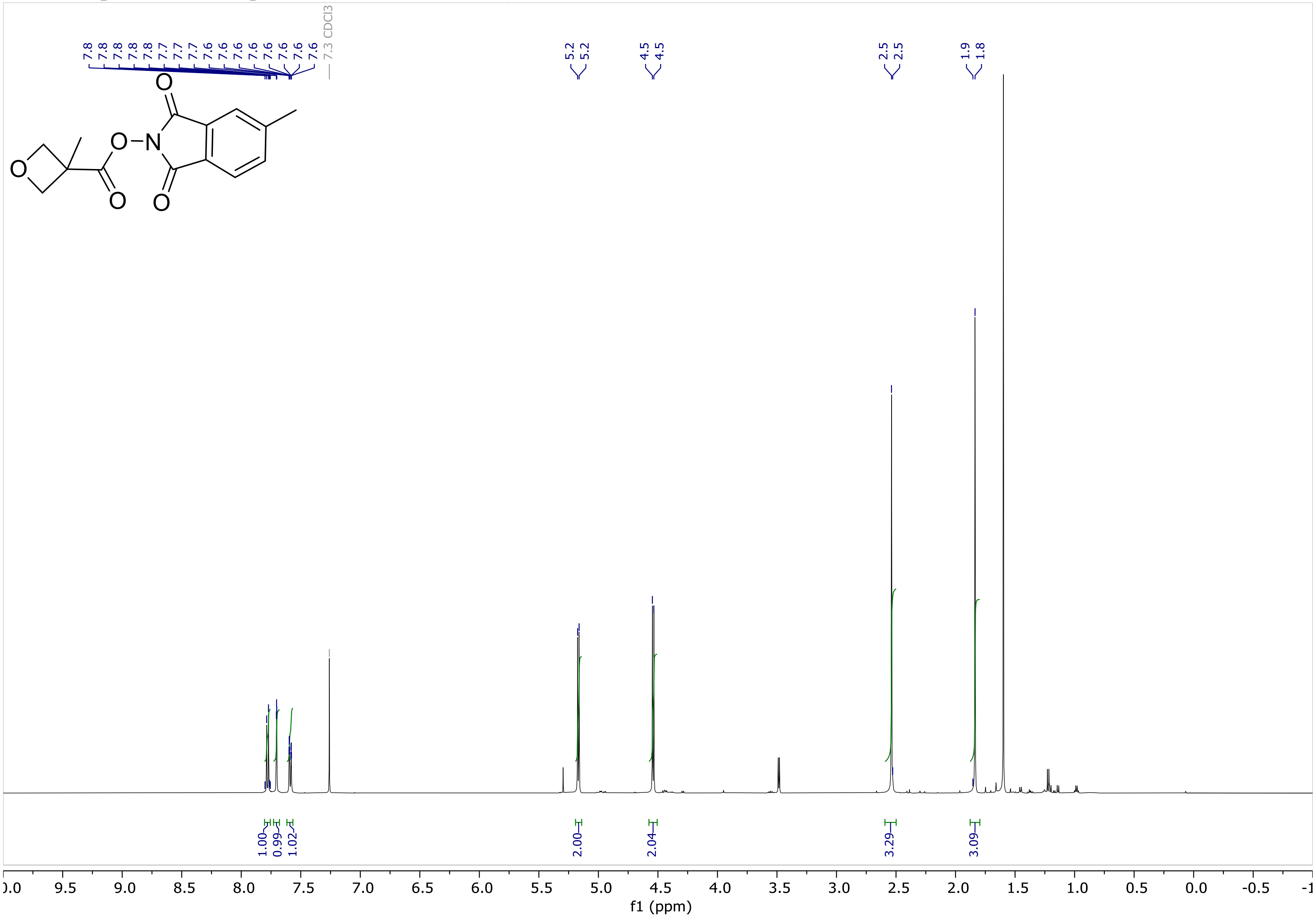

$^{13}C\{^{1}H\}$ NMR spectrum of compound 1t (126 MHz, $CDCl_3$)

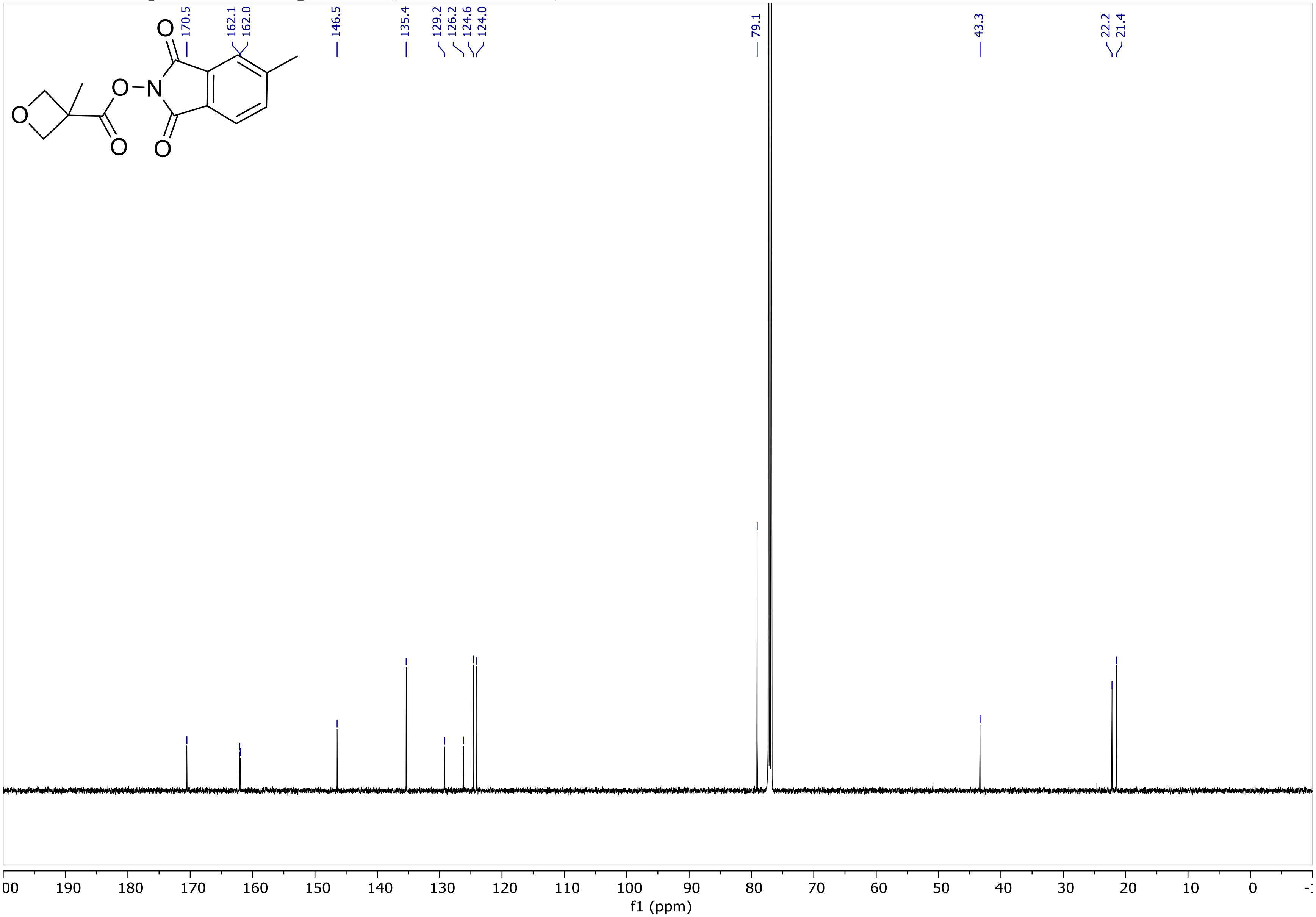

^{1}H NMR spectrum of compound 1u (500 MHz, $CDCl_3$)

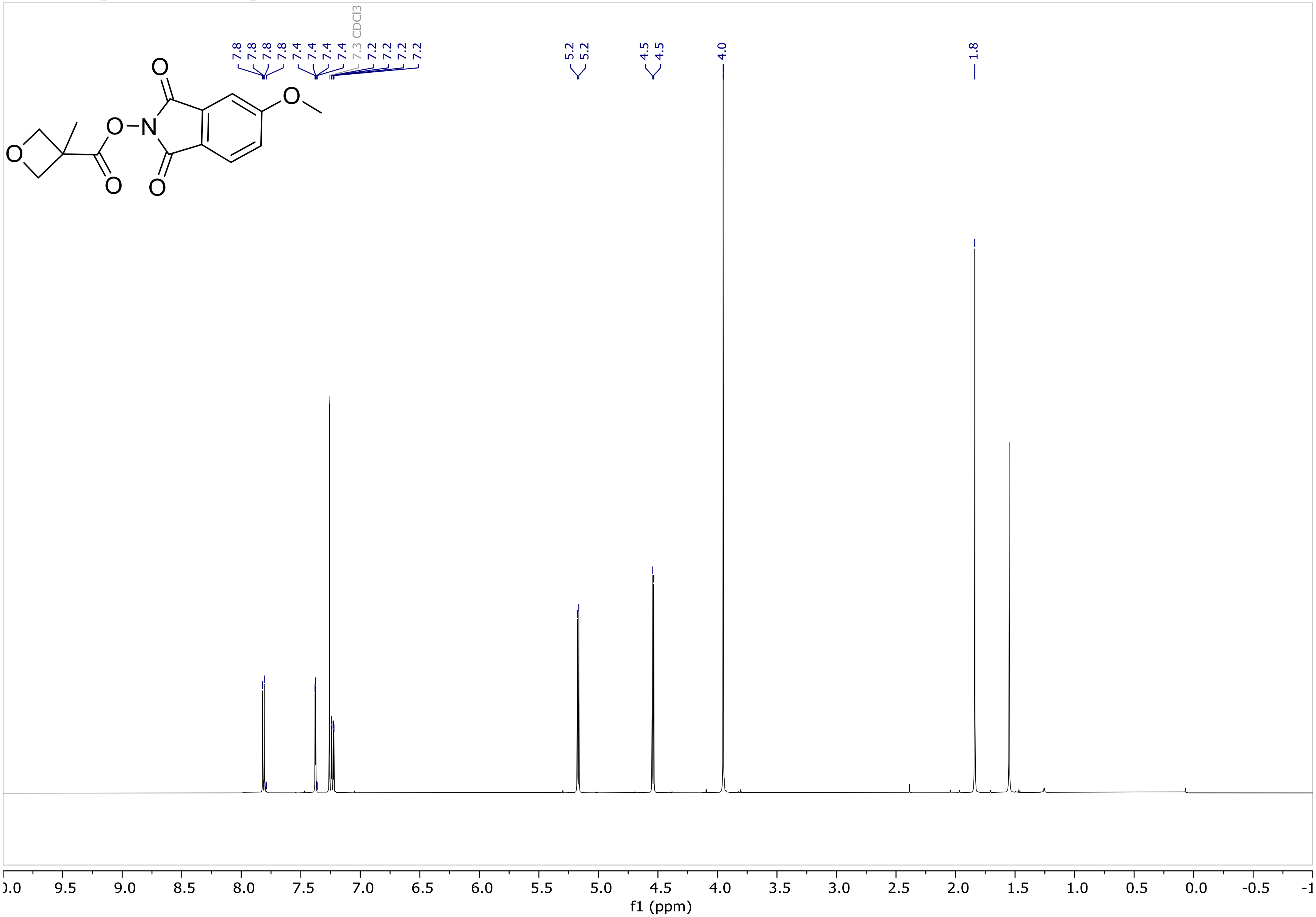

$^{13}C\{^{1}H\}$ NMR spectrum of compound 1u (126 MHz, $CDCl_3$)

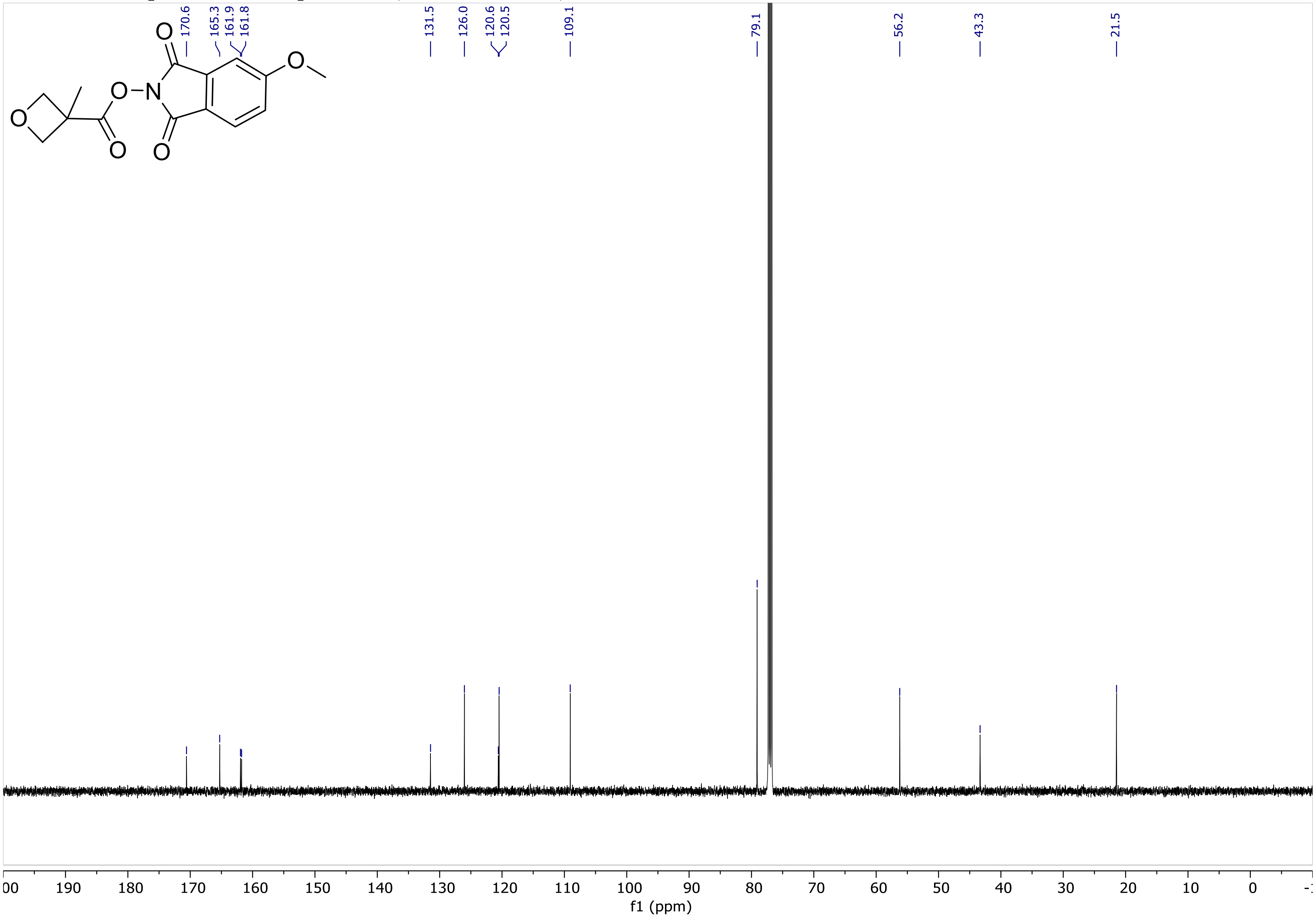

^{1}H NMR spectrum of compound 3a (500 MHz, $CDCl_3$)

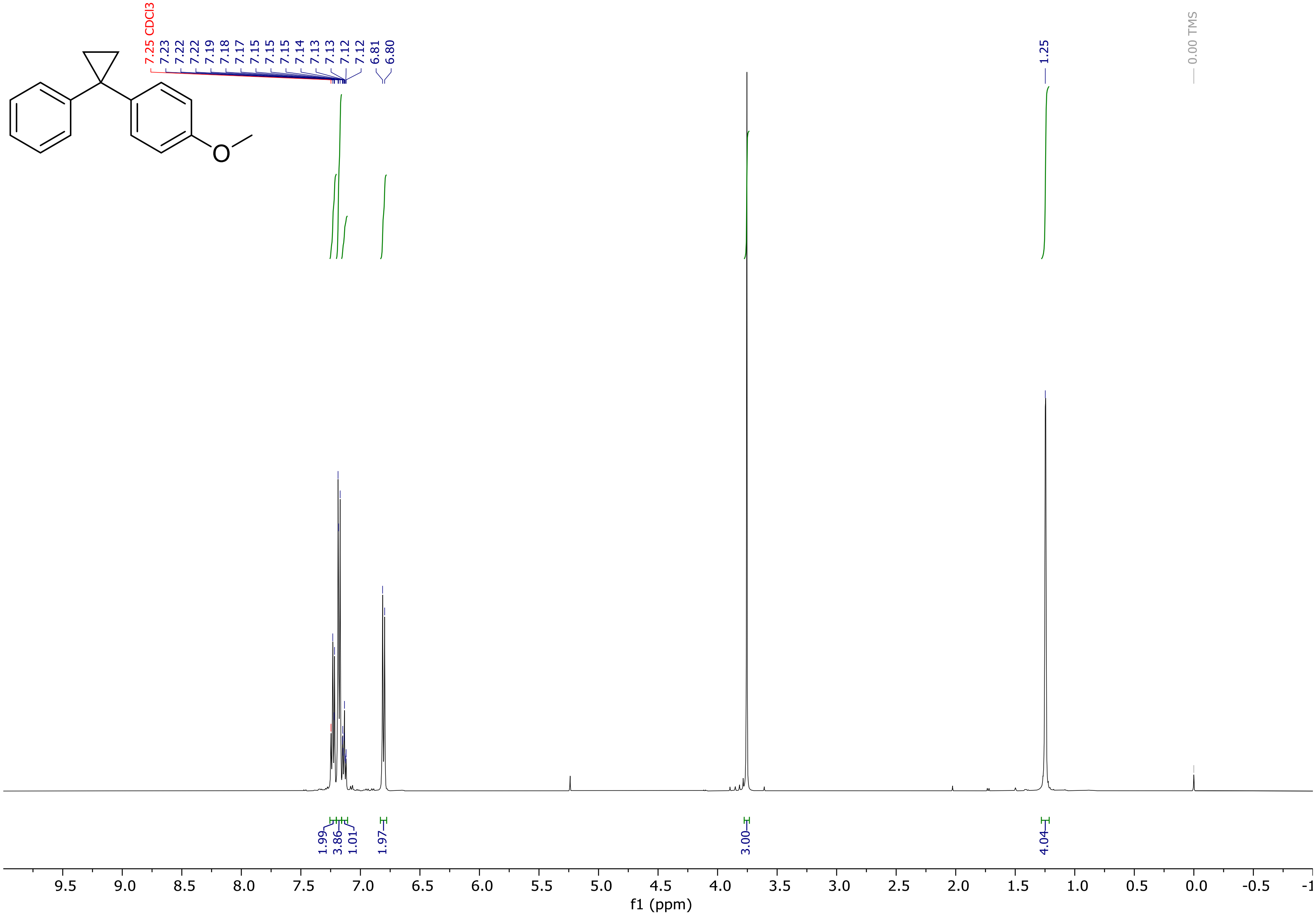

$^{13}C\{^1H\}$ NMR spectrum of compound 3a (126 MHz, $CDCl_3$)

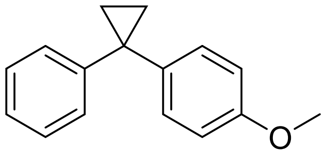

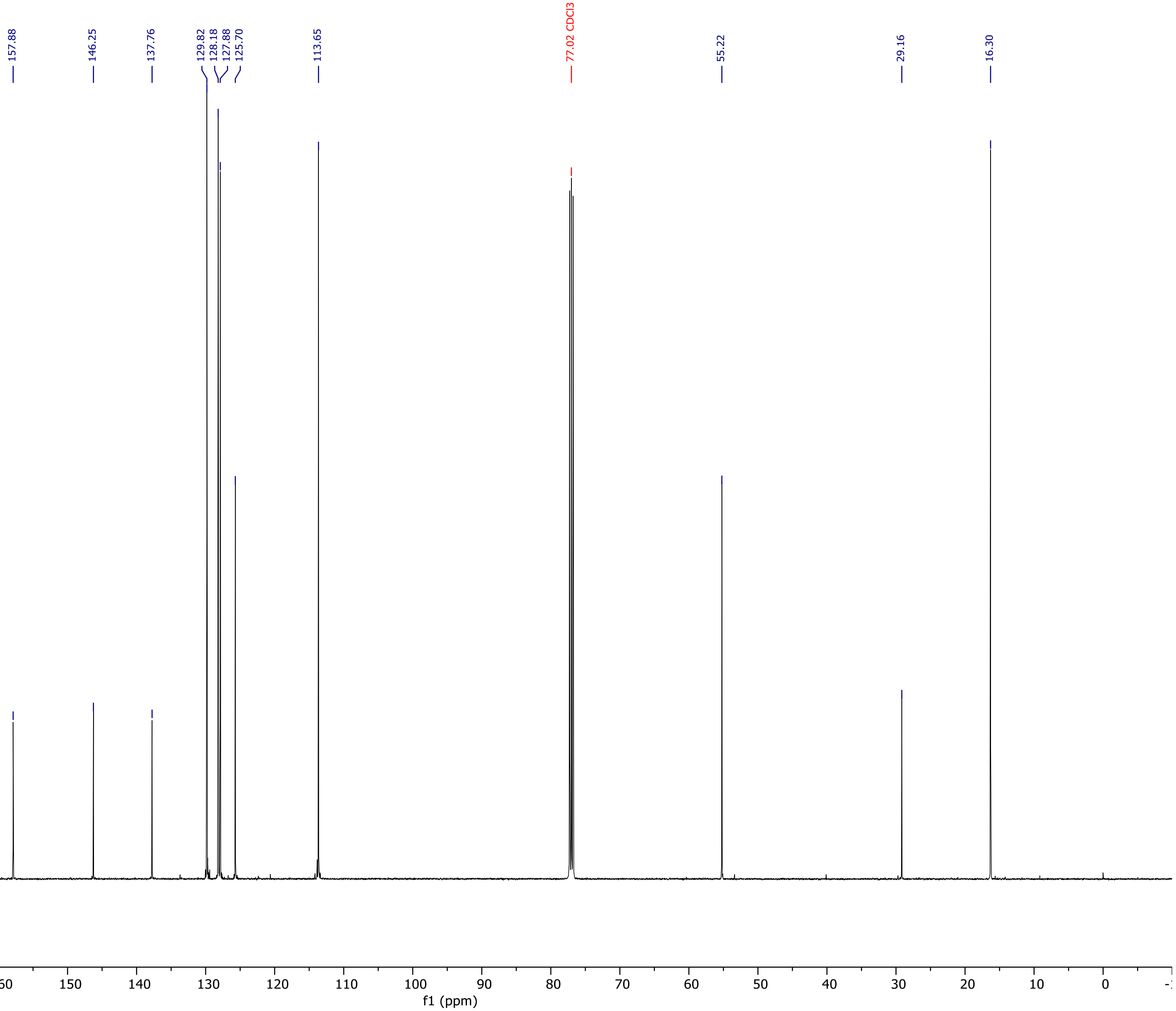

^{1}H NMR spectrum of compound 3b (500 MHz, $CDCl_3$)

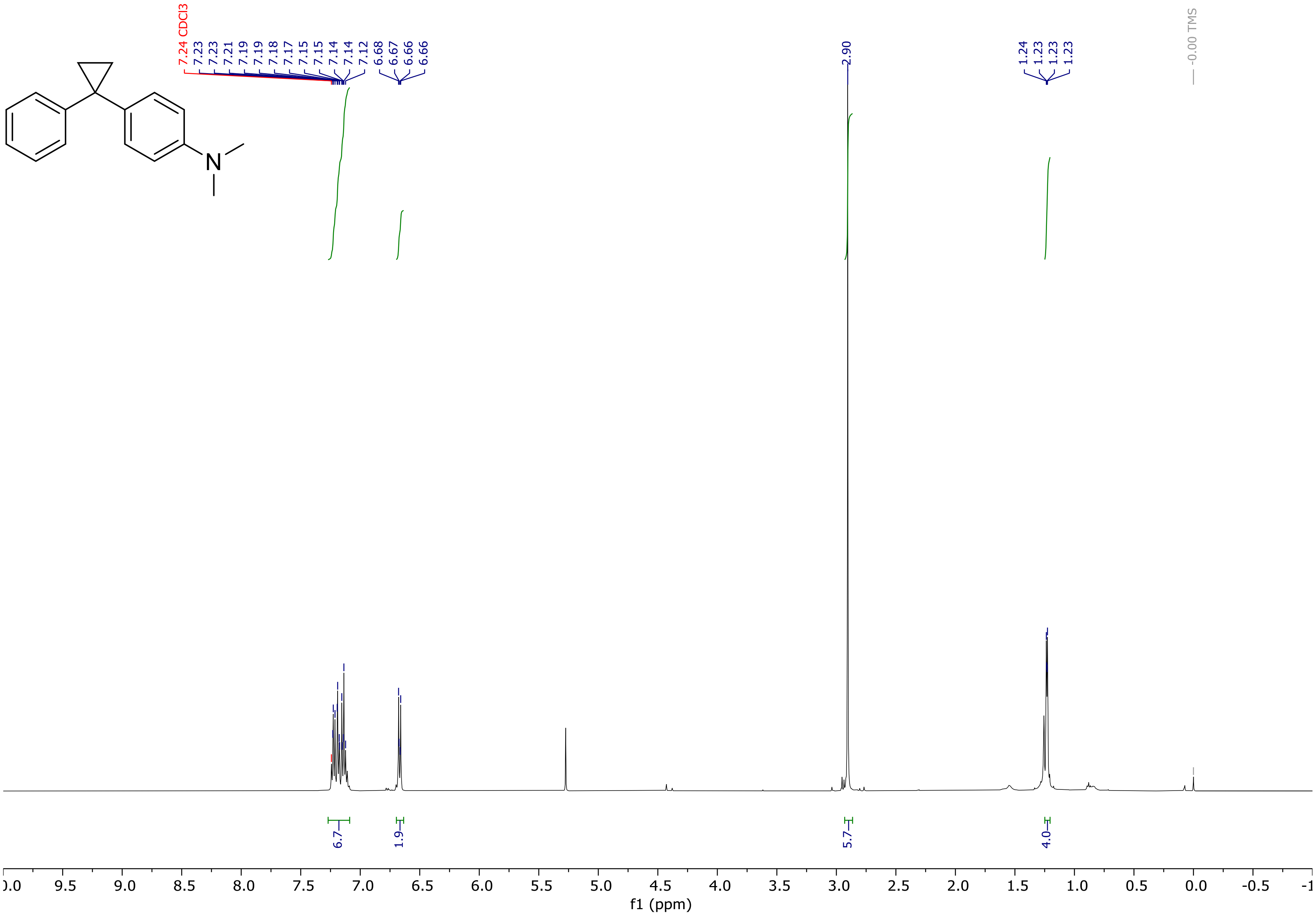

$^{13}C\{^{1}H\}$ NMR spectrum of compound 3b (126 MHz, $CDCl_3$)

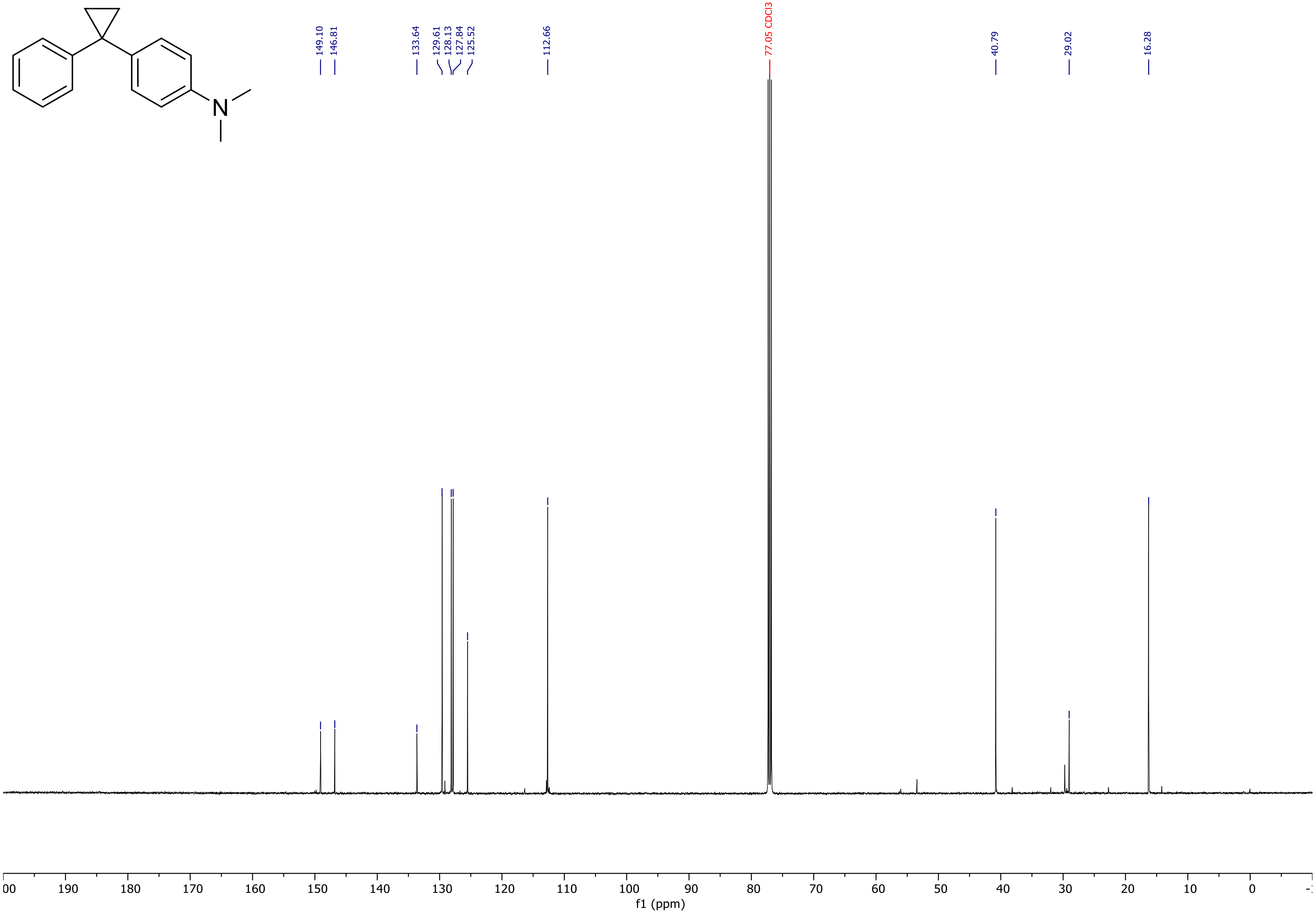

^{1}H NMR spectrum of compound 3c (400 MHz, CDCl₃)

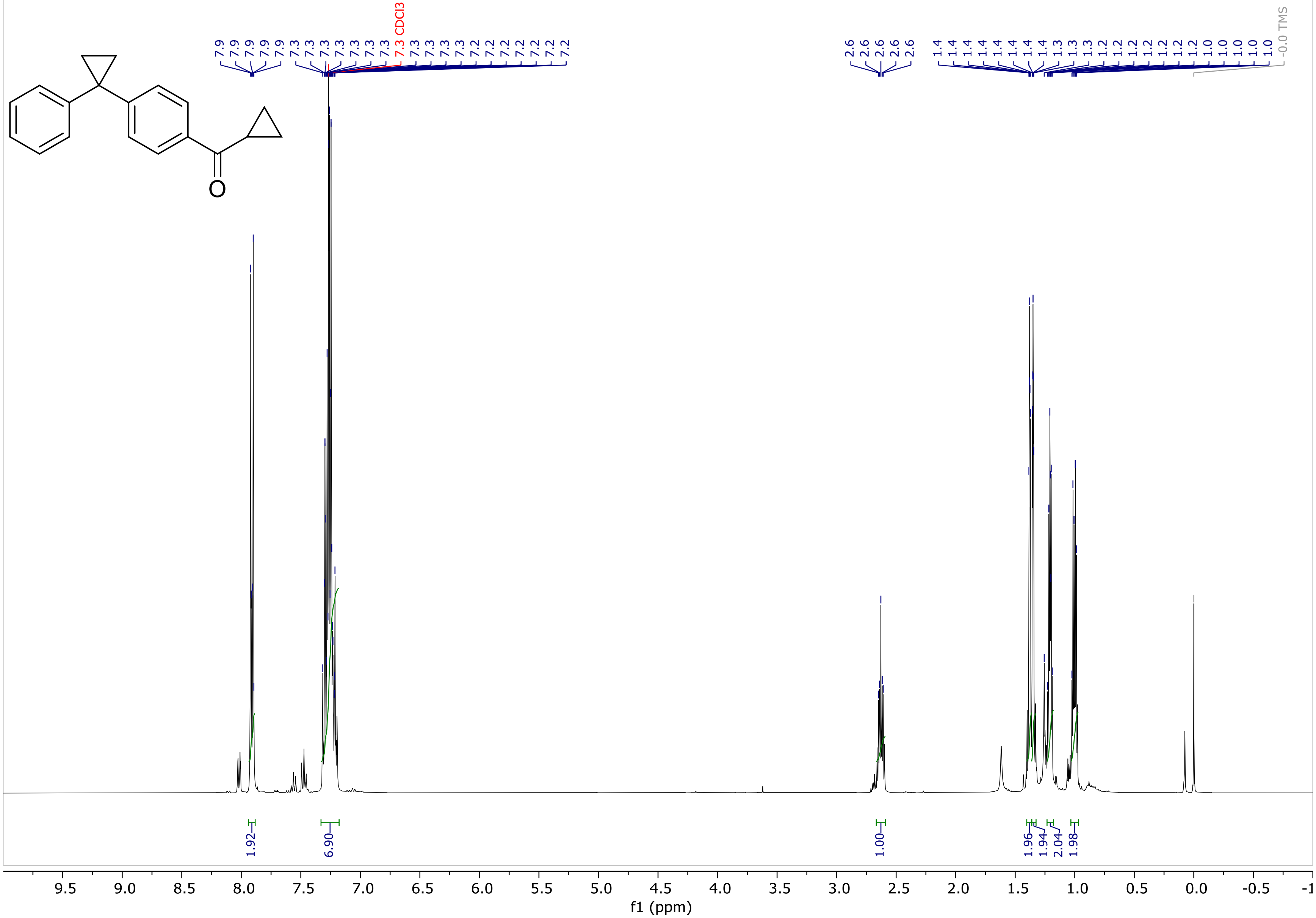

$^{13}C\{^1H\}$ NMR spectrum of compound 3c (126 MHz, CDCl$_3$)

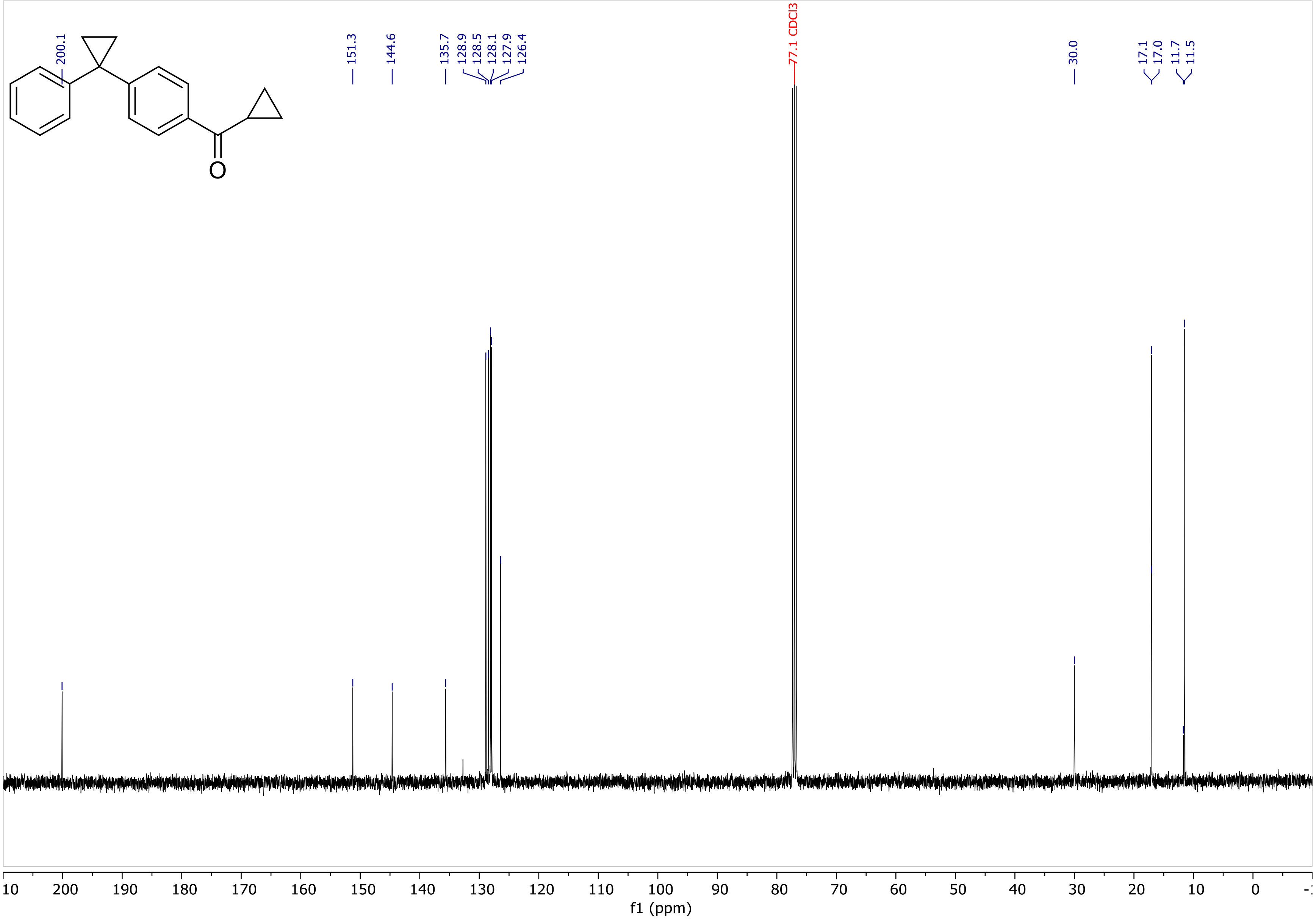

^{1}H NMR spectrum of compound 3d (500 MHz, $CDCl_3$)

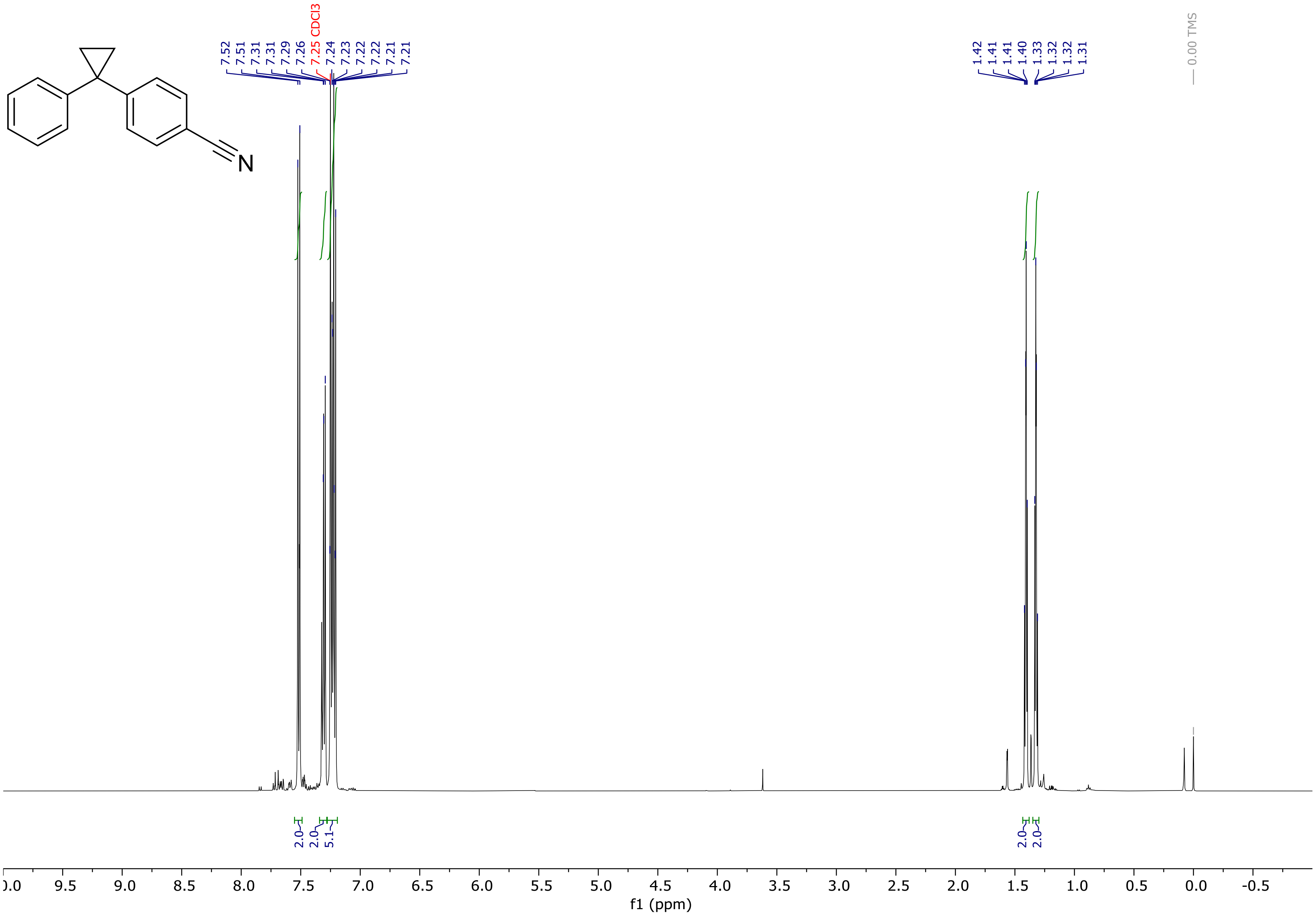

$^{13}C\{^1H\}$ NMR spectrum of compound 3d (126 MHz, $CDCl_3$)

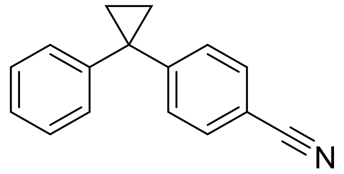

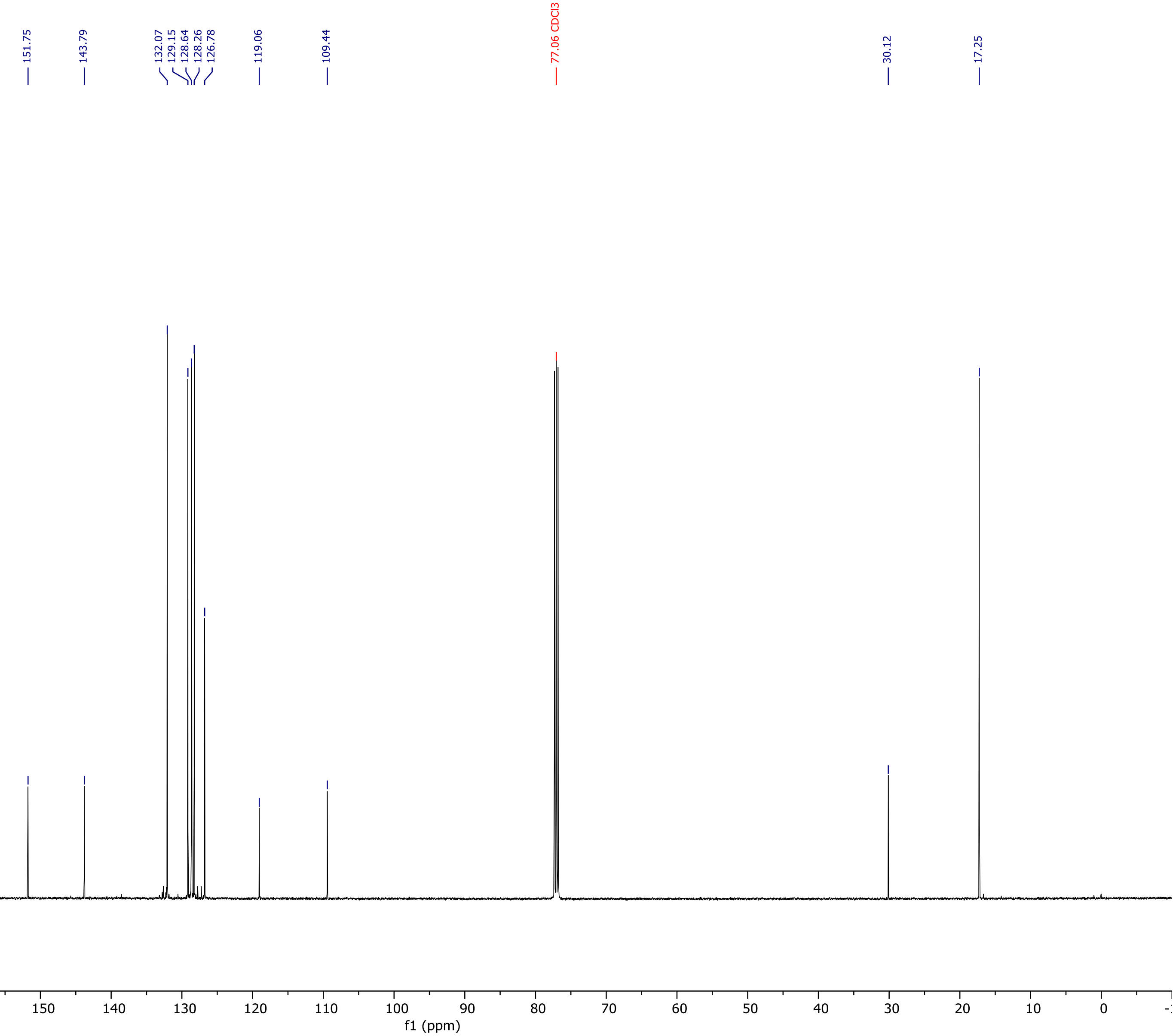

^{1}H NMR spectrum of compound 3e (500 MHz, CDCl$_3$)

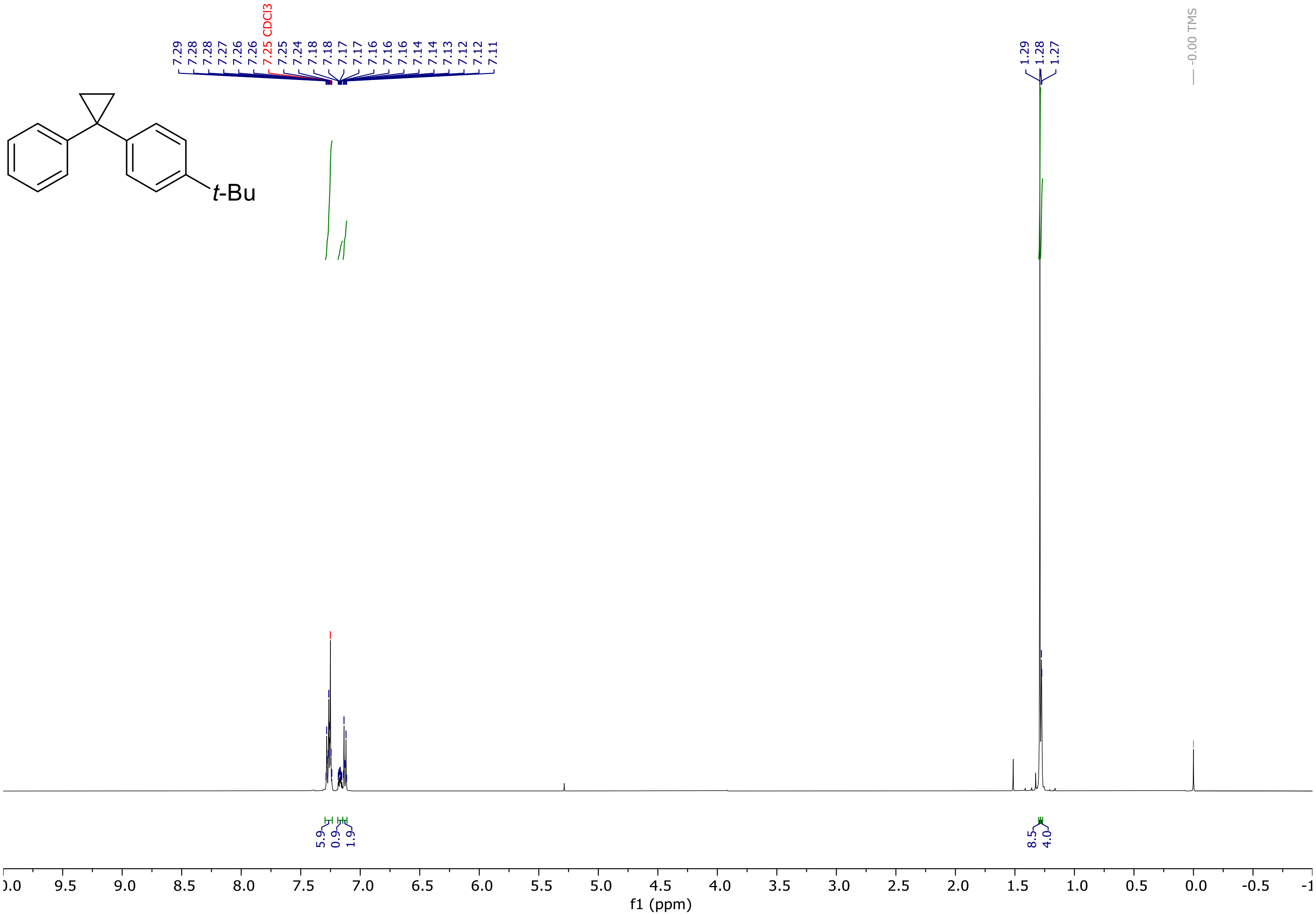

$^{13}C\{^1H\}$ NMR spectrum of compound 3e (126 MHz, $CDCl_3$)

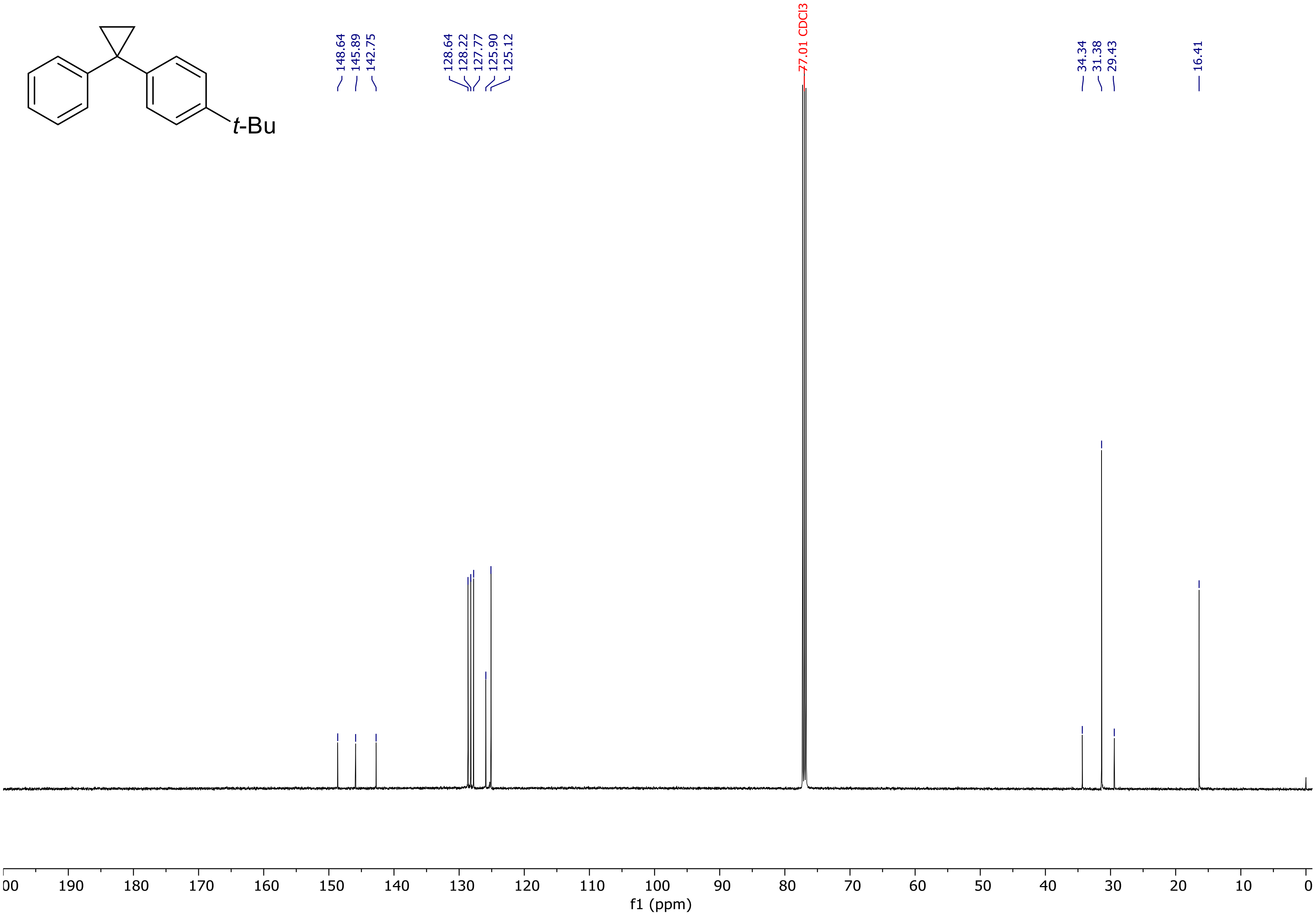

^{1}H NMR spectrum of compound 3f (500 MHz, $CDCl_3$)

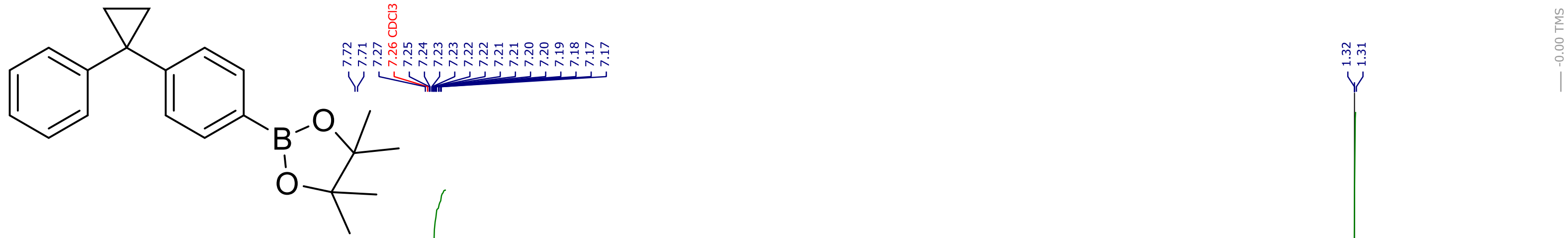

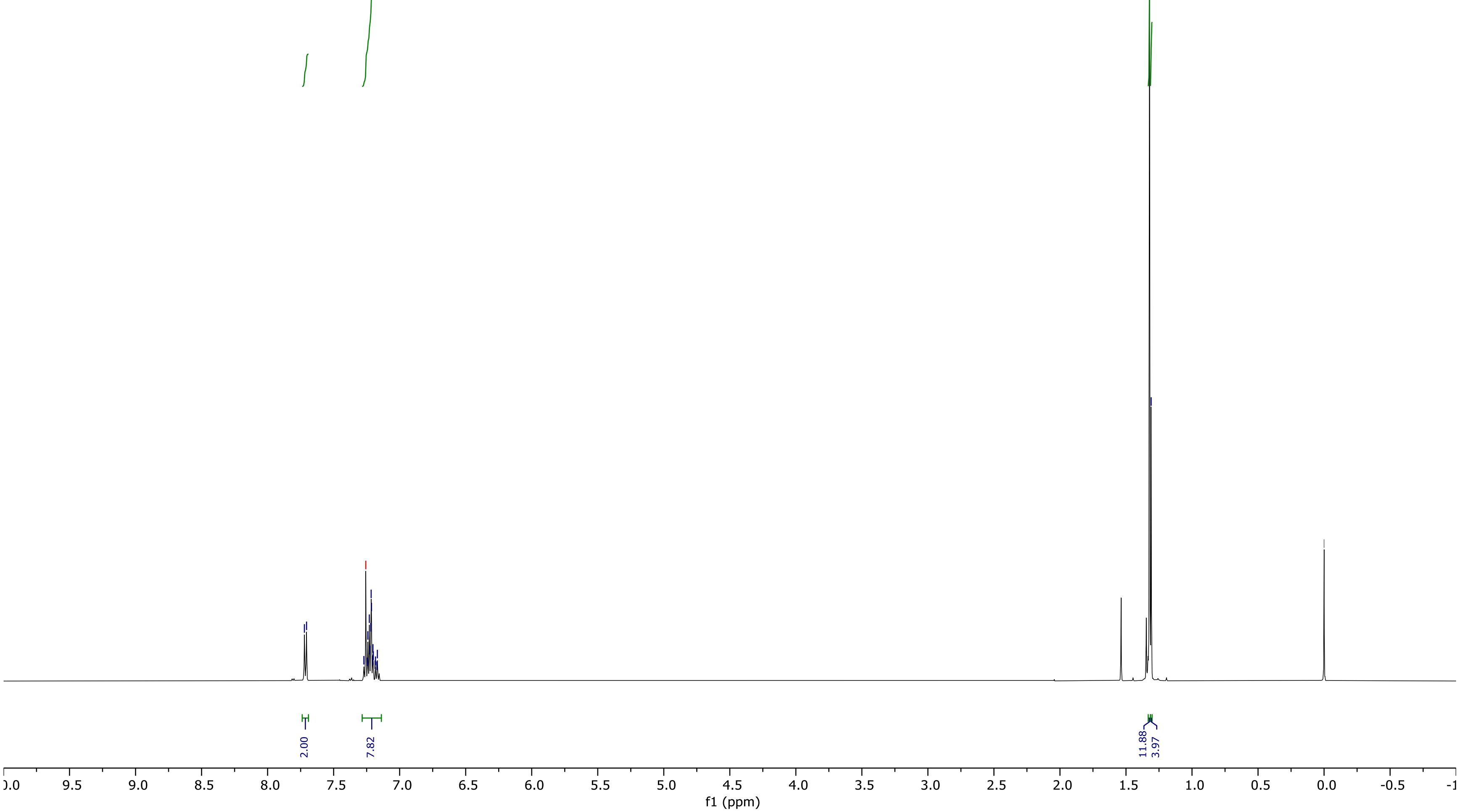

$^{13}C\{^{1}H\}$ NMR spectrum of compound 3f (126 MHz, $CDCl_3$)

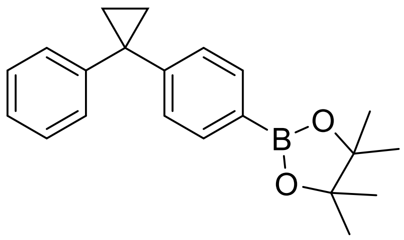

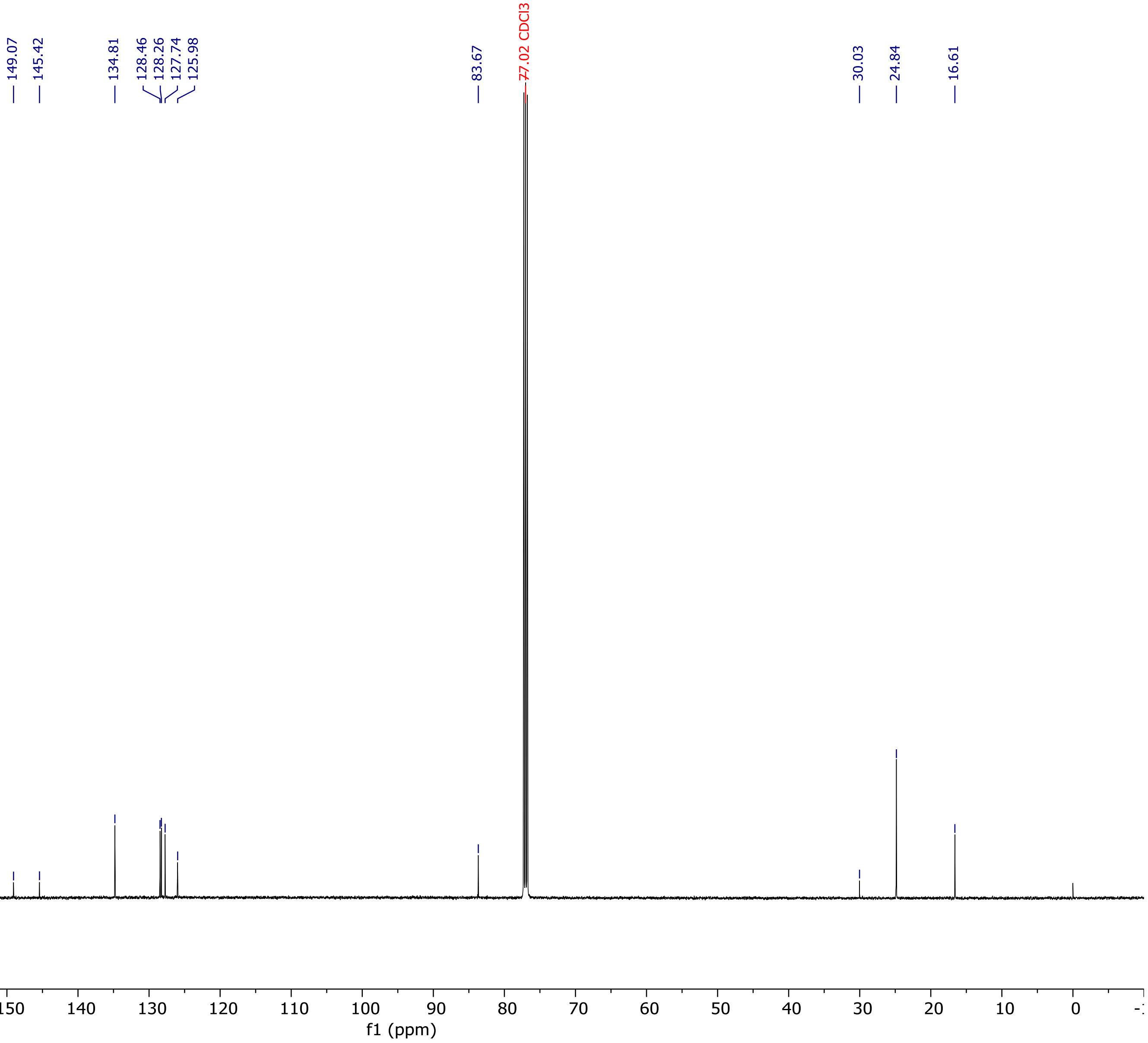

^{1}H NMR spectrum of compound 3g (400 MHz, $CDCl_3$)

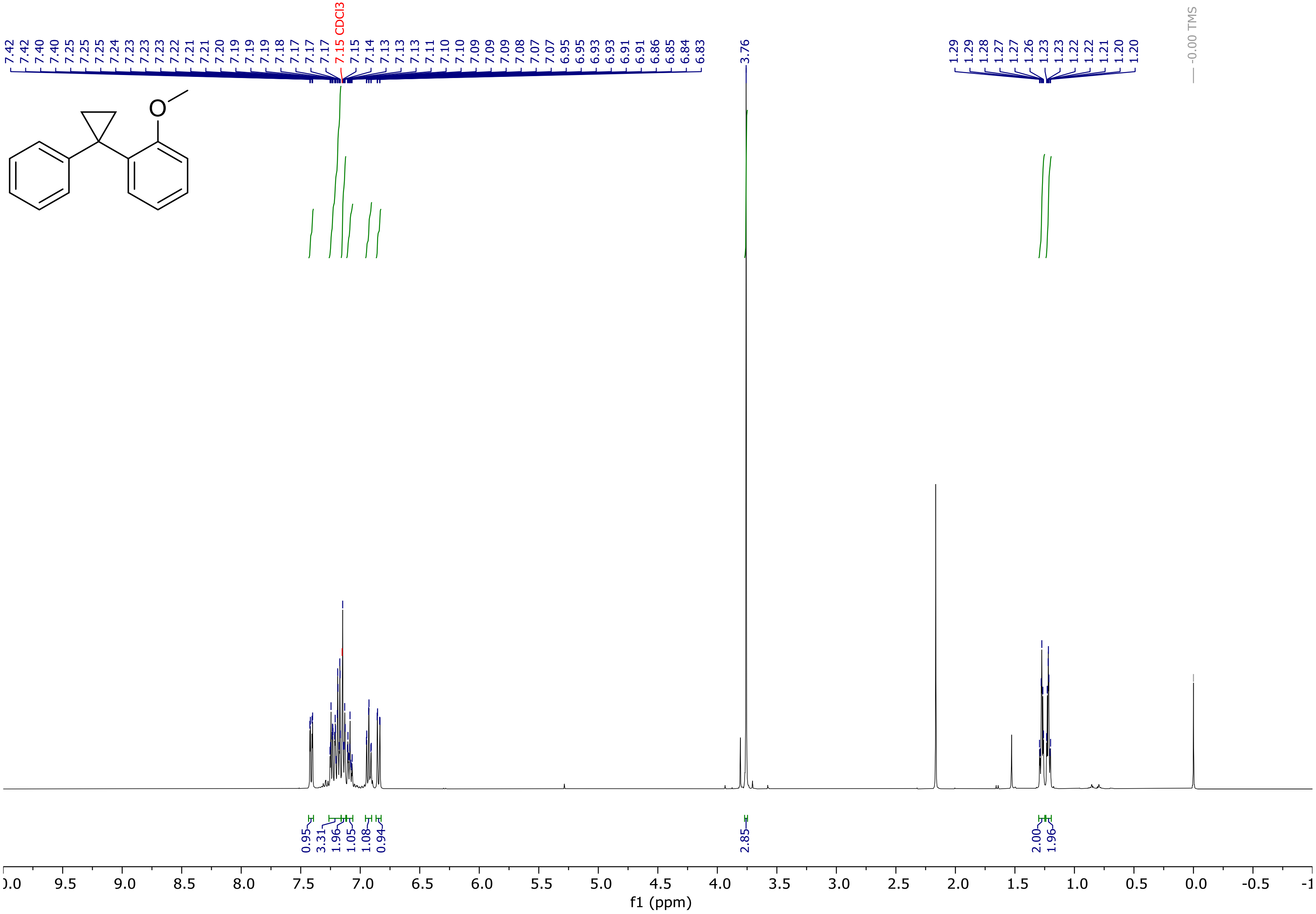

$^{13}C\{^1H\}$ NMR spectrum of compound 3g (101 MHz, $CDCl_3$)

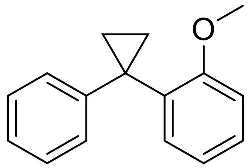

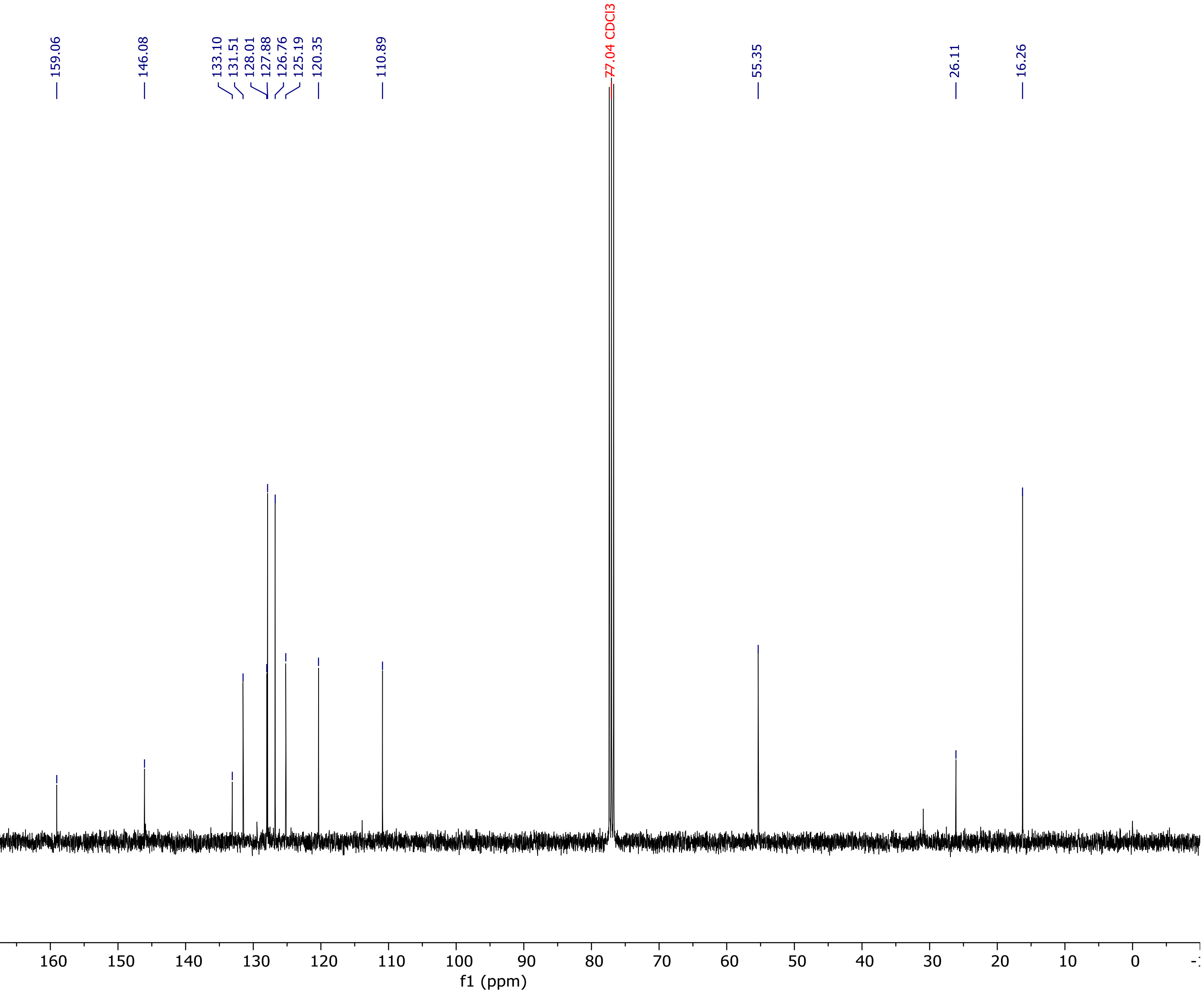

^{1}H NMR spectrum of compound 3h (500 MHz, $CDCl_3$)

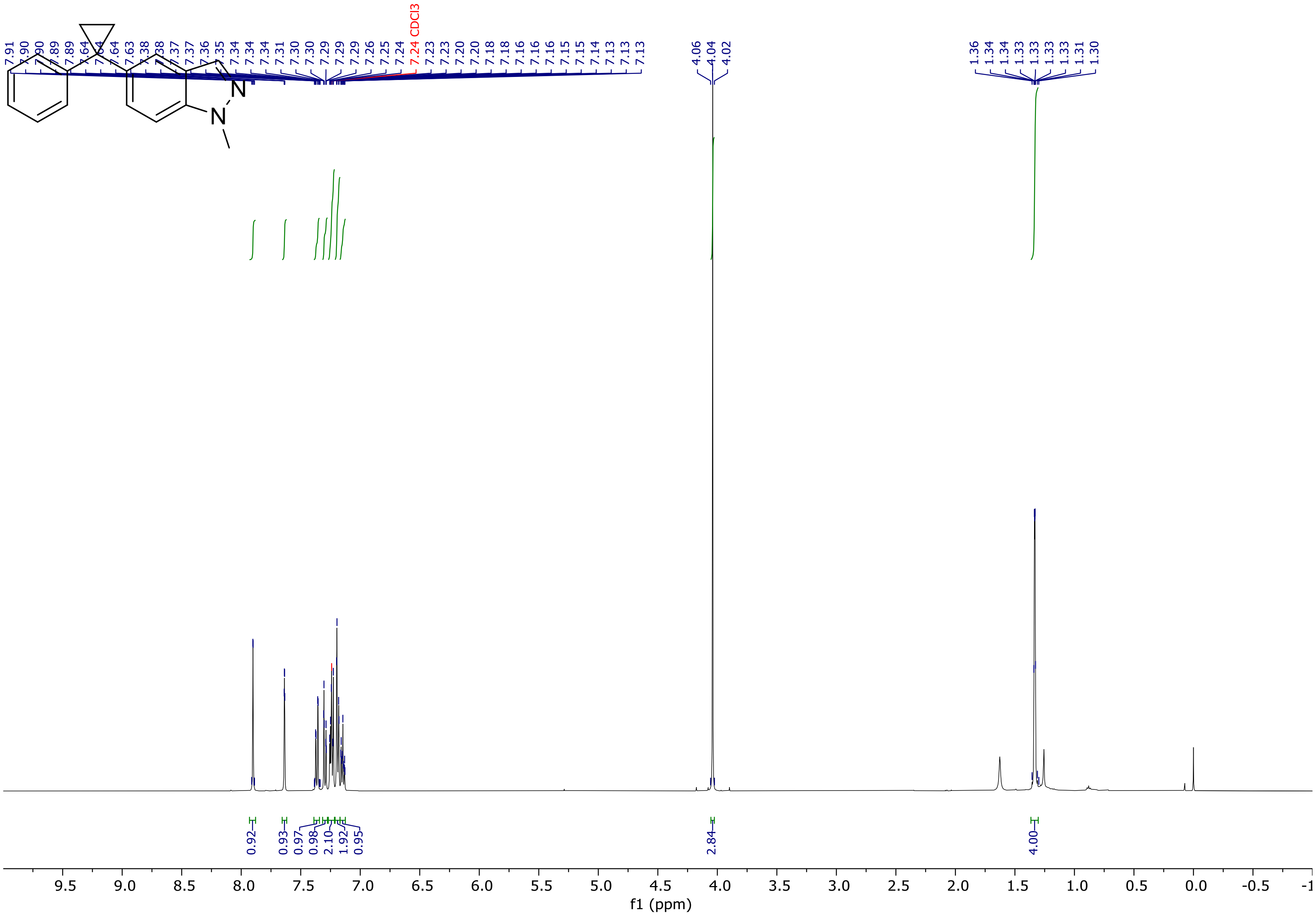

$^{13}C\{^{1}H\}$ NMR spectrum of compound 3h (126 MHz, $CDCl_3$)

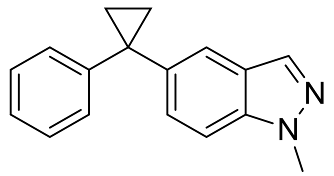

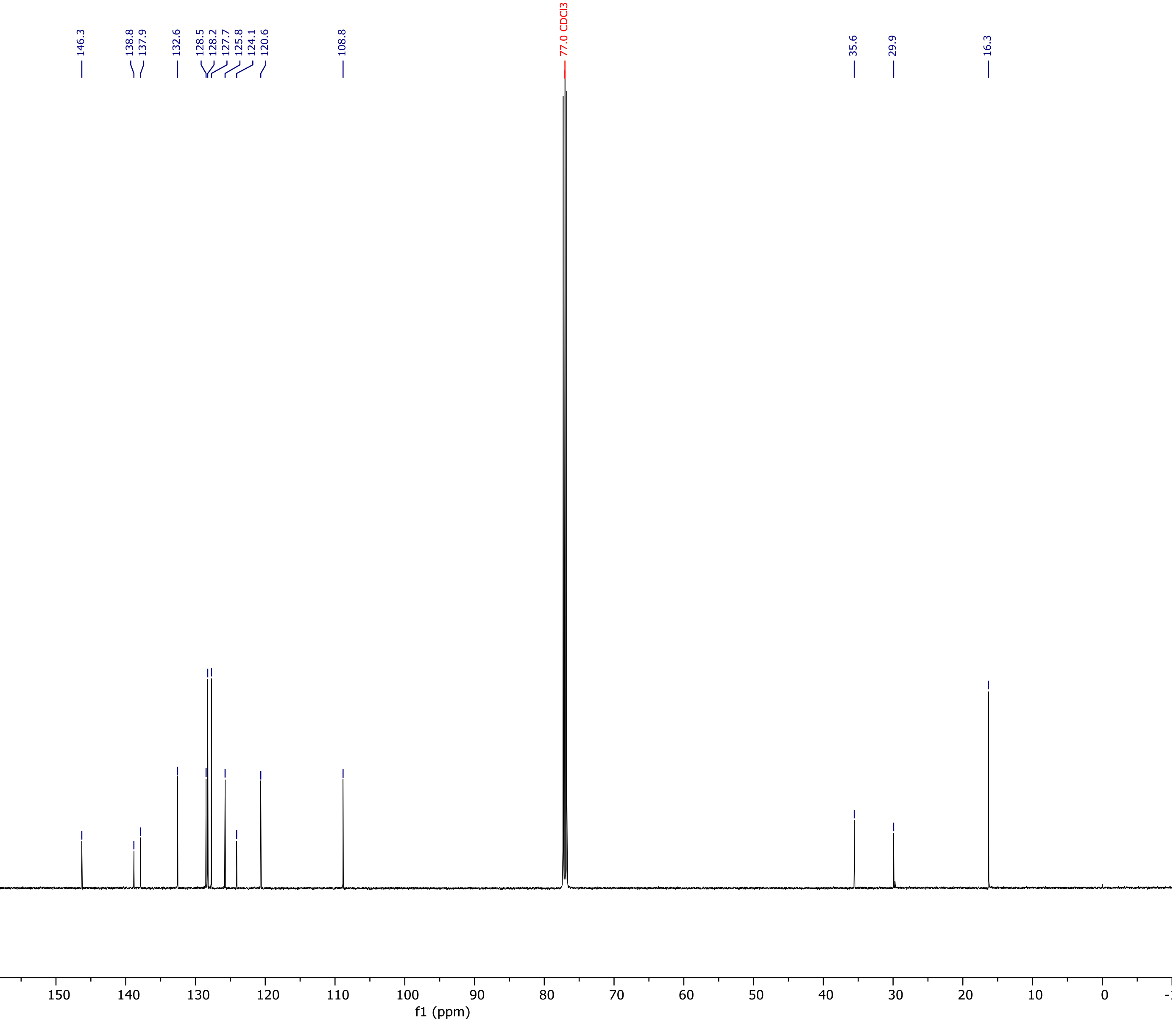

^{1}H NMR spectrum of compound 3i (400 MHz, CDCl$_3$)

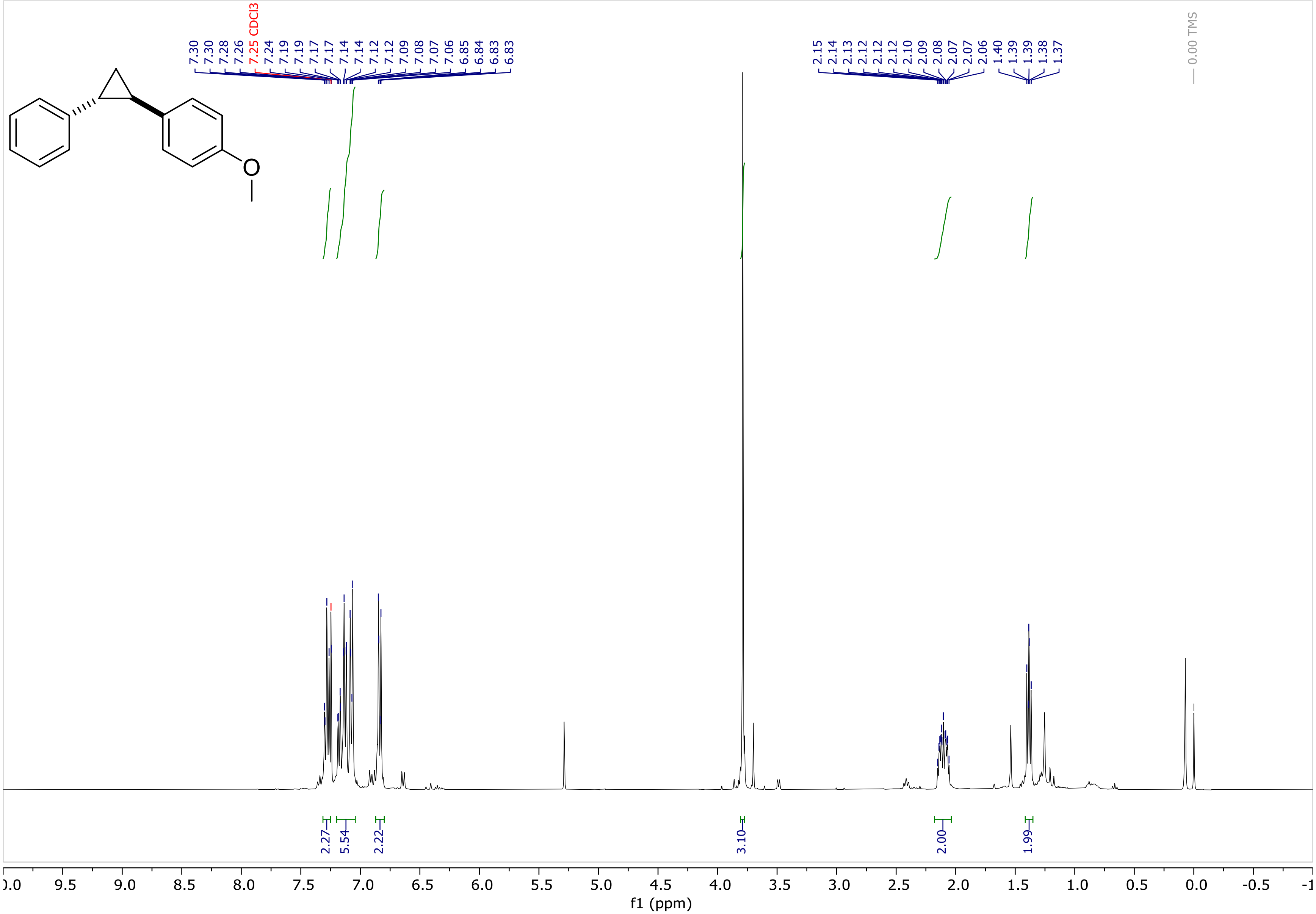

$^{13}C\{^1H\}$ NMR spectrum of compound 3i (101 MHz, $CDCl_3$)

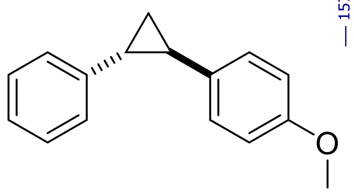

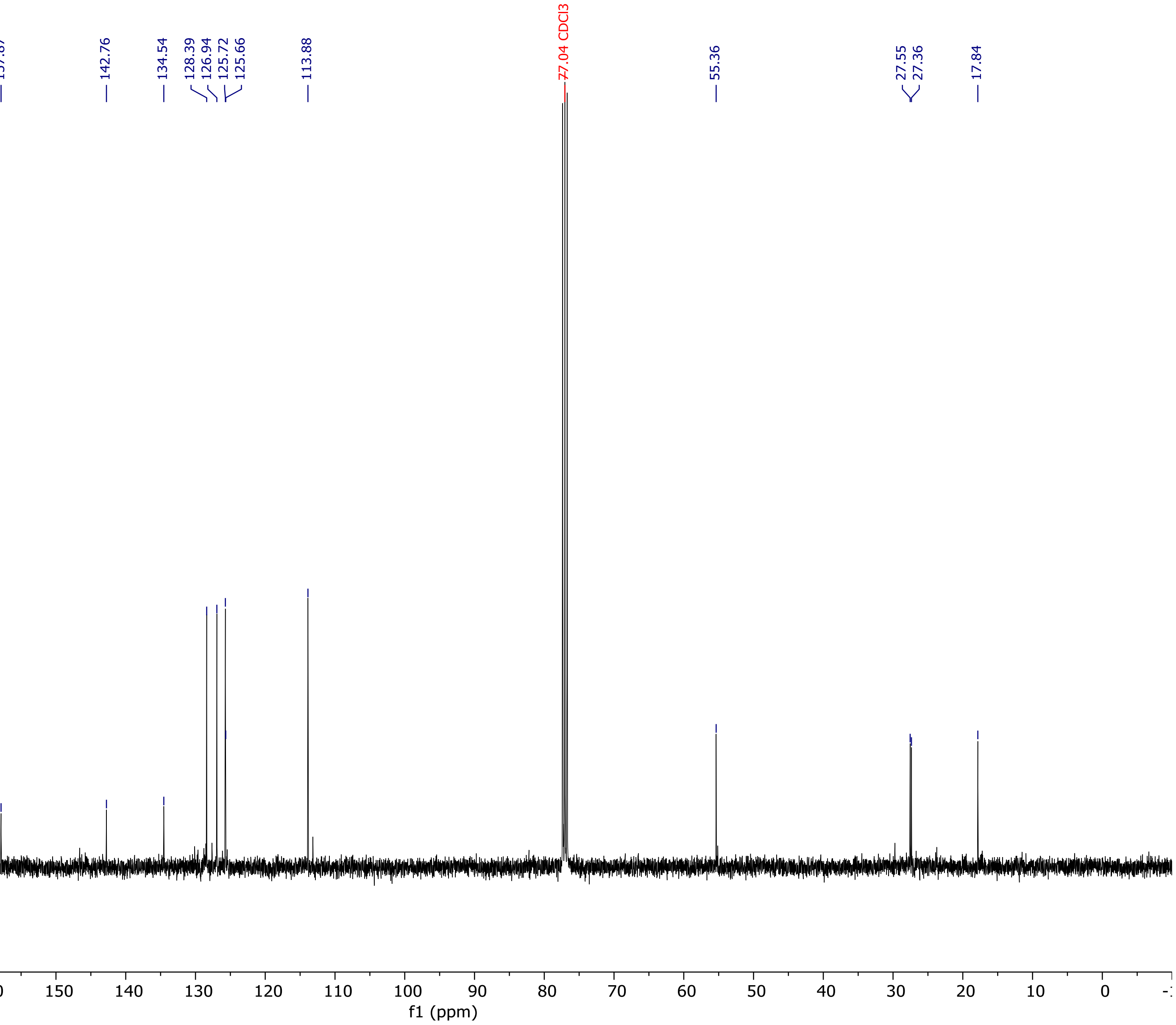

¹H NMR spectrum of compound 3j (500 MHz, $CDCl_3$)

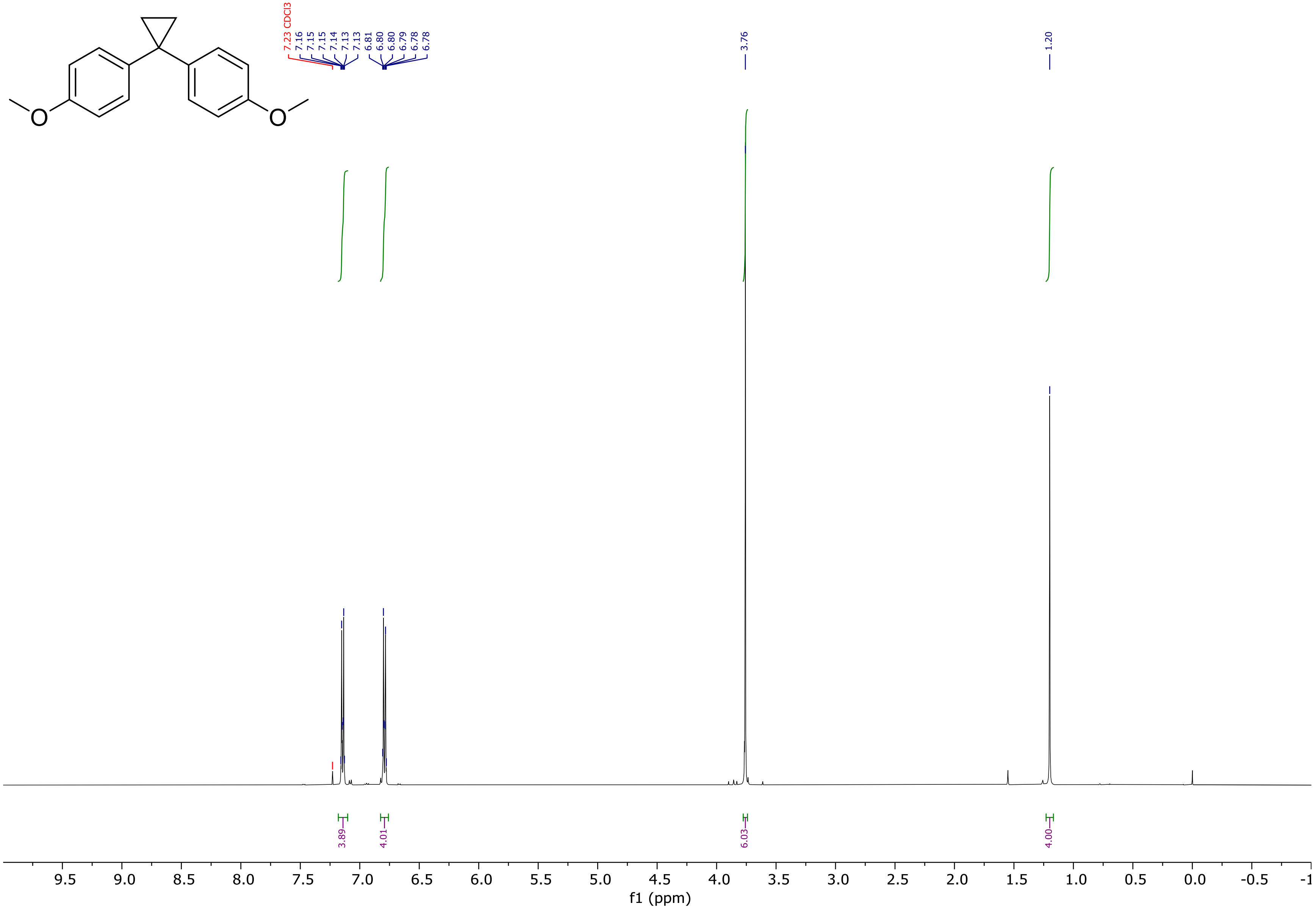

$^{13}C\{^{1}H\}$ NMR spectrum of compound 3j (126 MHz, $CDCl_3$)

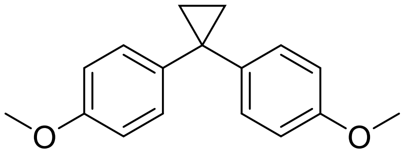

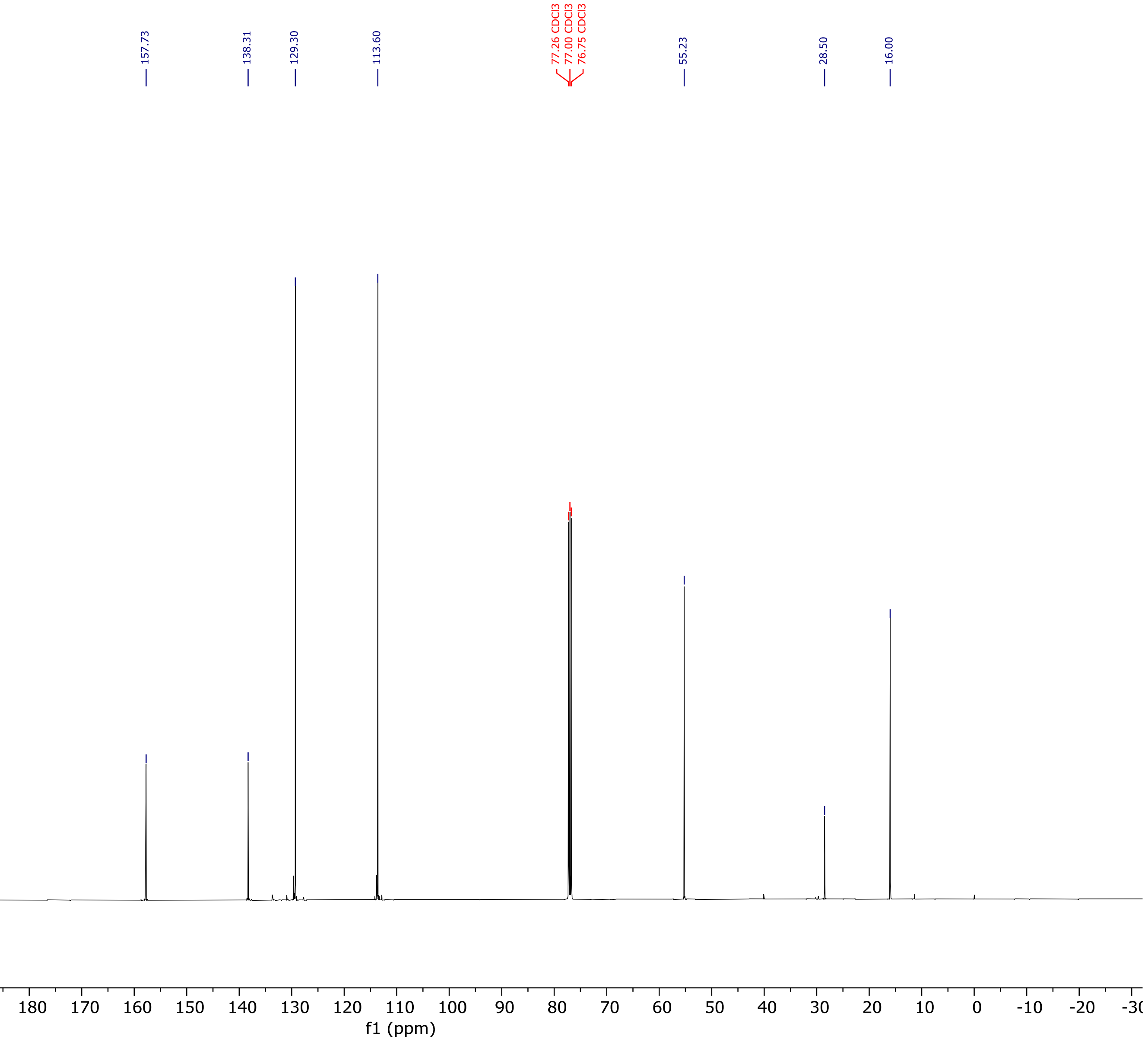

^{1}H NMR spectrum of compound 3k (500 MHz, $CDCl_3$)

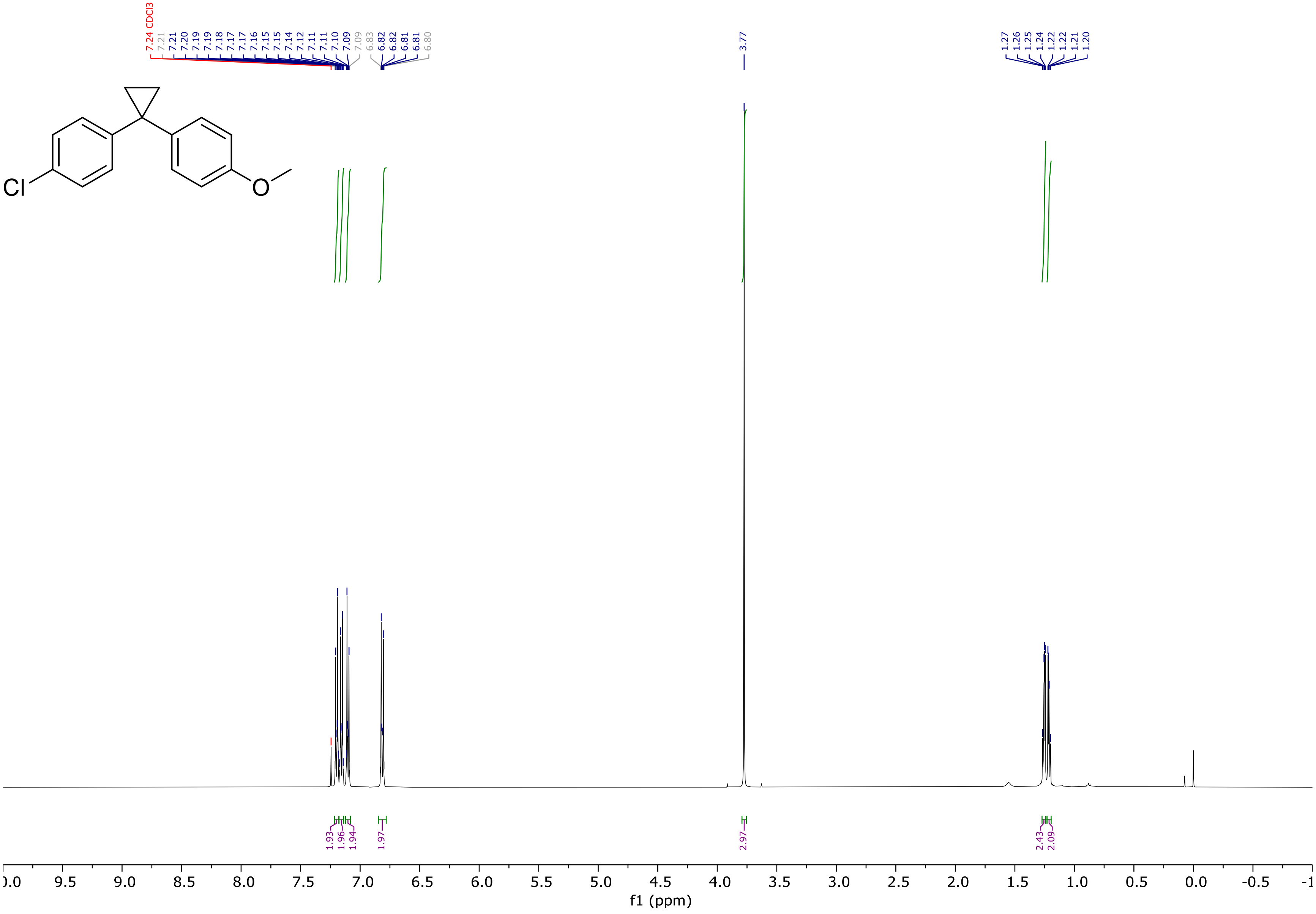

$^{13}C\{^{1}H\}$ NMR spectrum of compound 3k (126 MHz, $CDCl_3$)

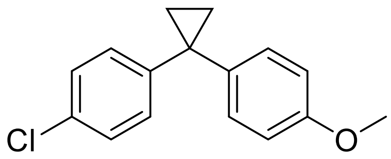

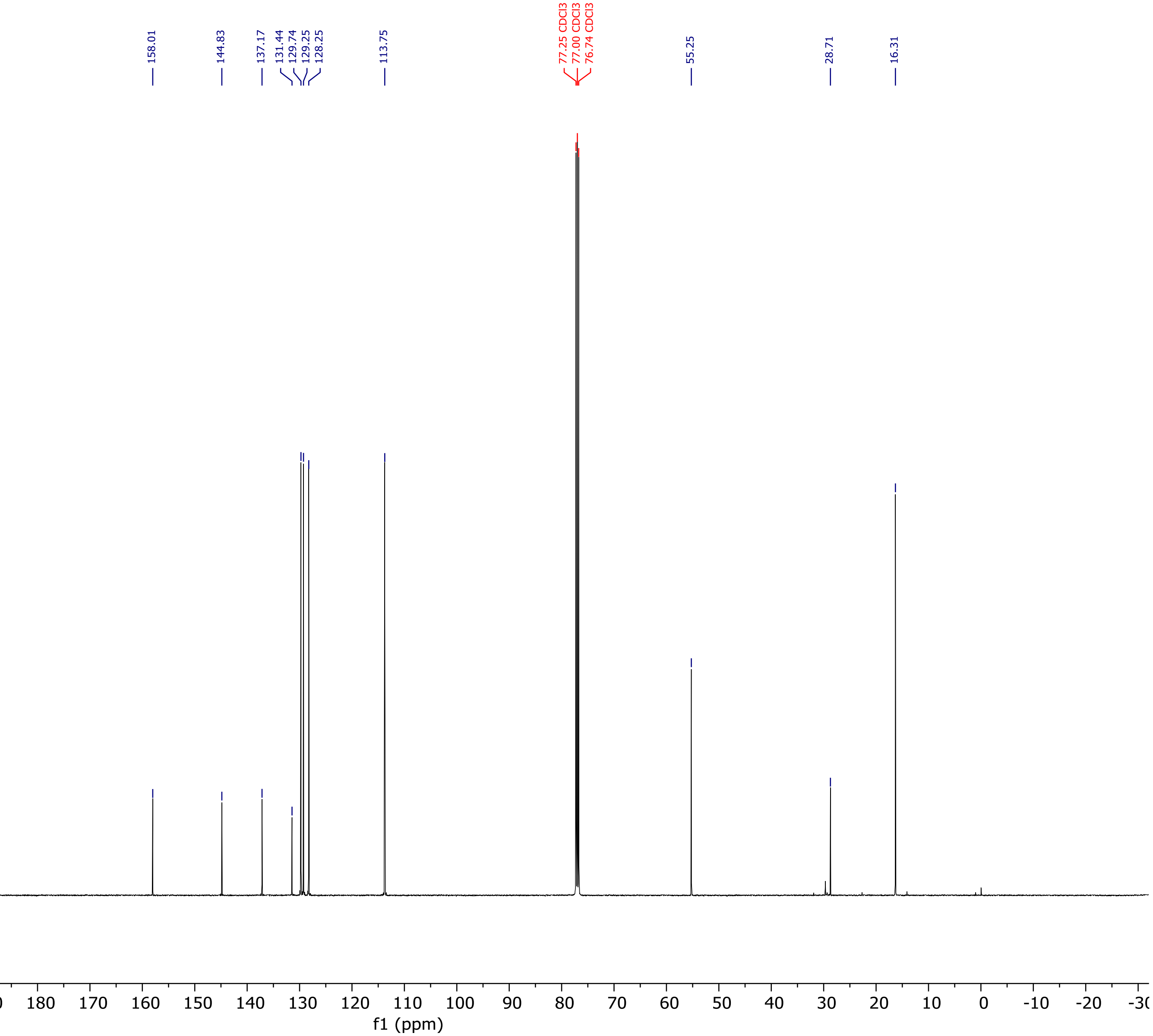

^{1}H NMR spectrum of compound 3l (500 MHz, $CDCl_3$)

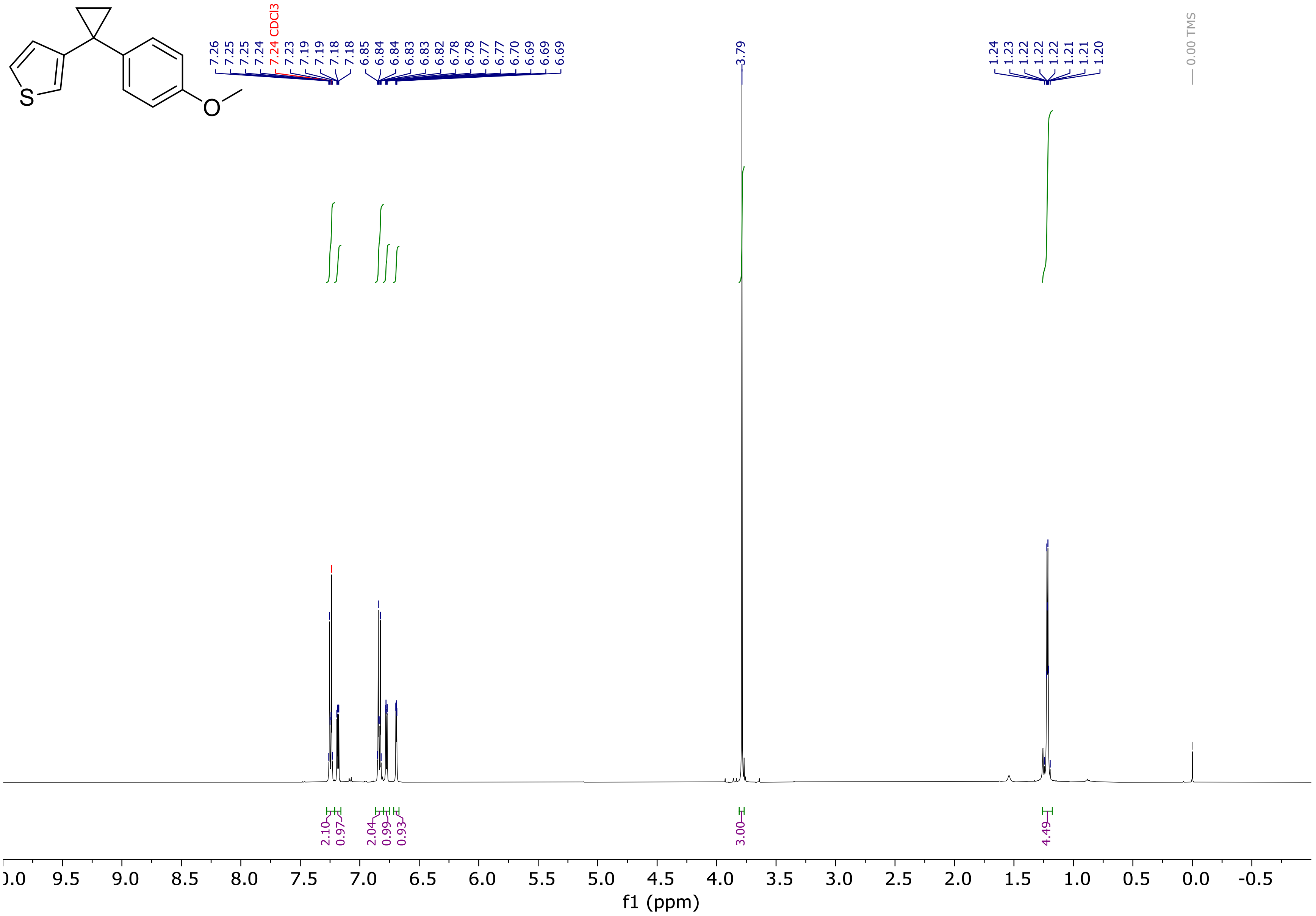

$^{13}C\{^{1}H\}$ NMR spectrum of compound 3l (126 MHz, $CDCl_3$)

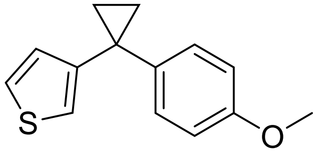

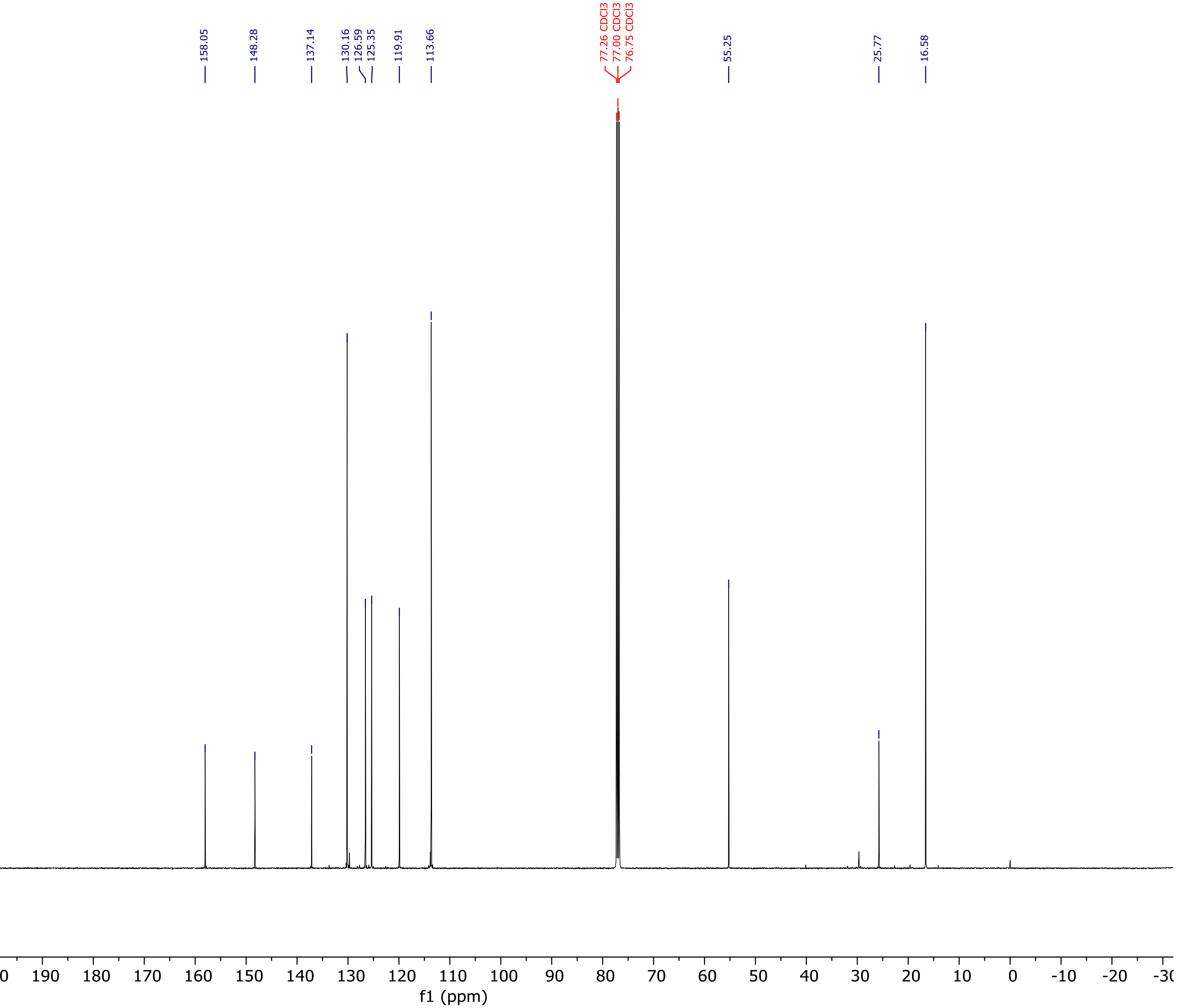

^{1}H NMR spectrum of compound 3m (500 MHz, $CDCl_3$)

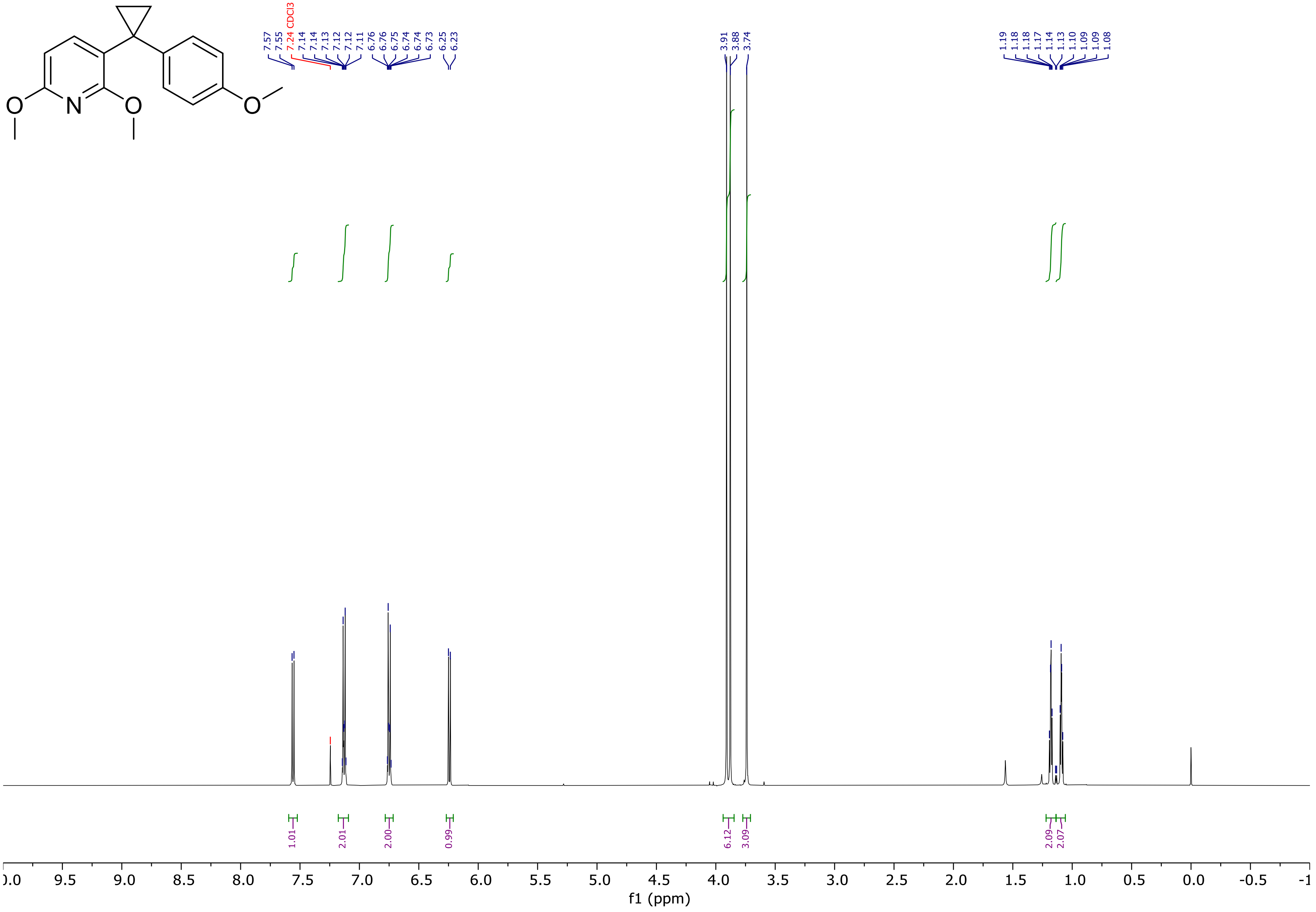

$^{13}C\{^{1}H\}$ NMR spectrum of compound 3m (126 MHz, $CDCl_3$)

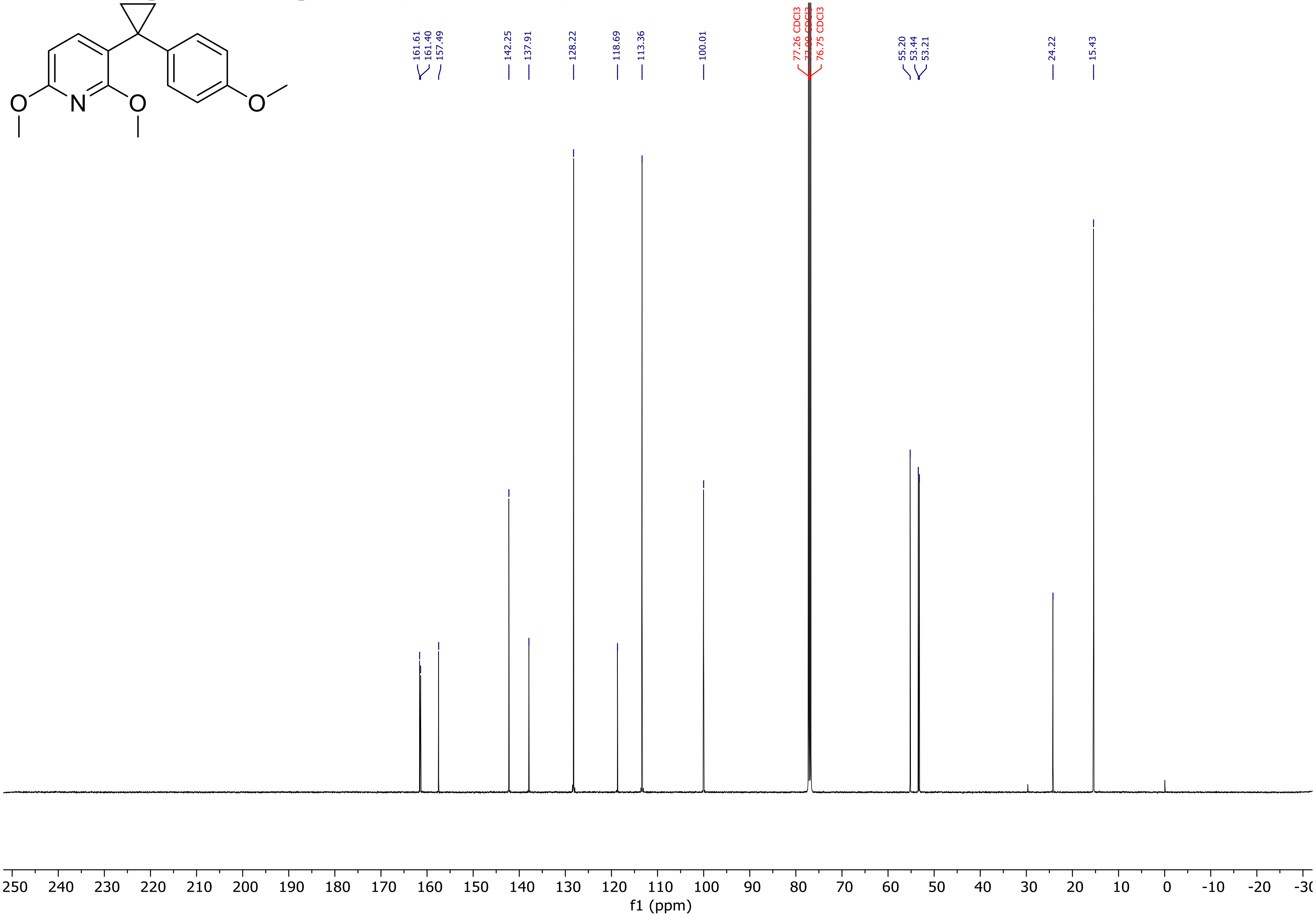

^{1}H NMR spectrum of compound 3n (500 MHz, $CDCl_3$)

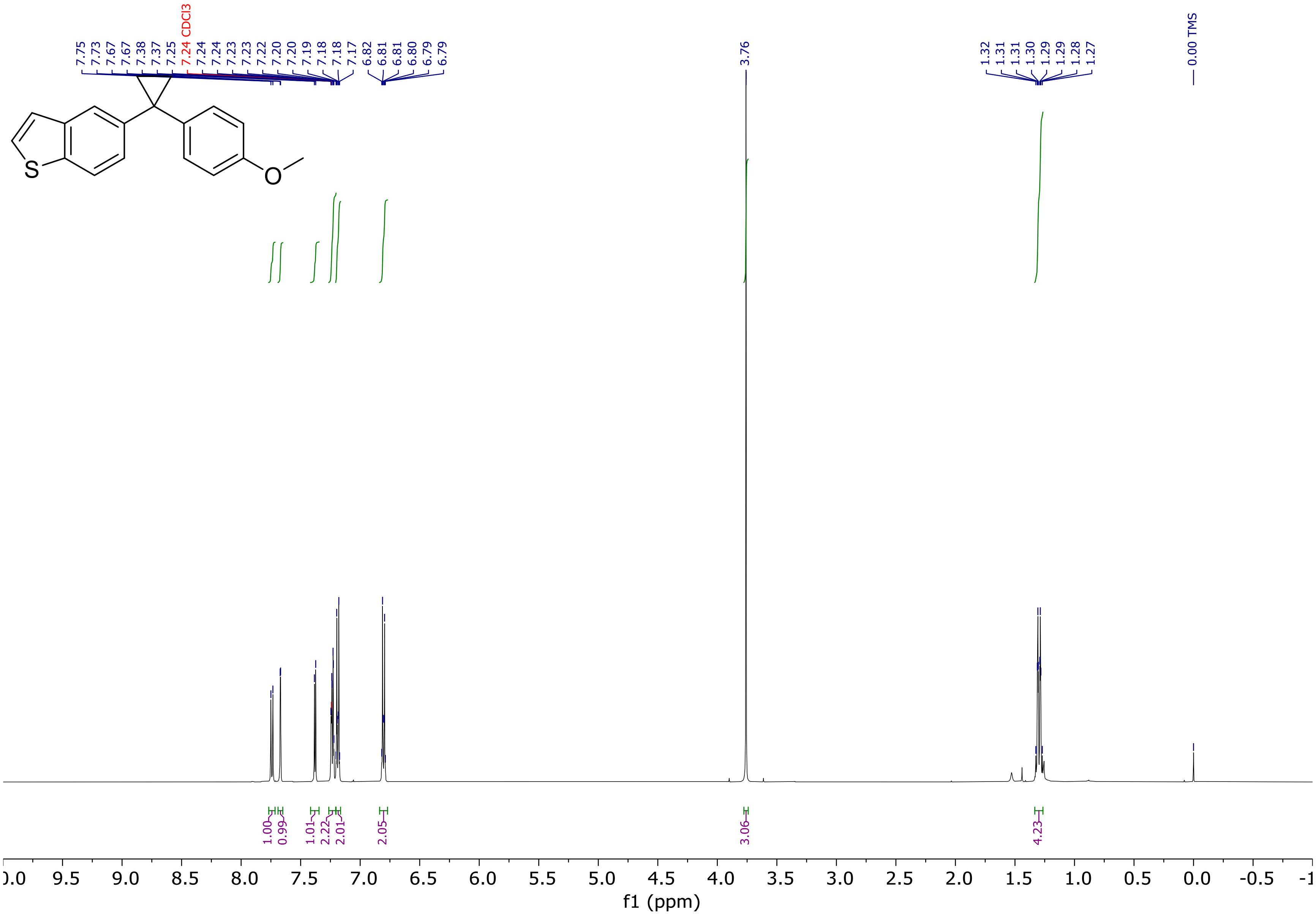

$^{13}C\{^{1}H\}$ NMR spectrum of compound 3n (126 MHz, $CDCl_3$)

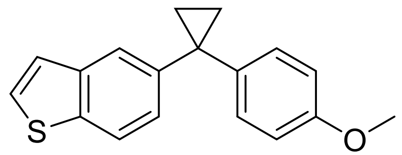

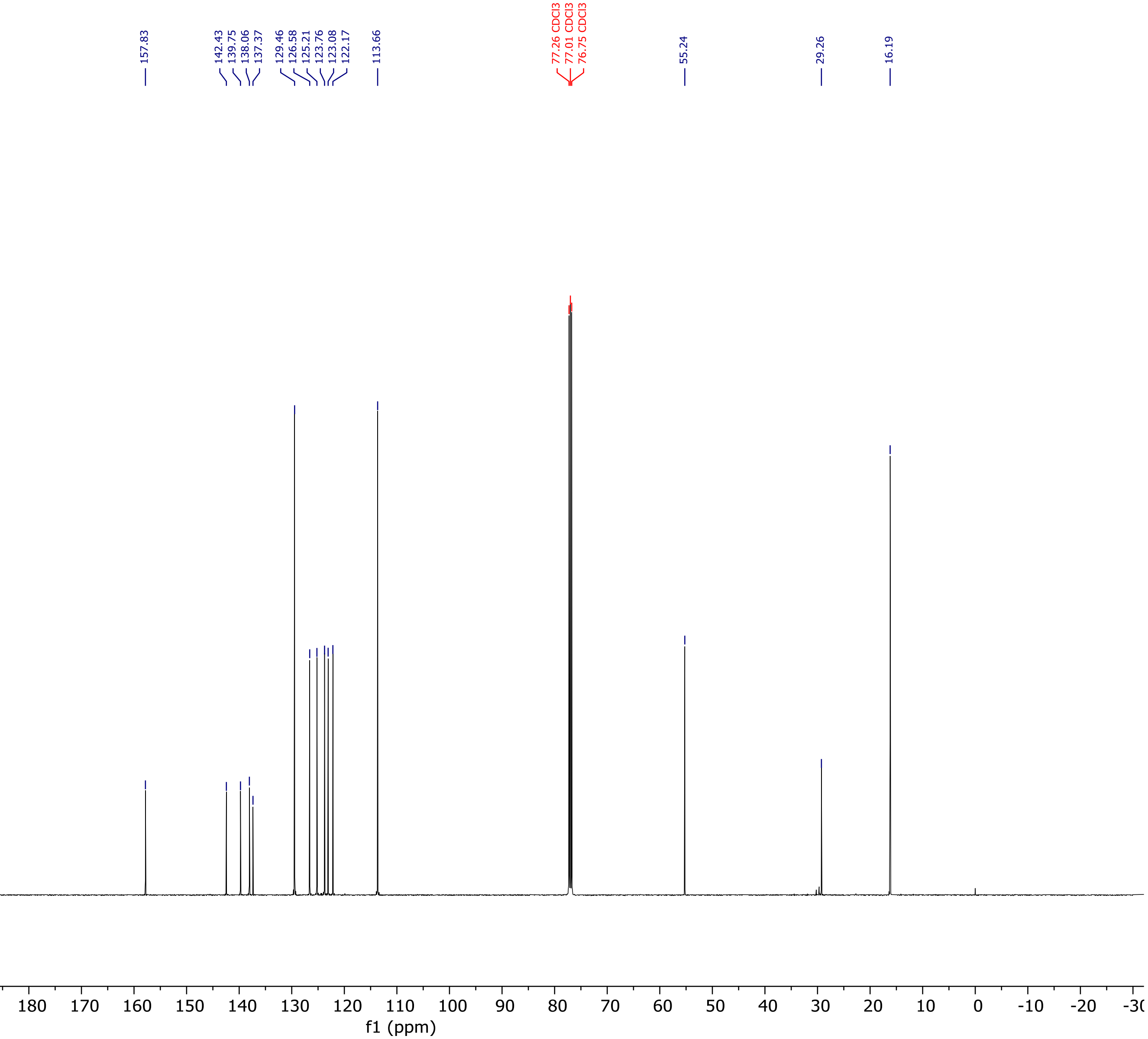

^{1}H NMR spectrum of compound 3o (500 MHz, CDCl$_3$)

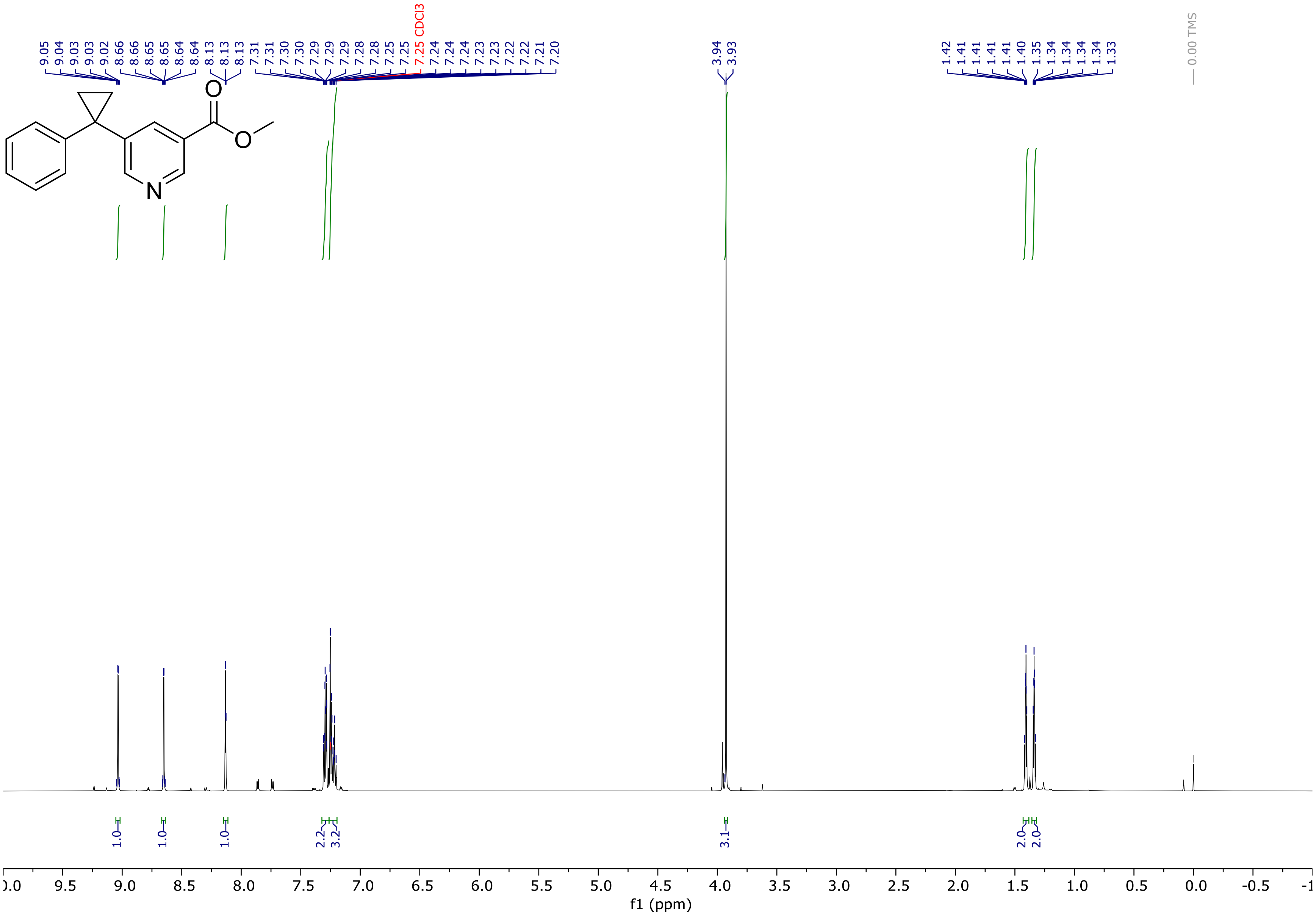

$^{13}C\{^{1}H\}$ NMR spectrum of compound 3o (126 MHz, $CDCl_3$)

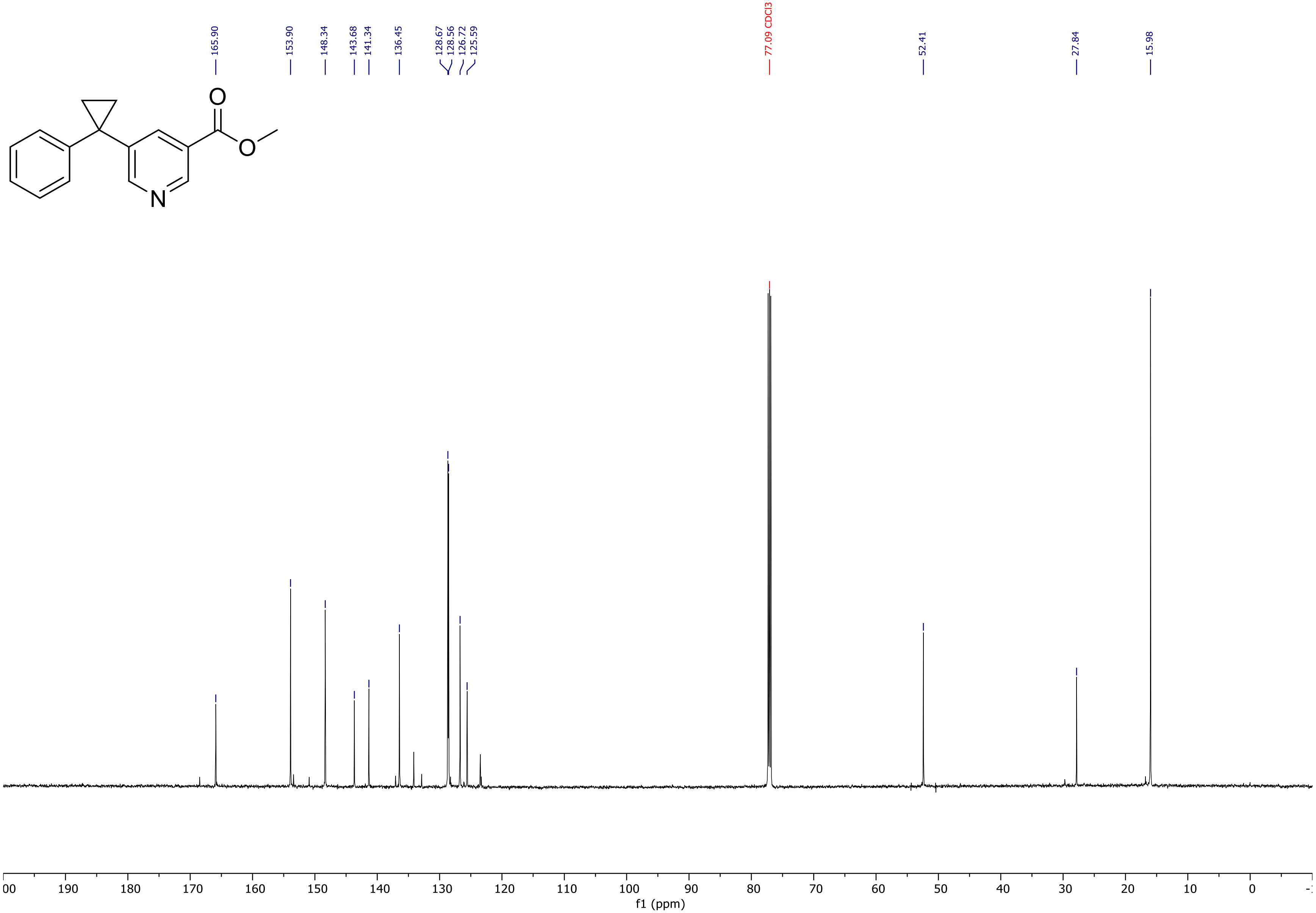

^{1}H NMR spectrum of compound 3p (500 MHz, $CDCl_3$)

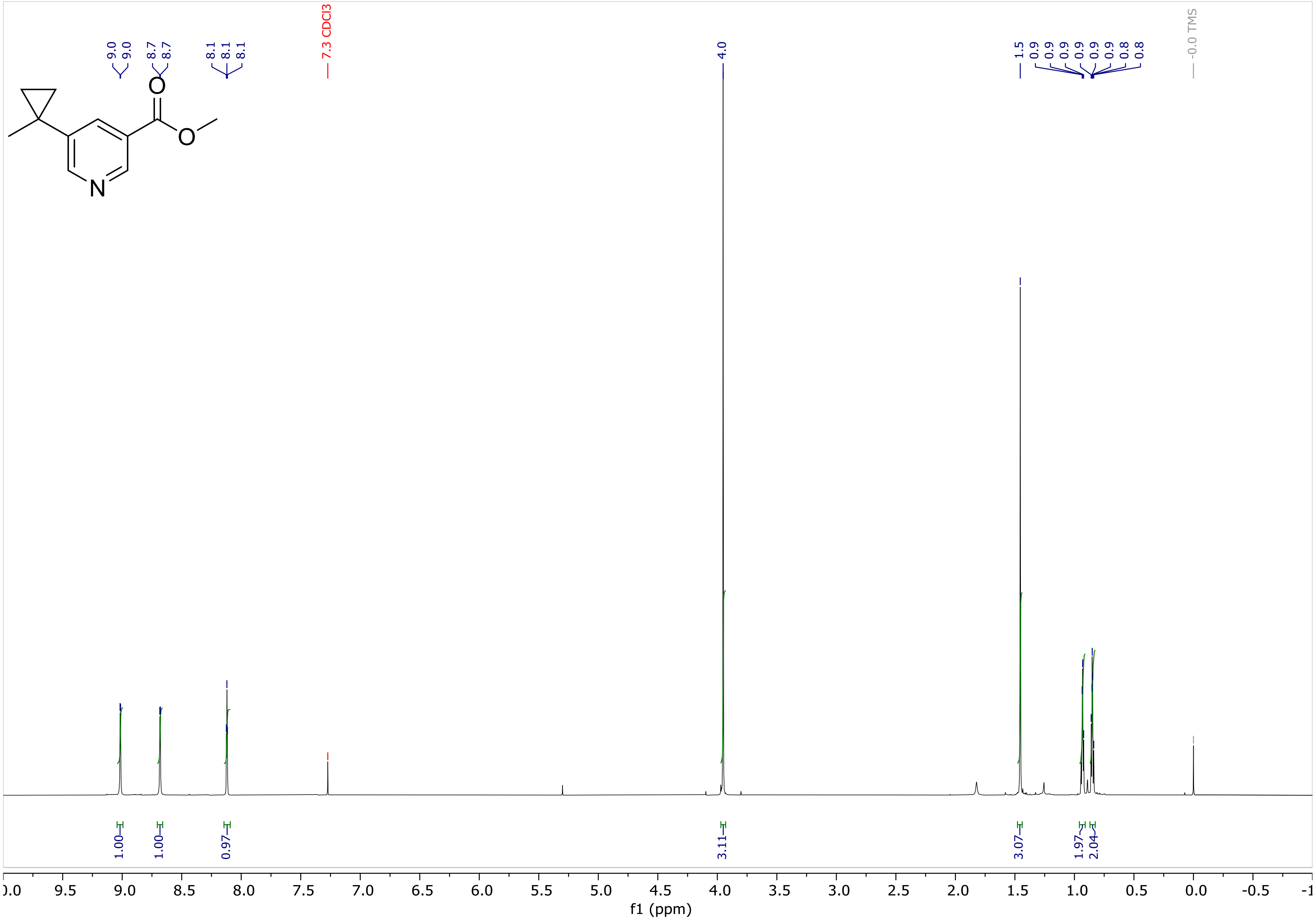

$^{13}C\{^1H\}$ NMR spectrum of compound 3p (126 MHz, $CDCl_3$)

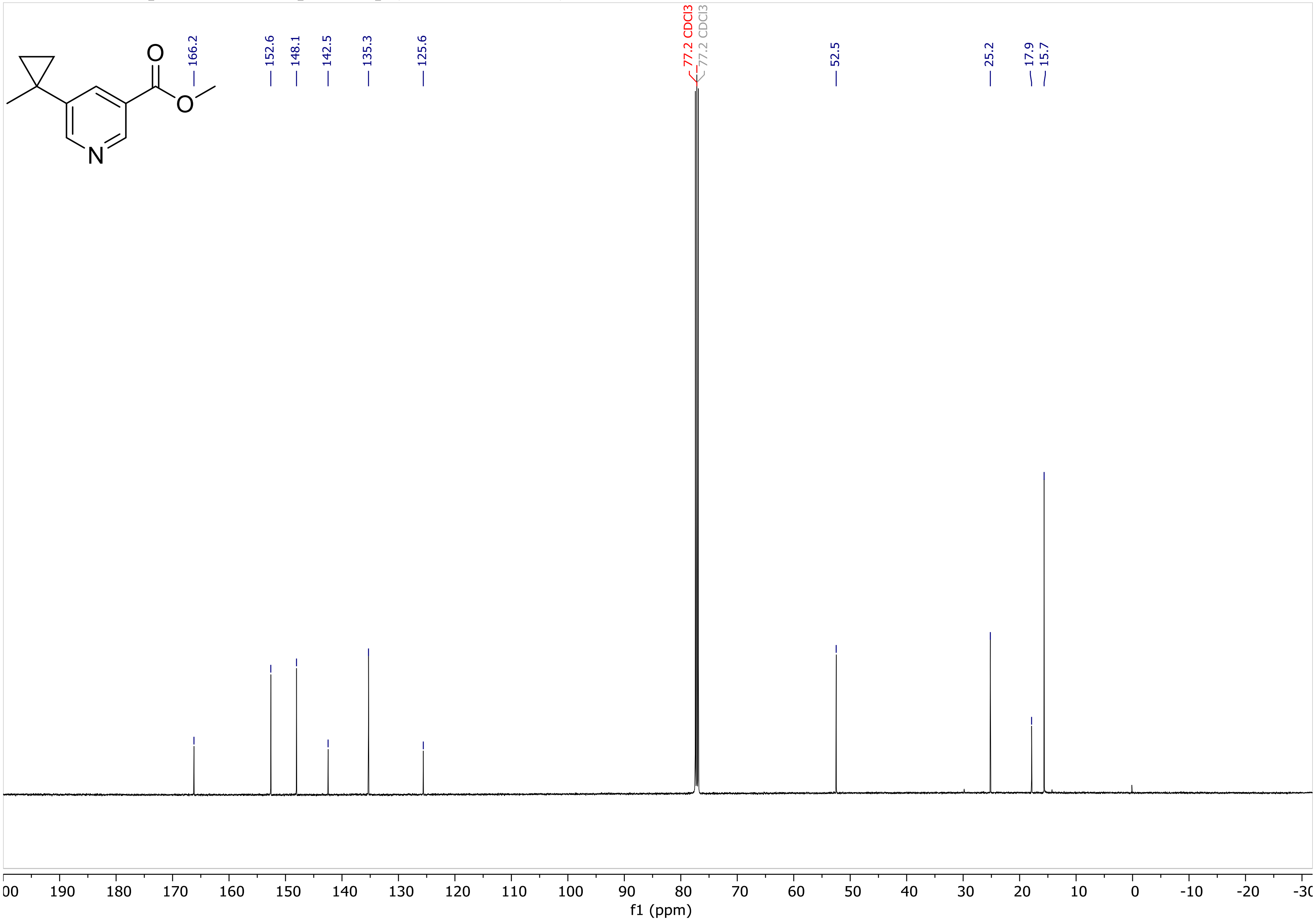

^{1}H NMR spectrum of compound 3q (600 MHz, $CDCl_3$)

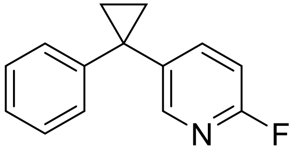

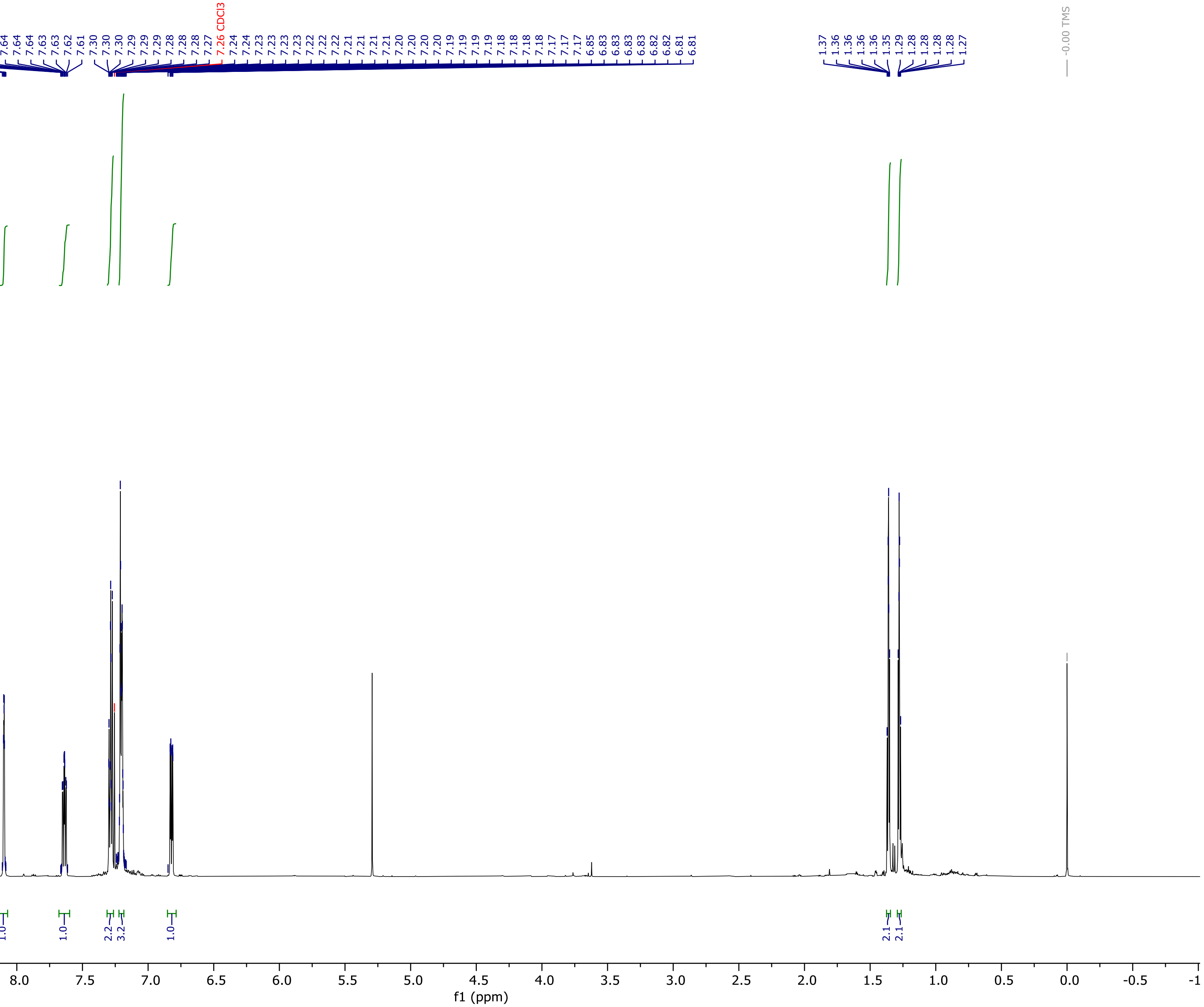

$^{13}C\{^1H\}$ NMR spectrum of compound 3q (151 MHz, $CDCl_3$)

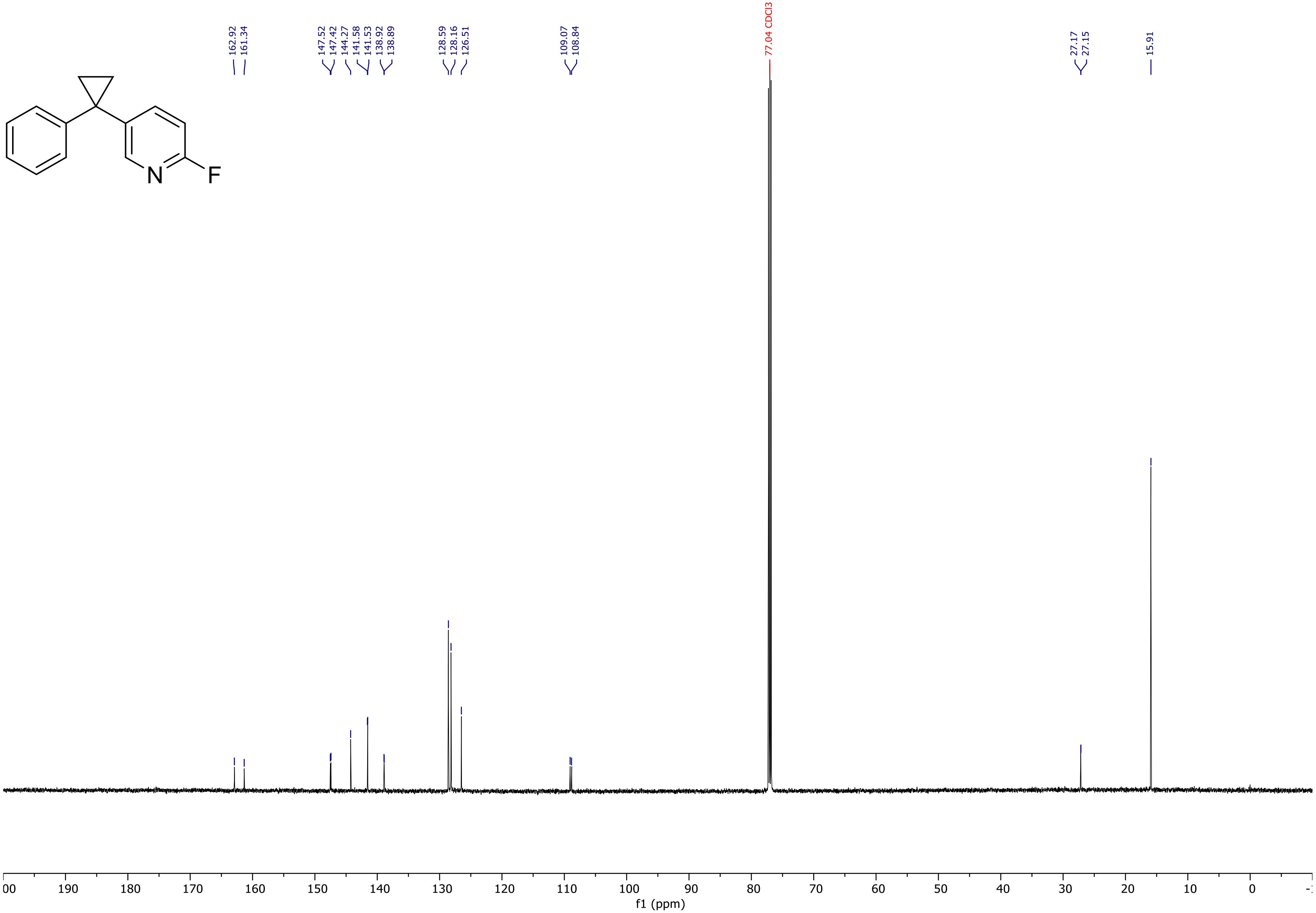

$^{19}F\{^{1}H\}$ NMR spectrum of compound 3q (377 MHz, $CDCl_3$)

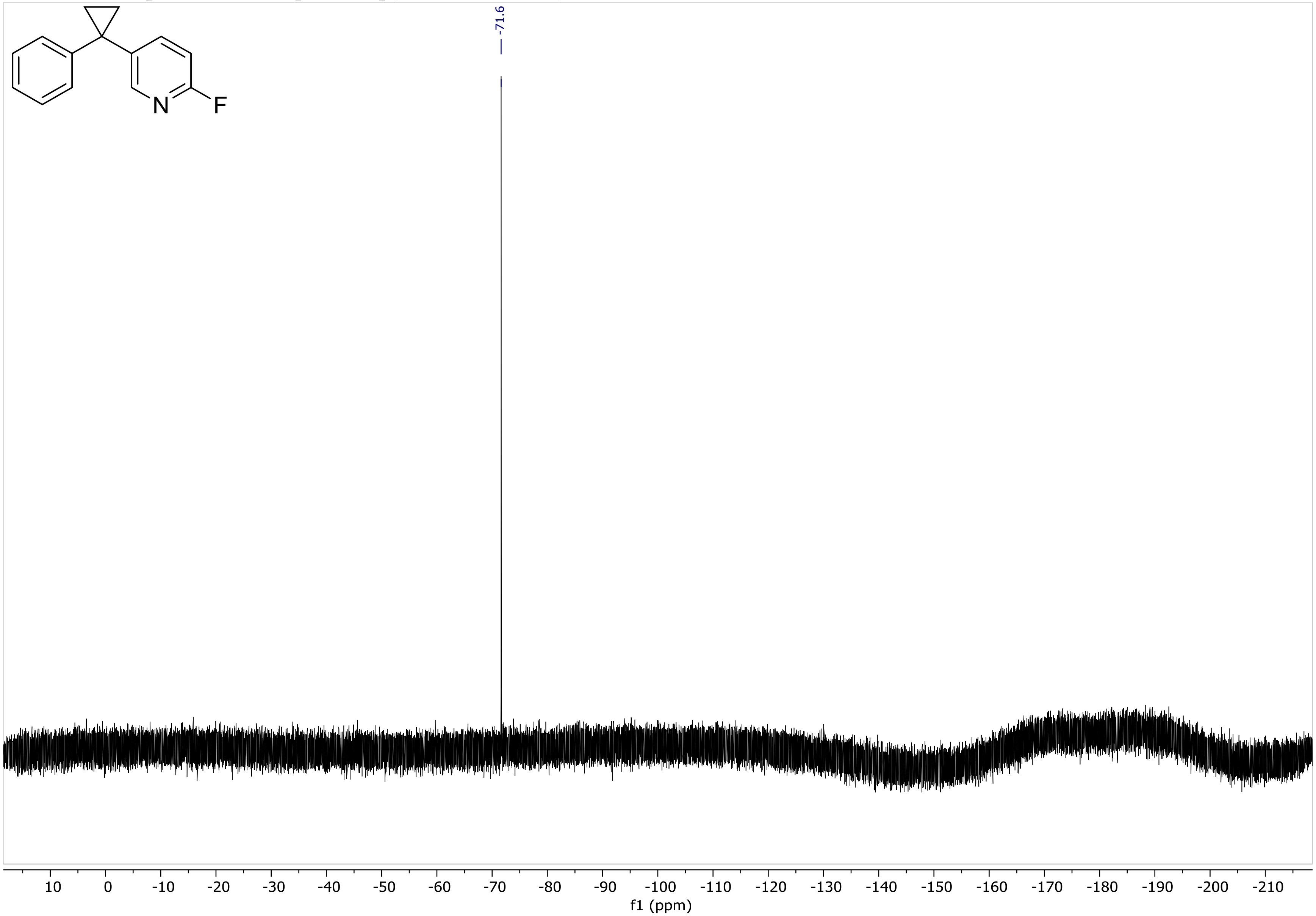

^{1}H NMR spectrum of compound 3r (500 MHz, $CDCl_3$)

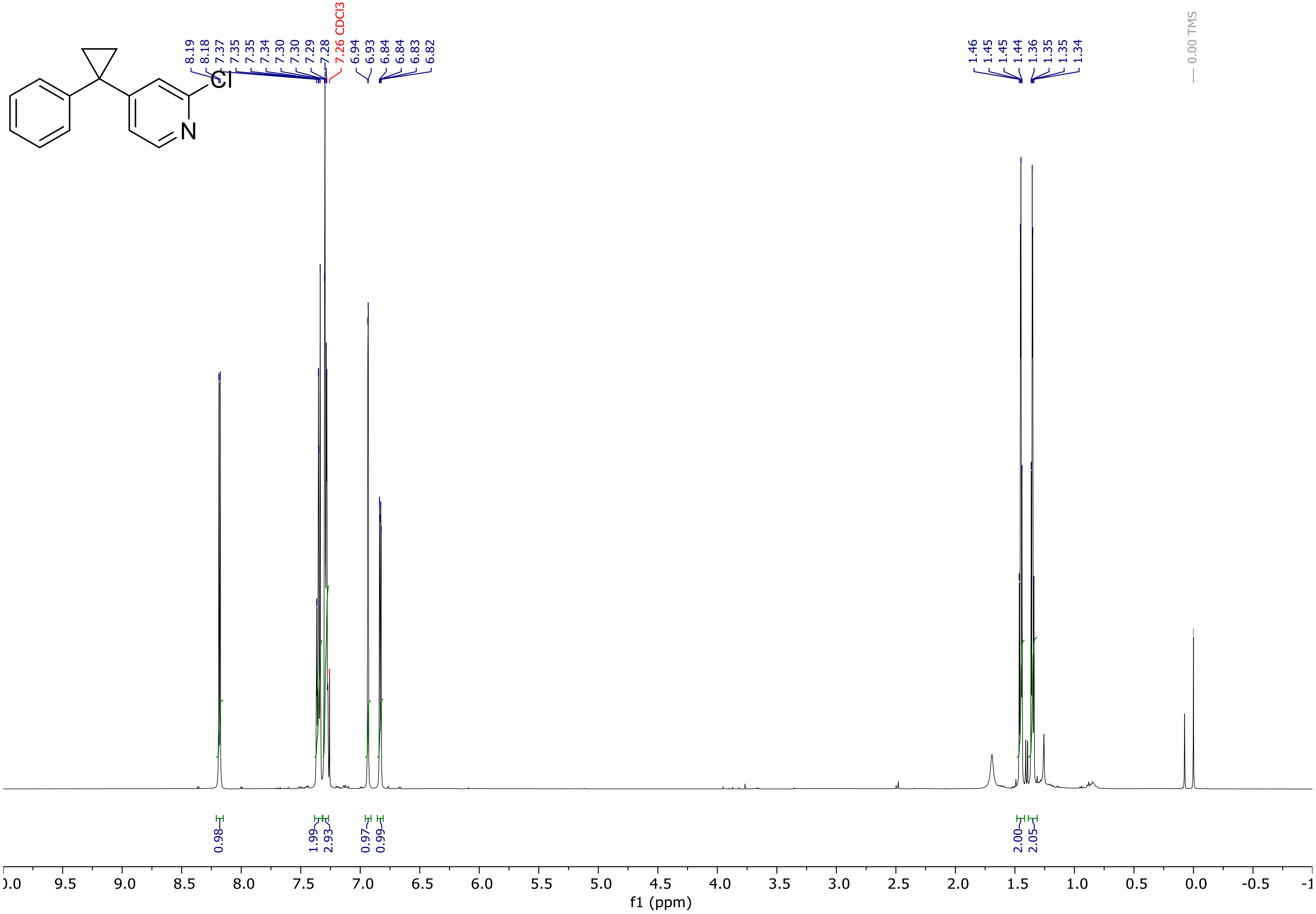

$^{13}C\{^1H\}$ NMR spectrum of compound 3r (126 MHz, $CDCl_3$)

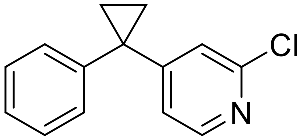

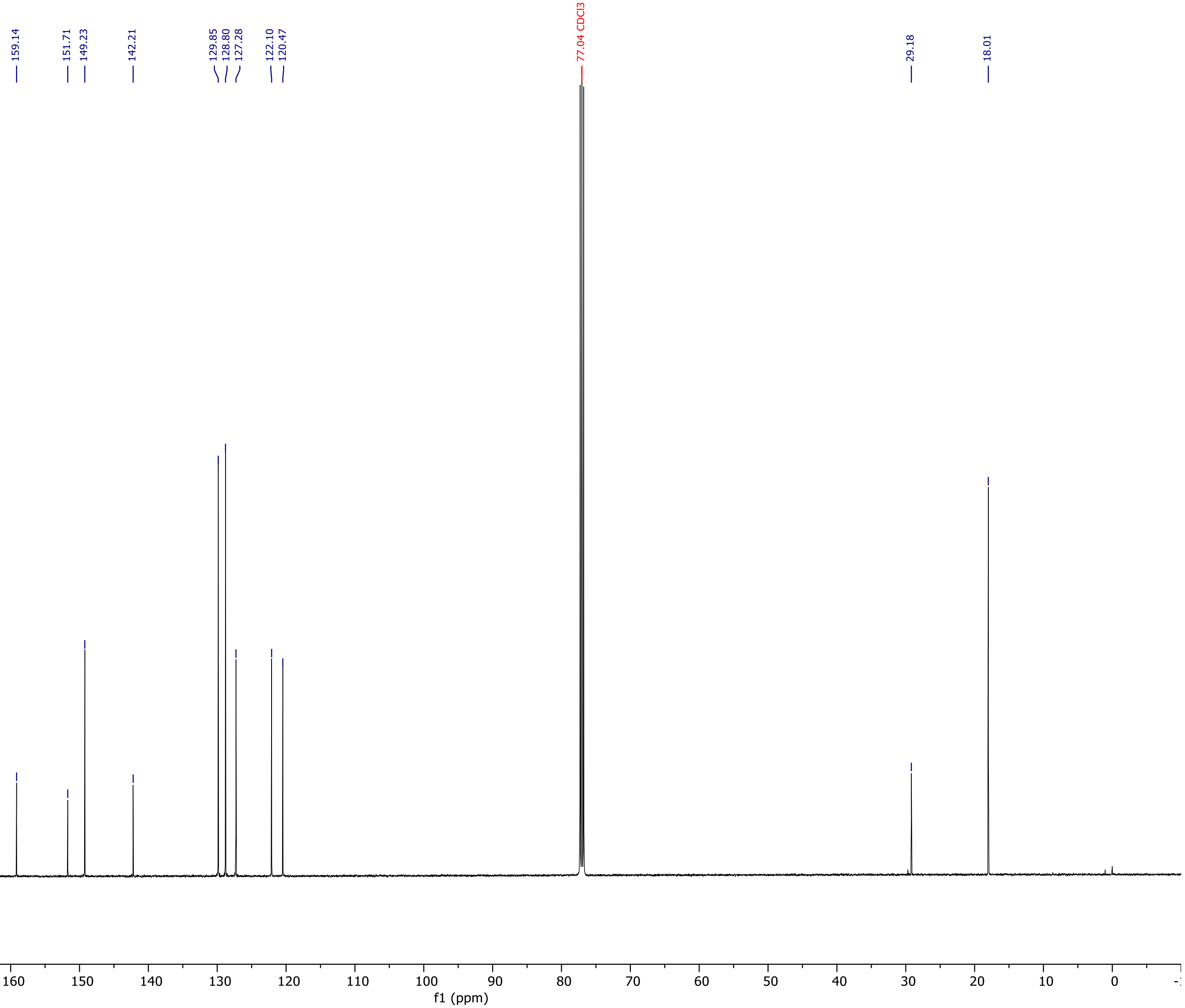

^{1}H NMR spectrum of compound 3s (500 MHz, $CDCl_3$)

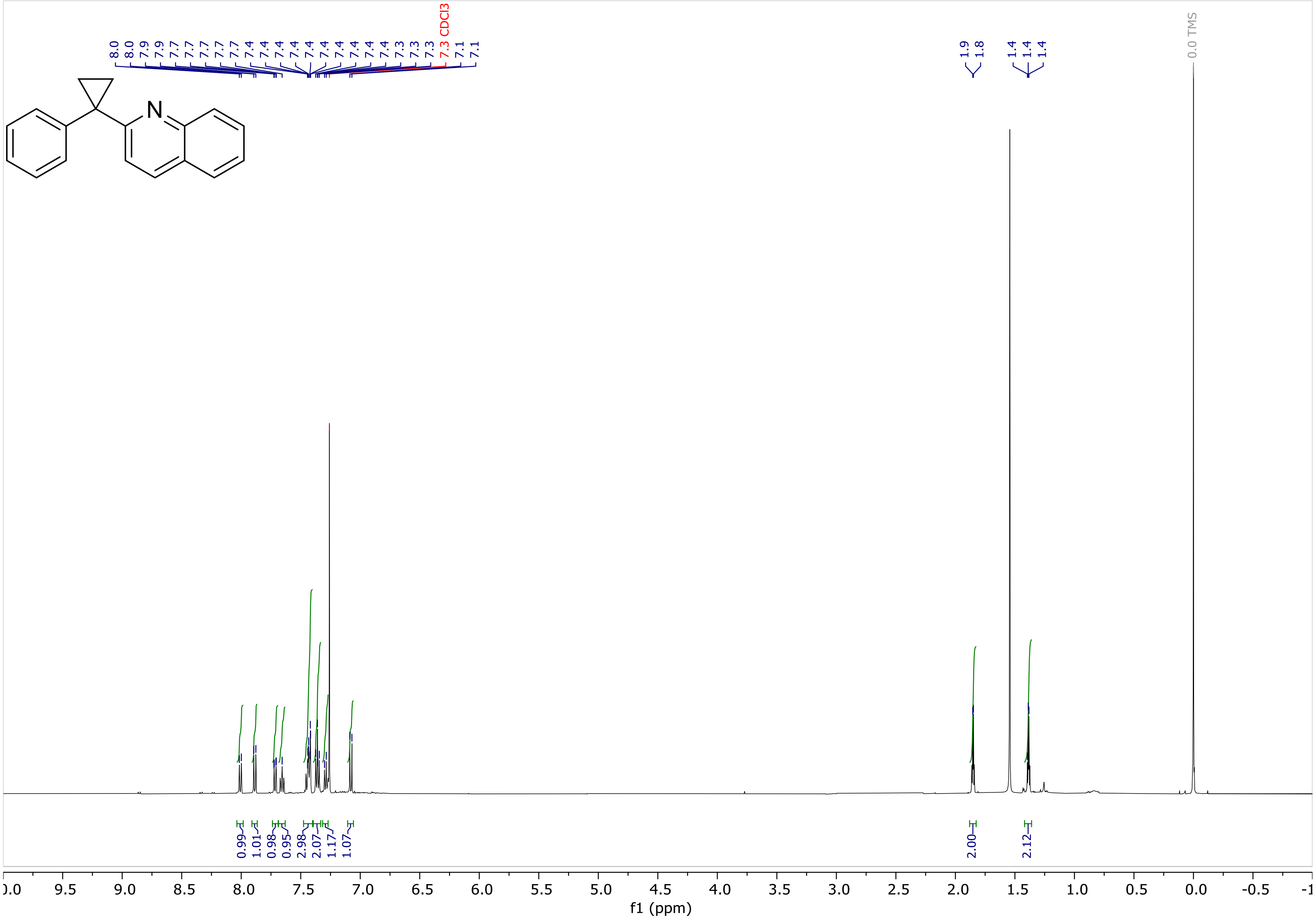

$^{13}C\{^1H\}$ NMR spectrum of compound 3s (126 MHz, $CDCl_3$)

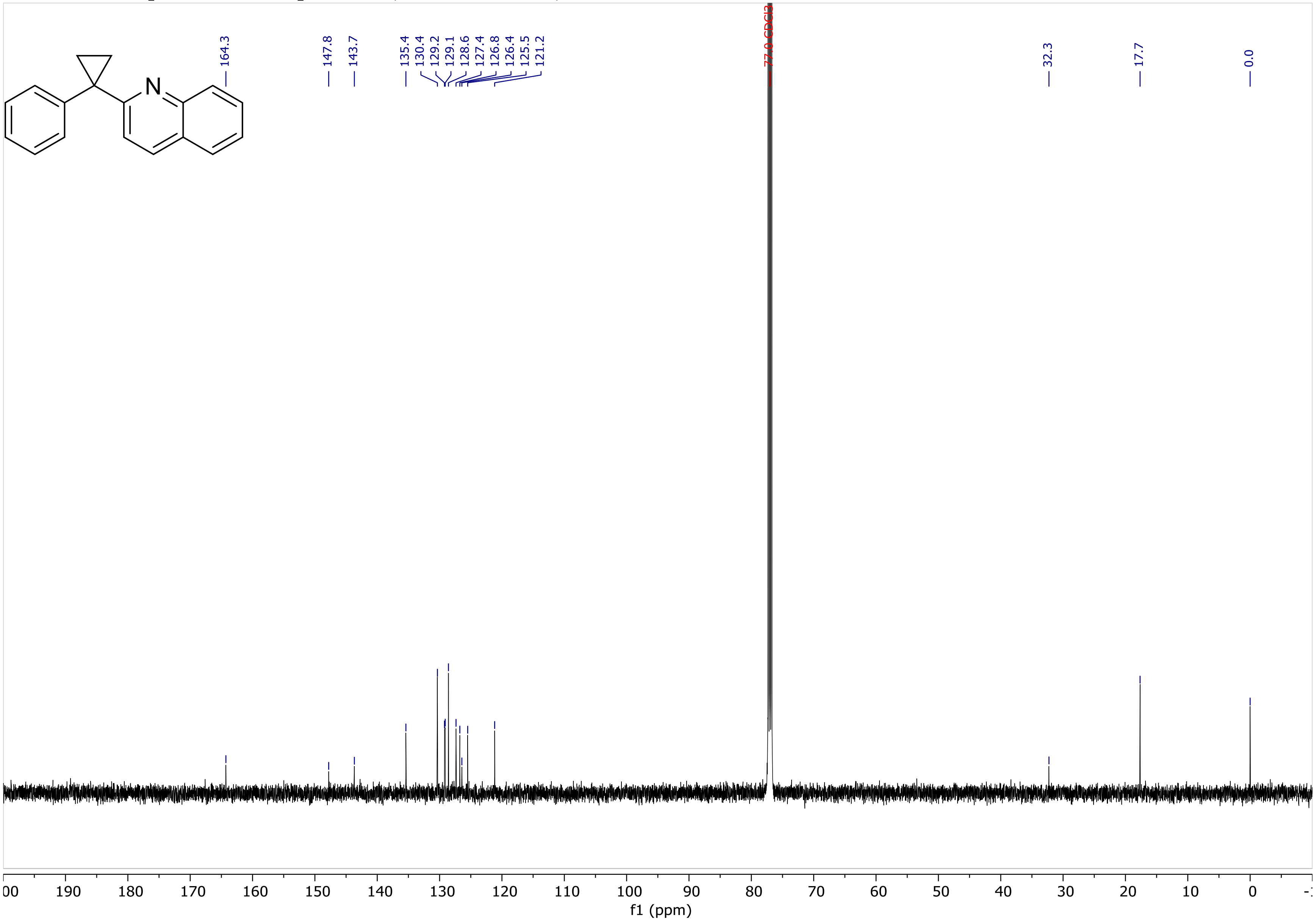

^{1}H NMR spectrum of compound 3t (500 MHz, $CDCl_3$)

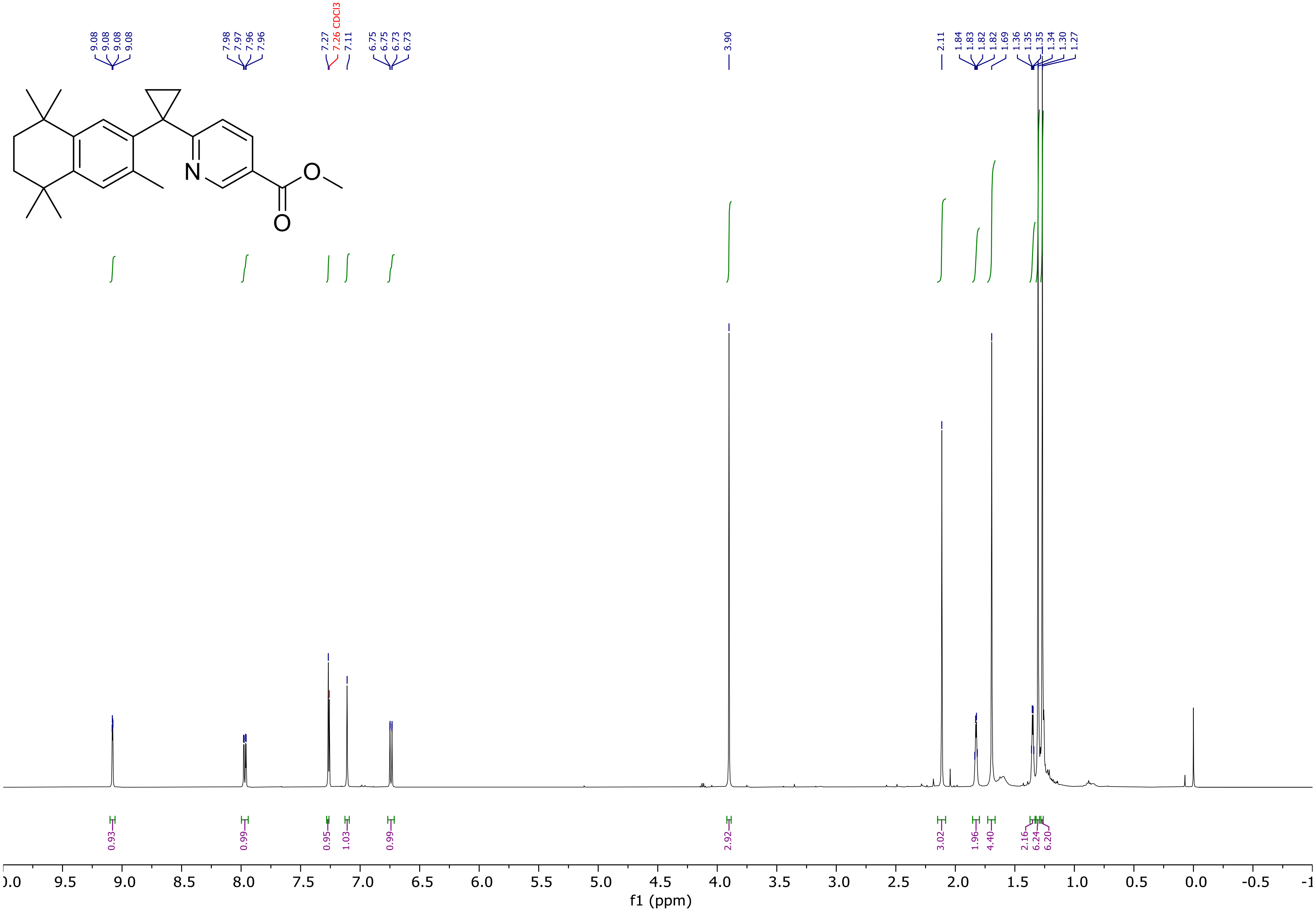

$^{13}C\{^1H\}$ NMR spectrum of compound 3t (126 MHz, $CDCl_3$)

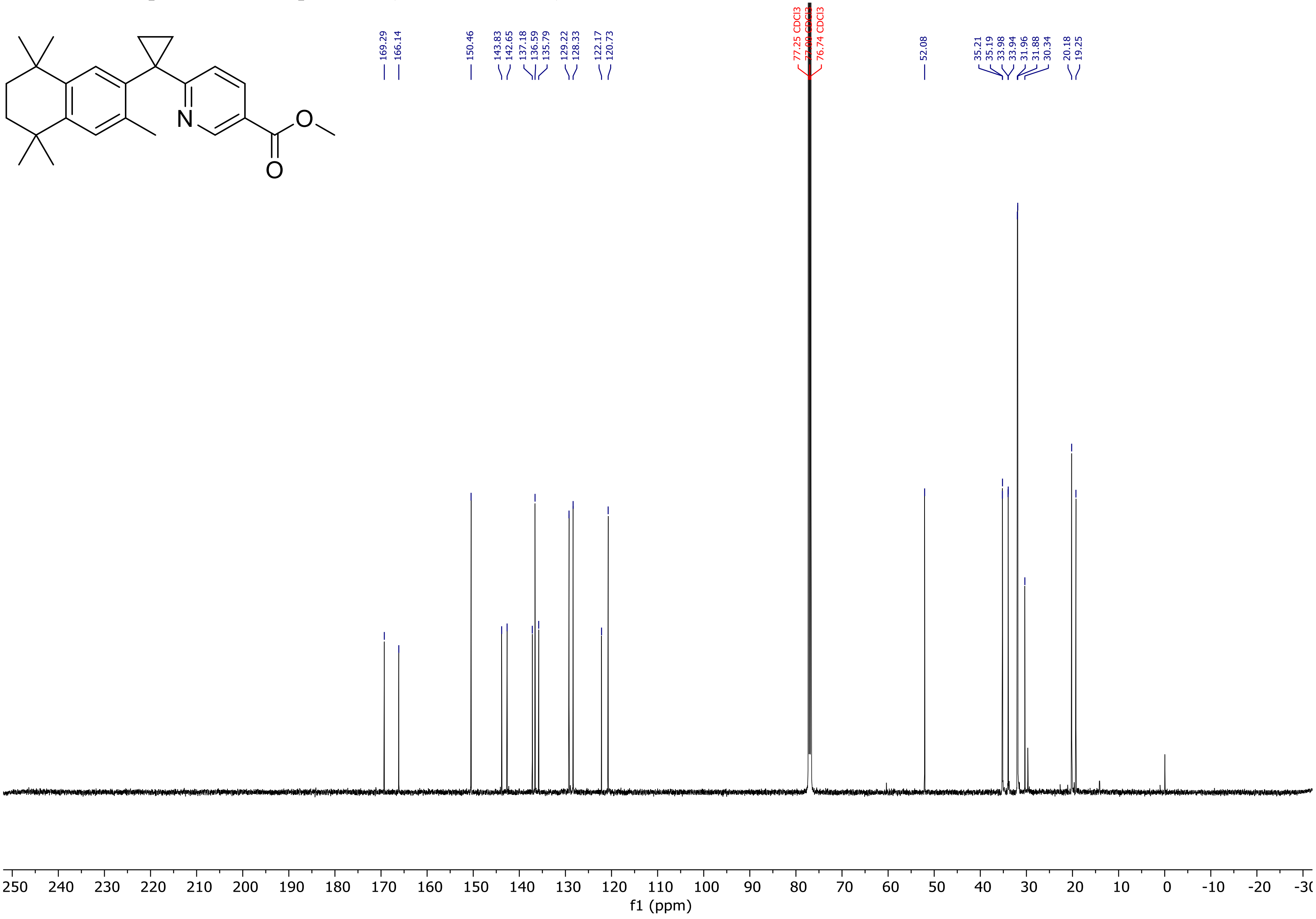

^{1}H NMR spectrum of compound 3u (600 MHz, $CDCl_3$)

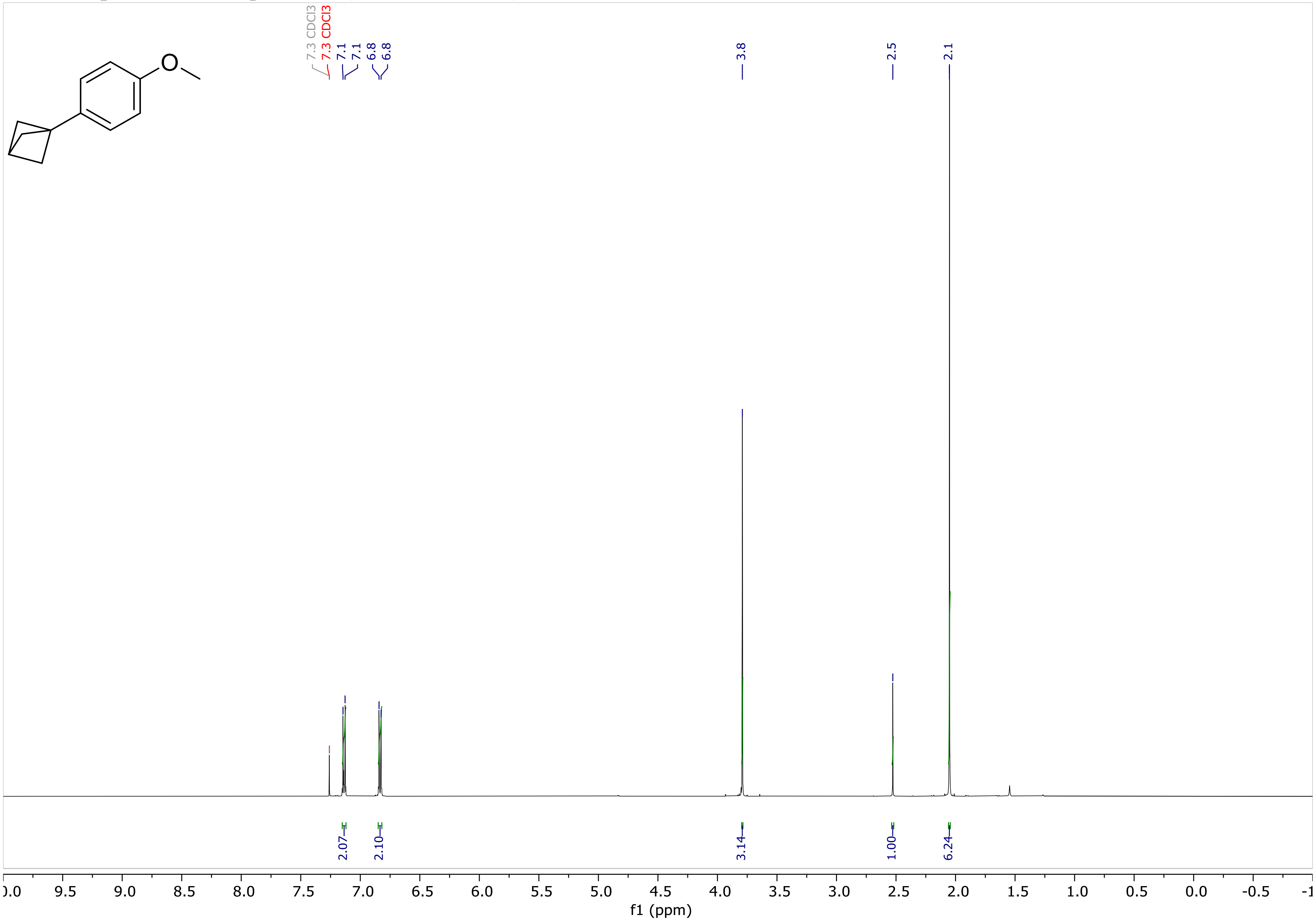

$^{13}C\{^1H\}$ NMR spectrum of compound 3u (151 MHz, $CDCl_3$)

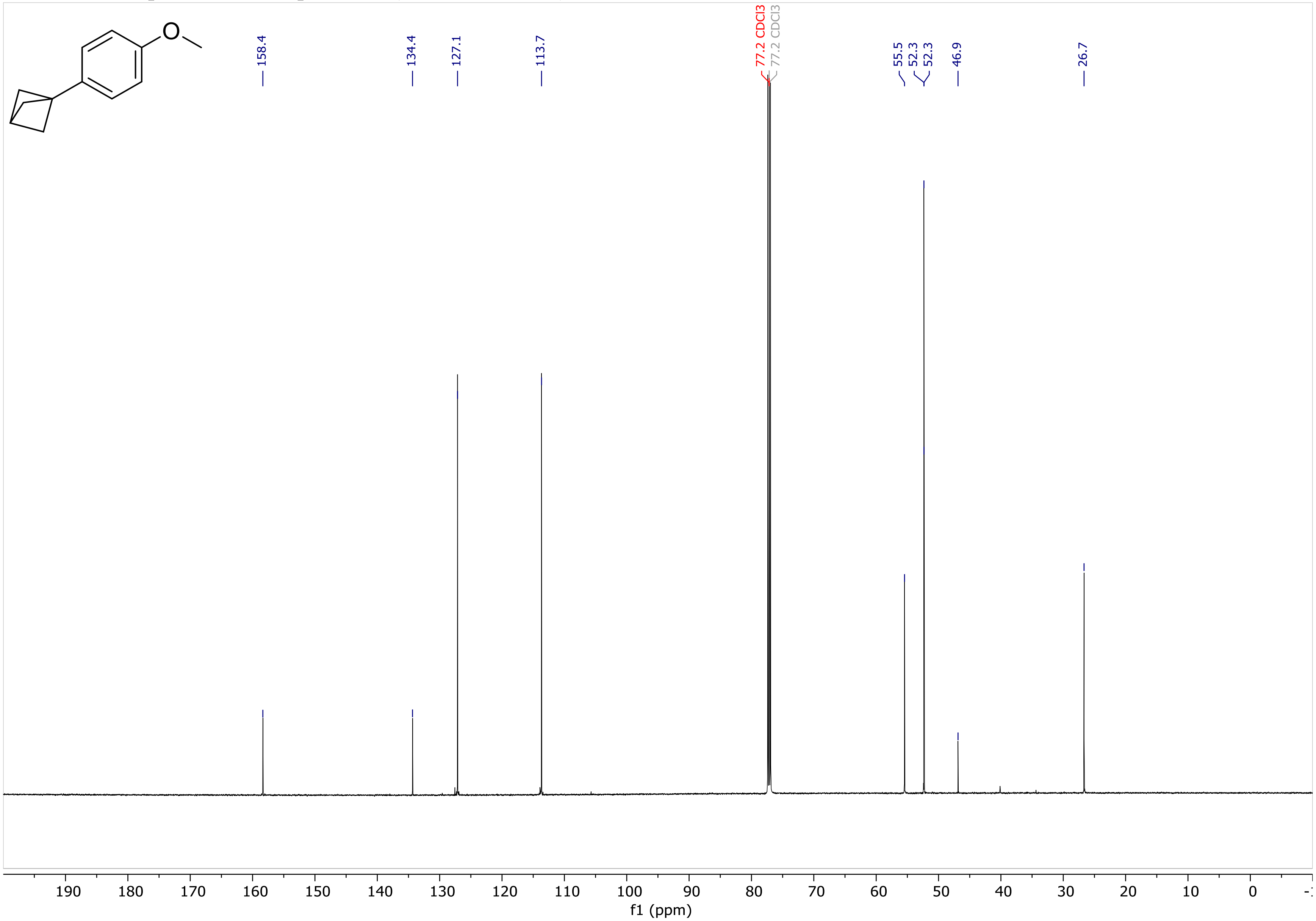

^{1}H NMR spectrum of compound 3v (600 MHz, $CDCl_3$)

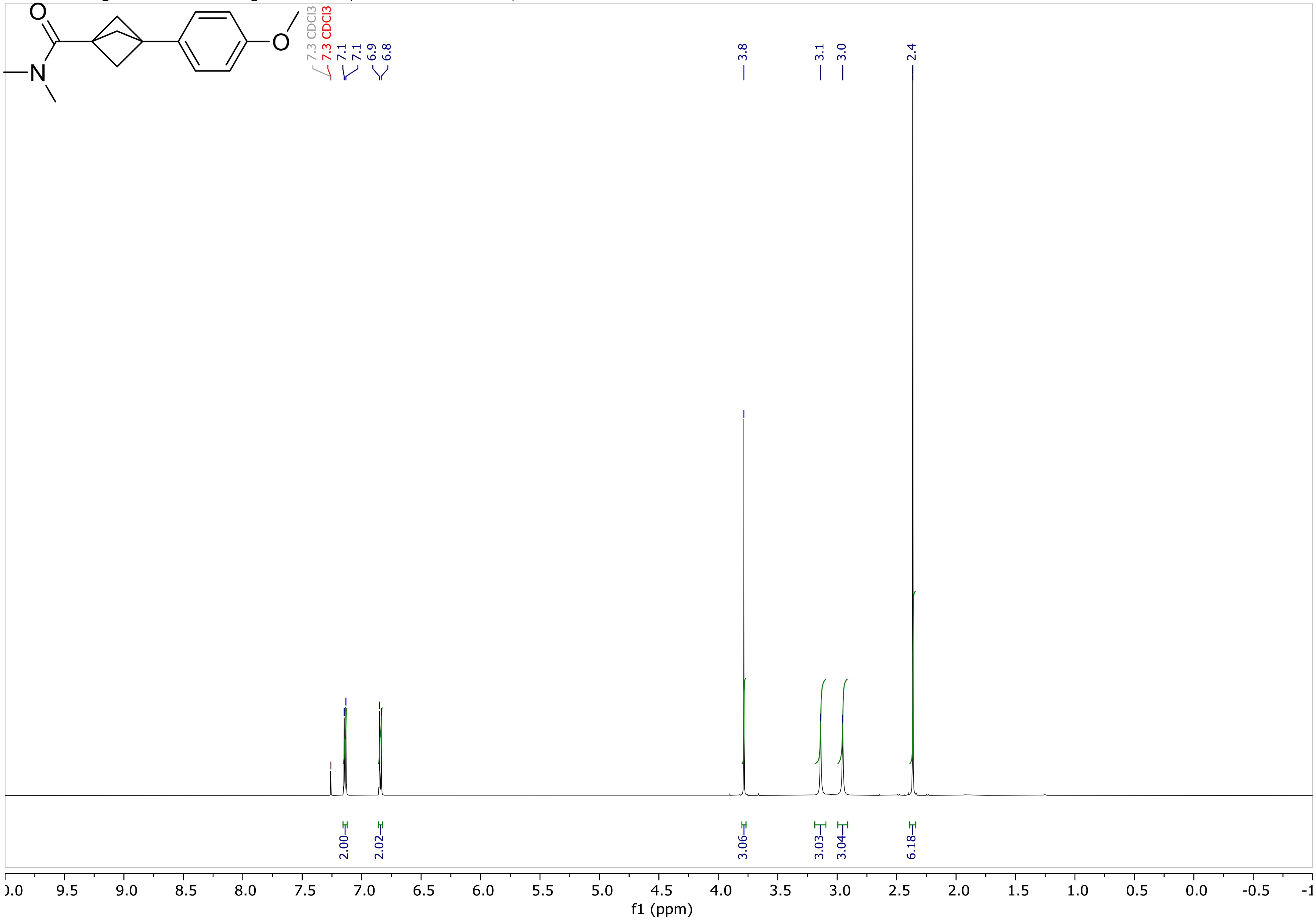

$^{13}C\{^{1}H\}$ NMR spectrum of compound 3v (151 MHz, $CDCl_3$)

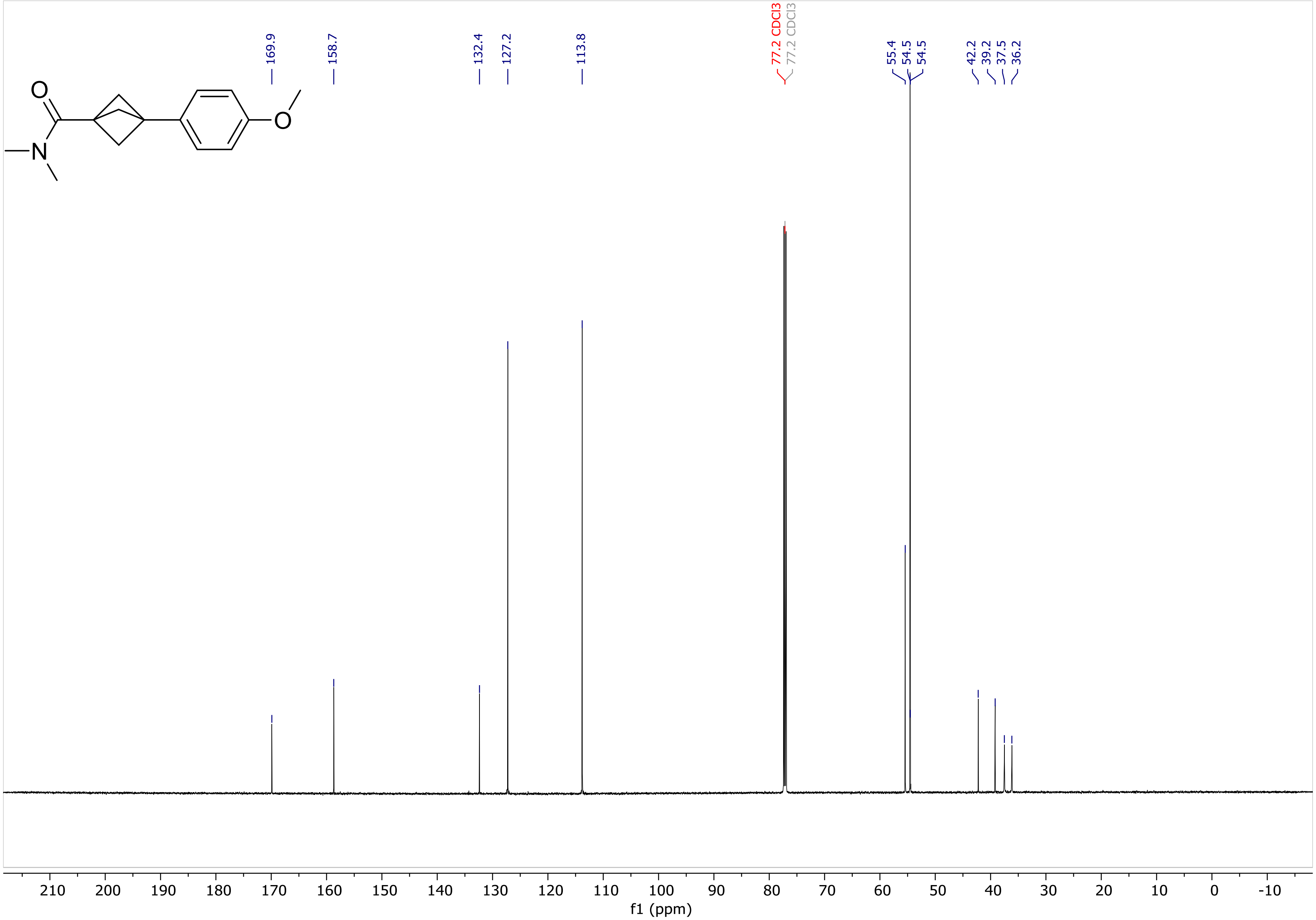

^{1}H NMR spectrum of compound 3w (500 MHz, $CDCl_3$)

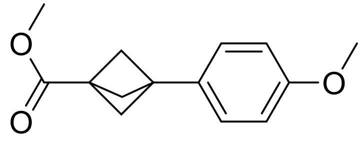

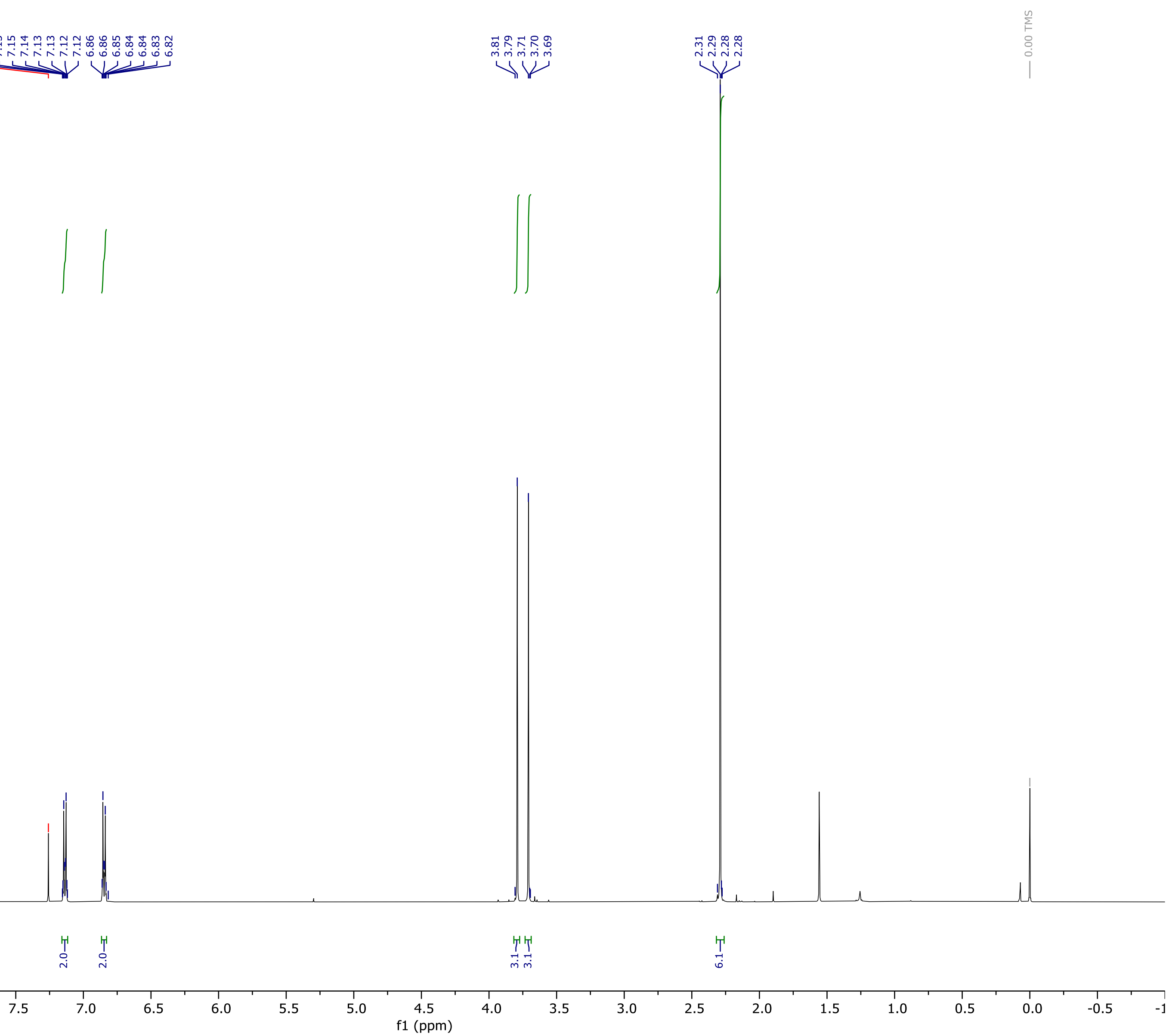

$^{13}C\{^{1}H\}$ NMR spectrum of compound 3w (126 MHz, $CDCl_3$)

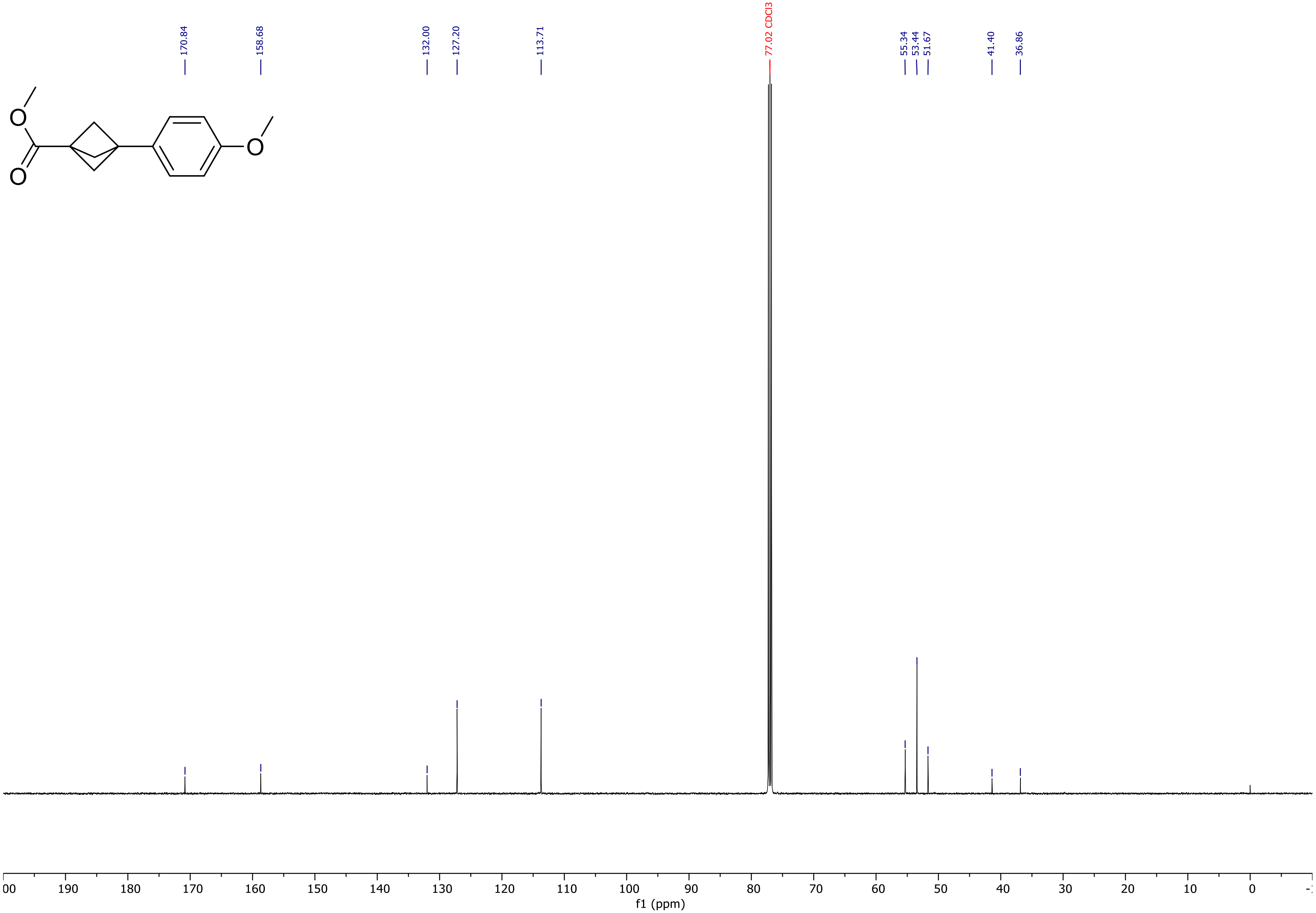

^{1}H NMR spectrum of compound 3x (500 MHz, $CDCl_3$)

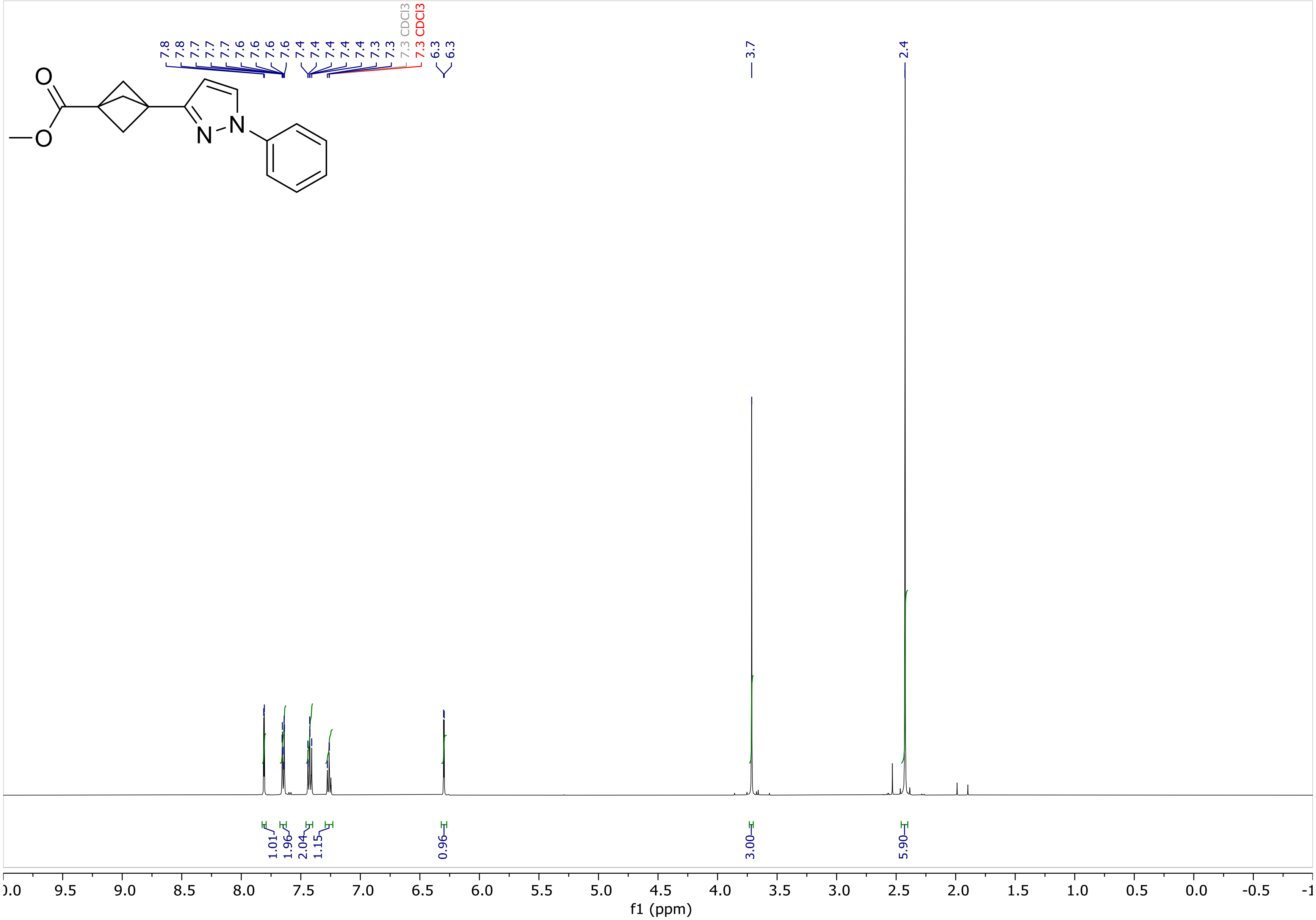

$^{13}C\{^1H\}$ NMR spectrum of compound 3x (126 MHz, $CDCl_3$)

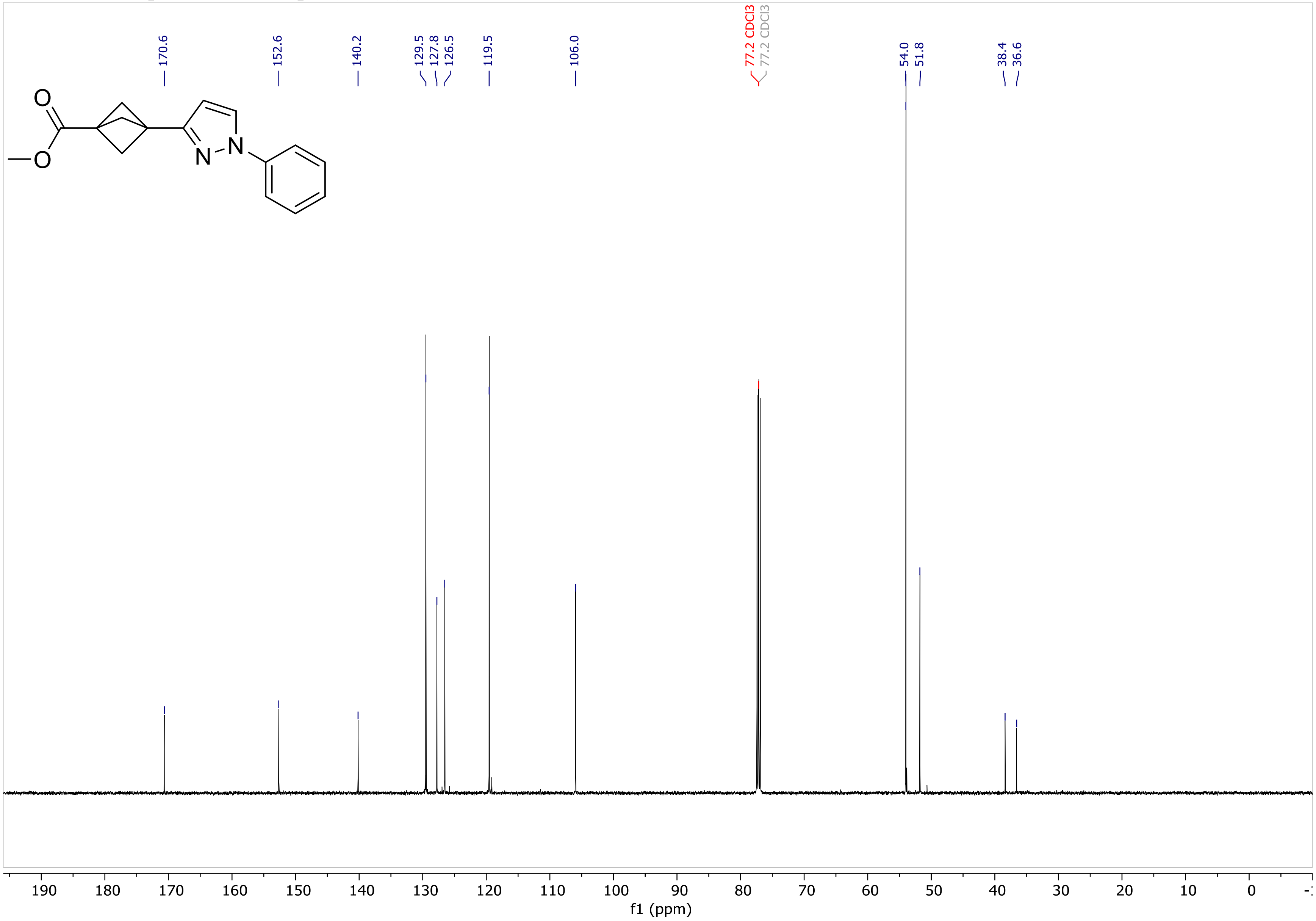

^{1}H NMR spectrum of compound 3y (600 MHz, $CDCl_3$)

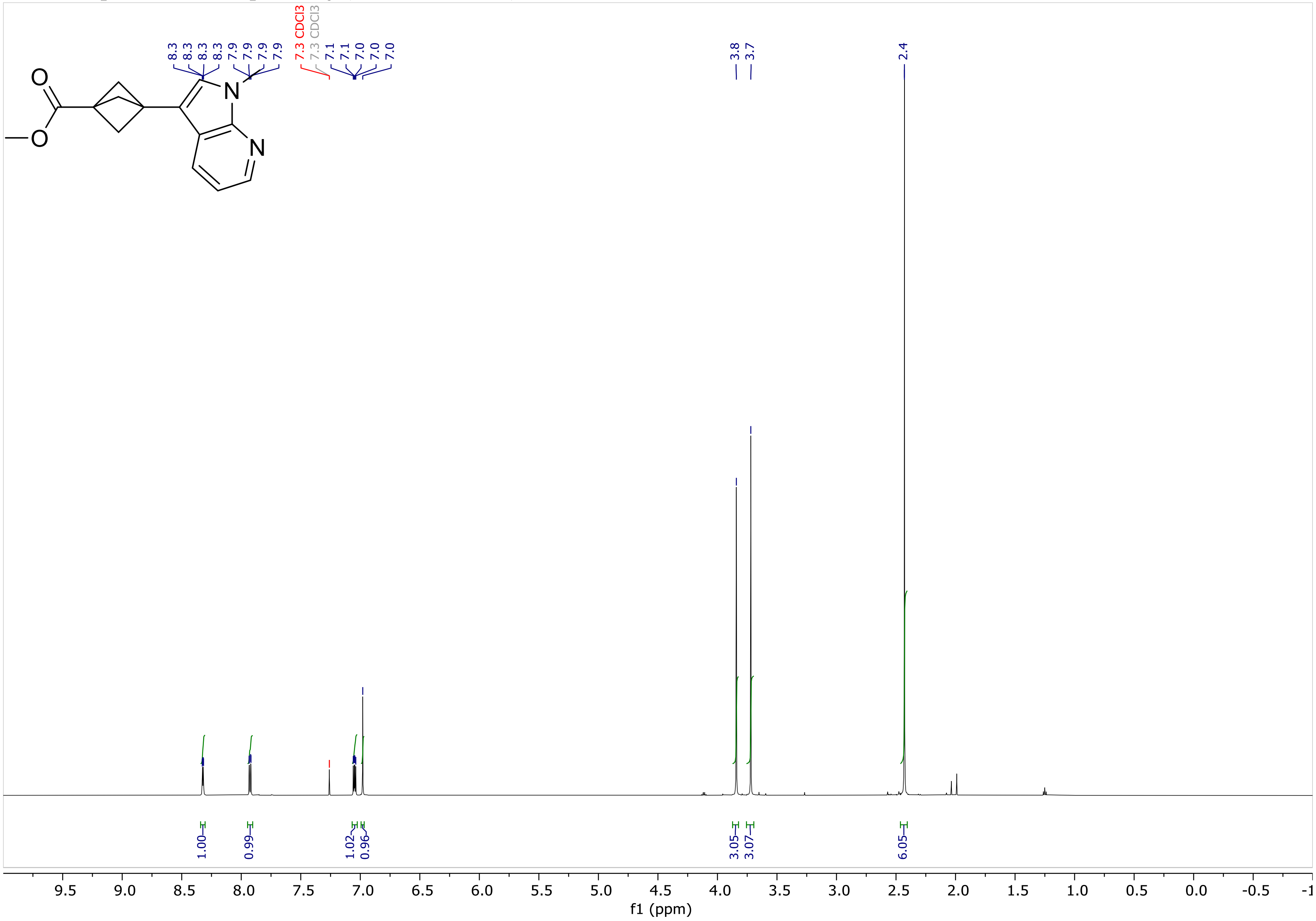

$^{13}C\{^1H\}$ NMR spectrum of compound 3y (151 MHz, $CDCl_3$)

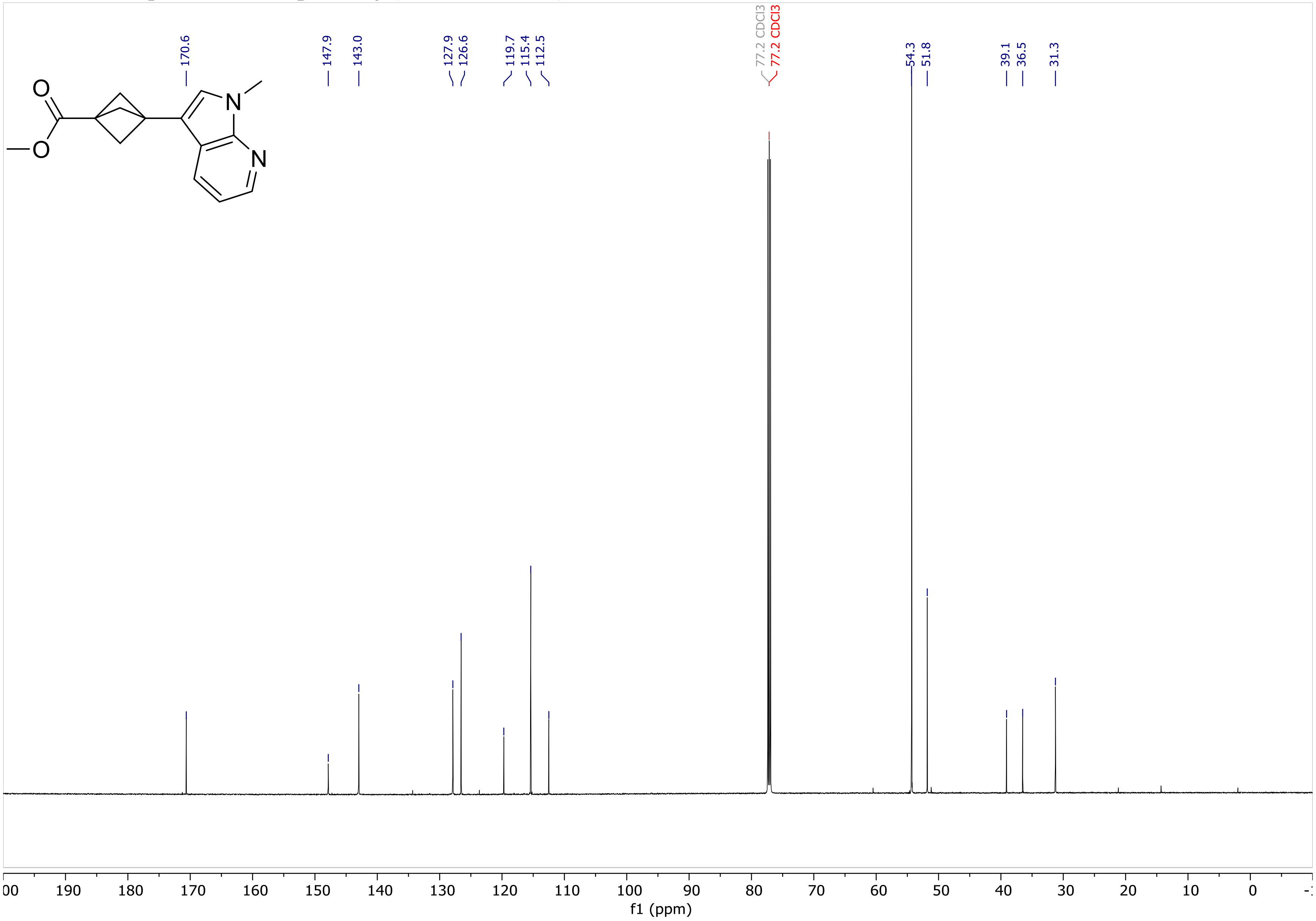

^{1}H NMR spectrum of compound 3z (600 MHz, $CDCl_3$)

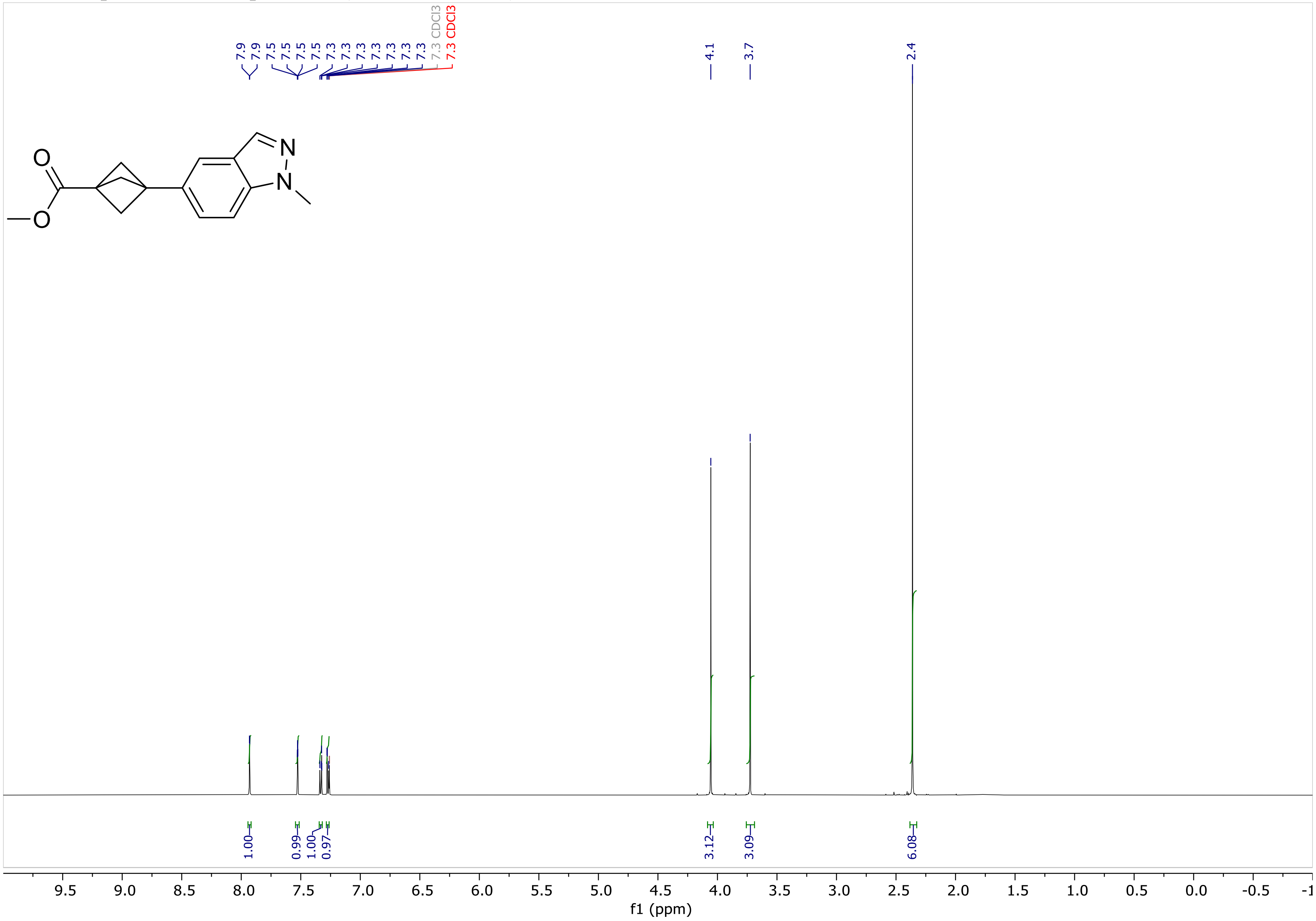

$^{13}C\{^1H\}$ NMR spectrum of compound 3z (151 MHz, $CDCl_3$)

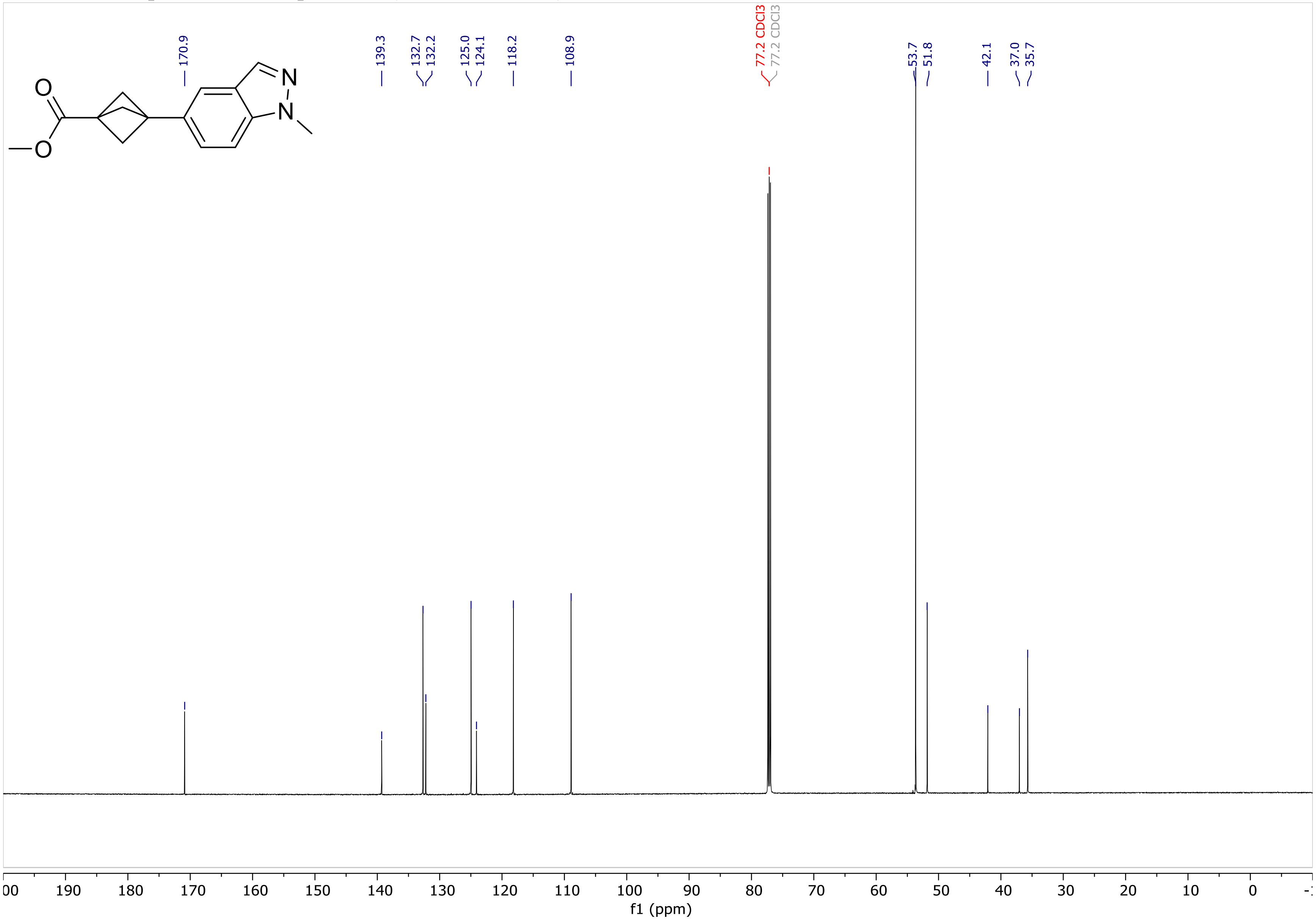

^{1}H NMR spectrum of compound 3aa (600 MHz, $CDCl_3$)

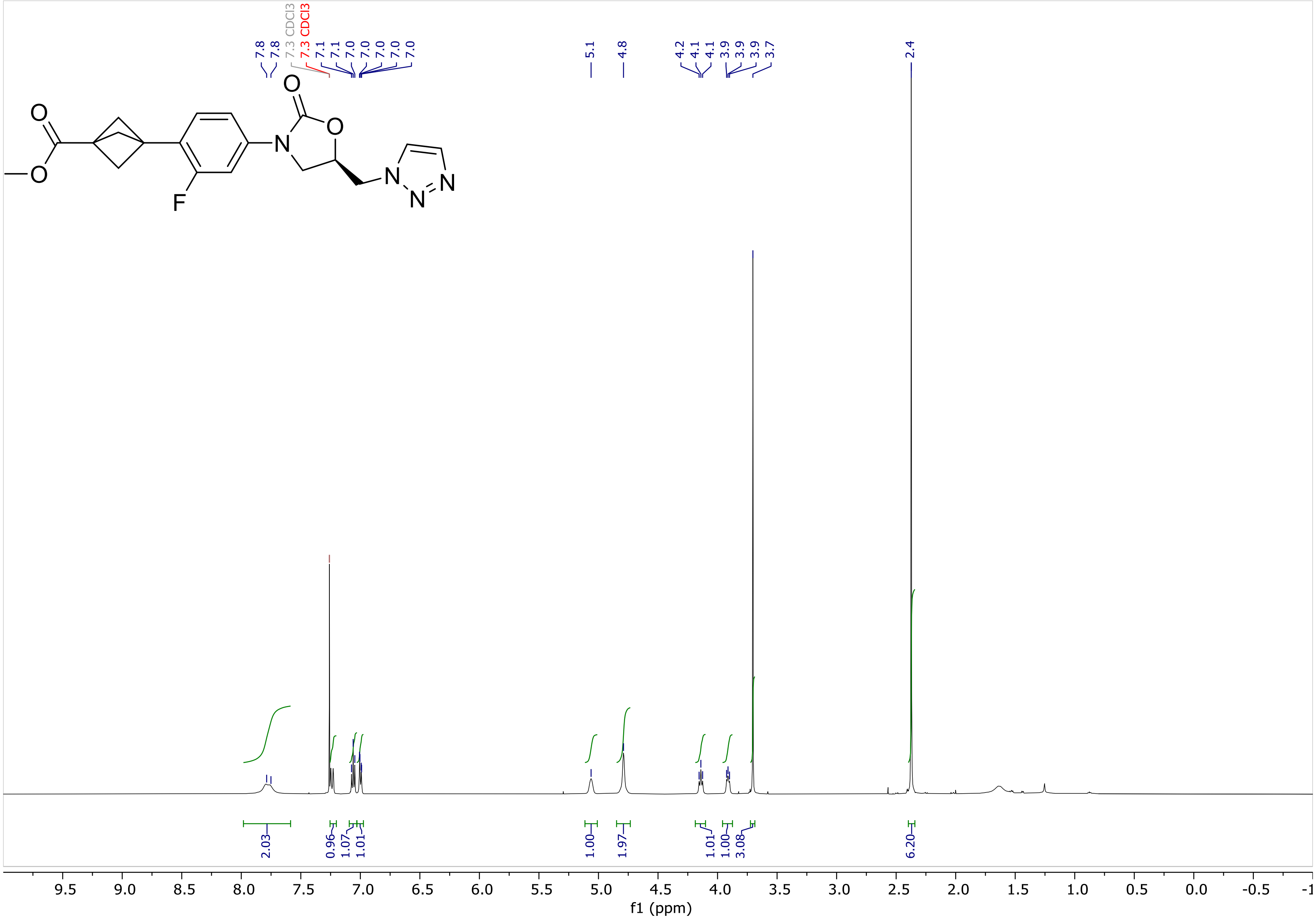

$^{13}C\{^1H\}$ **NMR spectrum of compound 3aa (151 MHz, $CDCl_3$)**

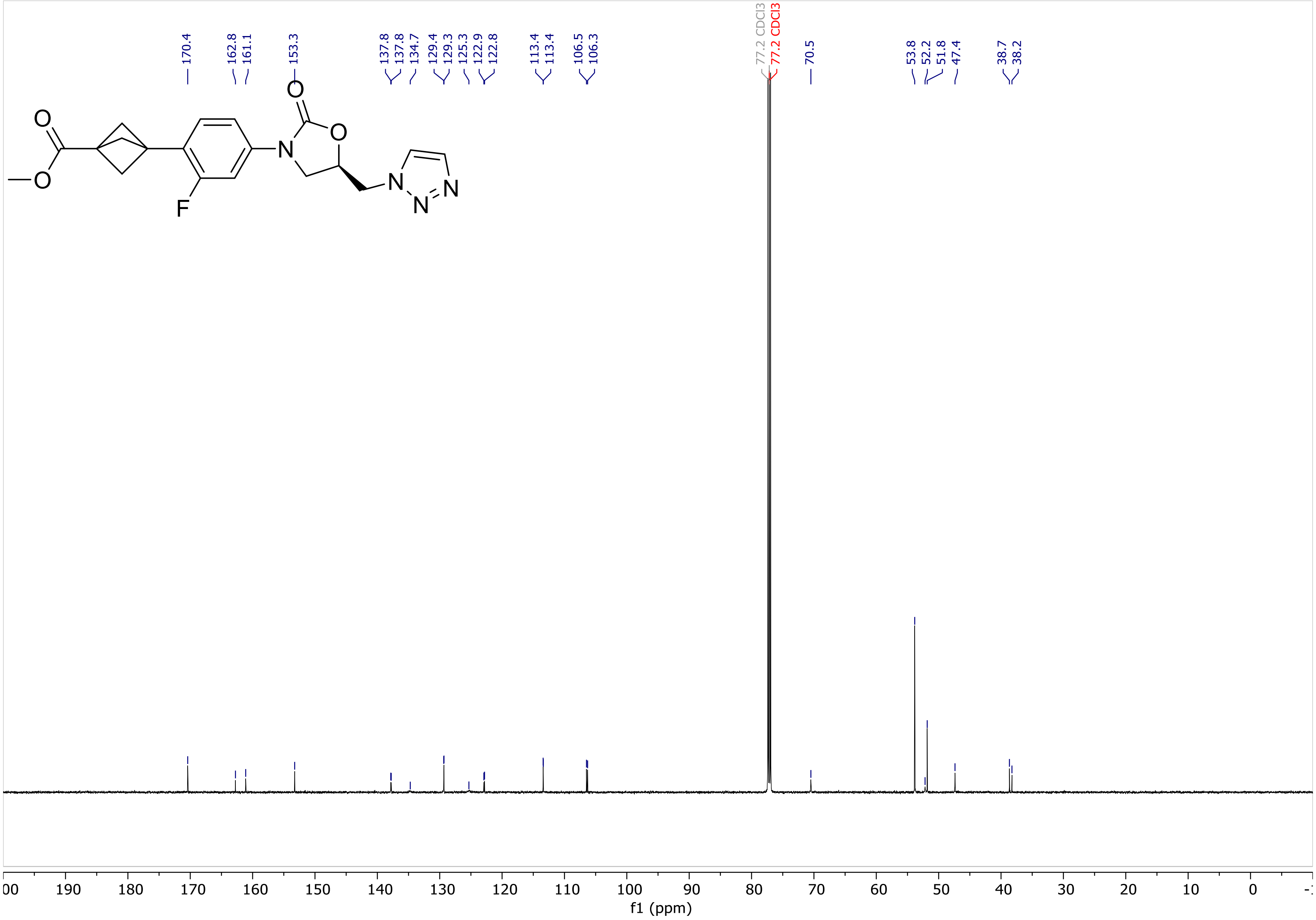

$^{19}F\{^{1}H\}$ NMR spectrum of compound 3aa (471 MHz, $CDCl_3$)

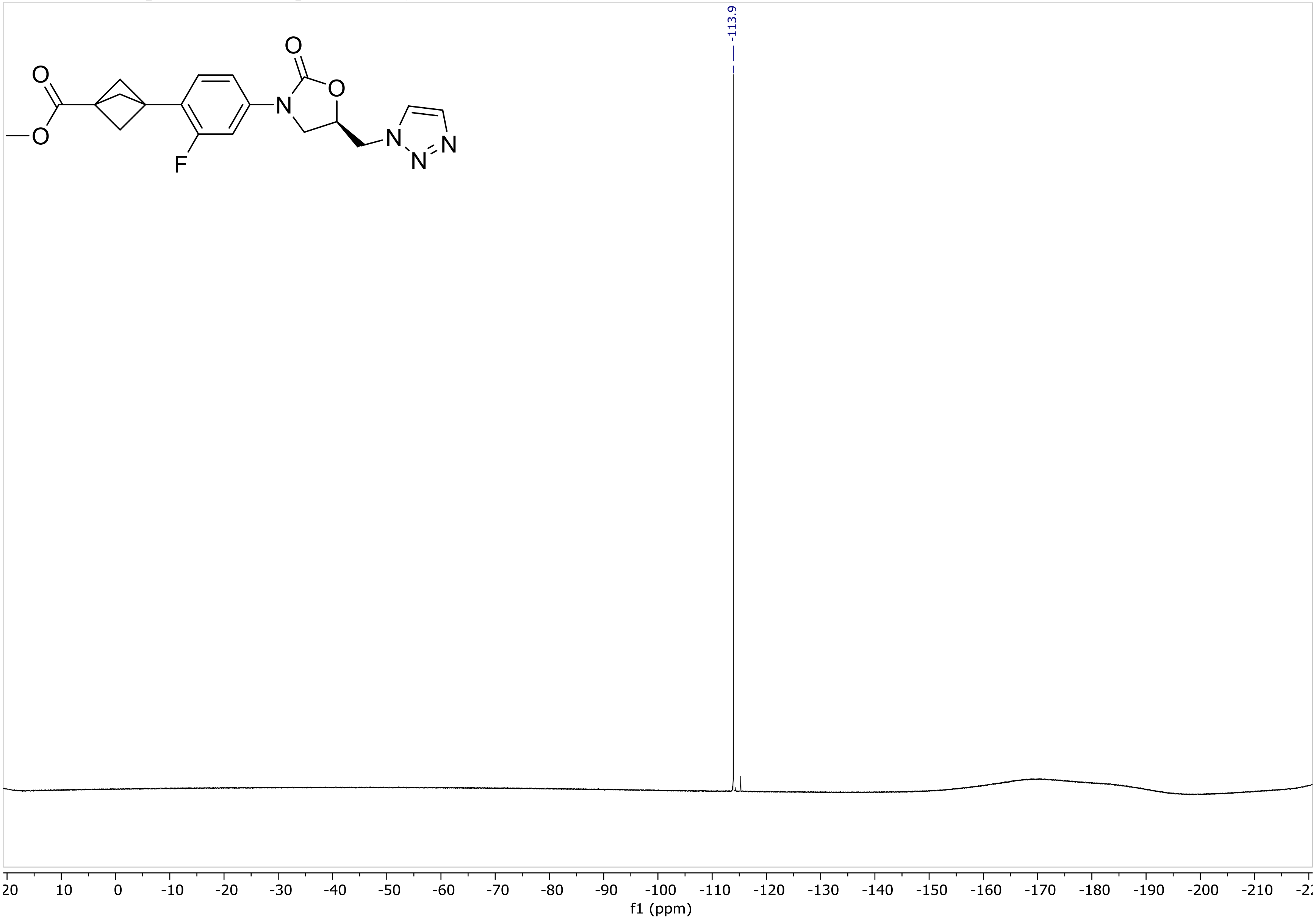

^{1}H NMR spectrum of compound 3ab (600 MHz, $CDCl_3$)

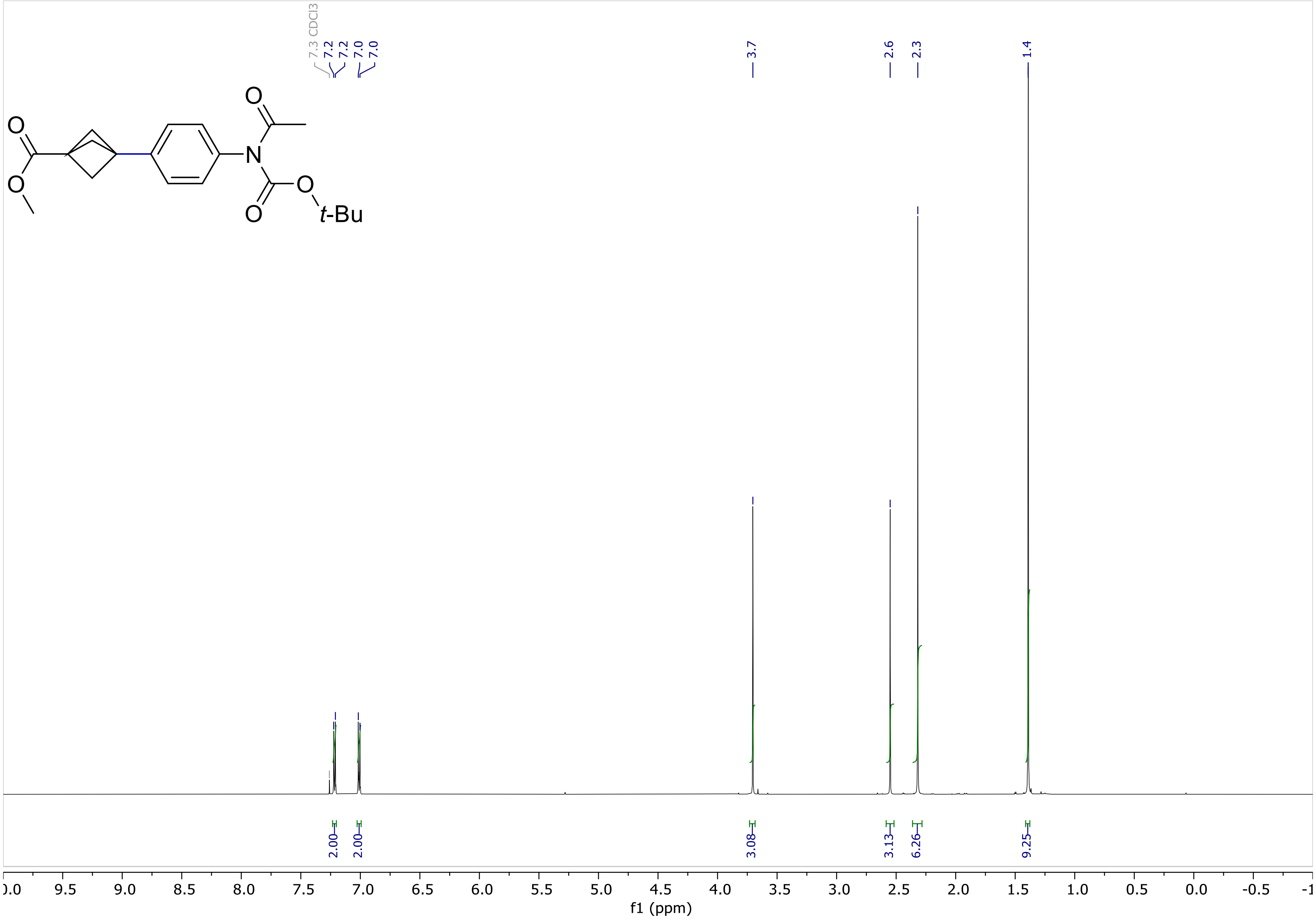

$^{13}C\{^{1}H\}$ NMR spectrum of compound 3ab (151 MHz, $CDCl_3$)

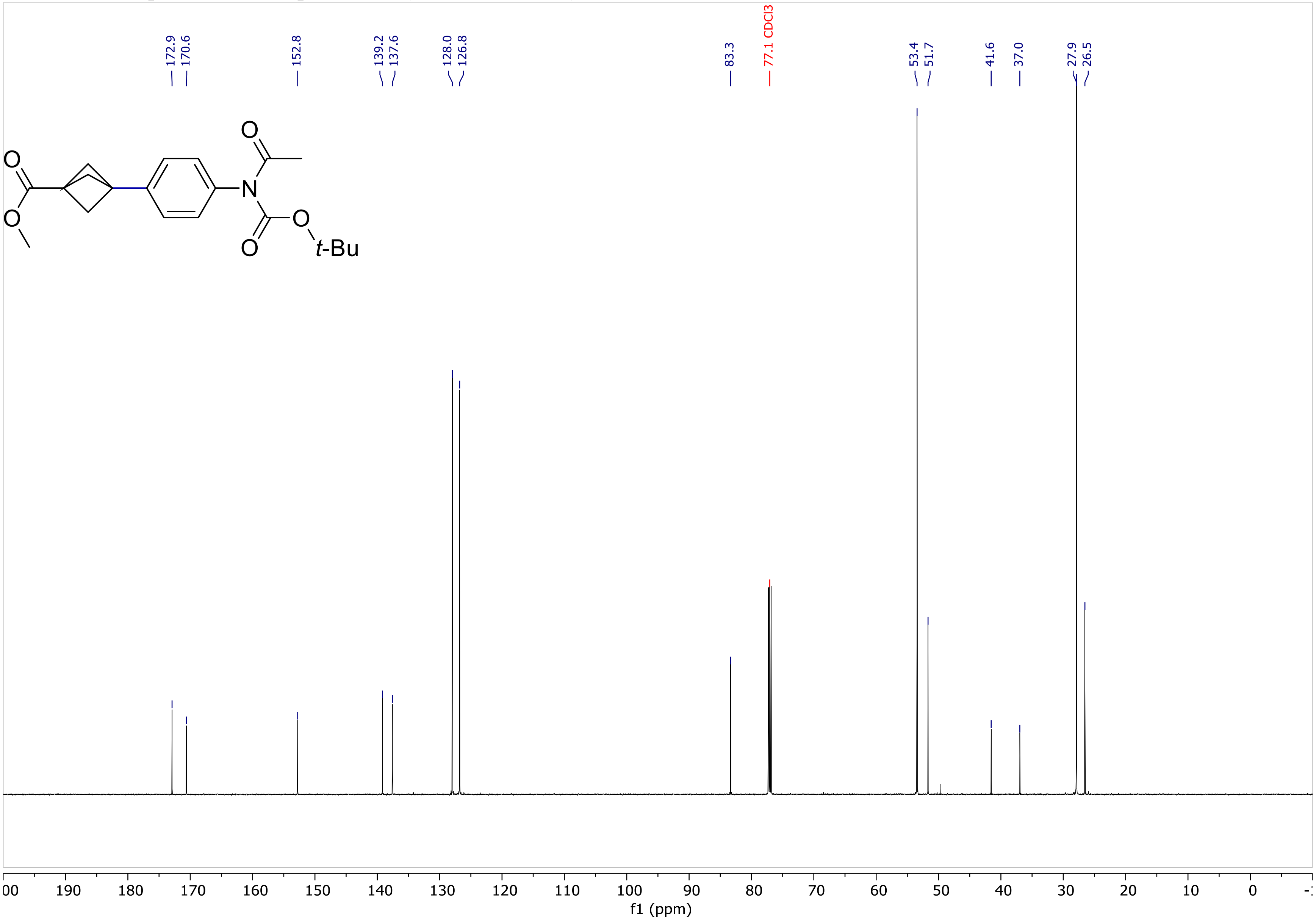

^{1}H NMR spectrum of compound 3ac (600 MHz, $CDCl_3$)

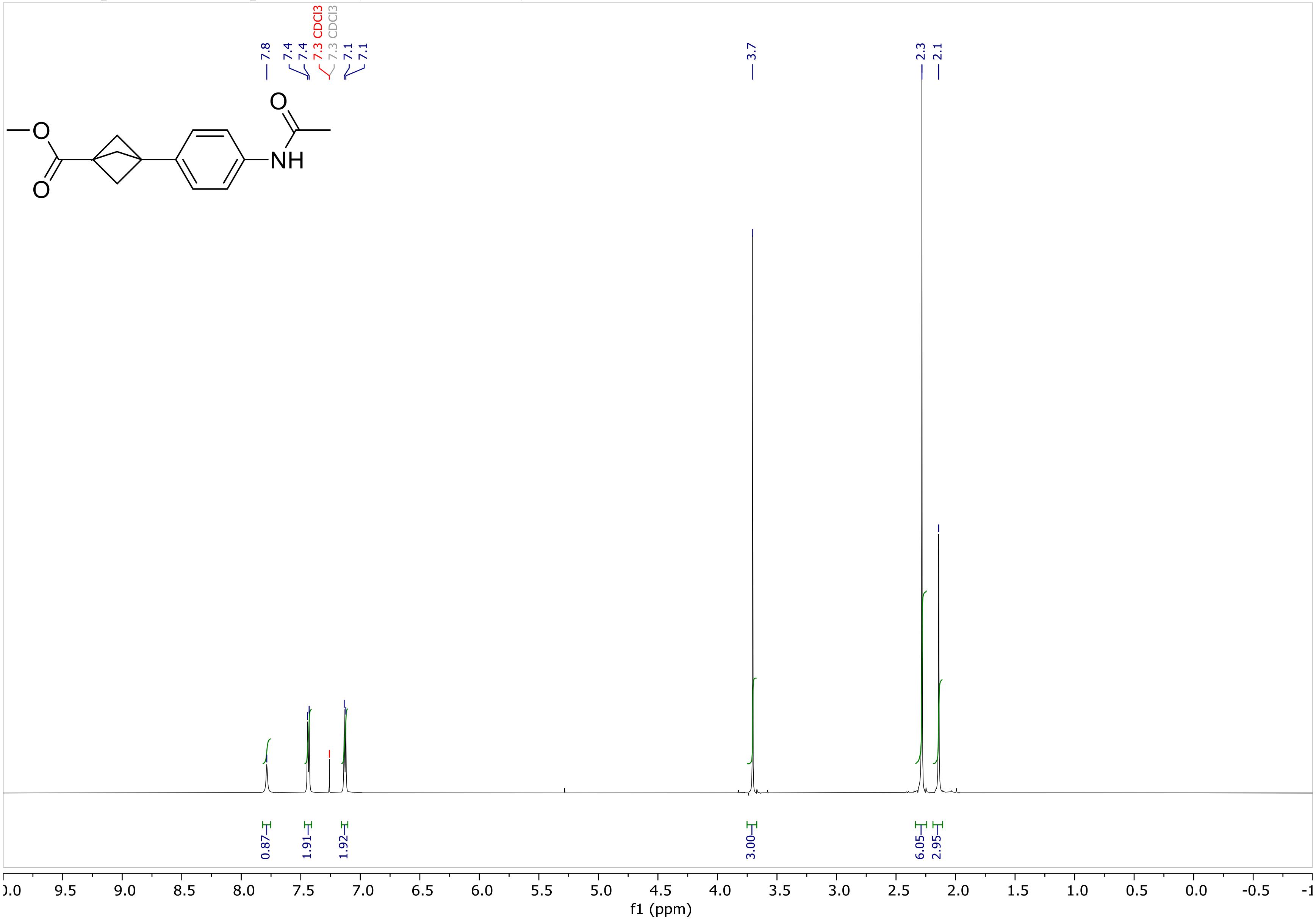

$^{13}C\{^{1}H\}$ NMR spectrum of compound 3ac (151 MHz, $CDCl_3$)

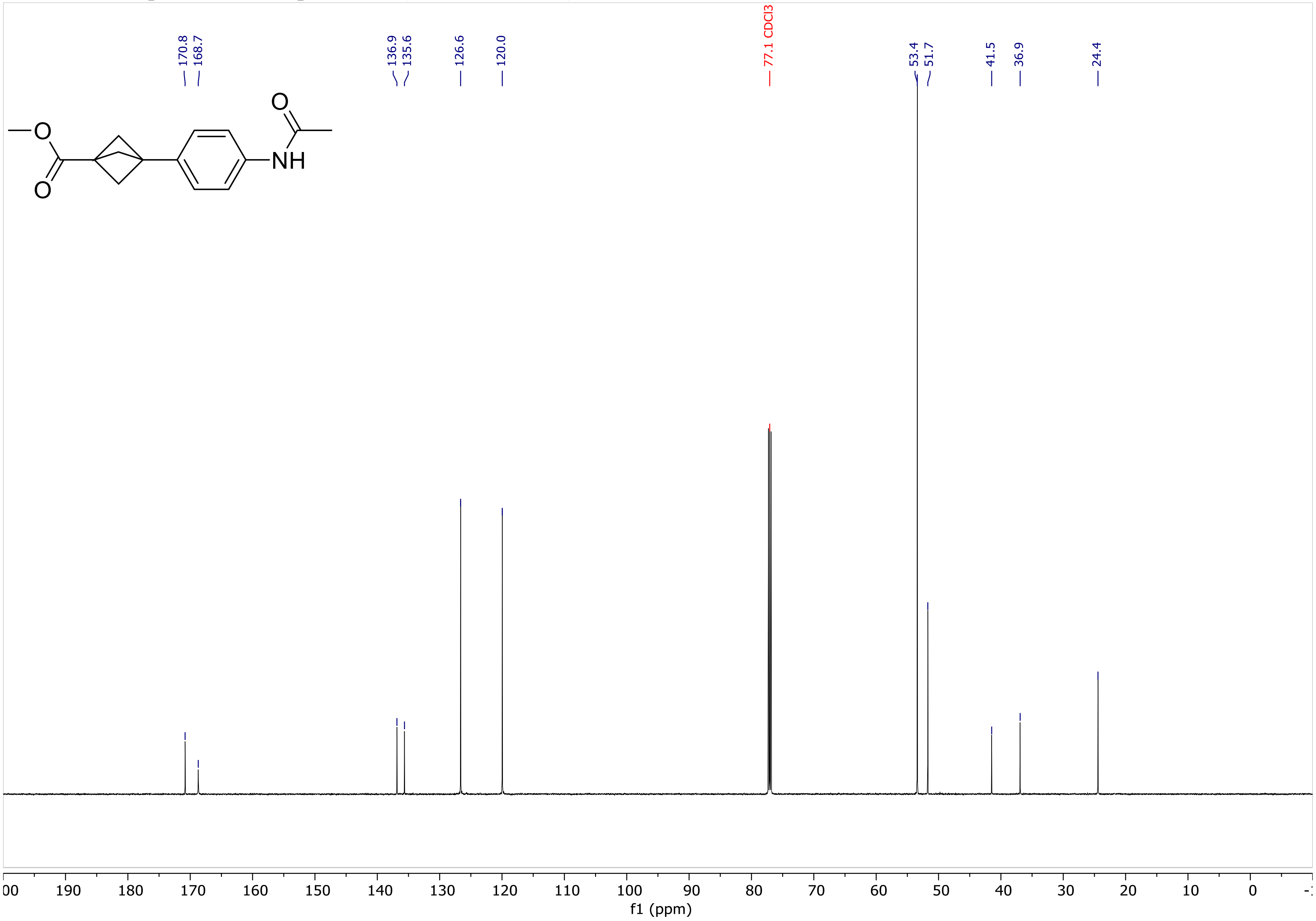

^{1}H NMR spectrum of compound 3ad (500 MHz, $CDCl_3$)

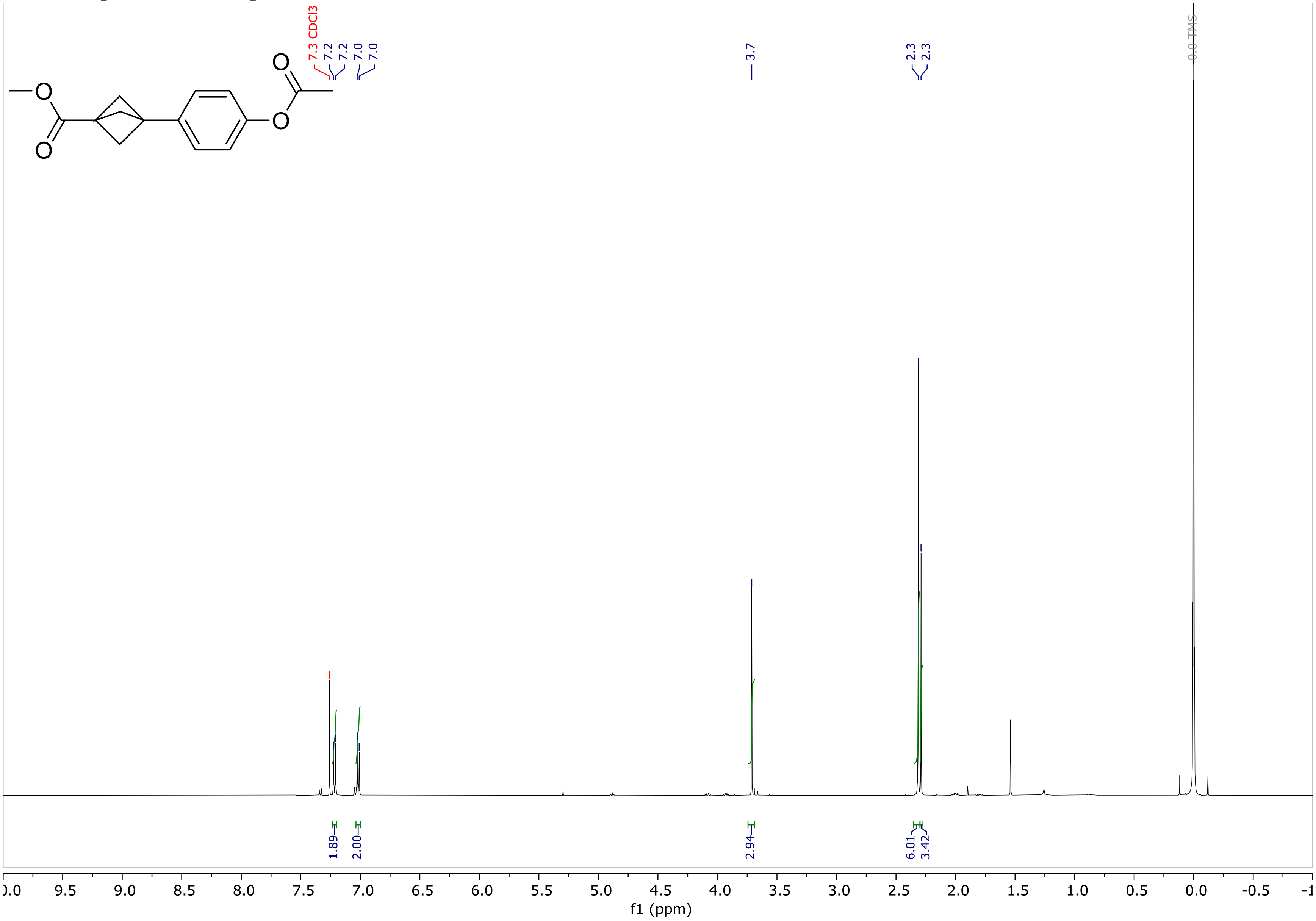

$^{13}C\{^{1}H\}$ **NMR spectrum of compound 3ad (126 MHz, $CDCl_3$)**

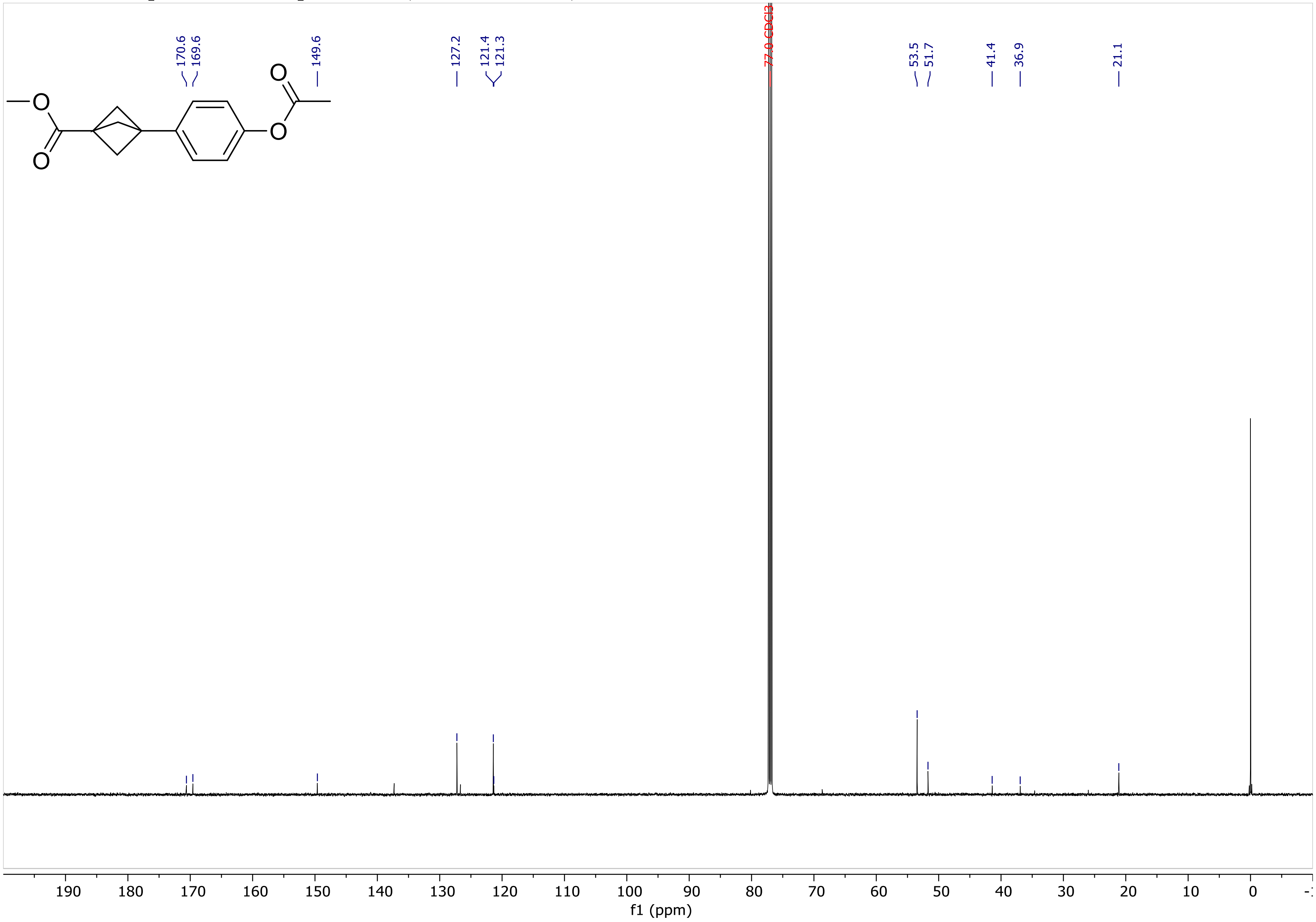

^{1}H NMR spectrum of compound 3ae (600 MHz, CDCl3)

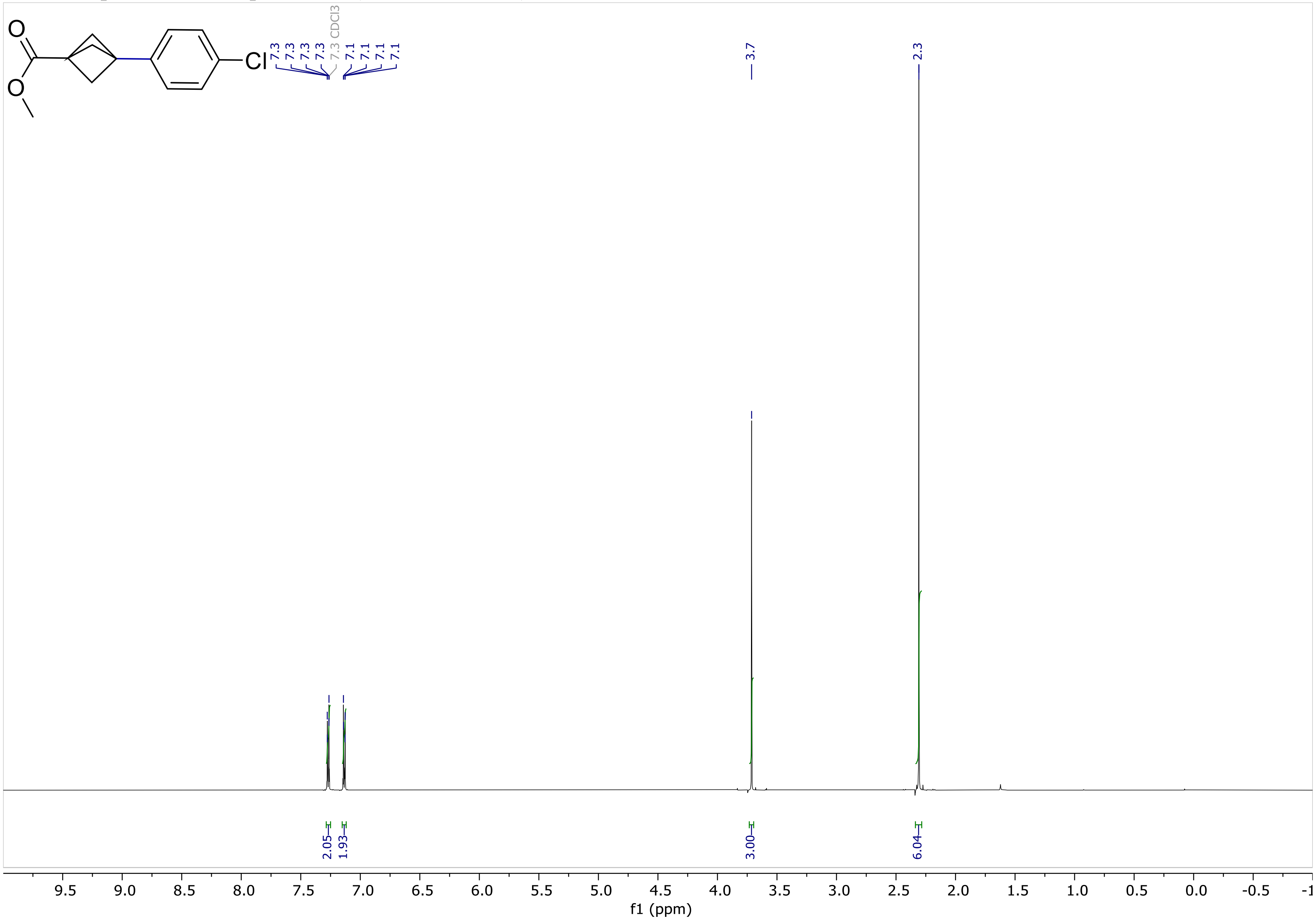

$^{13}C\{^1H\}$ NMR spectrum of compound 3ae (151 MHz, $CDCl_3$)

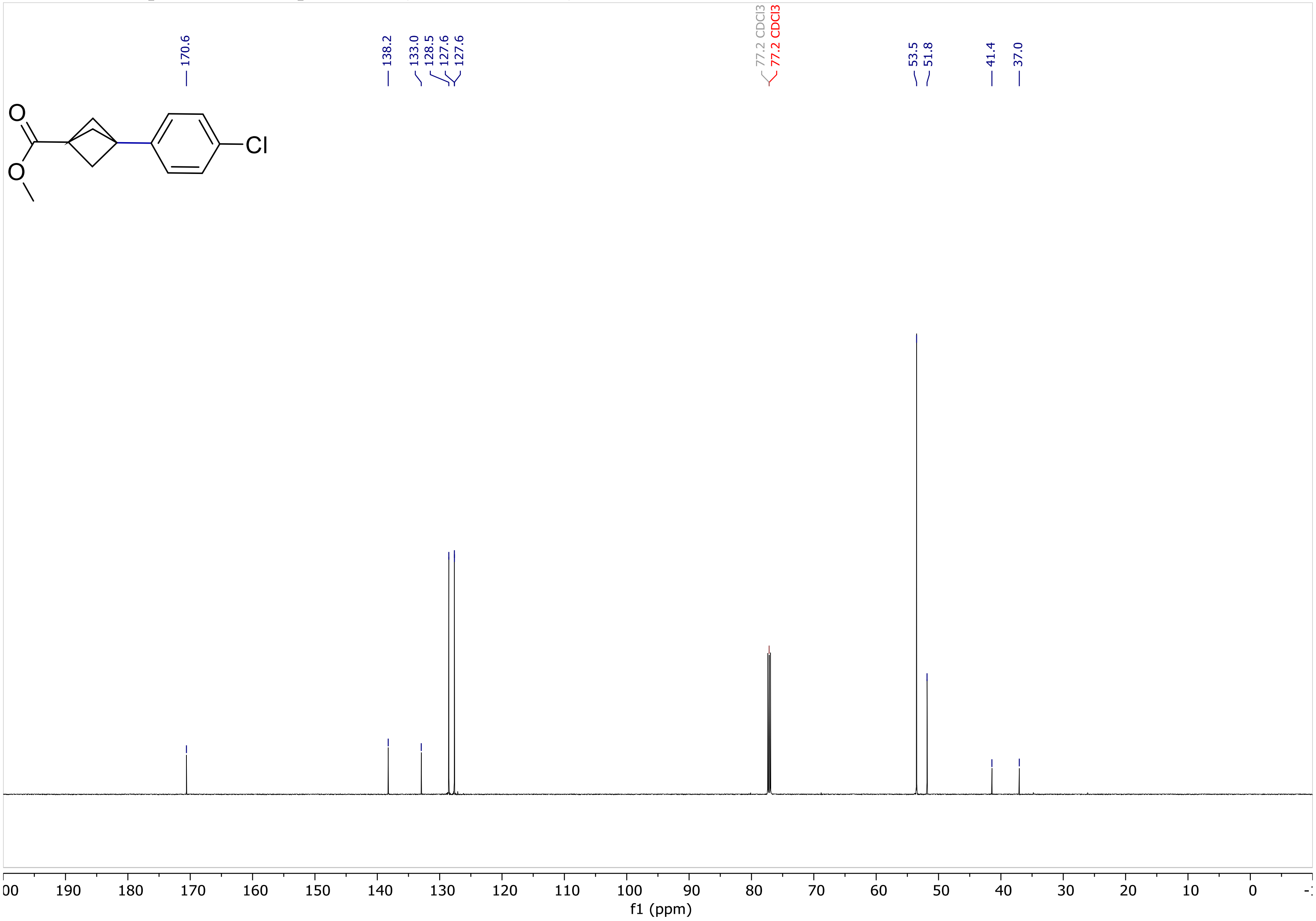

^{1}H NMR spectrum of compound 3af (500 MHz, CDCl$_3$)

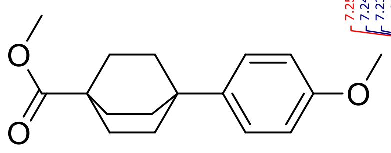

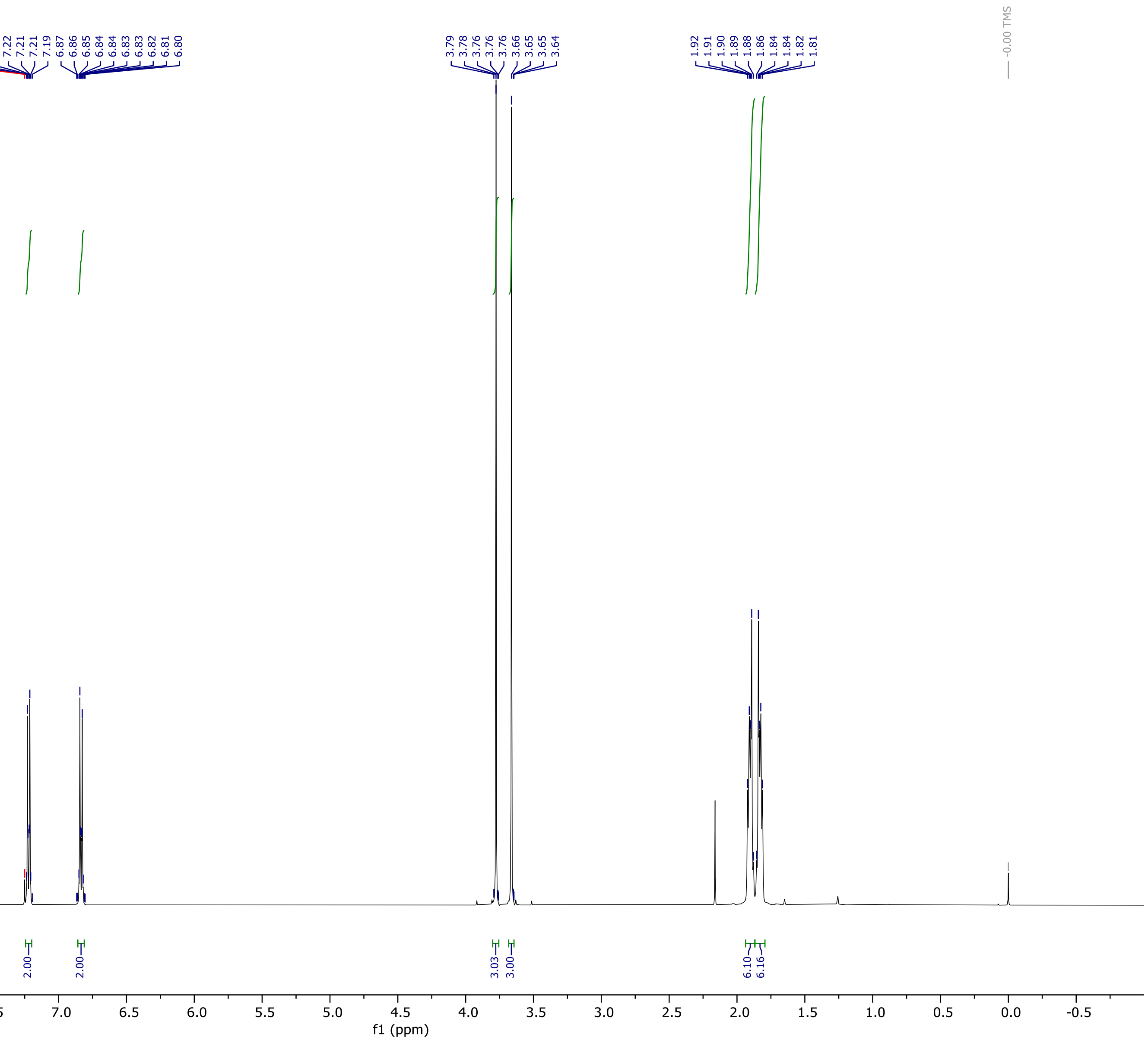

$^{13}C\{^{1}H\}$ NMR spectrum of compound 3af (126 MHz, $CDCl_3$)

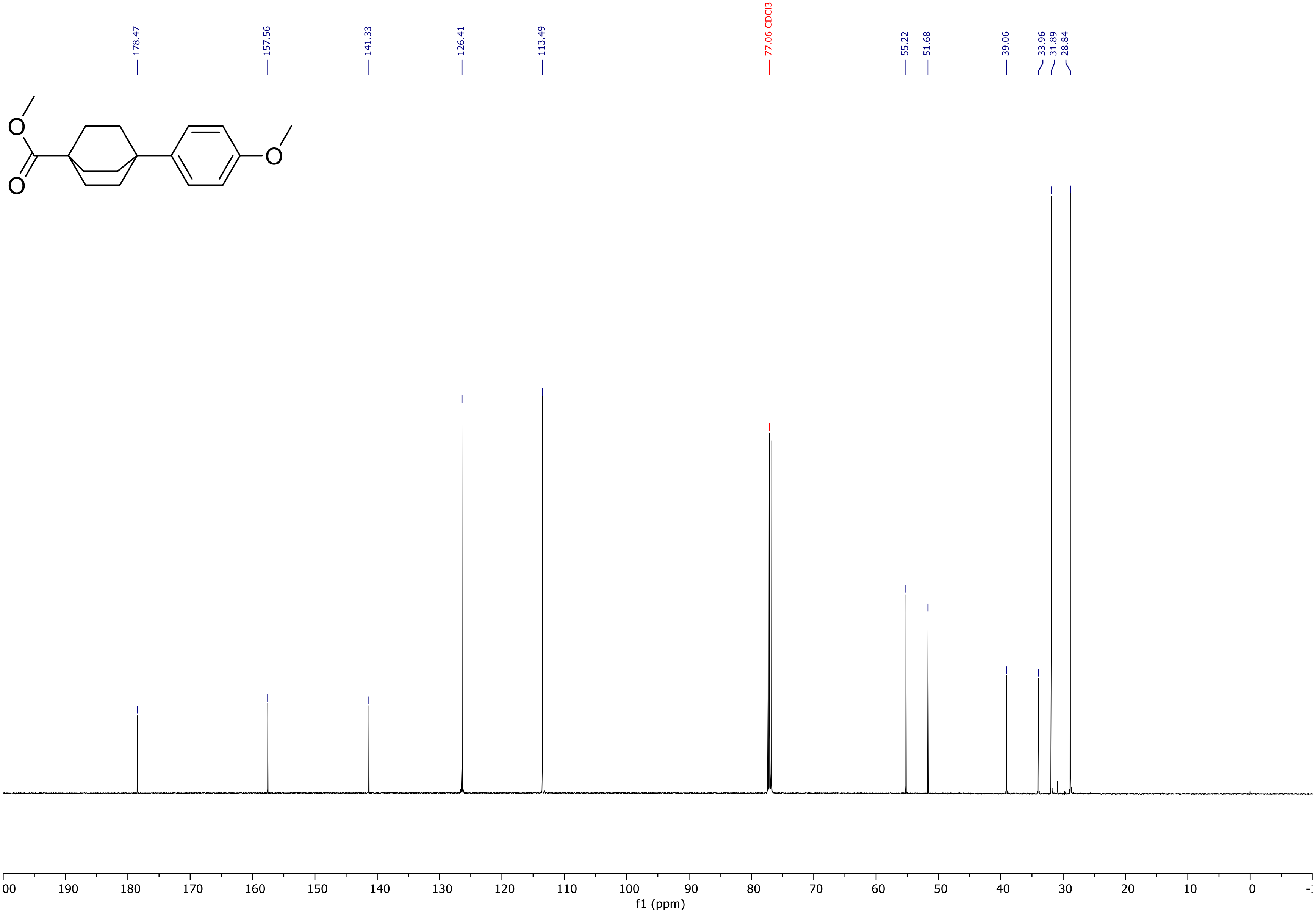

^{1}H NMR spectrum of compound 3ag (600 MHz, $CDCl_3$)

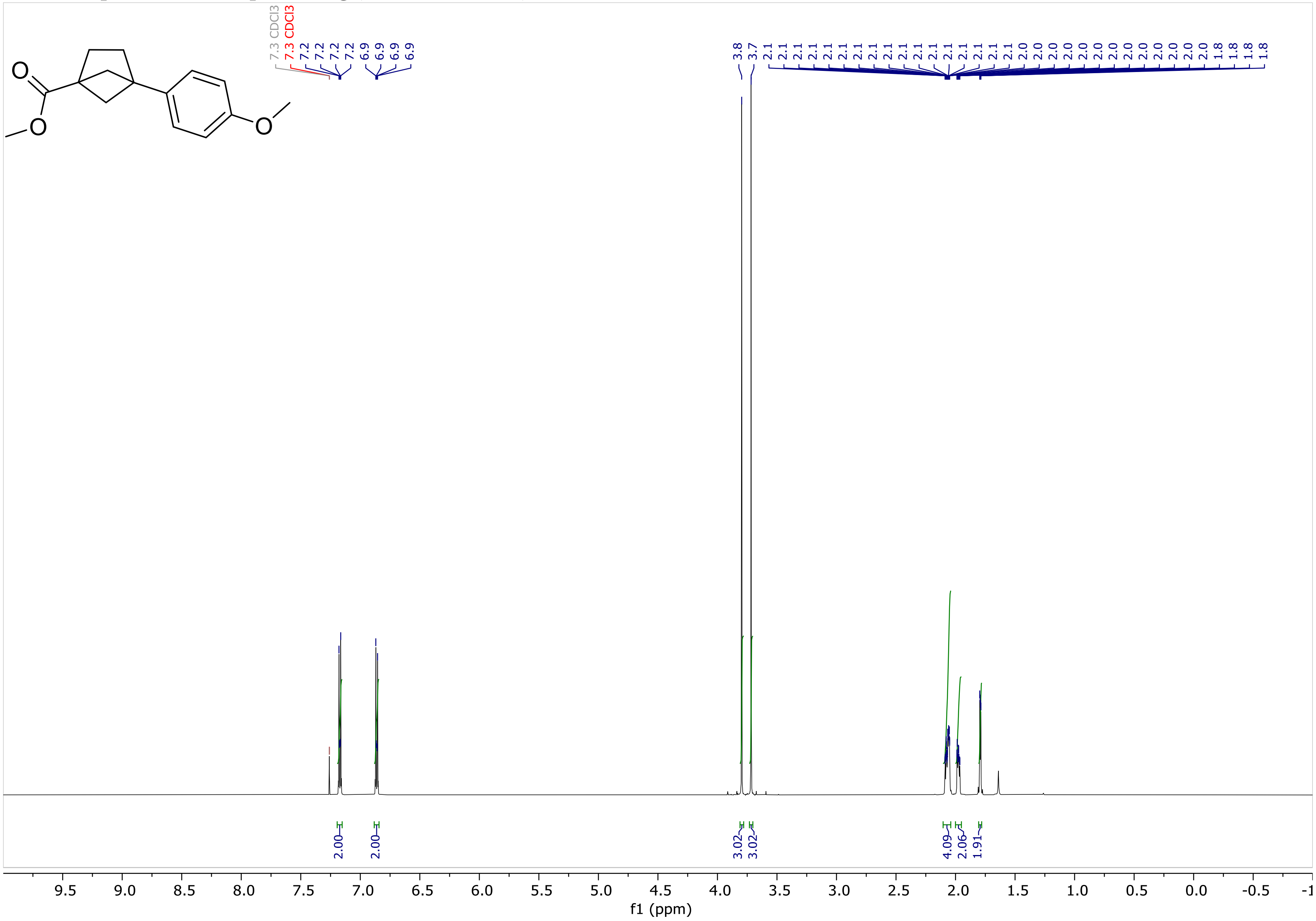

$^{13}C\{^{1}H\}$ NMR spectrum of compound 3ag (151 MHz, $CDCl_3$)

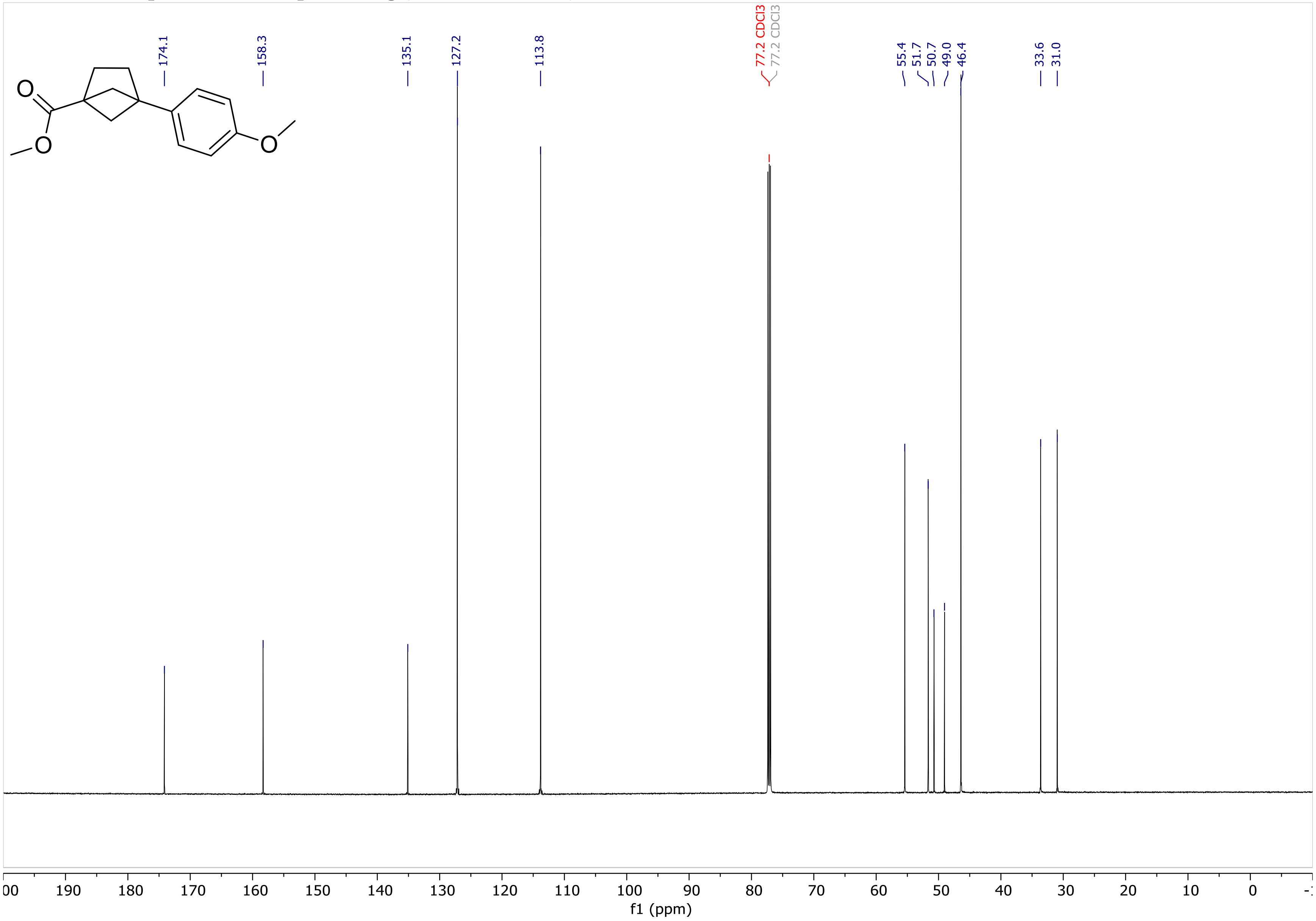

^{1}H NMR spectrum of compound 3ah (500 MHz, $CDCl_3$)

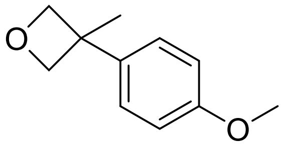

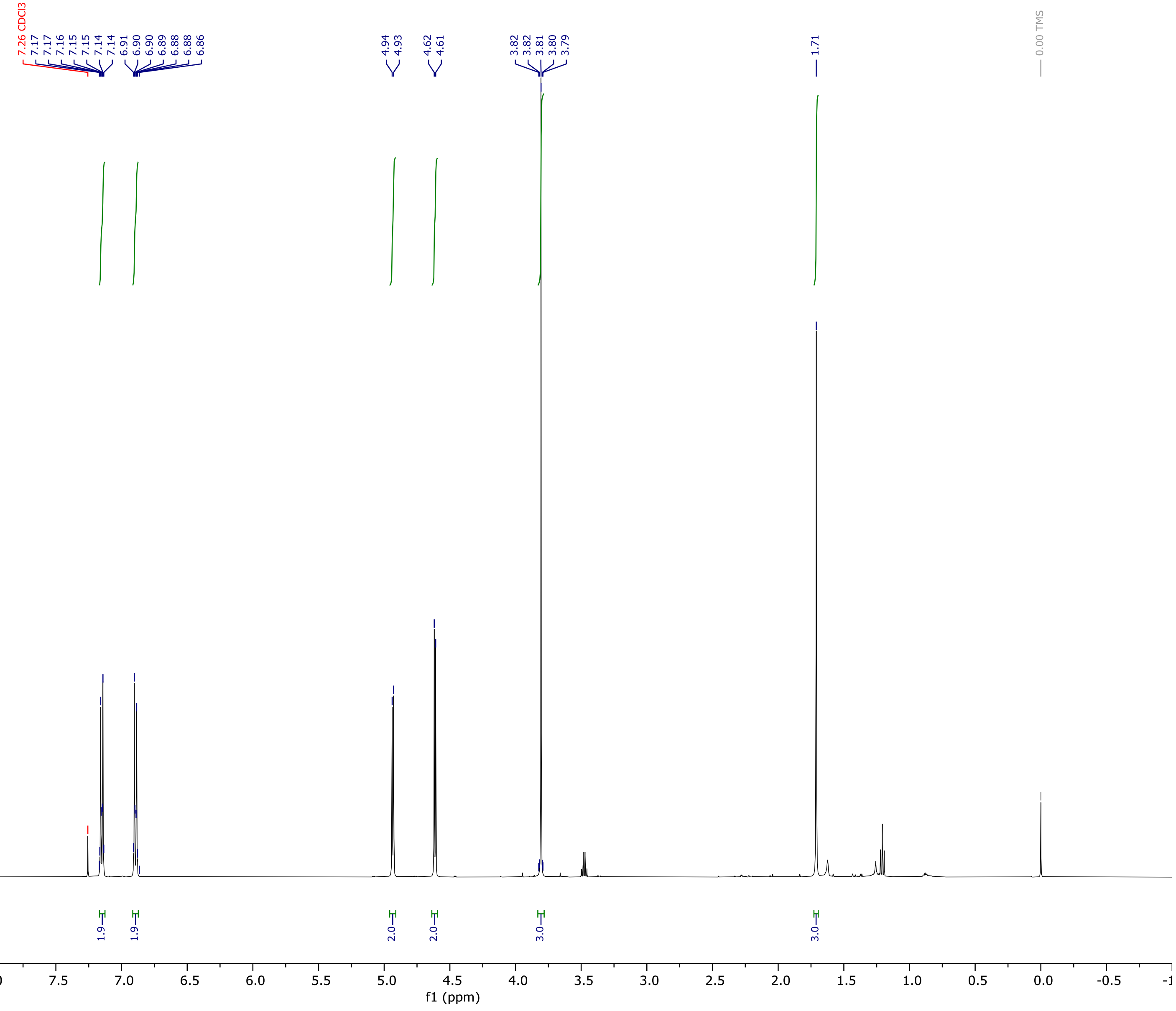

$^{13}C\{^1H\}$ **NMR spectrum of compound 3ah (126 MHz, $CDCl_3$)**

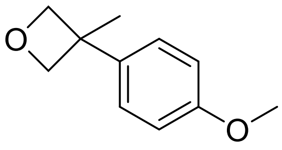

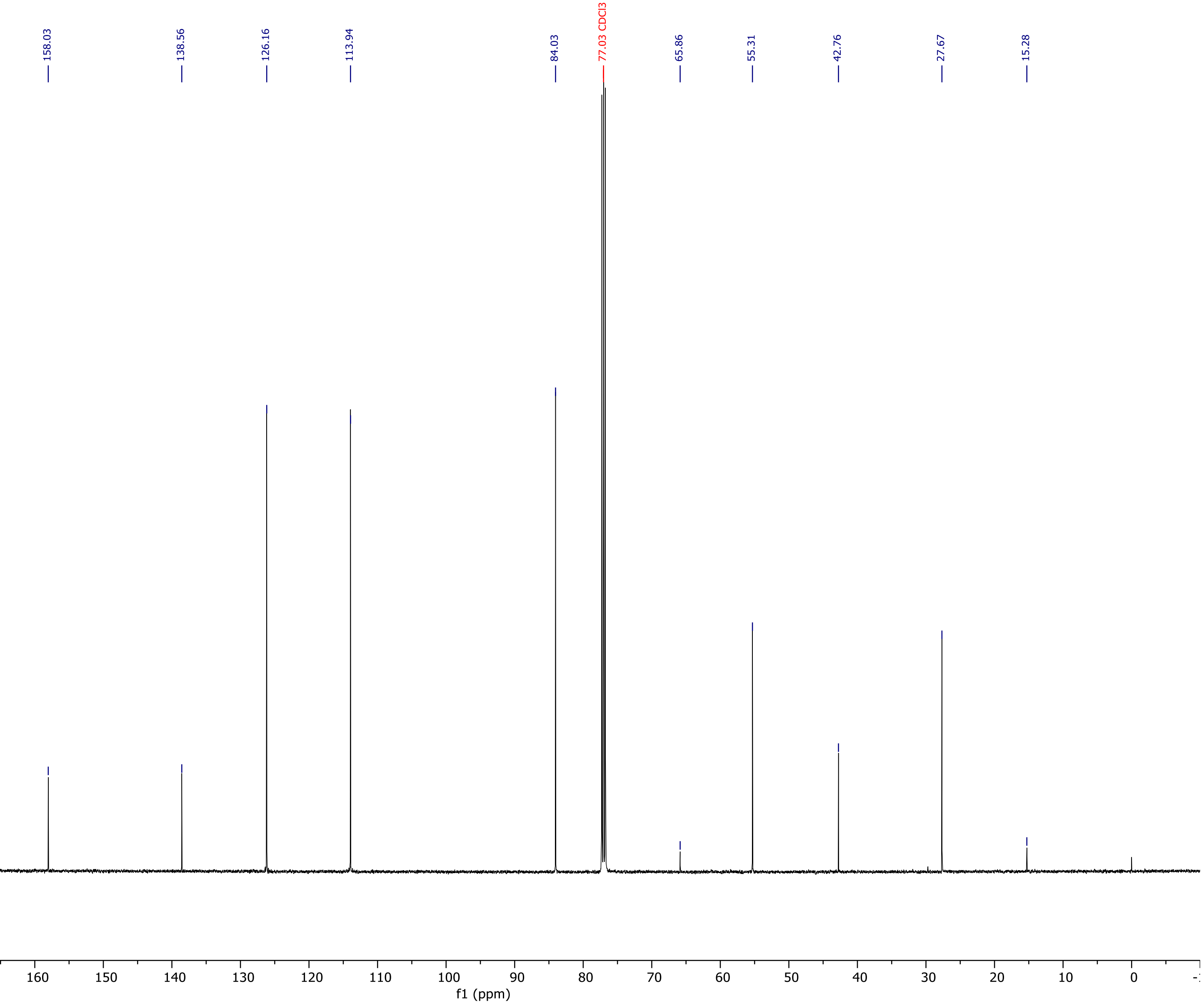

^{1}H NMR spectrum of compound 3ai (600 MHz, $CDCl_3$)

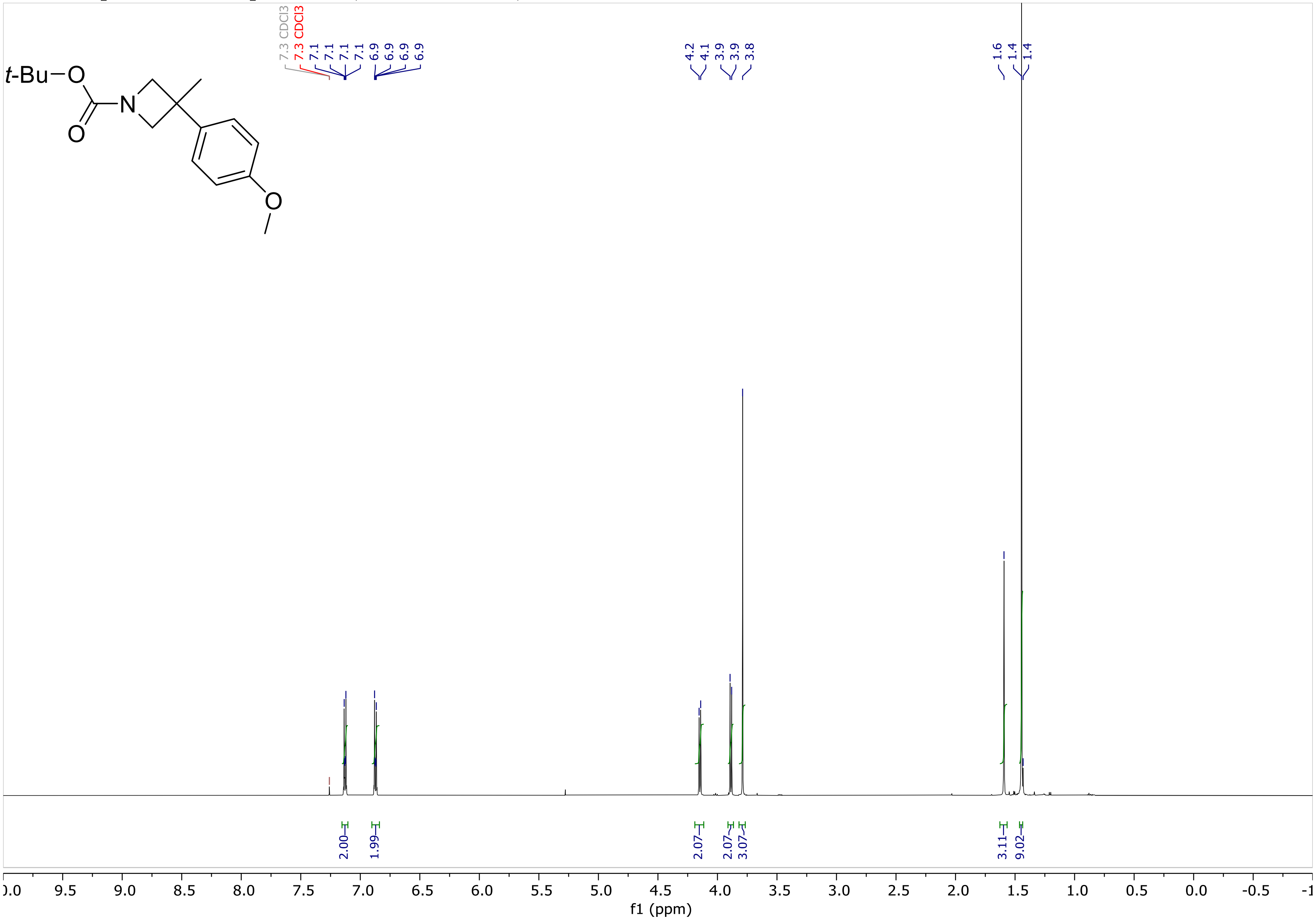

$^{13}C\{^{1}H\}$ NMR spectrum of compound 3ai (126 MHz, $CDCl_3$)

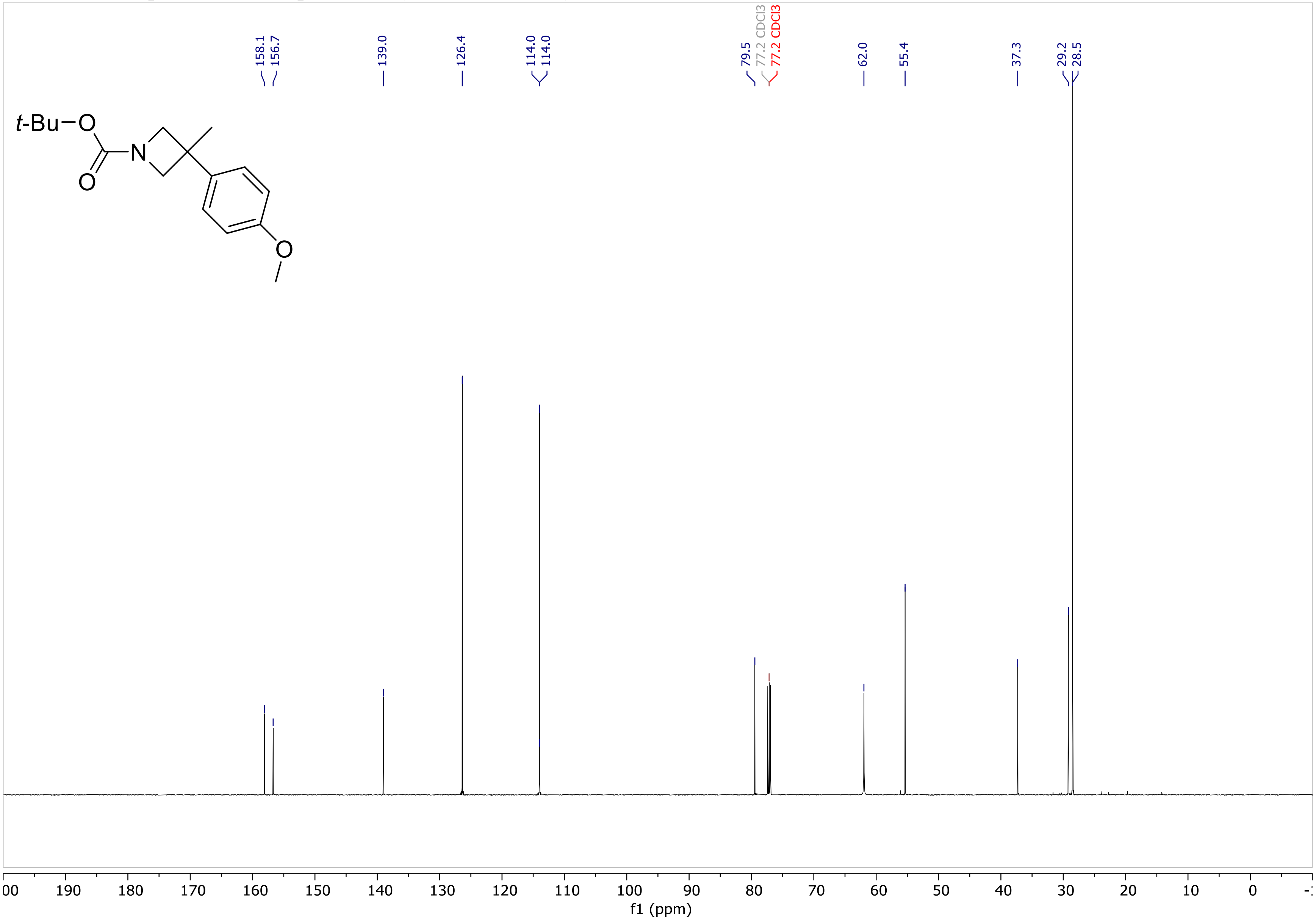

^{1}H NMR spectrum of compound 3aj (500 MHz, $CDCl_3$)

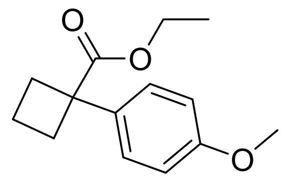

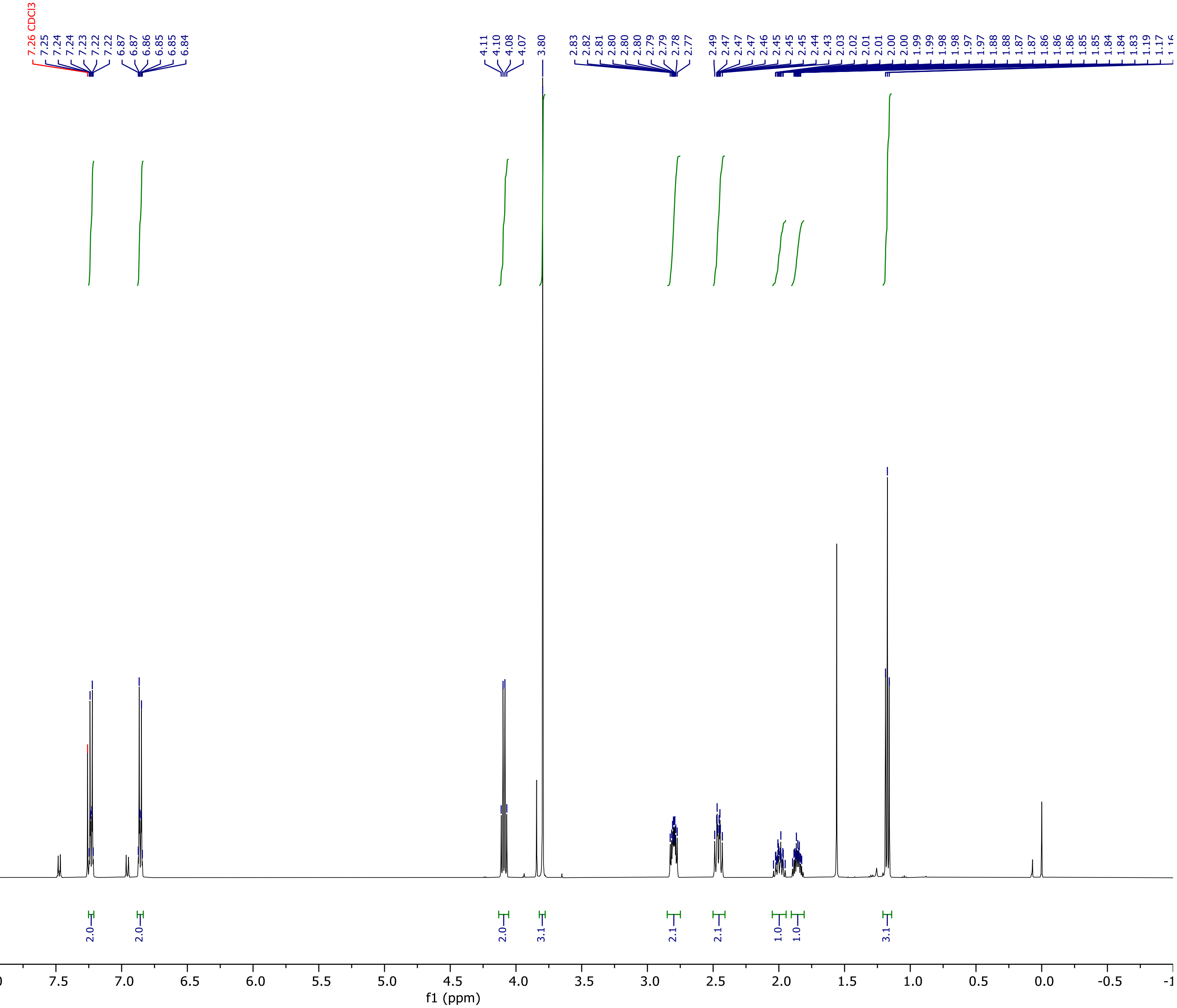

$^{13}C\{^{1}H\}$ **NMR spectrum of compound 3aj (126 MHz, $CDCl_3$)**

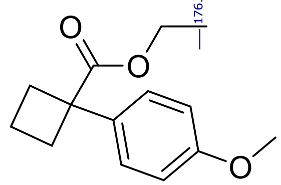

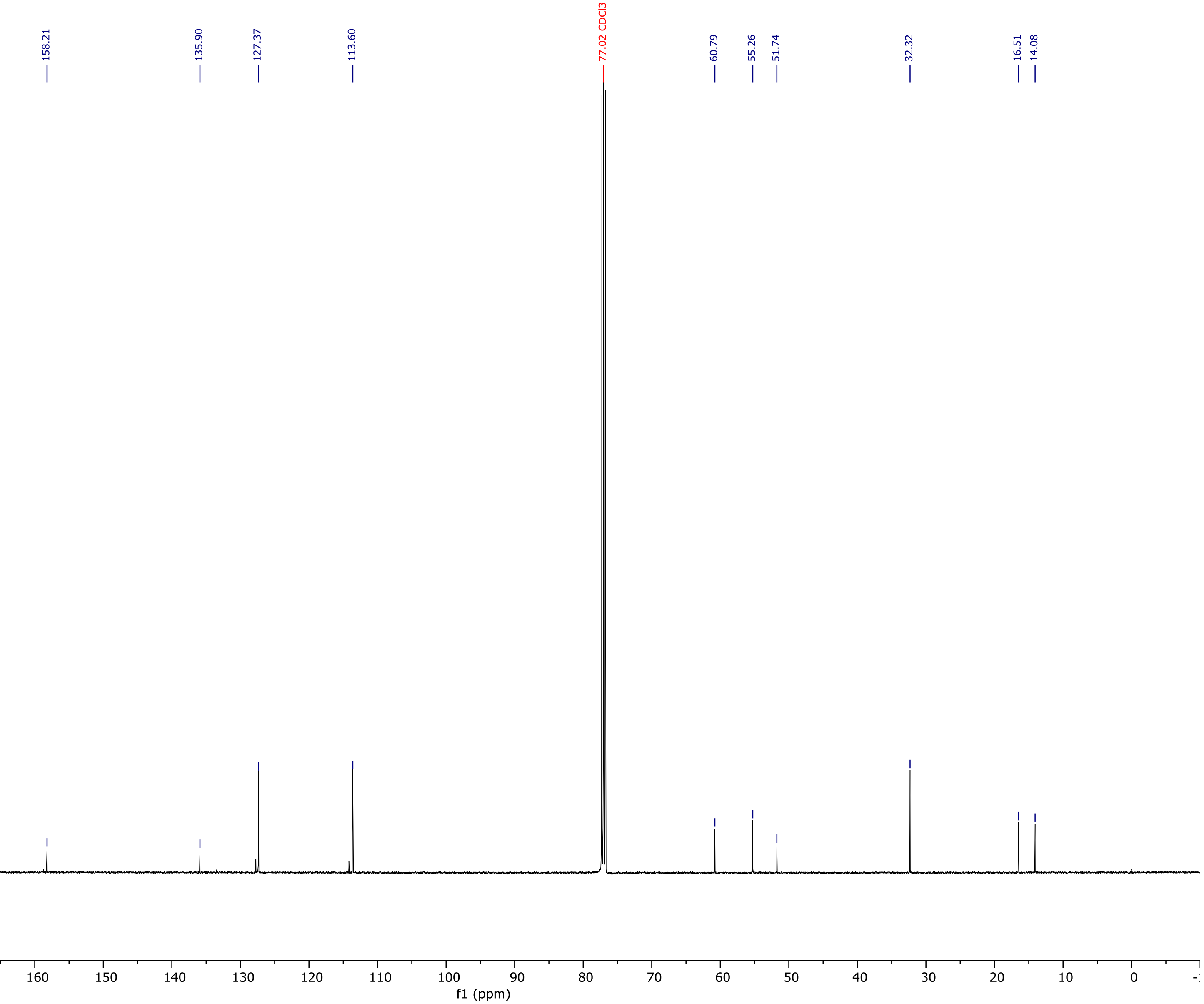

^{1}H NMR spectrum of compound 3ak (500 MHz, $CDCl_3$)

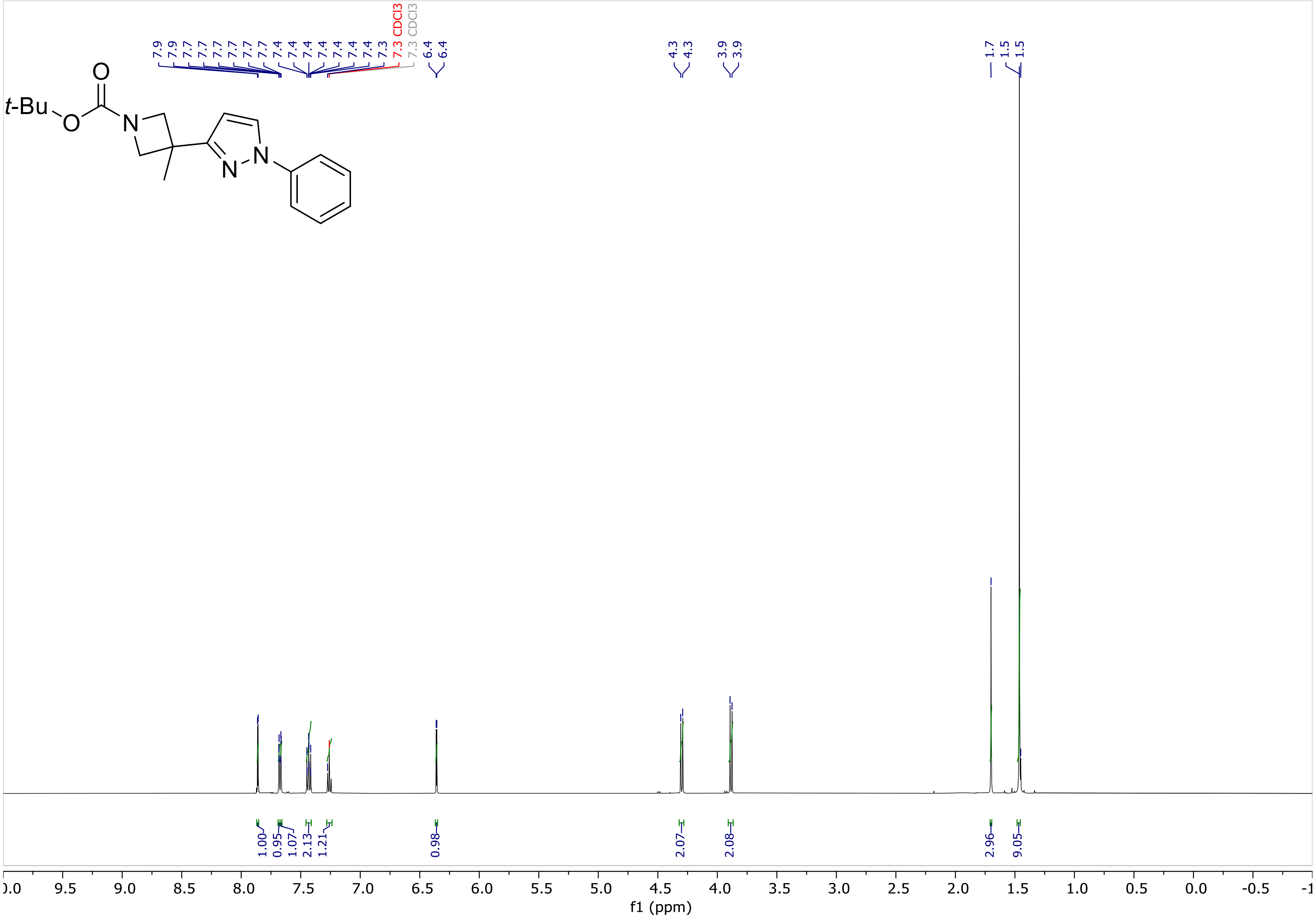

$^{13}C\{^{1}H\}$ NMR spectrum of compound 3ak (126 MHz, $CDCl_3$)

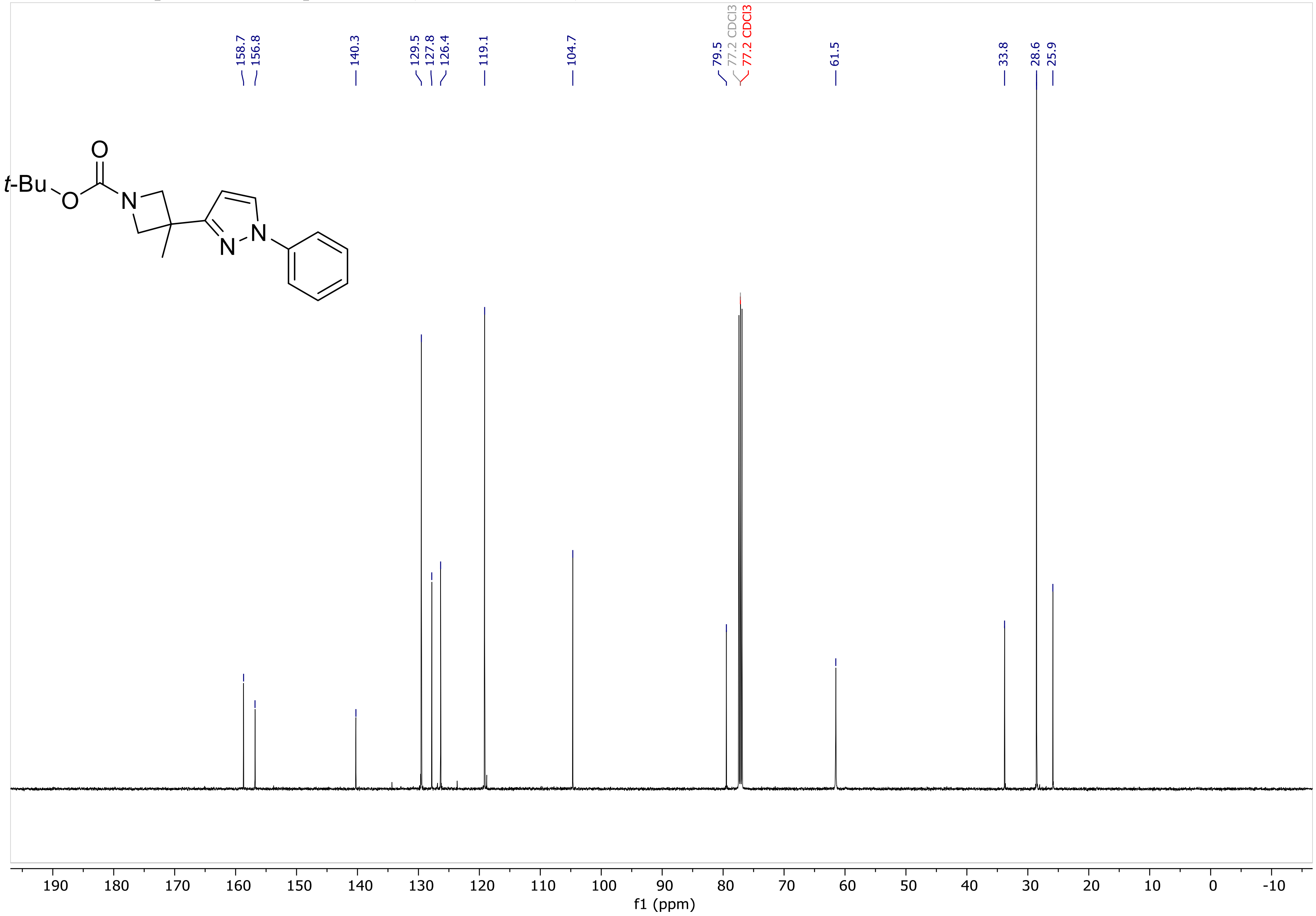

^{1}H NMR spectrum of compound 3al (500 MHz, $CDCl_3$)

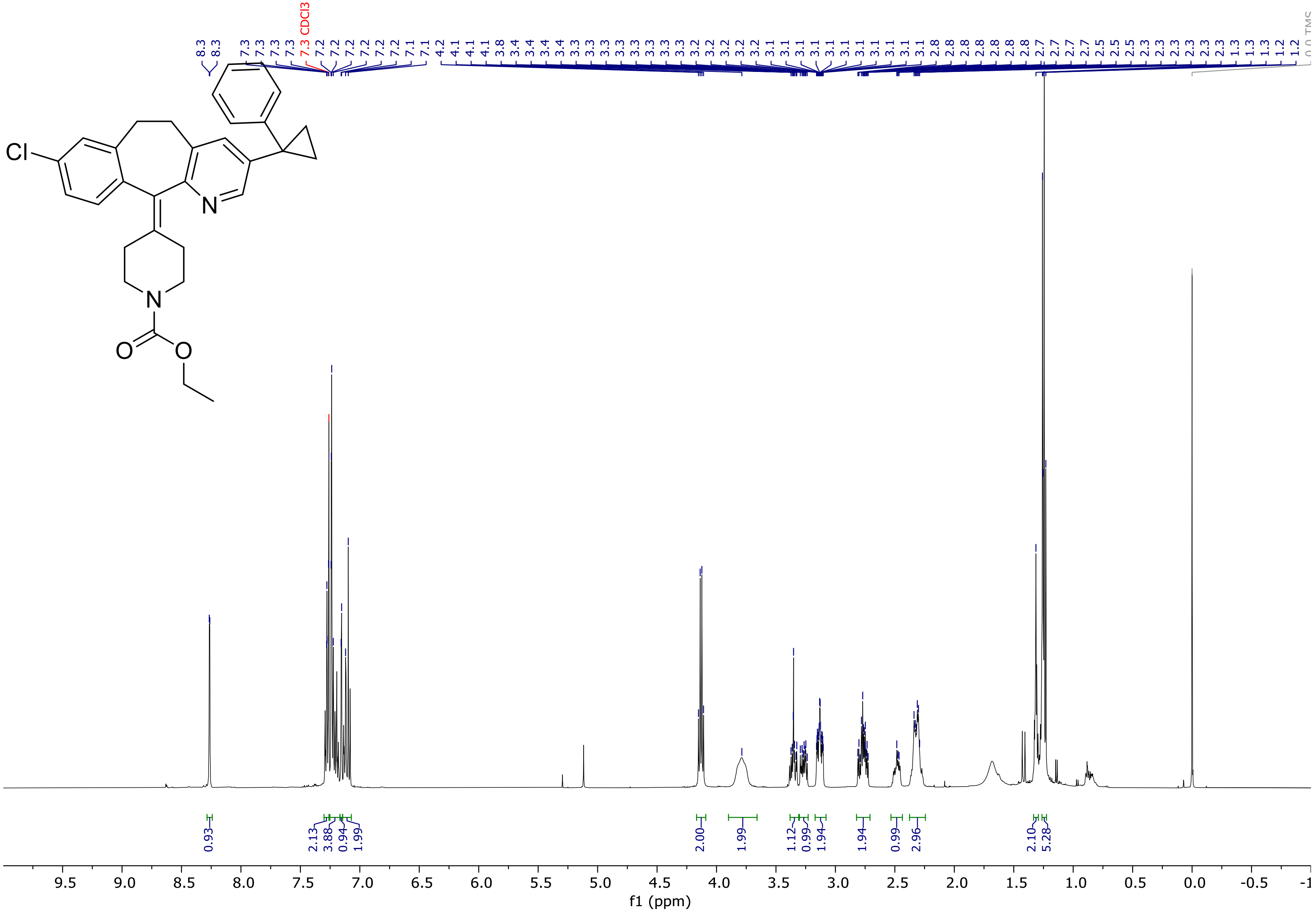

$^{13}C\{^1H\}$ NMR spectrum of compound 3al (126 MHz, $CDCl_3$)

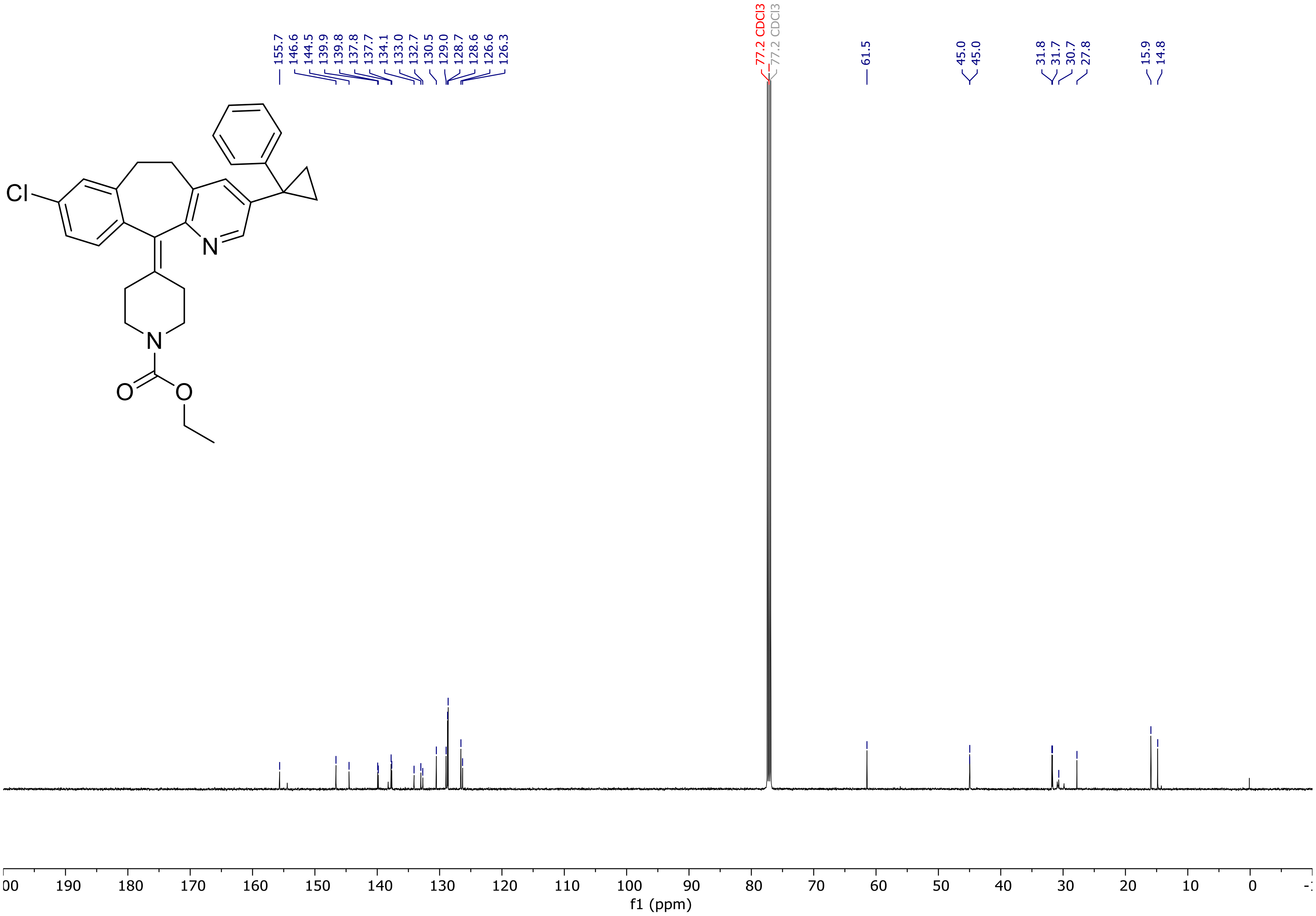

^{1}H NMR spectrum of compound ($^{t\text{-Bu}}$BpyCam^{CN})Ni(*o*-tol) (500 MHz, $CDCl_3$)

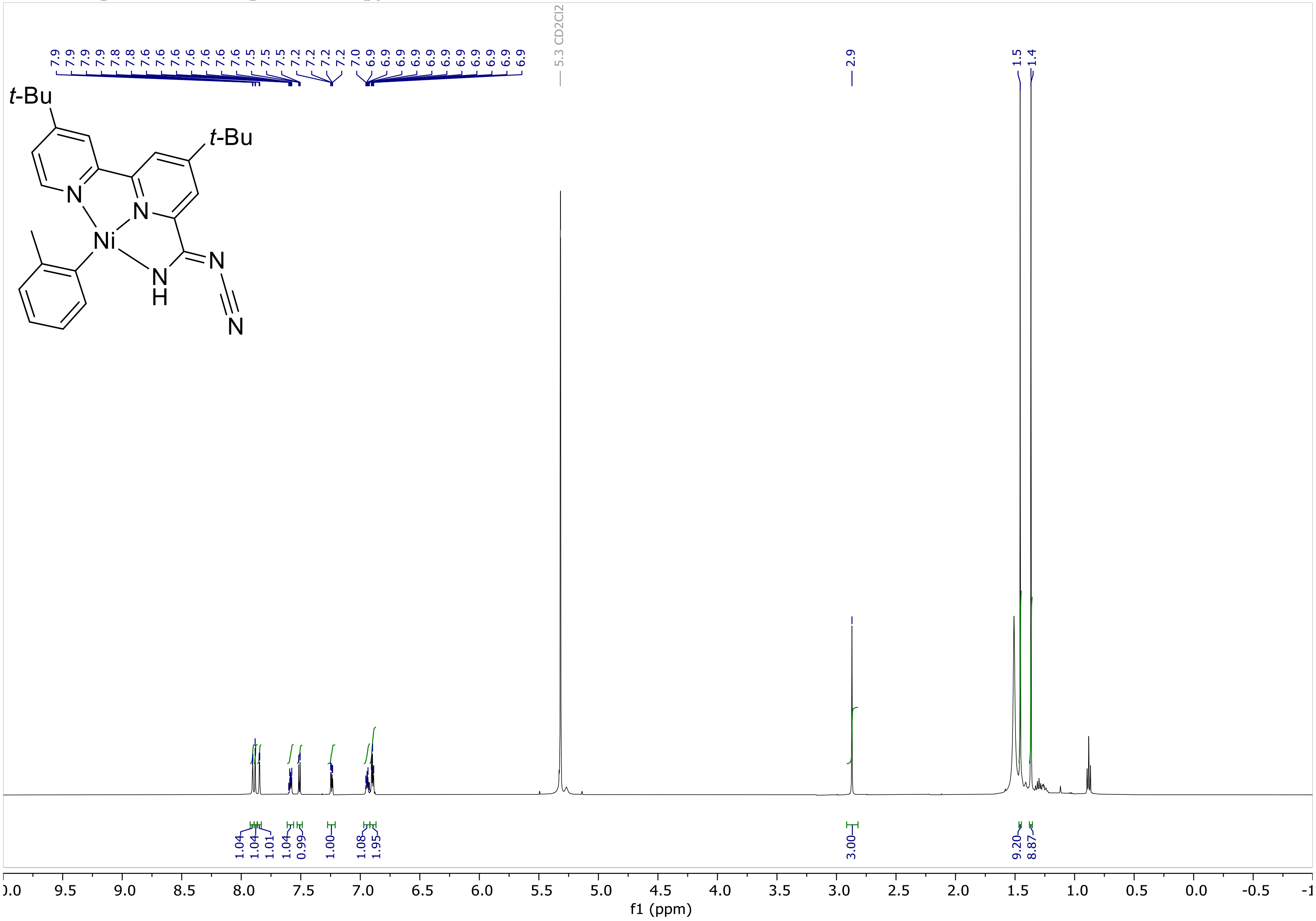

$^{13}C\{^{1}H\}$ NMR spectrum of compound ($^{t\text{-}Bu}BpyCam^{CN}$) (126 MHz, $CDCl_3$)

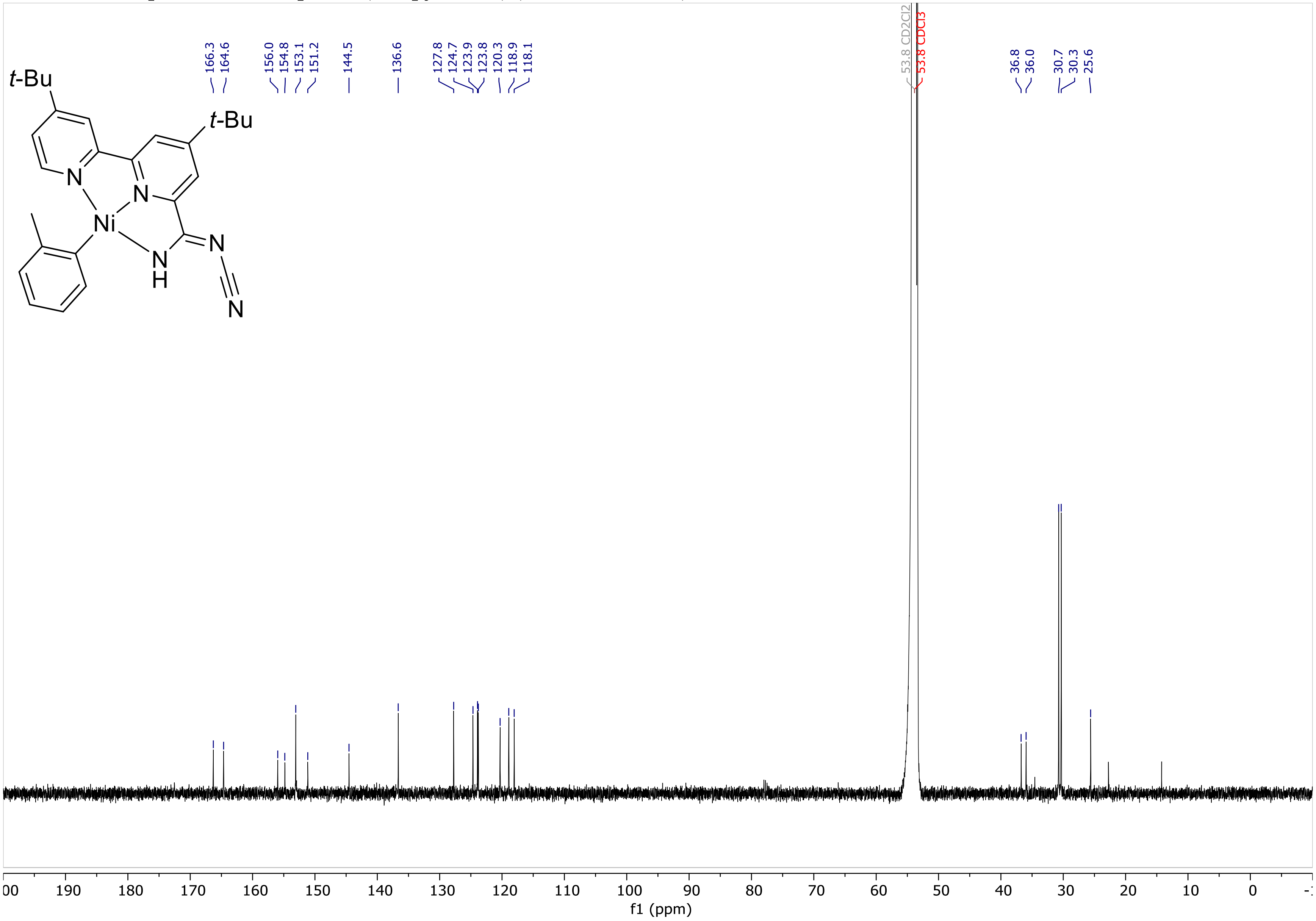

Supplement: Supplementary file 1 — Supporting Information [file ANIE-61-0-s001.pdf]
